# Supplementary material for: The Effect of Pre-Analytical Conditions on Blood Metabolomics in Epidemiological Studies
Source: Metabolites. 2019 Apr 3;9(4):64. doi: 10.3390/metabo9040064 (PMC6523923; doi:10.3390/metabo9040064)
Supplement: Supplementary file 1 [file metabolites-09-00064-s001.pdf]

metabolites

MDPI

Supplementary data

Article

The Effect of Pre-Analytical Conditions on Blood Metabolomics in Epidemiological Studies

Diana L. Santos Ferreira [1,2]*, Hannah J. Maple [1,2], Matt Goodwin [1,2], Judith S. Brand [1,2,3], Vikki Yip [2], Josine L. Min [1,2], Alix Groom [1,2], Debbie A. Lawlor [1,2,4] and Susan Ring [1,2]

1 Medical Research Council Integrative Epidemiology Unit at the University of Bristol, Bristol BS8 2BN, UK; diana.santosferreira@bristol.ac.uk (D.L.S.F.); hannah.maple@bio-techne.com (H.J.M); matt.goodwin@bristol.ac.uk (M.G.); judith.brand@bristol.ac.uk (J.S.B.); josine.min@bristol.ac.uk (J.L.M.); alix.groom@bristol.ac.uk (A.G.); d.a.lawlor@bristol.ac.uk (D.A.L.); s.m.ring@bristol.ac.uk (S.R.)
2 Population Health Sciences, Bristol Medical School, University of Bristol, Bristol BS8 2PS, UK; vikki.yip@bristol.ac.uk (V.Y.)
3 Clinical Epidemiology and Biostatistics, School of Medical Sciences, Örebro University, Örebro 701 85, Sweden
4 Bristol National Institute of Health Research Biomedical Research Centre, Bristol BS1 3NU, UK
* Correspondence: diana.santosferreira@bristol.ac.uk; Tel.: +44-117-331-0009

metabolites MDPI

Contents

Supplementary methods 4

Text S1. Multiple testing correction: Principal component analysis (PCA) based Bonferroni correction 4

Supplementary figures 5

Figure S1. Serum, pre-storage handling effects (spearman's rank correlation): 5

*Figure S1 (continued). Serum, pre-storage handling effects (spearman's rank correlation):*6

*Figure S1 (continued). Serum, pre-storage handling effects (spearman's rank correlation):*7

Figure S2. EDTA-plasma, pre-storage handling effects (spearman's rank correlation): 8

Figure S2 (continued). EDTA-plasma, pre-storage handling effects (spearman's rank correlation): 9

Figure S2 (continued). EDTA-plasma, pre-storage handling effects (spearman's rank correlation): 10

Figure S3. Serum, post-storage handling effects (spearman's rank correlation): 11

Figure S3 (continued). Serum, post-storage handling effects (spearman's rank correlation): 12

Figure S3 (continued). Serum, post-storage handling effects (spearman's rank correlation): 13

Figure S4. EDTA-plasma, post-storage handling effects (spearman's rank correlation):....14

Figure S4 (continued). EDTA-plasma, post-storage handling effects (spearman's rank correlation): 15

Figure S4 (continued). EDTA-plasma, post-storage handling effects (spearman's rank correlation): 16

Figure S5. Pre-storage handling effects (differences in mean levels): 17

Figure S5 (continued). Pre-storage handling effects (differences in mean levels):.............18

Figure S5 (continued). Pre-storage handling effects (differences in mean levels):.............19

Figure S5 (continued). Pre-storage handling effects (differences in mean levels):.............20

Figure S5 (continued). Pre-storage handling effects (differences in mean levels):.............21

Figure S6. Post-storage handling effects (differences in mean levels): 22

Figure S6 (continued). Post -storage handling effects (differences in mean levels):...........23

Figure S6 (continued). Post -storage handling effects (differences in mean levels):...........24

Figure S6 (continued). Post -storage handling effects (differences in mean levels):...........25

Figure S6 (continued). Post -storage handling effects (differences in mean levels):...........26

Figure S7-Principal Component Analysis (PCA) on serum non-lipid-related metabolic traits subjected to five different pre-storage conditions. 27

Supplementary tables 29

Table S1. Characteristics of metabolic traits 29

Table S2. Spearman's correlation: serum, pre-storage handling effects.............................41
Table S3. Spearman's correlation: EDTA-plasma, pre-storage handling effects.................63
Table S4. Spearman's correlation: serum, post-storage handling effects...........................85
Table S5. Spearman's correlation: EDTA-plasma, post-storage handling effects...............97
Table S6. Serum, pre-storage handling effects (differences in mean levels):....................109
Table S7. EDTA-Plasma, pre-storage handling effects (differences in mean levels):.........122
Table S8. Serum, post-storage handling effects (differences in mean levels):..................135
Table S9. EDTA-plasma, post-storage handling effects (differences in mean levels):.......148
Table S10. Literature table..161
References 178

metabolites

Supplementary methods

Text S1. Multiple testing correction: Principal component analysis (PCA) based Bonferroni correction

Principal component analysis (PCA) was performed separately on each serum and plasma datasets and included samples subjected to reference and *pre*-storage variant conditions. In each dataset, all individuals who had data on all metabolic traits were used and PCA was performed on the z-scored metabolic data. This method assumes that the independence of the principal components (PCs) is equivalent to the number of independent testes of the original metabolic dataset, and that retaining a number of PCs that is enough to explain at least 95% of the variance will only result in a small chance of a type 1 error [1]. Since the number of variables available varies across serum and plasma datasets (151 and 148 metabolic traits, respectively) and both metabolomes are slightly different, the number of PCs needed to explain 95% of the variation in the metabolic traits also varies. The PCA results are as follows, serum, 8 PCs and plasma, 9 PCs. The highest number (9 PCs) was used as a conservative estimate of the number of independent tests been performed. Therefore, the threshold of *p*-value <0.05 becomes *p*-value <0.006 (i.e. $\alpha/9$ where $\alpha=0.05$), when multiple testing is considered, for assessing associations of up to 151 metabolic traits. Number of PCs obtained using only samples subjected to the reference conditions were very similar.

metabolites

MDPI

Supplementary figures

Figure S1. Serum, pre-storage handling effects (Spearman's rank correlation): Spearman's rank correlation coefficients between lipoprotein particle and lipid concentration in reference samples (4 °C, 1.5 h) and samples incubated at (i) 4 °C, 24 h; (ii) 4 °C, 48 h; (iii) 21 °C, 24 h; (iv) 21 °C, 48 h, before centrifugation (correlations for other metabolic traits are given in Figure 1-2). Spearman's rank correlation coefficients and 95% confidence intervals are listed in Tables S2.

***Abbreviations**: **VLDL**=very-low-density lipoprotein.*

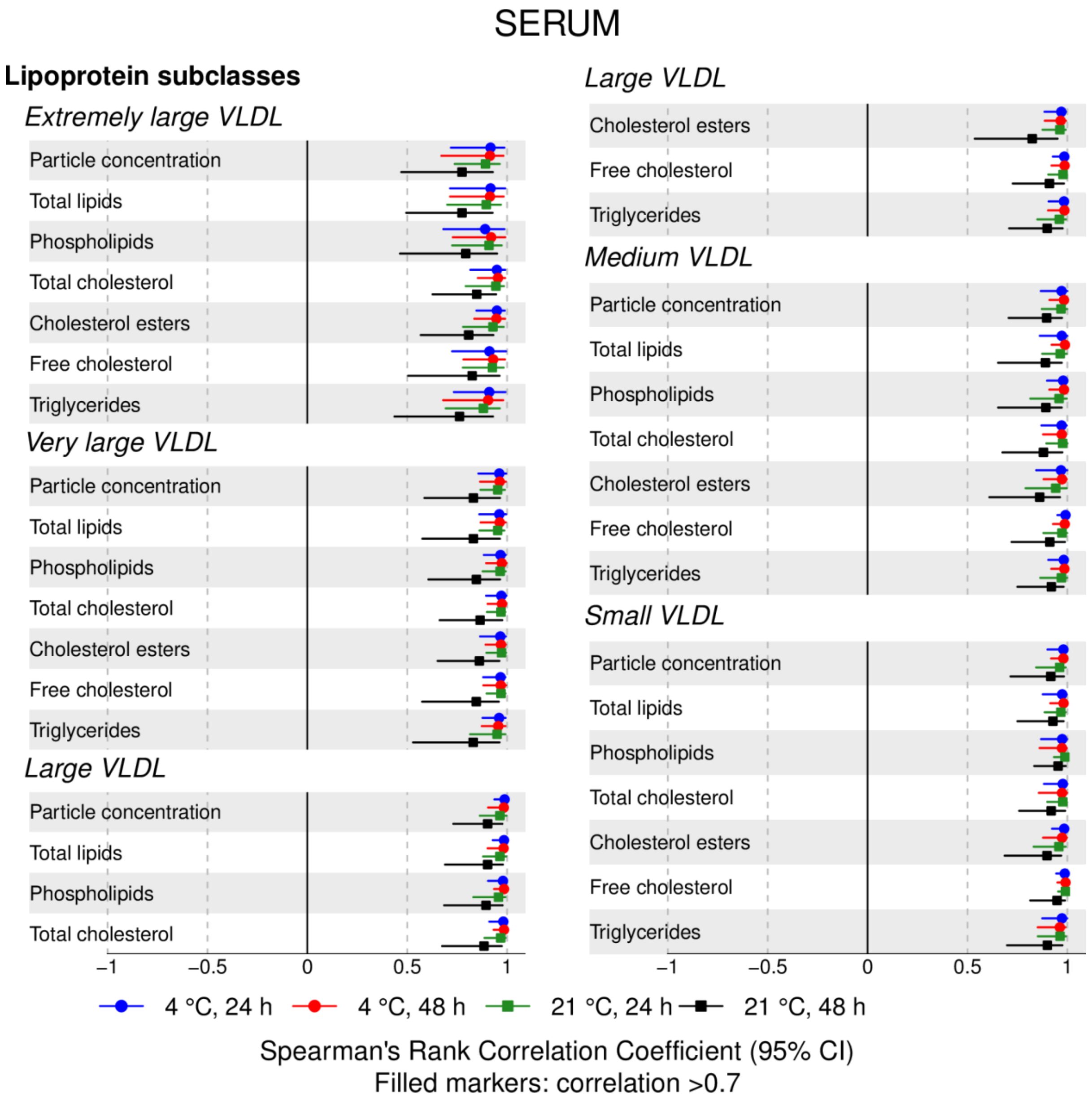

metabolites MDPI

Figure S1 (continued). Serum, pre-storage handling effects (Spearman's rank correlation): Spearman's rank correlation coefficients between lipoprotein particle and lipid concentration in reference samples (4 °C, 1.5 h) and samples incubated at (i) 4 °C, 24 h; (ii) 4 °C, 48 h; (iii) 21 °C, 24 h; (iv) 21 °C, 48 h, before centrifugation (correlations for other metabolic traits are given in Figure 1-2). Spearman's rank correlation coefficients and 95% confidence intervals are listed in Tables S2.

Abbreviations: ***IDL***=*intermediate-density lipoprotein*; ***LDL***=*low-density lipoprotein*; ***VLDL***=*very-low-density lipoprotein.*

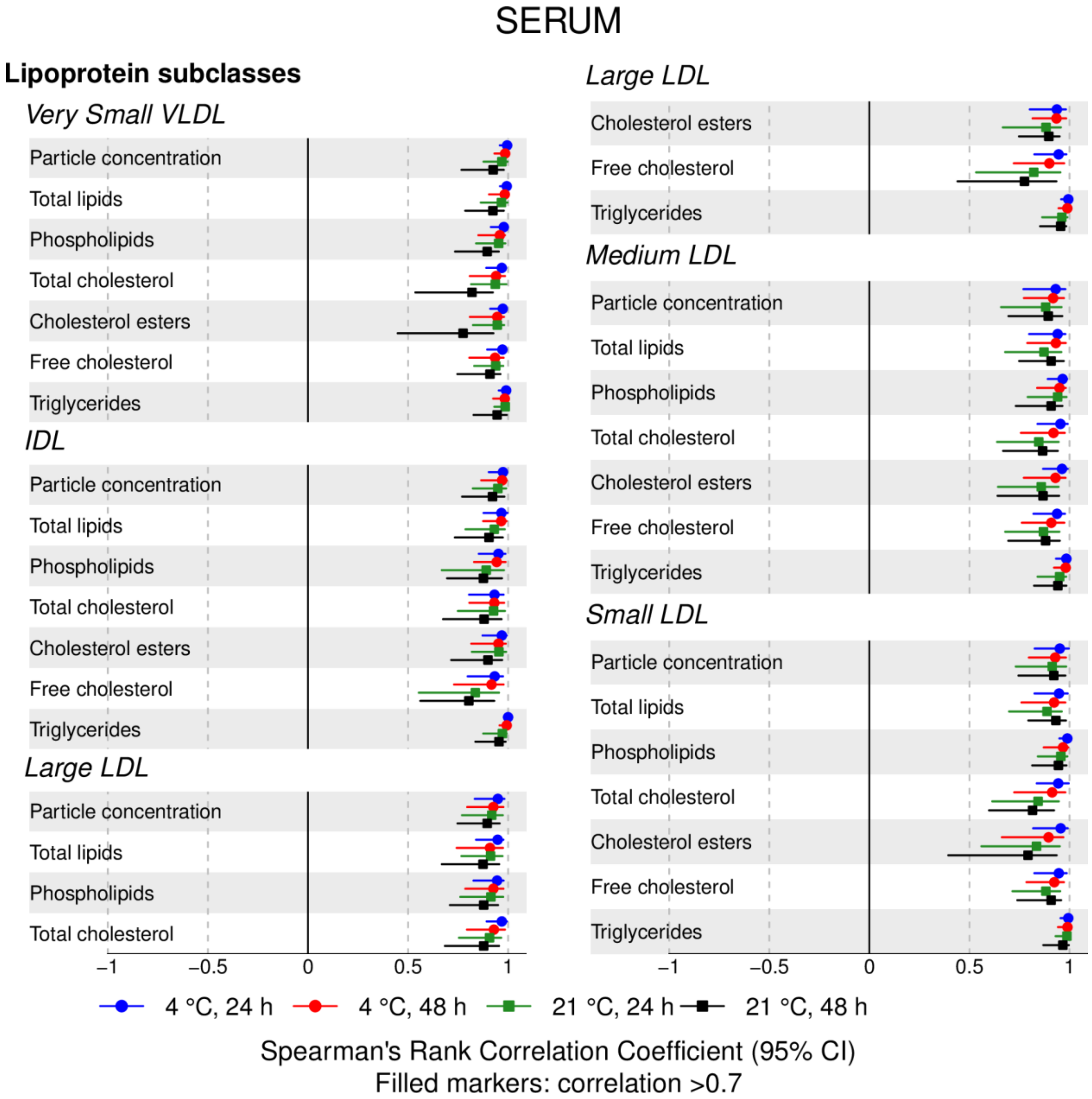

metabolites MDPI

Figure S1 (continued). Serum, pre-storage handling effects (Spearman's rank correlation): Spearman's rank correlation coefficients between lipoprotein particle and lipid concentration in reference samples (4 °C, 1.5 h) and samples incubated at (i) 4 °C, 24 h; (ii) 4 °C, 48 h; (iii) 21 °C, 24 h; (iv) 21 °C, 48 h, before centrifugation (correlations for other metabolic traits are given in Figure 1-2). Spearman's rank correlation coefficients and 95% confidence intervals are listed in Tables S2.

***Abbreviations**: **HDL**=high-density lipoprotein.*

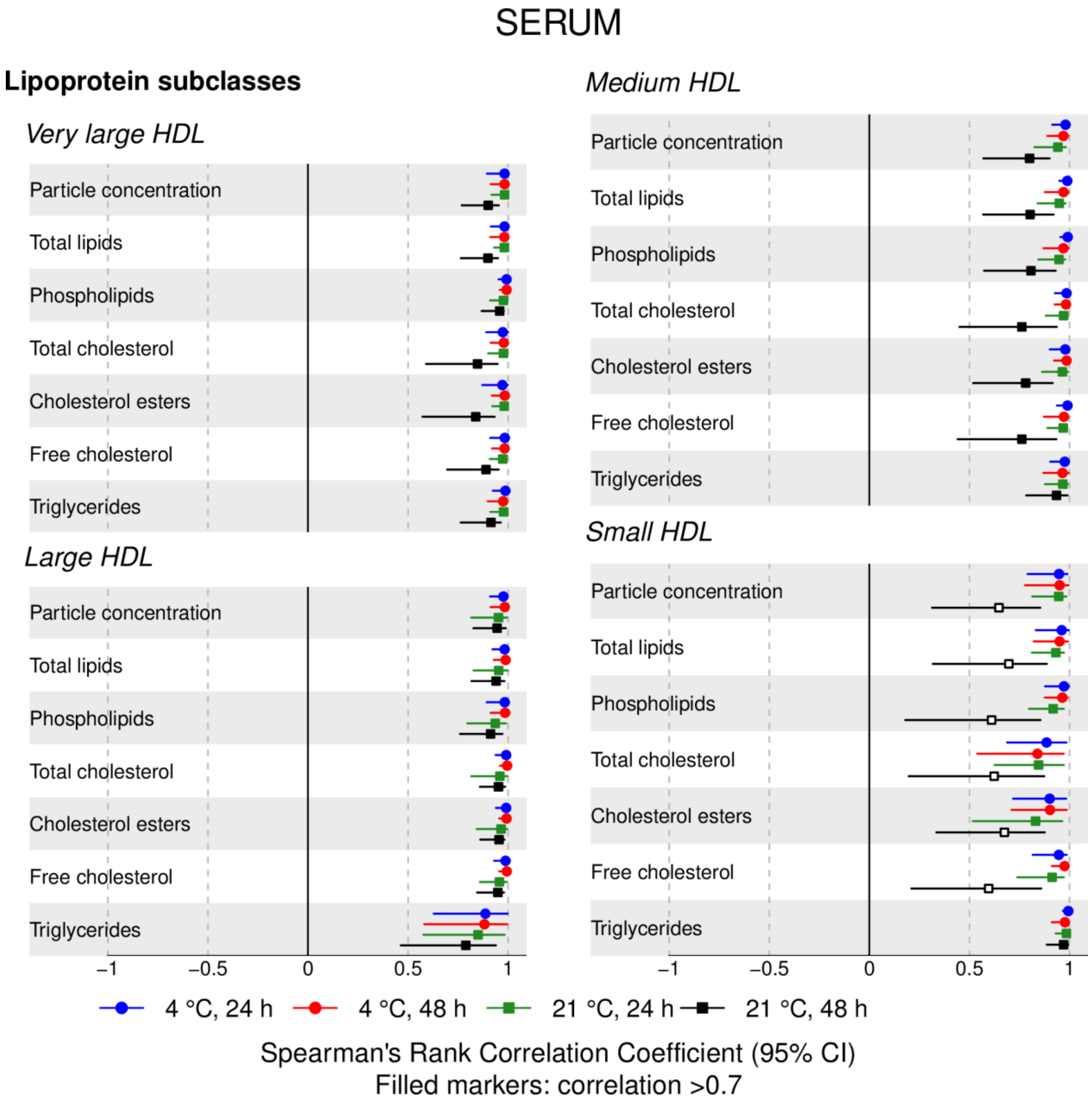

metabolites MDPI

Figure S2. EDTA-plasma, pre-storage handling effects (Spearman's rank correlation): Spearman's rank correlation coefficients between lipoprotein particle and lipid concentration in reference samples (4°C, 1.5h) and samples incubated at (i) 4 °C, 24 h; (ii) 4 °C, 48 h; (iii) 21 °C, 24 h; (iv) 21 °C, 48 h, before centrifugation (correlations for other metabolic traits are given in Figure 3-4). Spearman's rank correlation coefficients and 95% confidence intervals are listed in Tables S3.

***Abbreviations**: **VLDL**=very-low-density lipoprotein.*

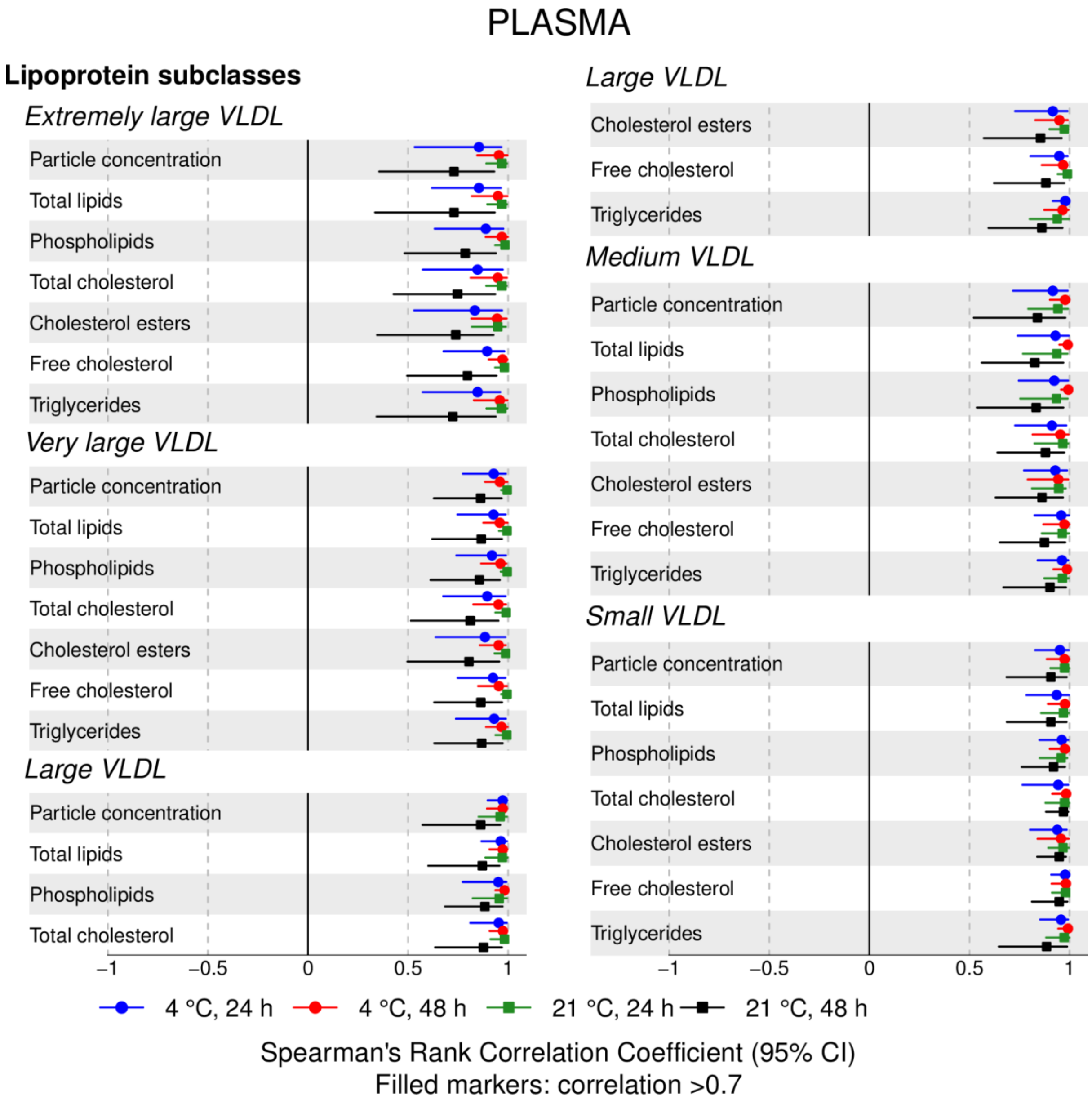

Figure S2 (continued). EDTA-plasma, pre-storage handling effects (Spearman's rank correlation): Spearman's rank correlation coefficients between lipoprotein particle and lipid concentration in reference samples (4 °C, 1.5 h) and samples incubated at (i) 4 °C, 24 h; (ii) 4 °C, 48 h; (iii) 21 °C, 24 h; (iv) 21 °C, 48 h, before centrifugation (correlations for other metabolic traits are given in Figure 3-4). Spearman's rank correlation coefficients and 95% confidence intervals are listed in Tables S3.

***Abbreviations**: **IDL**=intermediate-density lipoprotein; **LDL**=low-density lipoprotein; **VLDL**=very-low-density lipoprotein.*

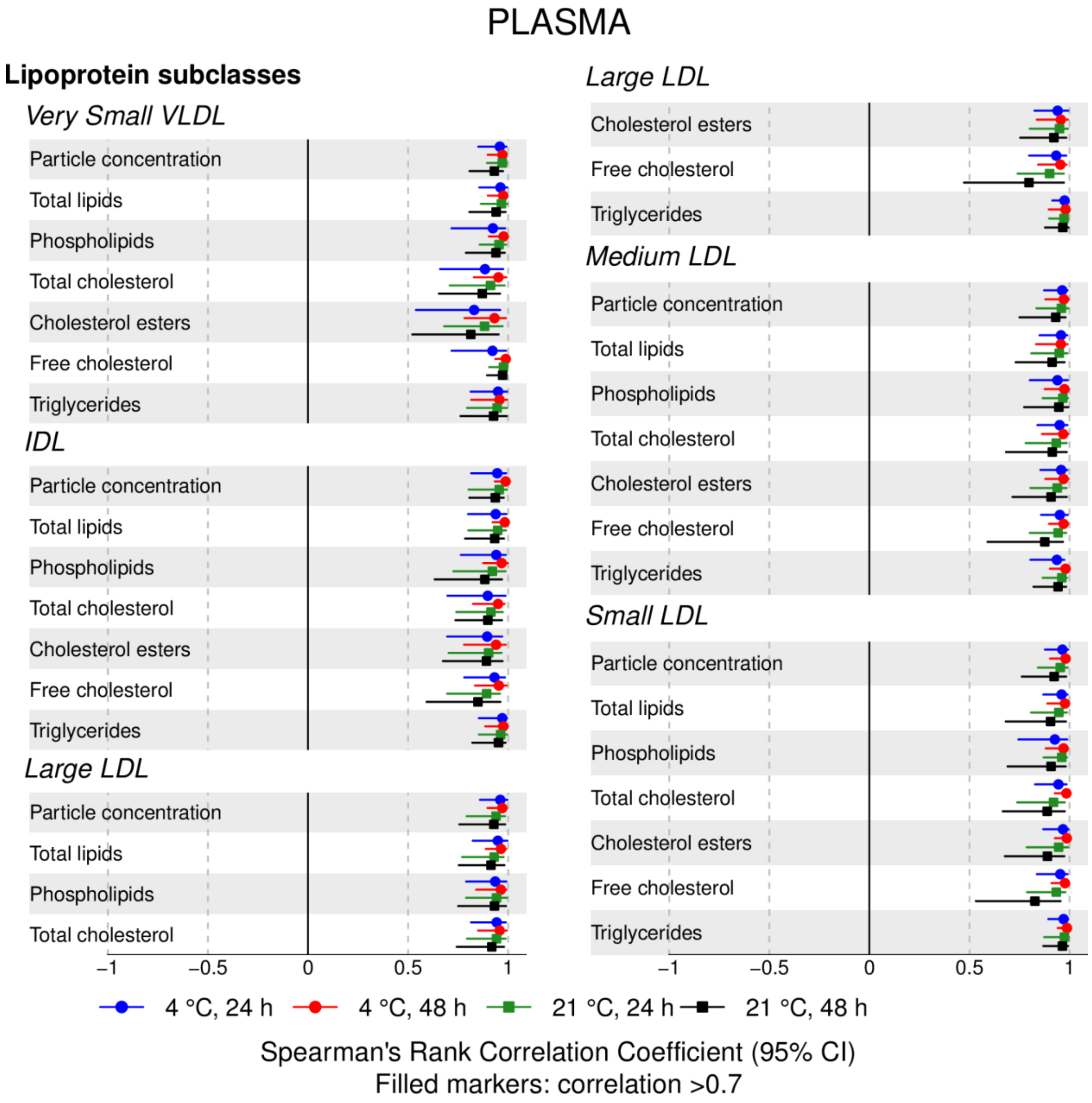

Figure S2 (continued). EDTA-plasma, pre-storage handling effects (Spearman's rank correlation): Spearman's rank correlation coefficients between lipoprotein particle and lipid concentration in reference samples (4 °C, 1.5 h) and samples incubated at (i) 4 °C, 24 h; (ii) 4 °C, 48 h; (iii) 21 °C, 24 h; (iv) 21 °C, 48 h, before centrifugation (correlations for other metabolic traits are given in Figure 3-4). Spearman's rank correlation coefficients and 95% confidence intervals are listed in Tables S3.

***Abbreviations**: **HDL**=high-density lipoprotein.*

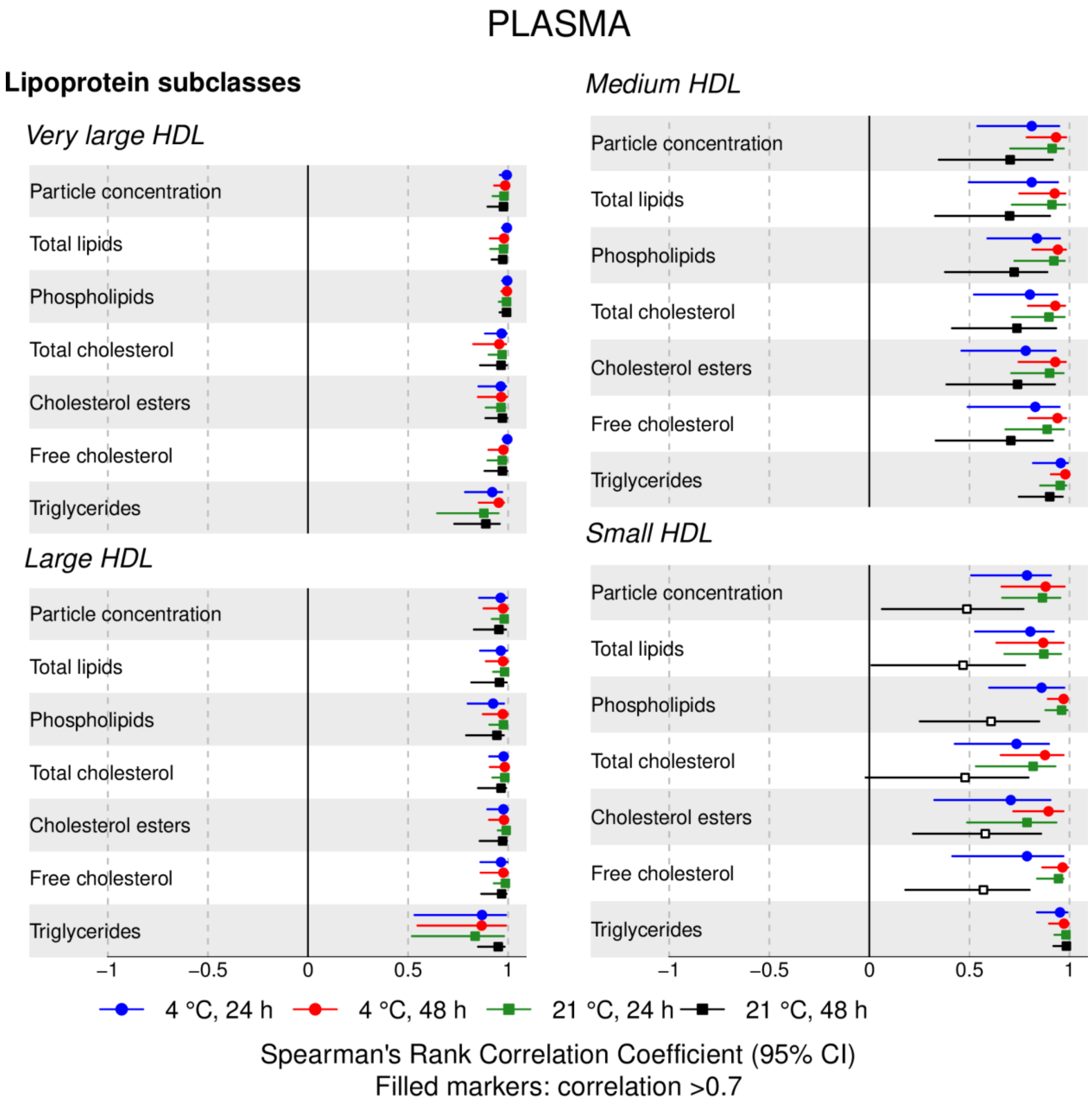

Figure S3. Serum, post-storage handling effects (Spearman's rank correlation): Spearman's rank correlation coefficients between lipoprotein particle and lipid concentration in reference samples (no buffer addition delay or Nuclear Magnetic Resonance (NMR)-analysis delay) and sample subjected to two variant post-storage conditions in which samples thawed overnight and afterwards (i) were left for 24 h before addition of sodium buffer followed by immediate NMR analysis (buffer delay); (ii) addition of sodium buffer, then left for 24 h before NMR profiling (NMR delay) (correlations for other metabolic traits are given in Figure 5-6). Spearman's rank correlation coefficients and 95% confidence intervals are listed in Tables S4. ***Abbreviations***: ***VLDL***=*very-low-density lipoprotein.*

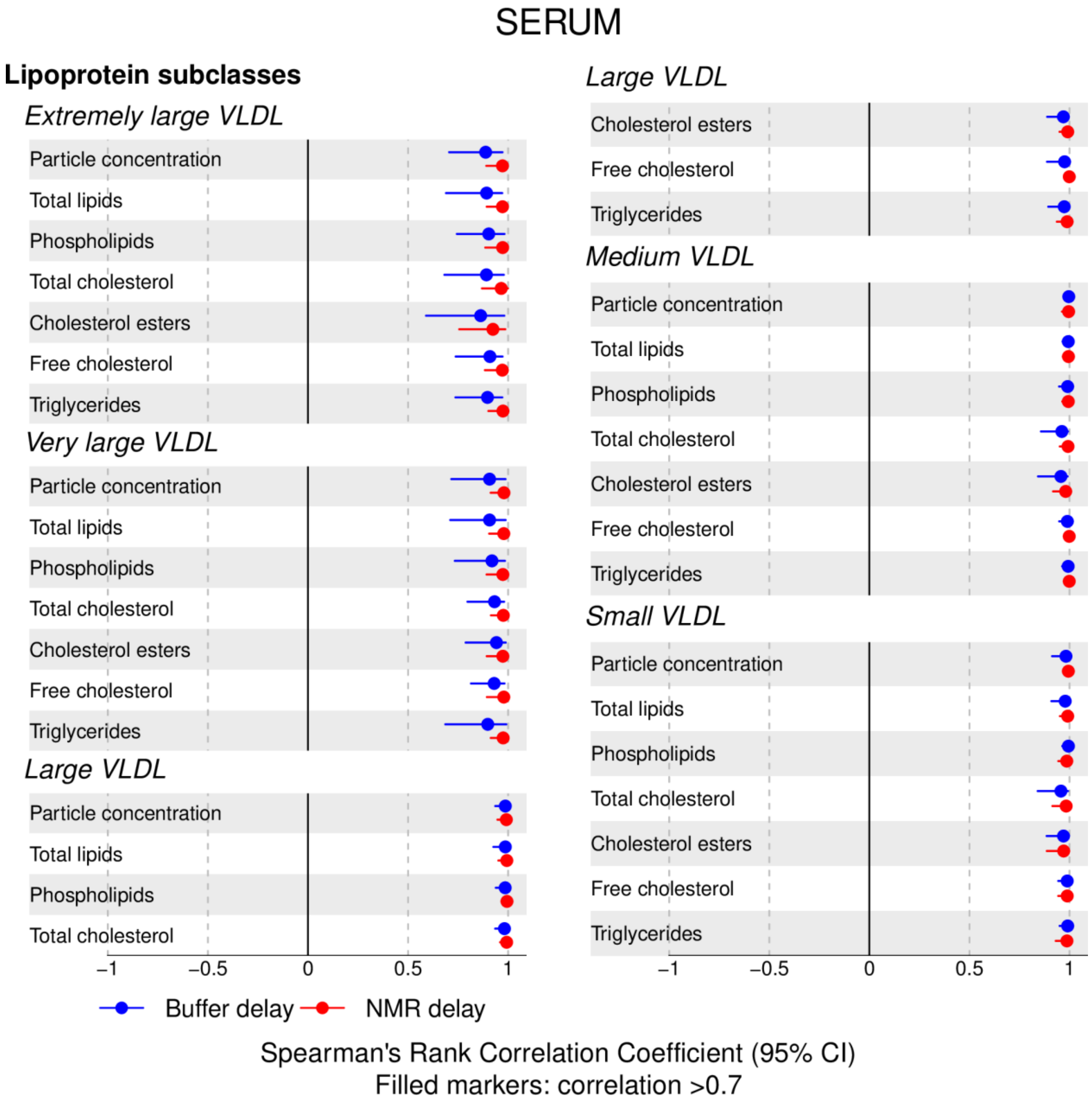

Figure S3 (continued). Serum, post-storage handling effects (Spearman's rank correlation): Spearman's rank correlation coefficients between lipoprotein particle and lipid concentration in reference samples (no buffer addition delay or Nuclear Magnetic Resonance (NMR)-analysis delay) and sample subjected to two variant post-storage conditions in which samples thawed overnight and afterwards (i) were left for 24 h before addition of sodium buffer followed by immediate NMR analysis (buffer delay); (ii) addition of sodium buffer, then left for 24 h before NMR profiling (NMR delay) (correlations for other metabolic traits are given in Figure 5-6). Spearman's rank correlation coefficients and 95% confidence intervals are listed in Tables S4.

***Abbreviations**: **IDL**=intermediate-density lipoprotein; **LDL**=low-density lipoprotein; **VLDL**=very-low-density lipoprotein.*

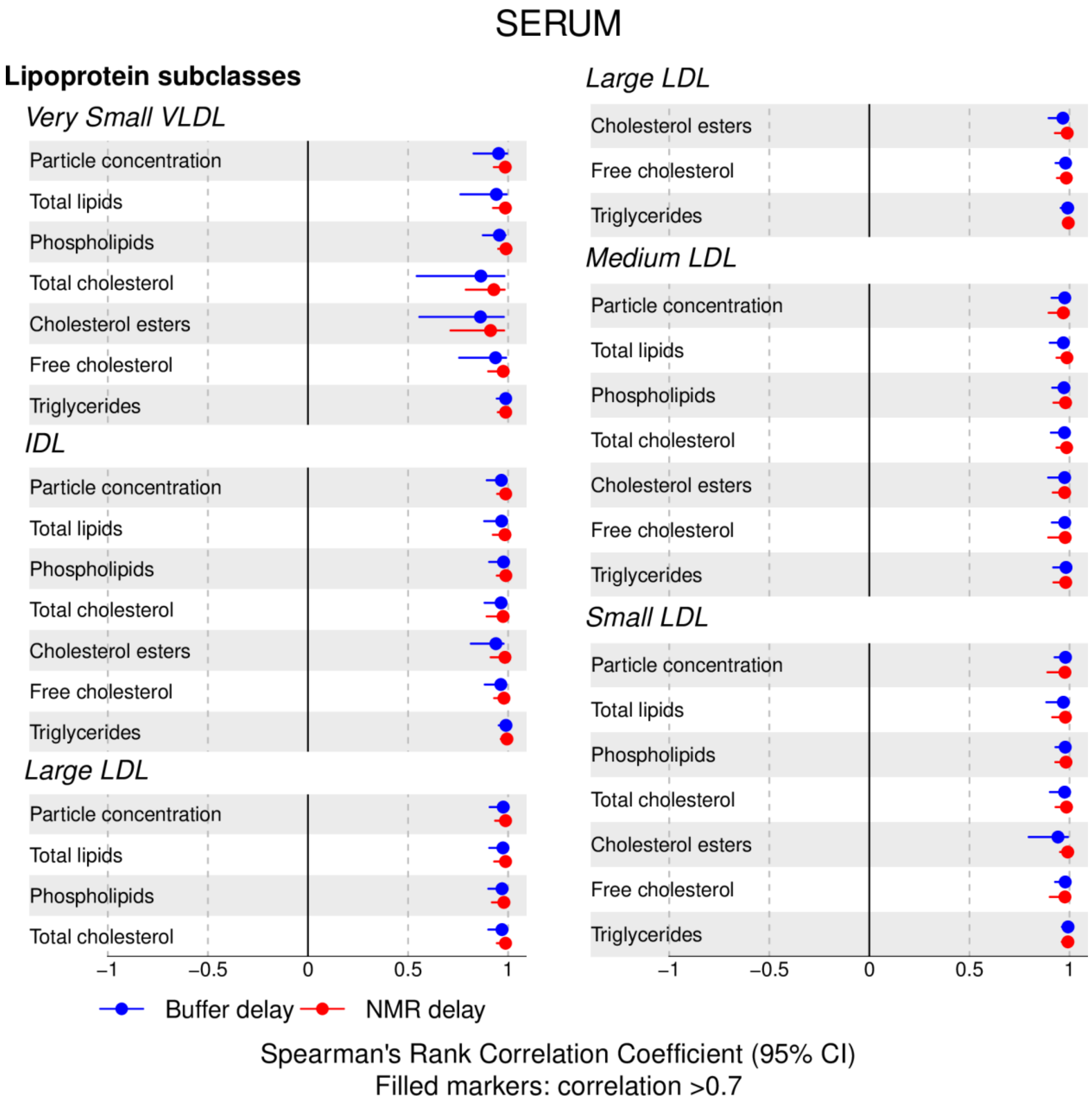

Figure S3 (continued). Serum, post-storage handling effects (Spearman's rank correlation): Spearman's rank correlation coefficients between lipoprotein particle and lipid concentration in reference samples (no buffer addition delay or Nuclear Magnetic Resonance (NMR)-analysis delay) and sample subjected to two variant post-storage conditions in which samples thawed overnight and afterwards (i) were left for 24 h before addition of sodium buffer followed by immediate NMR analysis (buffer delay); (ii) addition of sodium buffer, then left for 24 h before NMR profiling (NMR delay) (correlations for other metabolic traits are given in Figure 5-6). Spearman's rank correlation coefficients and 95% confidence intervals are listed in Tables S4.

***Abbreviations**: **HDL**=high-density lipoprotein.*

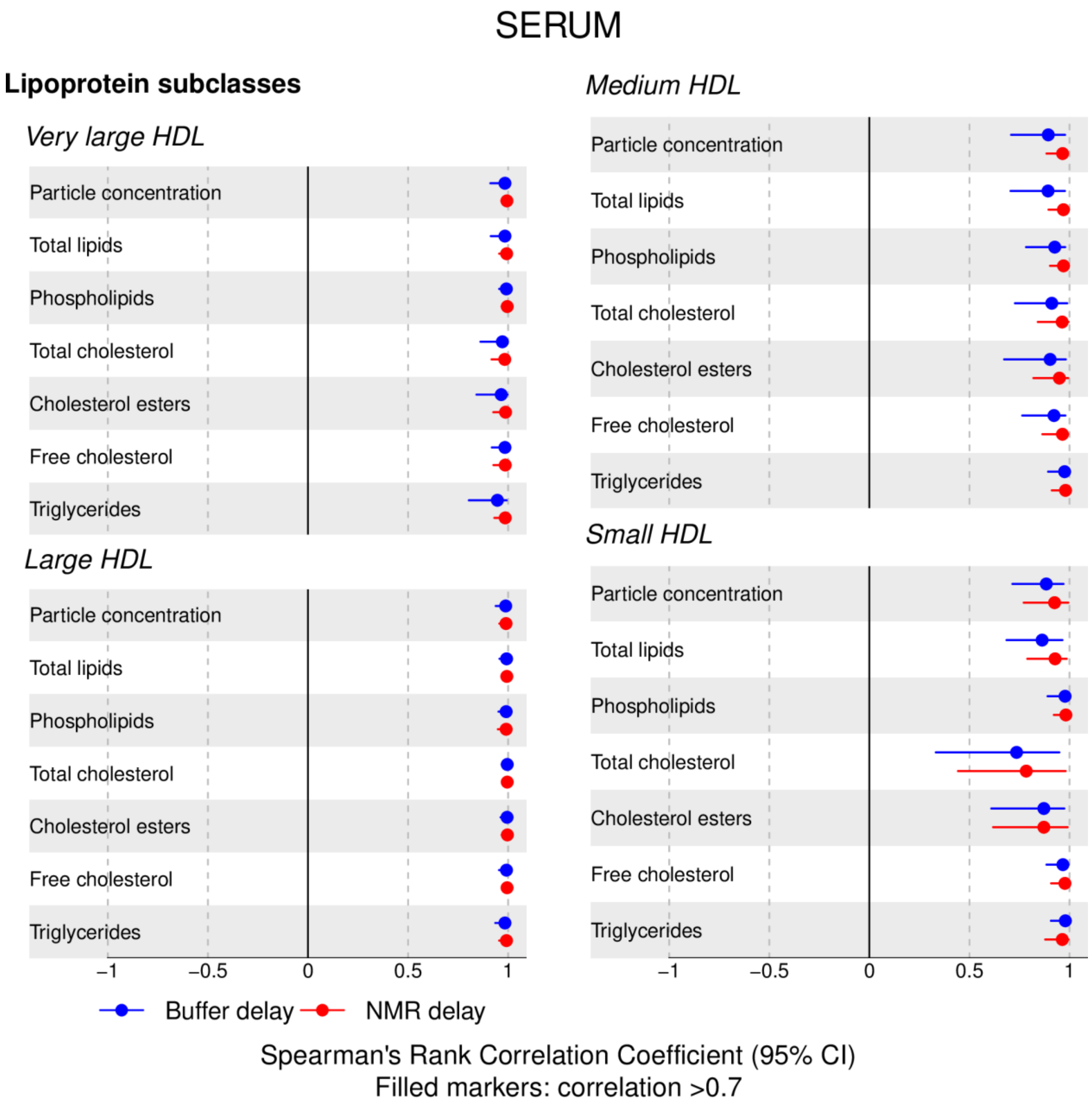

Figure S4. EDTA-plasma, post-storage handling effects (Spearman's rank correlation): Spearman's rank correlation coefficients between lipoprotein particle and lipid concentration in reference samples (no buffer addiction delay or Nuclear Magnetic Resonance (NMR)-analysis delay) and sample subjected to two variant post-storage conditions in which samples thawed overnight and afterwards (i) were left for 24 h before addition of sodium buffer followed by immediate NMR analysis (buffer delay); and (ii) addition of sodium buffer, then left for 24 h before NMR profiling (NMR delay) (correlations for other metabolic traits are given in Figure 7-8). Spearman's rank correlation coefficients and 95% confidence intervals are listed in Tables S5.

***Abbreviations**: **VLDL**=very-low-density lipoprotein.*

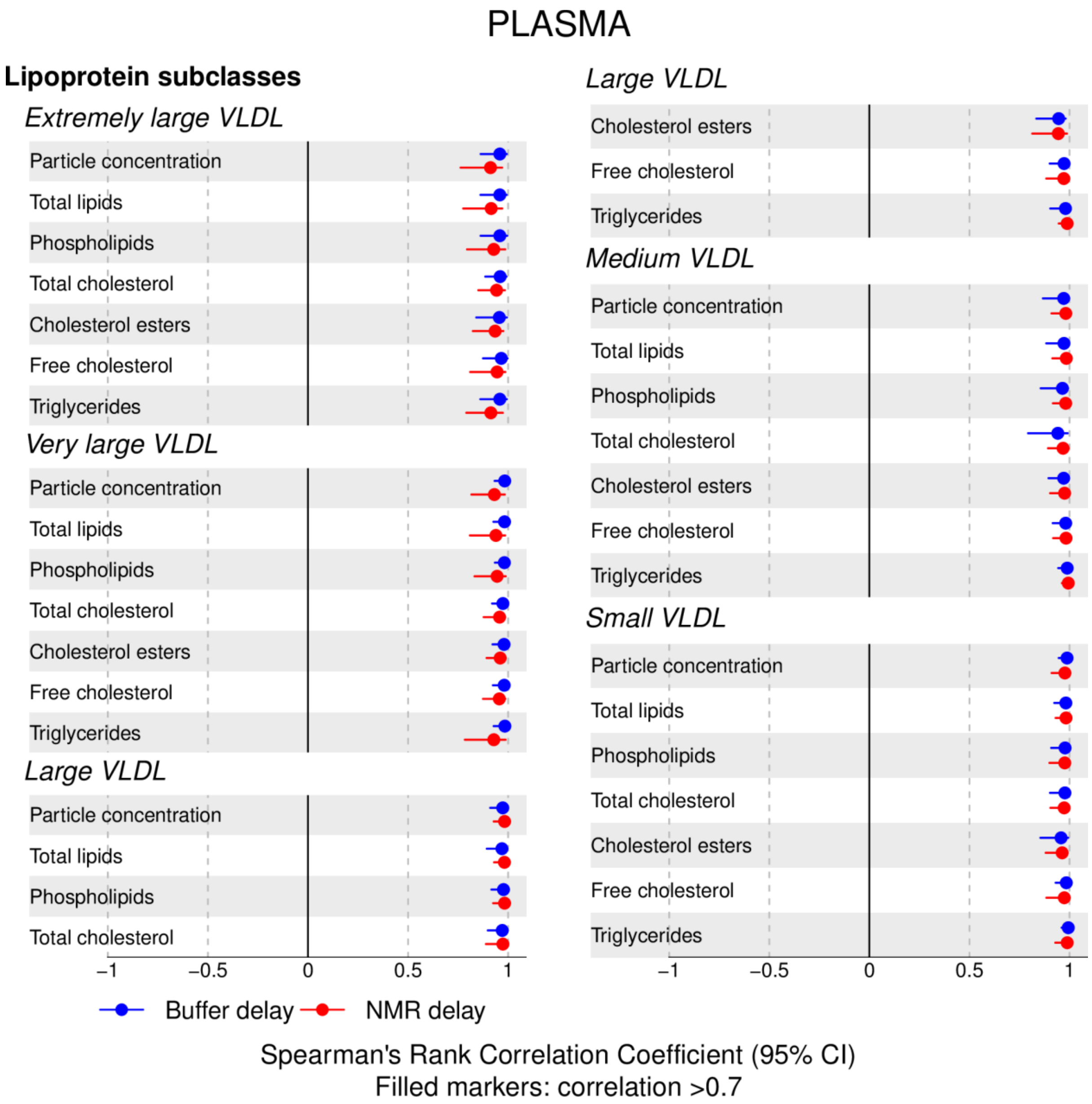

Figure S4 (continued). EDTA-plasma, post-storage handling effects (spearman's rank correlation): Spearman's rank correlation coefficients between lipoprotein particle and lipid concentration in reference samples (no buffer addiction delay or Nuclear Magnetic Resonance (NMR)-analysis delay) and sample subjected to two variant post-storage conditions in which samples thawed overnight and afterwards (i) were left for 24 h before addition of sodium buffer followed by immediate NMR analysis (buffer delay); and (ii) addition of sodium buffer, then left for 24 h before NMR profiling (NMR delay) (correlations for other metabolic traits are given in Figure 7-8). Spearman's rank correlation coefficients and 95% confidence intervals are listed in Tables S5.

***Abbreviations**: **IDL**=intermediate-density lipoprotein; **LDL**=low-density lipoprotein; **VLDL**=very-low-density lipoprotein.*

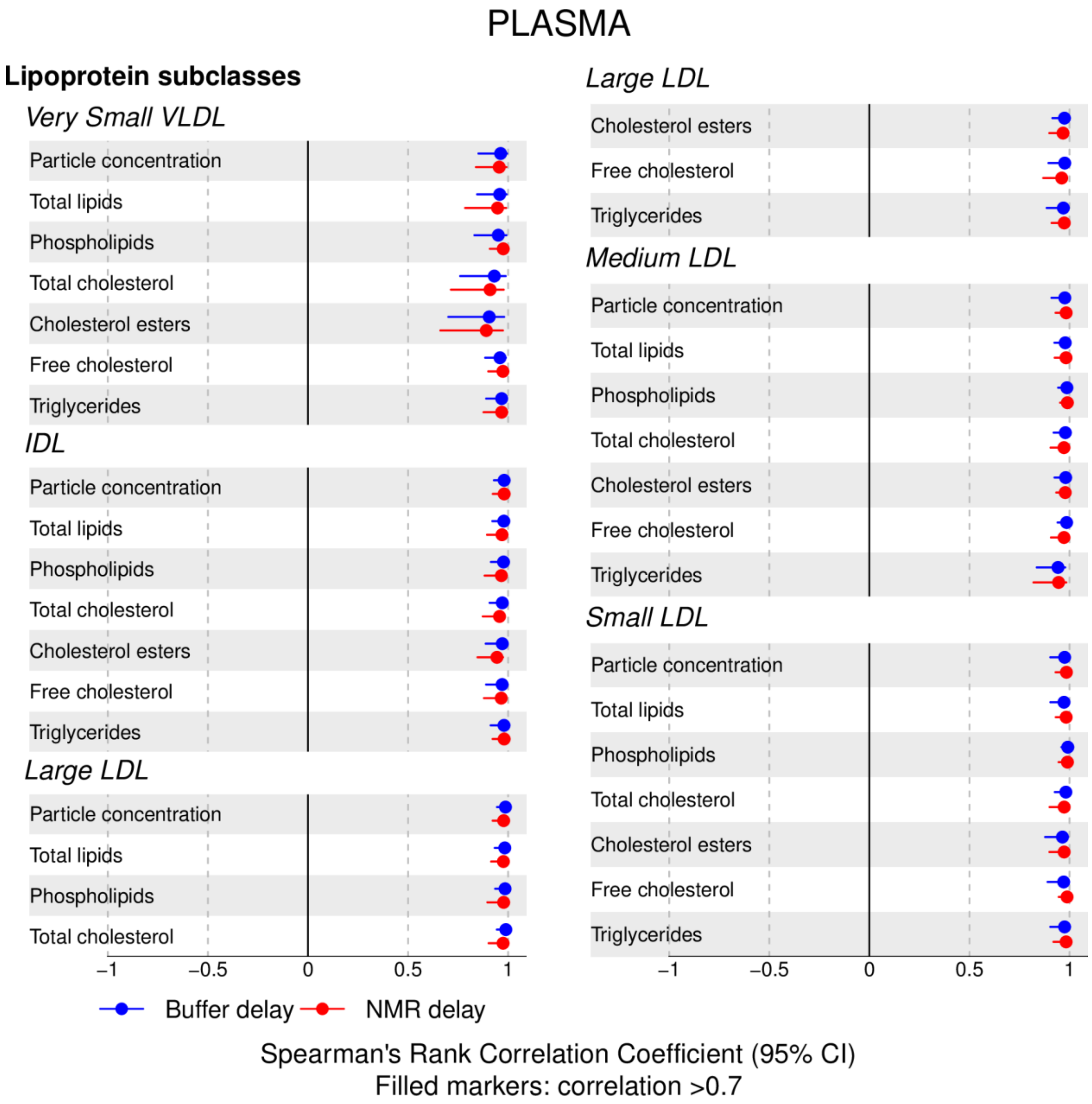

Figure S4 (continued). EDTA-plasma, post-storage handling effects (spearman's rank correlation): Spearman's rank correlation coefficients between lipoprotein particle and lipid concentration in reference samples (no buffer addiction delay or Nuclear Magnetic Resonance (NMR)-analysis delay) and sample subjected to two variant post-storage conditions in which samples thawed overnight and afterwards (i) were left for 24 h before addition of sodium buffer followed by immediate NMR analysis (buffer delay); and (ii) addition of sodium buffer, then left for 24 h before NMR profiling (NMR delay) (correlations for other metabolic traits are given in Figure 7-8). Spearman's rank correlation coefficients and 95% confidence intervals are listed in Tables S5.

***Abbreviations**: **HDL**=high-density lipoprotein.*

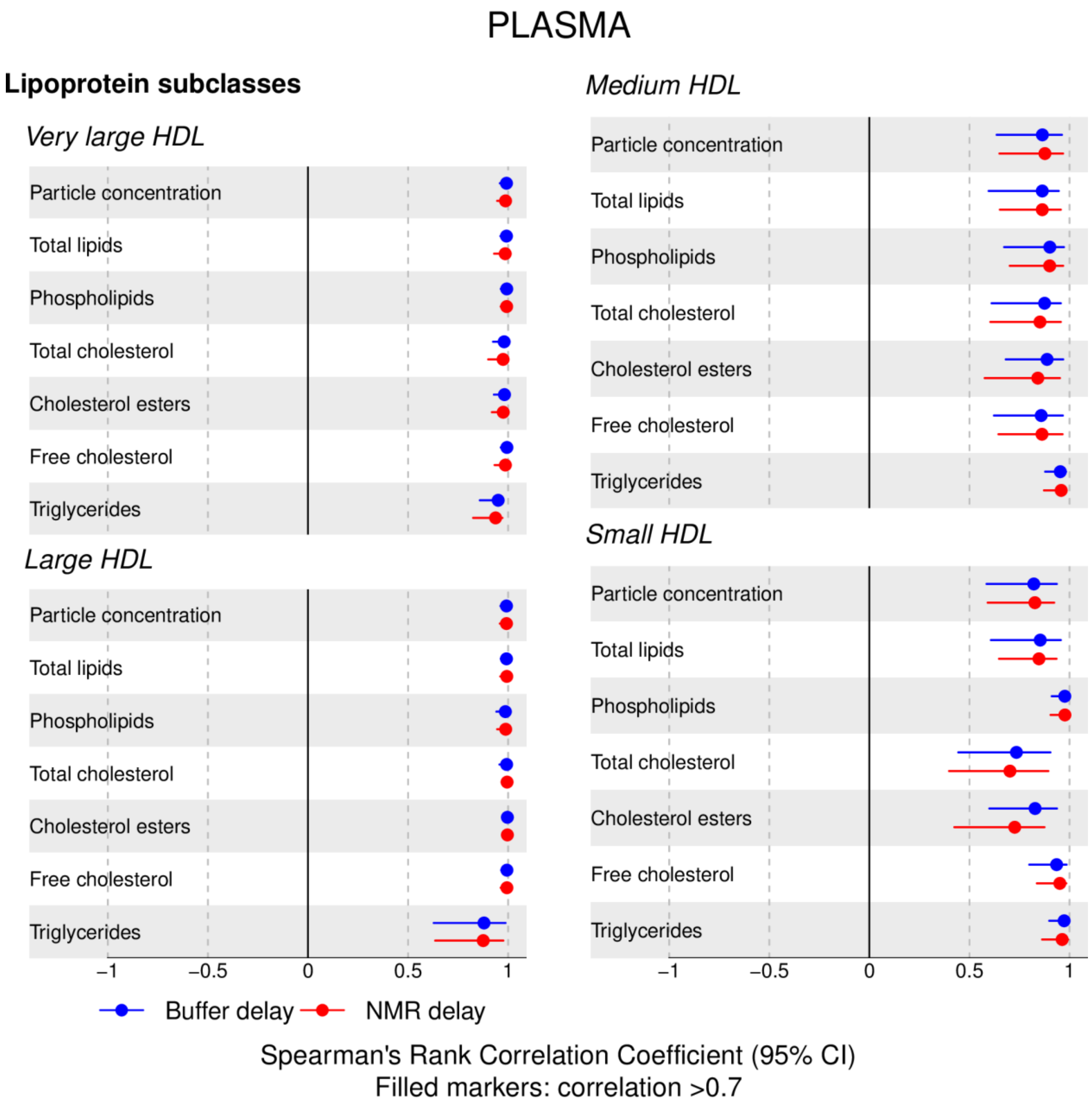

Figure S5. Pre-storage handling effects (differences in mean levels): standardized mean differences in metabolic traits concentration, in serum and EDTA-plasma, per 24 h increment in incubation duration at 4 °C and 21 °C. Mean differences in absolute units are listed in Tables S6 and S7.

***Abbreviations**: **VLDL**=very-low-density lipoprotein.*

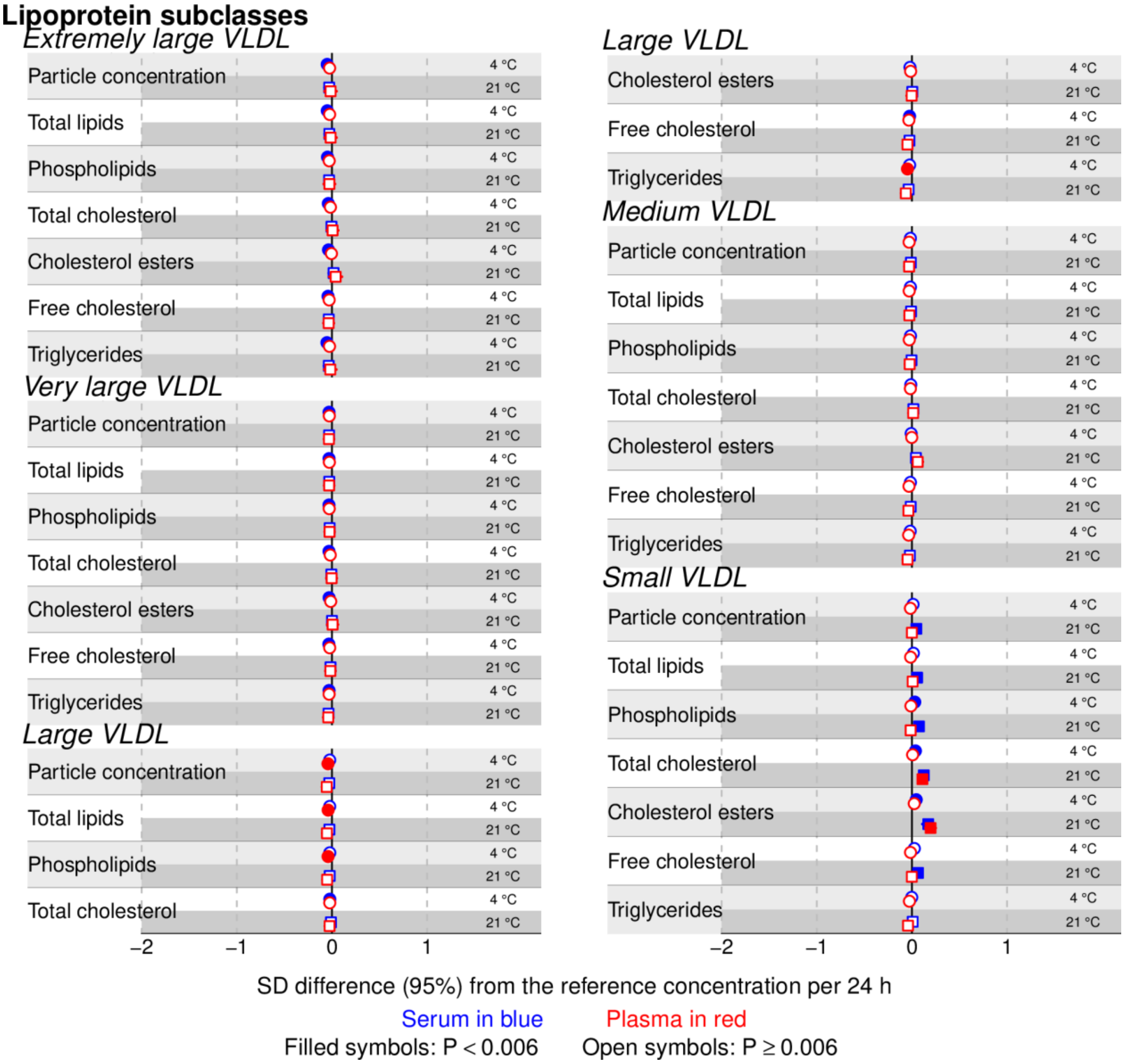

Figure S5 (continued). Pre-storage handling effects (differences in mean levels): standardized mean differences in metabolic traits concentration, serum and EDTA-plasma, per 24 h increment in incubation duration at 4 °C and 21 °C. Mean differences in absolute units are listed in Tables S6 and S7.

***Abbreviations**: **IDL**=intermediate-density lipoprotein; **LDL**=low-density lipoprotein; **VLDL**=very-low-density lipoprotein.*

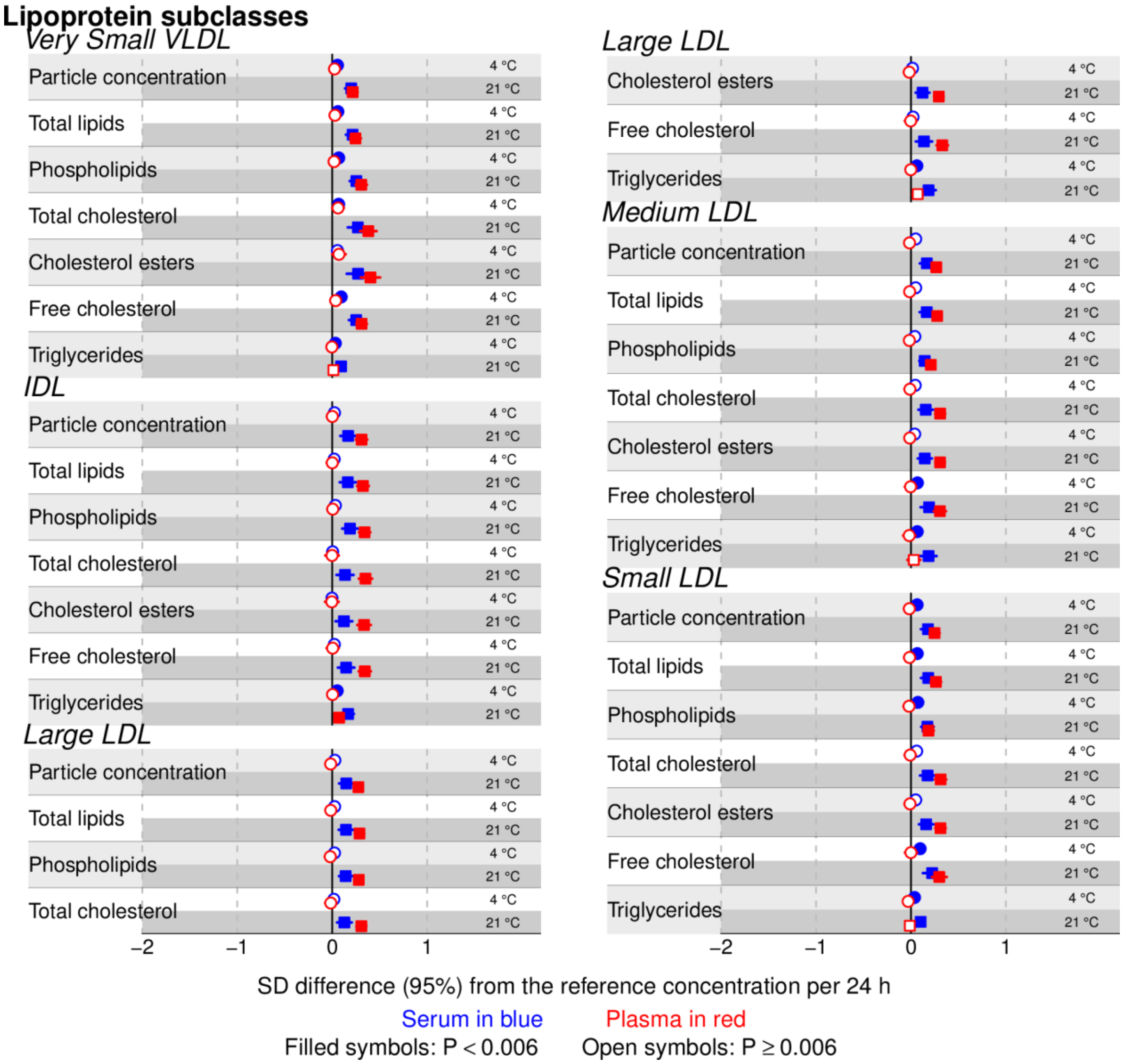

metabolites MDPI

Figure S5 (continued). Pre-storage handling effects (differences in mean levels): standardized mean differences in metabolic traits concentration, in serum and EDTA-plasma, per 24 h increment in incubation duration at 4 °C and 21 °C. Mean differences in absolute units are listed in Tables S6 and S7.

***Abbreviations**: **HDL**=high-density lipoprotein; **LDL**=low-density lipoprotein; **VLDL**=very-low-density lipoprotein.*

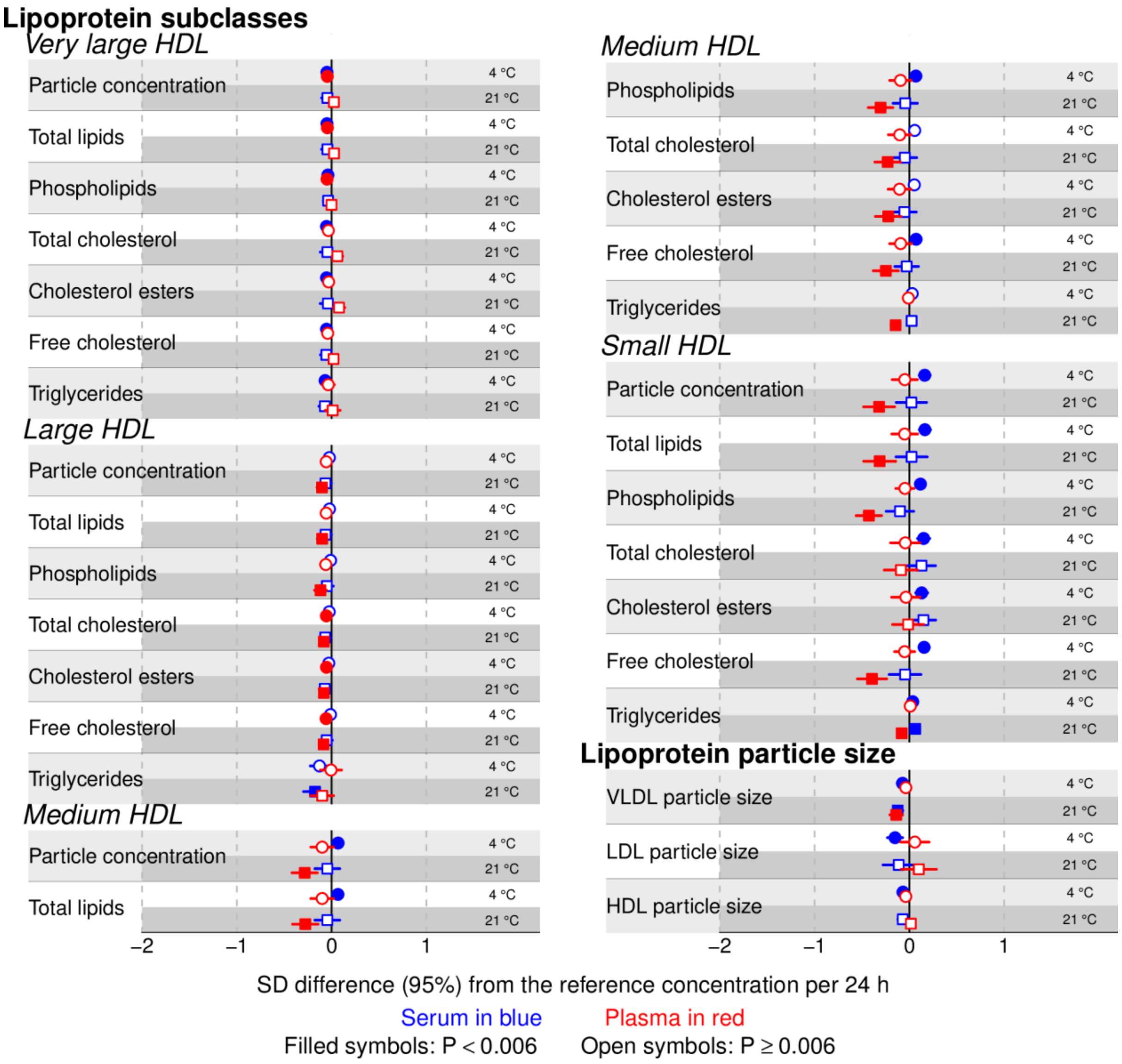

Figure S5 (continued). Pre-storage handling effects (differences in mean levels): standardized mean differences in metabolic traits concentration, serum and EDTA-plasma, per 24 h increment in incubation duration at 4 °C and 21 °C. Mean differences in absolute units are listed in Tables S6 and S7.

Abbreviations: ***C***=cholesterol; **HDL**=high-density lipoprotein; **LDL**=low-density lipoprotein; ***MUFA***=*monounsaturated fatty acids;* ***PUFA***=*polyunsaturated fatty acids*; **VLDL**=very-low-density lipoprotein.

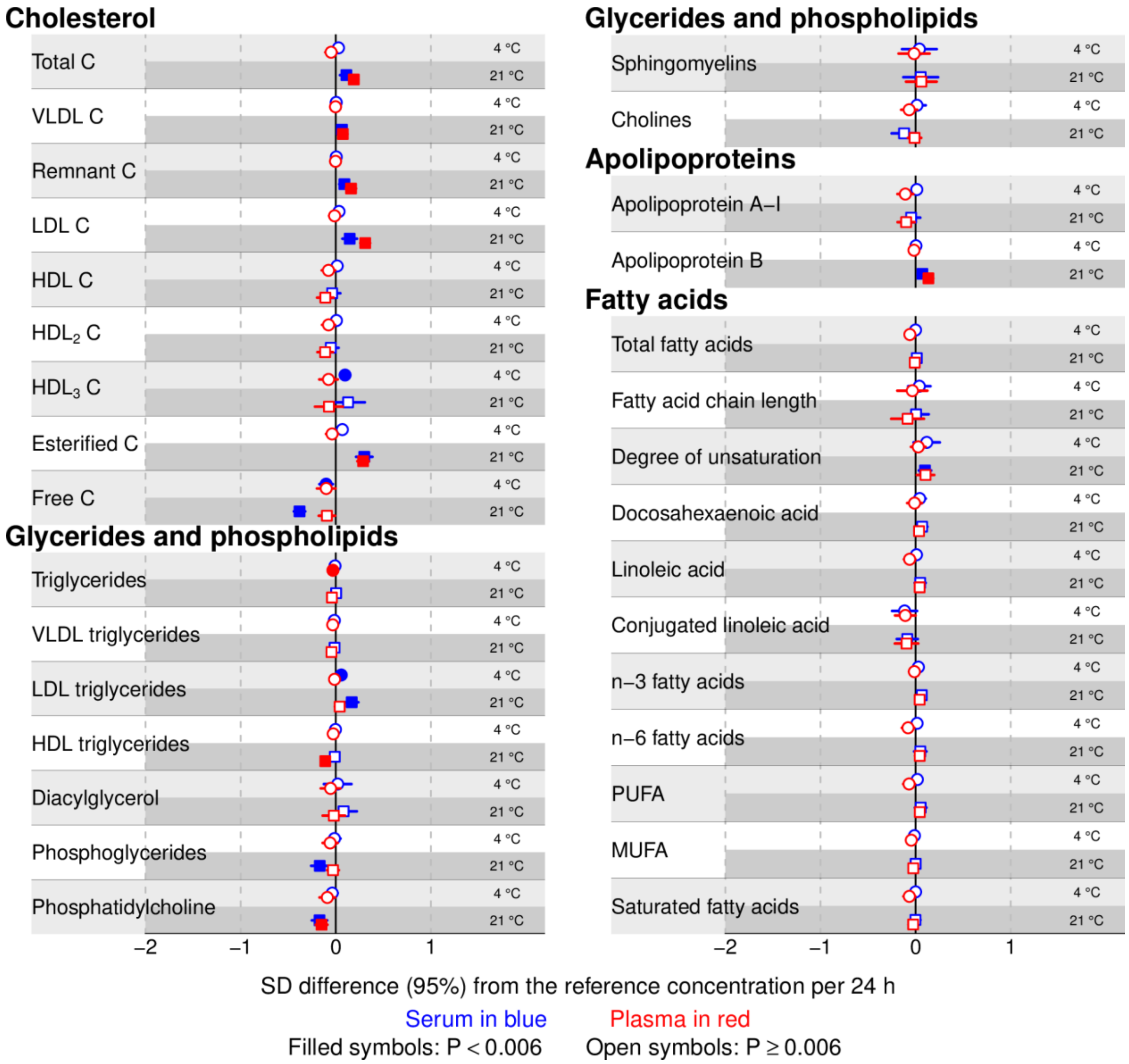

Figure S5 (continued). Pre-storage handling effects (differences in mean levels): standardized mean differences in metabolic traits concentration, in serum and EDTA-plasma, per 24 h increment in incubation duration at 4 °C and 21 °C. Mean differences in absolute units are listed in Tables S6 and S7.

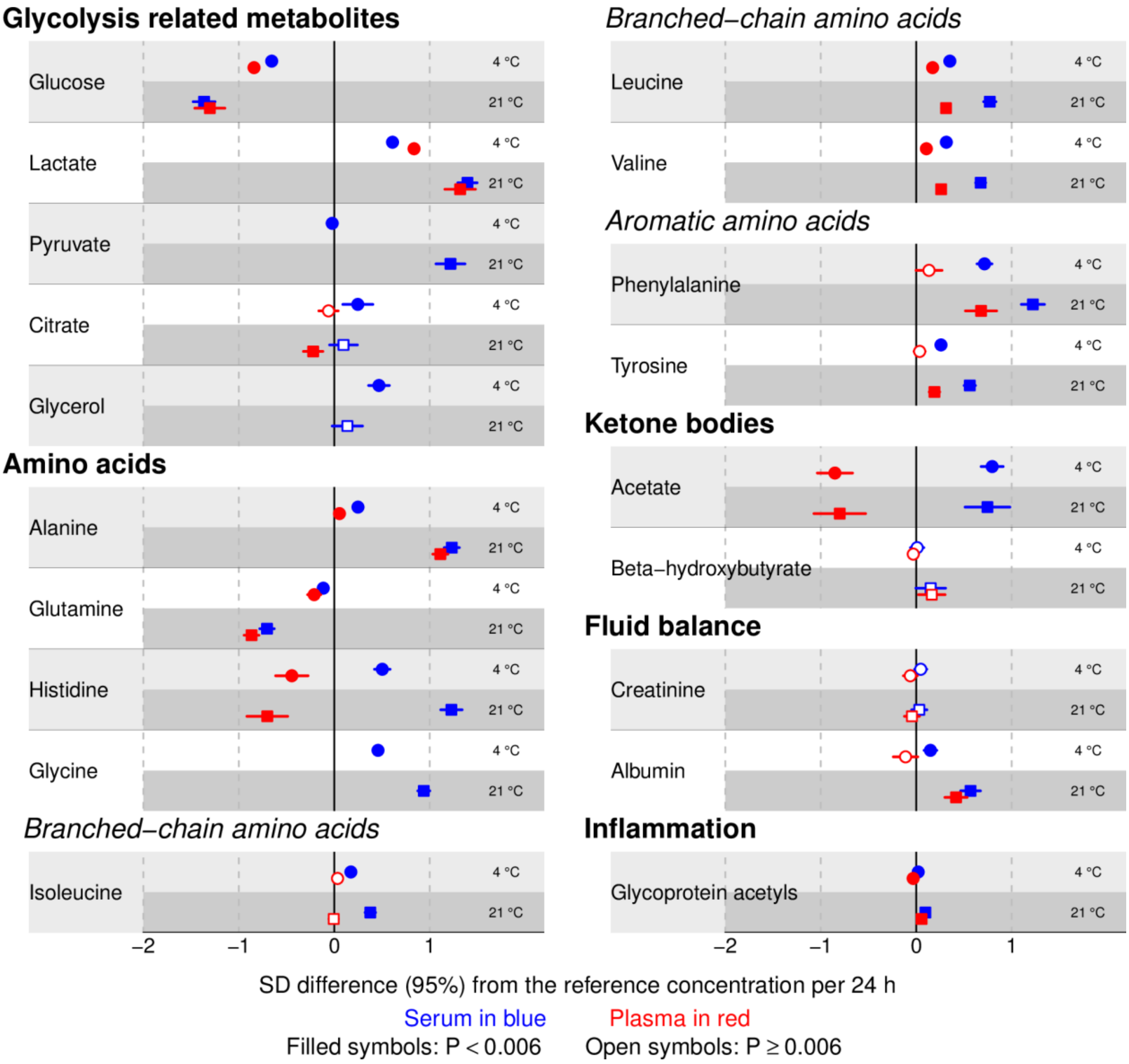

metabolites MDPI

Figure S6. Post-storage handling effects (differences in mean levels): standardized mean differences in metabolic traits concentration comparing delays in buffer addition (i.e. 24 h delay in buffer addition) and NMR profiling (i.e. 24 h delay in NMR analysis) to the reference (no delays), for serum and EDTA-plasma. Mean differences in absolute units are listed in Tables S8 and S9.

***Abbreviations**: **VLDL**=very-low-density lipoprotein.*

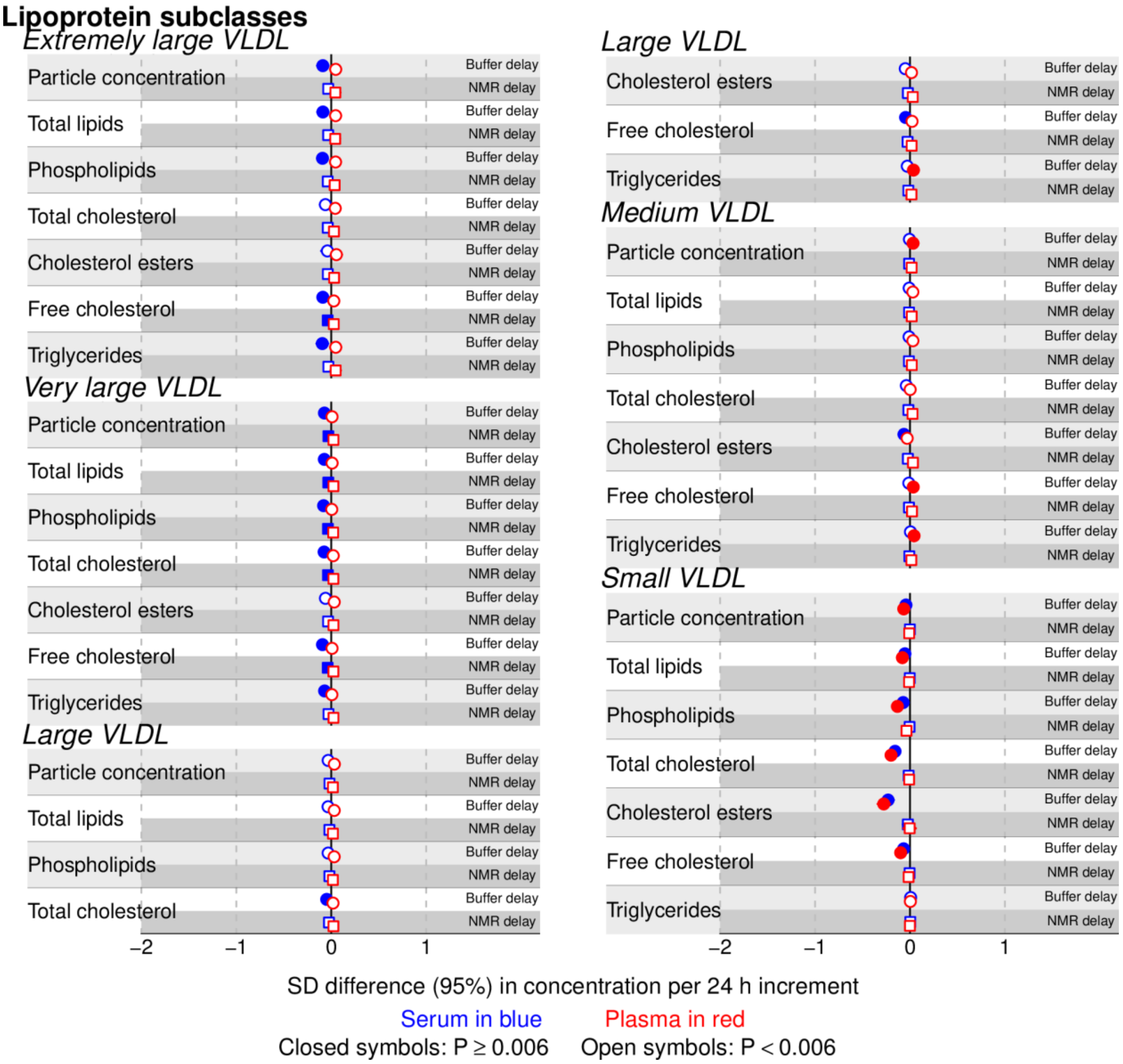

metabolites MDPI

Figure S6 (continued). Post -storage handling effects (differences in mean levels): standardized mean differences in metabolic traits concentration comparing delays in buffer addition (i.e. 24 h delay in buffer addition) and NMR profiling (i.e. 24 h delay in NMR analysis) to the reference (no delays), for serum and EDTA-plasma. Mean differences in absolute units are listed in Tables S8 and S9.

***Abbreviations**: **IDL**=intermediate-density lipoprotein; **LDL**=low-density lipoprotein; **VLDL**=very-low-density lipoprotein.*

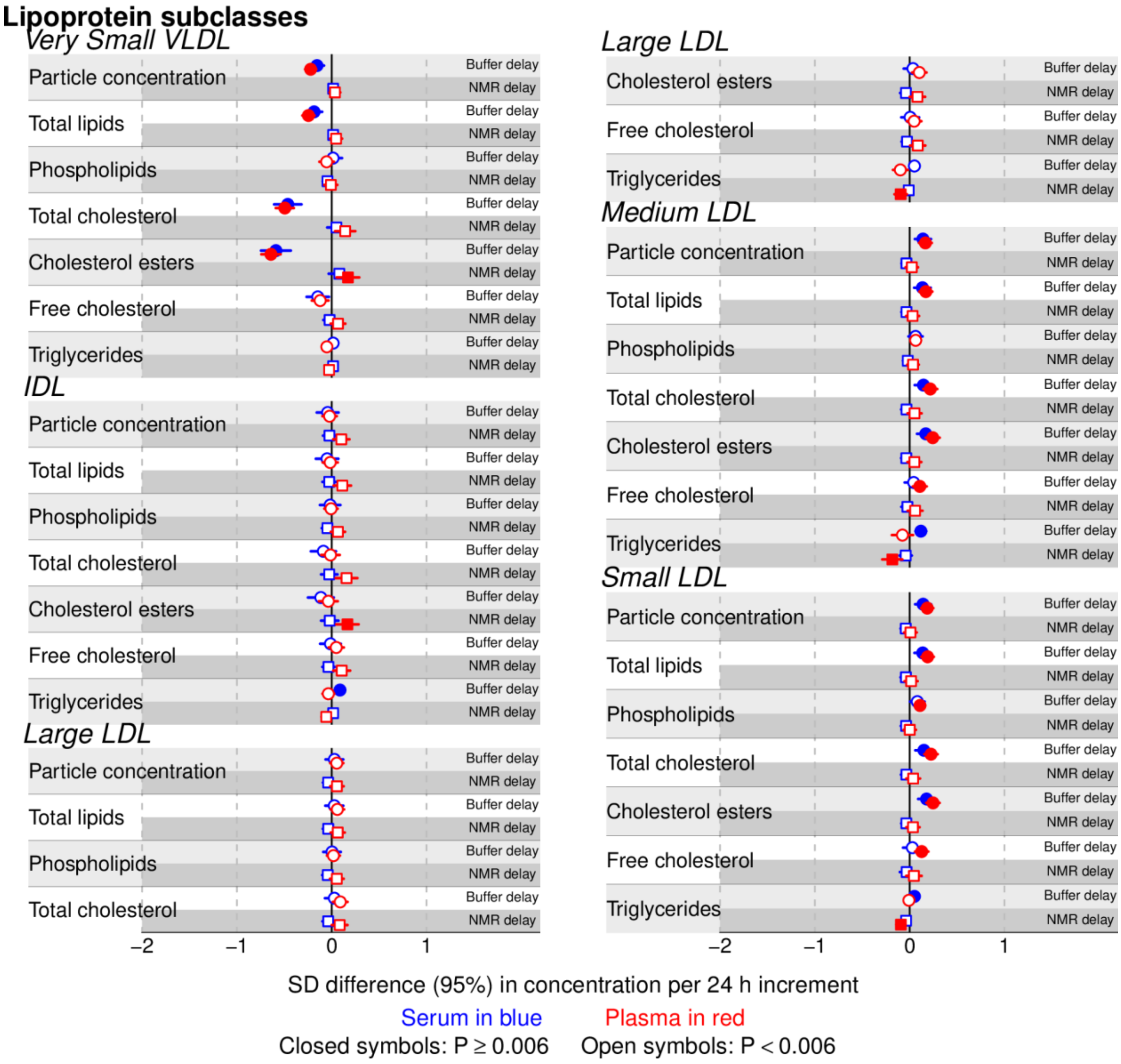

metabolites

MDPI

Figure S6 (continued). Post -storage handling effects (differences in mean levels): standardized mean differences in metabolic traits concentration comparing delays in buffer addition (i.e. 24 h delay in buffer addition) and NMR profiling (i.e. 24 h delay in NMR analysis) to the reference (no delays), for serum and EDTA-plasma. Mean differences in absolute units are listed in Tables S8 and S9.

***Abbreviations**: **HDL**=high-density lipoprotein; **LDL**=low-density lipoprotein; **VLDL**=very-low-density lipoprotein.*

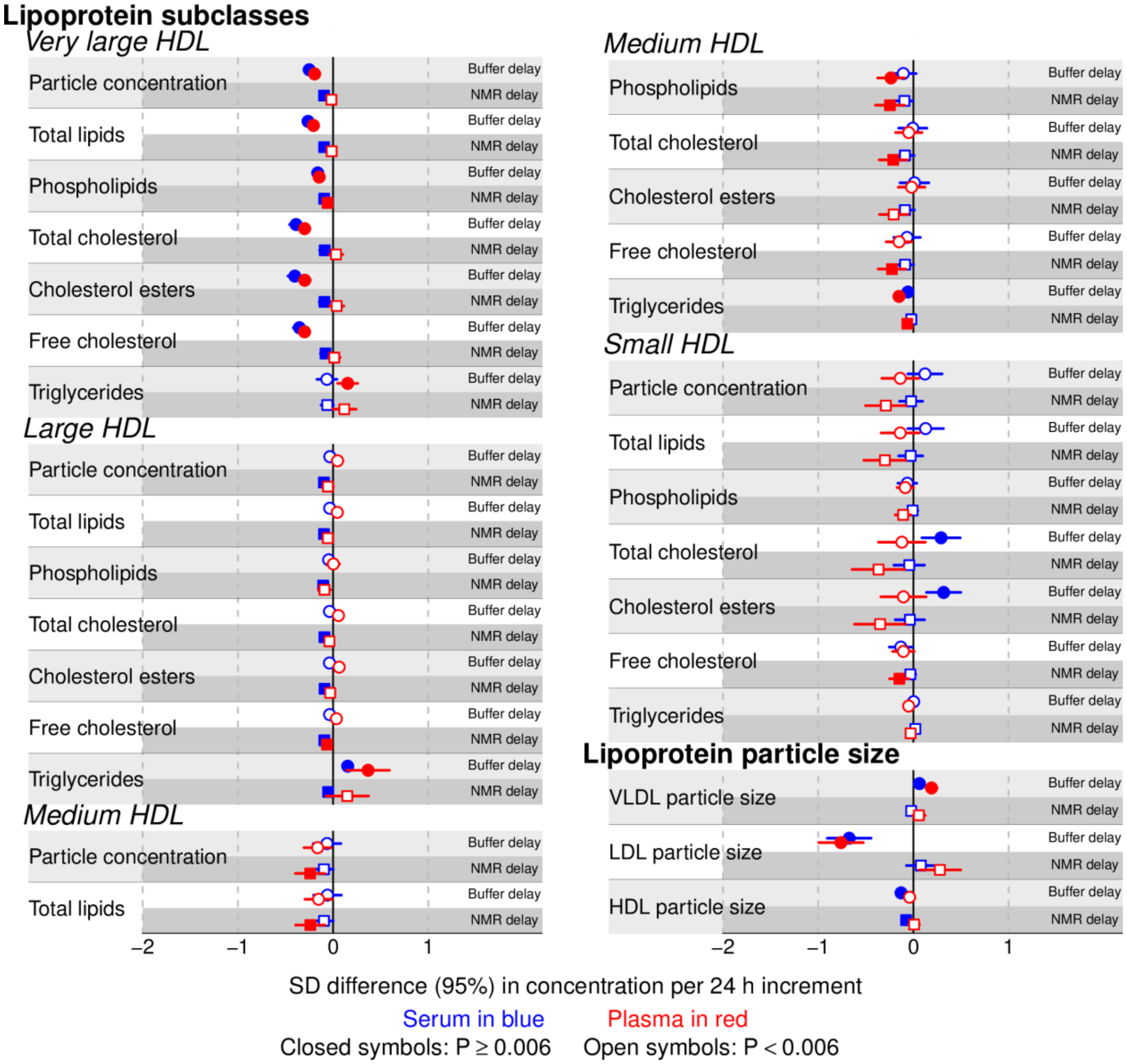

Figure S6 (continued). Post -storage handling effects (differences in mean levels): standardized mean differences in metabolic traits concentration comparing delays in buffer addition (i.e. 24 h delay in buffer addition) and NMR profiling (i.e. 24 h delay in NMR analysis) to the reference (no delays), for serum and EDTA-plasma. Mean differences in absolute units are listed in Tables S8 and S9.

***Abbreviations**: **C**=cholesterol; **HDL**=high-density lipoprotein; **LDL**=low-density lipoprotein; **MUFA**=monounsaturated fatty acids; **PUFA**=polyunsaturated fatty acids; **VLDL**=very-low-density lipoprotein.*

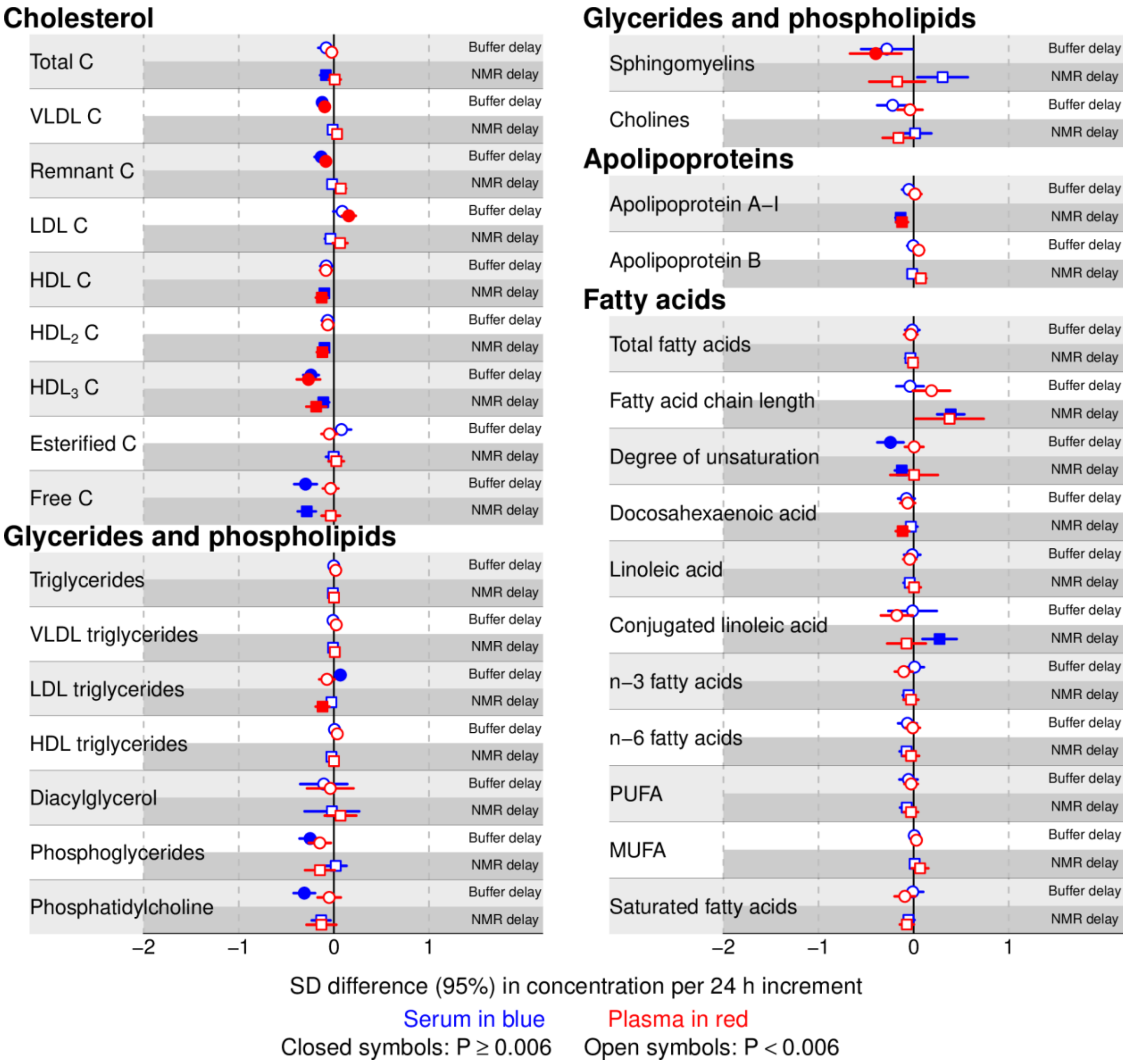

Figure S6 (continued). Post -storage handling effects (differences in mean levels): standardized mean differences in metabolic traits concentration comparing delays in buffer addition (i.e. 24 h delay in buffer addition) and NMR profiling (i.e. 24 h delay in NMR analysis) to the reference (no delays), for serum and EDTA-plasma. Mean differences in absolute units are listed in Tables S8 and S9.

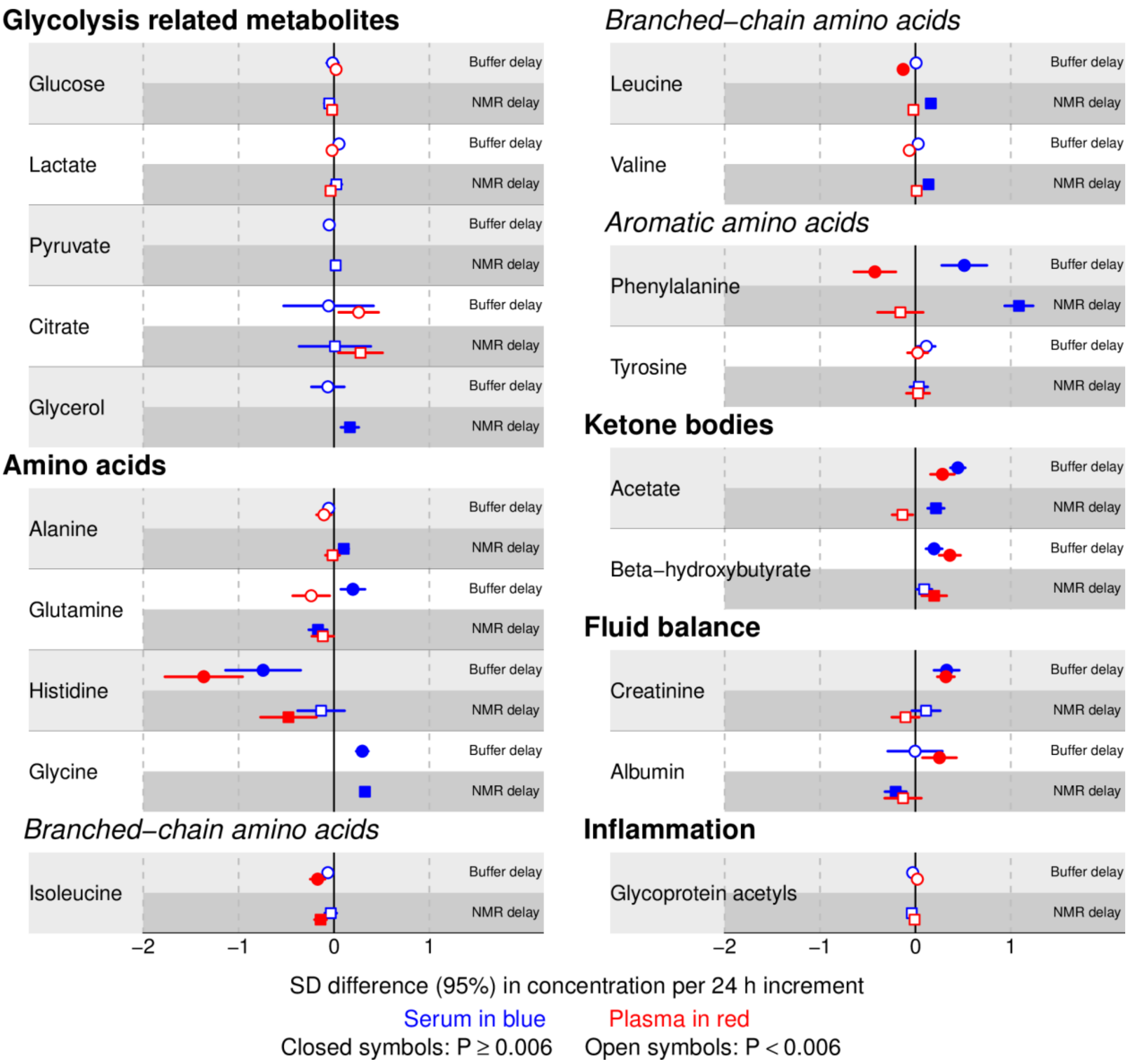

Figure S7-Principal Component Analysis (PCA) on serum non-lipid-related metabolic traits subjected to five different pre-storage conditions. PCA was done on subsetted metabolic profiles which only includes the 19 (out of 151) non-lipid-related metabolic traits. Principal component (PC) 2 versus PC1. PCA is an exploratory method as it does not focus on finding differences between pre-storage conditions but to explain as much of the variation as possible of the metabolomic profiles with a few new variables (i.e. PCs).[2] ***[A] Correlation circle****: each metabolic trait is shown as a vector. For the displayed PCs [2], angles between metabolite vectors indicate their degree of correlation. Positively correlated metabolic traits are grouped together (≈ 0° angle); negatively correlated ones are positioned on opposite sides of the plot origin (≈ 180° degrees); a 90° angle between two metabolites indicate that they are uncorrelated. Dashed grey circles represent 100%, 50% and 25% explained variance of a metabolic trait by a PC. Metabolic traits, whose variance is explained by less than 50% by a PC are usually considered nonrelevant for that PC.* ***[B] Scores plot with ellipses enclosing samples from the same participant:*** *each participant is shown as a number and is represented by 5 data-points corresponding to each pre-storage condition; each pre-storage condition is colour coded. Clustering of samples indicates their compositional similarity for the metabolic traits described by the PCs displayed [2]. Samples from the same participant are enclosed by dotted lines.* ***[C] Scores plot with ellipses enclosing samples subjected to the same pre-storage conditions:*** *same as [B] but with samples subjected by the same pre-storage handling conditions enclosed by dotted lines. Ellipses are colour coded by pre-storage conditions.*

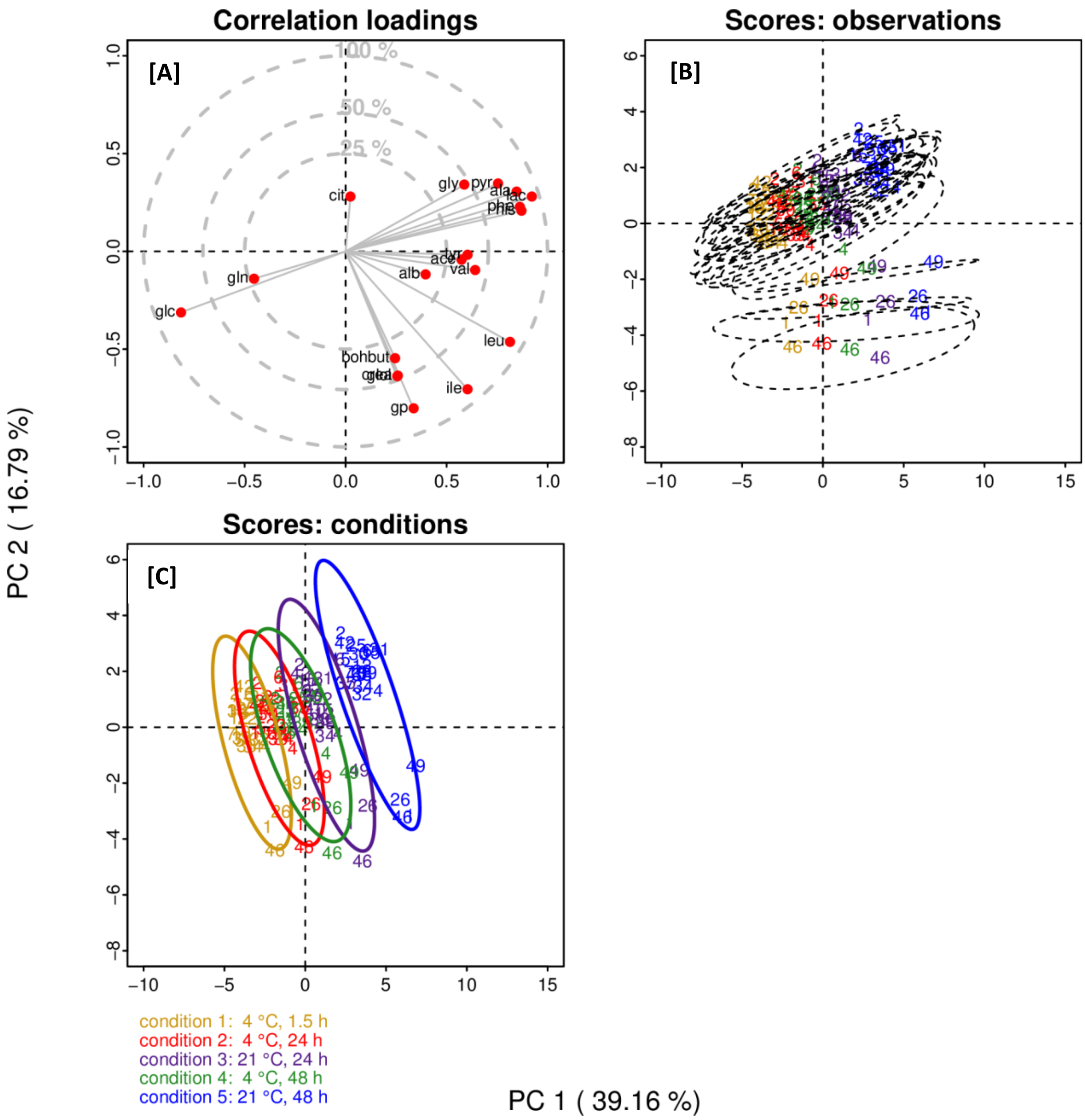

***PCA on non-lipid-related metabolic traits**: PC1 and PC2, which explains 55.95 % of the total variance of the non-lipid-related metabolic traits (19 of the 151 metabolic traits), contains information of both, pre-storage conditions (PC1) and inter-individual differences (PC2). On PC1, from left to right, samples cluster according to increase in incubation temperature first and duration in second (sample clustering order: reference condition (4 ^{0}C-1.5 h); 4 ^{0}C-24 h; 4 ^{0}C-48 h; 21 ^{0}C-24 h; 21 ^{0}C-48 h) illustrating the overall sample degradation signature. Moreover, from left (reference condition) to right (21 ^{0}C-48 h), samples have progressive decreasing concentrations of glucose and higher concentrations of lactate, pyruvate, alanine, histidine, and phenylalanine. Glucose is inversely correlated (≈180^0 angle) with lactate, pyruvate, histidine, phenylalanine and alanine. Moreover, the latter 4 metabolites are positively correlated amongst themselves (≈ 0^0 angles). PC1 explains more than 50% of each of these 5 metabolites variance (i.e. correlation between PC1 scores and each metabolite is more than ± 0.71). No further clustering by pre-storage handling conditions at higher PC dimensions.*

In plasma, the overall degradation fingerprint includes decrease in glucose and glutamine, alongside increased lactate and alanine (data not shown).

*PCA on the **whole** serum metabolic profiles (i.e. the 151 lipid and non-lipid-related metabolic traits), did not differentiate samples by pre-storage conditions until PCs 4 and 5, which had strong contributions of glycolysis related metabolites and amino-acids (data not shown). In the scores plot of the first two PCs, samples clustered only according to participant, indicating that metabolic profile differences between participants were larger than metabolic concentration differences due to pre-storage conditions. Since Nightingale Health© NMR platform is mostly a lipidomic platform (only 13% of the metabolic traits are non-lipid-related) these traits are over represented in the metabolic profiles, and since these lipid-related traits are mostly robust to pre-storage conditions, the biggest variation in the whole metabolic profiles (and explained by the first few PCs) is associated with inter-individual differences and not differences induced by the pre-storage conditions (which only affects up to 9% of the metabolic traits).*

***Abbreviations**: **ala**=alanine; **alb**=albumin; **ace**=acetate; **acace**=acetoacetate; **bohbut**=beta-hydroxybutyrate; **cit**=citrate; **crea** =creatinine; **glc**=glucose; **lac**=lactate; **pyr**=pyruvate; **glol**=glycerol; **gln**=glutamine; **his**=histidine; **gly**=glycine; **ile**= isoleucine; **leu**= leucine; **val**= valine; **phe**=phenylalanine; **tyr**=tyrosine; **gp**=glycoprotein acetyls.*

Supplementary tables

Table S1. Characteristics of metabolic traits: metabolic traits concentration (or value) in serum and EDTA-plasma samples, subjected to the reference pre and post-storage conditions, from individuals who contributed to at least one pair of exposure-outcome analysis (n=37). Pyruvate, glycerol and glycine are not quantified in Ethylenediaminetetraacetic acid (EDTA) - plasma samples due to the interfering resonances of EDTA on their signals.

***Abbreviations**: **C**=cholesterol; **IDL**=intermediate-density lipoprotein; **iqr25**= 25th percentile; **iqr75**= 75th percentile; **LDL**=low-density lipoprotein; **HDL**=high-density lipoprotein; **MUFA**=monounsaturated fatty acids; **PUFA**=polyunsaturated fatty acids; **sd**= standard deviation; **VLDL**=very-low-density lipoprotein.*

Metabolic traits	sample	mean	sd	median	iqr25	iqr75	min	max
**Lipoprotein subclasses**								
Extremely large VLDL								
Particle concentration (mol/l)	SERUM	1.2e-10	1.9e-10	6.7e-11	4.4e-11	1.3e-10	0.0e+00	9.7e-10
Particle concentration (mol/l)	PLASMA	1.1e-10	2.1e-10	5.3e-11	0.0e+00	9.0e-11	0.0e+00	9.2e-10
Total lipids (mmol/l)	SERUM	2.5e-02	4.0e-02	1.4e-02	9.2e-03	2.8e-02	0.0e+00	2.1e-01
Total lipids (mmol/l)	PLASMA	2.4e-02	4.6e-02	1.1e-02	0.0e+00	1.9e-02	0.0e+00	2.0e-01
Phospholipids (mmol/l)	SERUM	3.1e-03	5.0e-03	1.6e-03	8.8e-04	3.4e-03	0.0e+00	2.6e-02
Phospholipids (mmol/l)	PLASMA	3.0e-03	5.7e-03	1.3e-03	0.0e+00	2.3e-03	0.0e+00	2.5e-02
Total cholesterol (mmol/l)	SERUM	4.3e-03	7.4e-03	2.3e-03	8.8e-04	4.6e-03	0.0e+00	3.9e-02
Total cholesterol (mmol/l)	PLASMA	4.2e-03	8.6e-03	1.3e-03	0.0e+00	3.3e-03	0.0e+00	3.7e-02
Cholesterol esters (mmol/l)	SERUM	2.4e-03	4.2e-03	1.1e-03	2.2e-04	2.3e-03	0.0e+00	2.2e-02
Cholesterol esters (mmol/l)	PLASMA	2.3e-03	4.8e-03	3.6e-04	0.0e+00	2.0e-03	0.0e+00	2.1e-02
Free cholesterol (mmol/l)	SERUM	1.9e-03	3.3e-03	9.6e-04	3.3e-04	2.1e-03	0.0e+00	1.7e-02
Free cholesterol (mmol/l)	PLASMA	1.9e-03	3.8e-03	6.6e-04	0.0e+00	1.3e-03	0.0e+00	1.6e-02
Triglycerides (mmol/l)	SERUM	1.8e-02	2.8e-02	1.0e-02	7.2e-03	2.0e-02	0.0e+00	1.4e-01
Triglycerides (mmol/l)	PLASMA	1.7e-02	3.2e-02	8.4e-03	0.0e+00	1.4e-02	0.0e+00	1.4e-01
Very large VLDL								
Particle concentration (mol/l)	SERUM	6.4e-10	1.1e-09	3.1e-10	7.0e-11	7.7e-10	0.0e+00	5.6e-09
Particle concentration (mol/l)	PLASMA	6.8e-10	1.3e-09	2.8e-10	0.0e+00	6.4e-10	0.0e+00	5.5e-09
Total lipids (mmol/l)	SERUM	6.2e-02	1.1e-01	3.0e-02	6.6e-03	7.5e-02	0.0e+00	5.5e-01
Total lipids (mmol/l)	PLASMA	6.6e-02	1.3e-01	2.6e-02	0.0e+00	6.1e-02	0.0e+00	5.3e-01

Metabolic traits	sample	mean	sd	median	iqr25	iqr75	min	max
Phospholipids (mmol/l)	SERUM	1.0e-02	1.8e-02	4.8e-03	1.4e-03	1.2e-02	0.0e+00	9.2e-02
Phospholipids (mmol/l)	PLASMA	1.1e-02	2.1e-02	3.2e-03	0.0e+00	9.0e-03	0.0e+00	8.9e-02
Total cholesterol (mmol/l)	SERUM	1.2e-02	2.2e-02	5.4e-03	5.2e-04	1.5e-02	0.0e+00	1.1e-01
Total cholesterol (mmol/l)	PLASMA	1.2e-02	2.5e-02	3.5e-03	0.0e+00	8.6e-03	0.0e+00	1.1e-01
Cholesterol esters (mmol/l)	SERUM	6.6e-03	1.2e-02	3.1e-03	3.0e-06	7.6e-03	0.0e+00	6.2e-02
Cholesterol esters (mmol/l)	PLASMA	6.7e-03	1.4e-02	1.9e-03	0.0e+00	5.0e-03	0.0e+00	6.0e-02
Free cholesterol (mmol/l)	SERUM	5.4e-03	1.0e-02	2.1e-03	5.2e-04	6.3e-03	0.0e+00	5.2e-02
Free cholesterol (mmol/l)	PLASMA	5.6e-03	1.2e-02	1.7e-03	0.0e+00	4.2e-03	0.0e+00	5.0e-02
Triglycerides (mmol/l)	SERUM	4.0e-02	6.9e-02	2.0e-02	4.3e-03	4.8e-02	0.0e+00	3.5e-01
Triglycerides (mmol/l)	PLASMA	4.3e-02	7.9e-02	2.0e-02	0.0e+00	4.2e-02	0.0e+00	3.4e-01
Large VLDL								
Particle concentration (mol/l)	SERUM	4.4e-09	6.3e-09	2.4e-09	1.3e-09	5.1e-09	0.0e+00	3.2e-08
Particle concentration (mol/l)	PLASMA	4.8e-09	7.3e-09	2.2e-09	1.6e-09	4.7e-09	0.0e+00	3.2e-08
Total lipids (mmol/l)	SERUM	2.5e-01	3.7e-01	1.4e-01	7.0e-02	3.0e-01	0.0e+00	1.9e+00
Total lipids (mmol/l)	PLASMA	2.8e-01	4.2e-01	1.2e-01	8.8e-02	2.7e-01	0.0e+00	1.8e+00
Phospholipids (mmol/l)	SERUM	4.6e-02	6.6e-02	2.6e-02	1.3e-02	5.5e-02	0.0e+00	3.4e-01
Phospholipids (mmol/l)	PLASMA	5.1e-02	7.6e-02	2.3e-02	1.6e-02	5.1e-02	0.0e+00	3.3e-01
Total cholesterol (mmol/l)	SERUM	5.5e-02	8.5e-02	2.7e-02	1.1e-02	6.6e-02	0.0e+00	4.4e-01
Total cholesterol (mmol/l)	PLASMA	5.8e-02	9.8e-02	2.4e-02	1.1e-02	5.3e-02	0.0e+00	4.2e-01
Cholesterol esters (mmol/l)	SERUM	2.9e-02	4.2e-02	1.6e-02	7.8e-03	3.2e-02	0.0e+00	2.2e-01
Cholesterol esters (mmol/l)	PLASMA	3.0e-02	4.9e-02	1.3e-02	7.2e-03	2.7e-02	0.0e+00	2.1e-01
Free cholesterol (mmol/l)	SERUM	2.6e-02	4.3e-02	1.3e-02	3.1e-03	3.1e-02	0.0e+00	2.2e-01
Free cholesterol (mmol/l)	PLASMA	2.8e-02	4.9e-02	1.1e-02	4.7e-03	2.7e-02	0.0e+00	2.1e-01
Triglycerides (mmol/l)	SERUM	1.5e-01	2.2e-01	8.6e-02	4.7e-02	1.7e-01	0.0e+00	1.1e+00
Triglycerides (mmol/l)	PLASMA	1.7e-01	2.5e-01	8.2e-02	5.6e-02	1.7e-01	0.0e+00	1.1e+00
Medium VLDL								
Particle concentration (mol/l)	SERUM	1.5e-08	1.6e-08	9.5e-09	7.8e-09	1.6e-08	2.5e-09	8.3e-08
Particle concentration (mol/l)	PLASMA	1.6e-08	1.8e-08	9.5e-09	8.5e-09	1.5e-08	3.3e-09	8.3e-08
Total lipids (mmol/l)	SERUM	5.1e-01	5.2e-01	3.2e-01	2.6e-01	5.2e-01	8.6e-02	2.8e+00

Metabolic traits	sample	mean	sd	median	iqr25	iqr75	min	max
Total lipids (mmol/l)	PLASMA	5.5e-01	6.0e-01	3.1e-01	2.8e-01	5.1e-01	1.1e-01	2.7e+00
Phospholipids (mmol/l)	SERUM	1.0e-01	1.0e-01	6.7e-02	5.5e-02	1.1e-01	2.1e-02	5.3e-01
Phospholipids (mmol/l)	PLASMA	1.1e-01	1.1e-01	6.3e-02	5.9e-02	1.0e-01	2.5e-02	5.3e-01
Total cholesterol (mmol/l)	SERUM	1.4e-01	1.3e-01	9.3e-02	7.1e-02	1.4e-01	2.6e-02	7.1e-01
Total cholesterol (mmol/l)	PLASMA	1.4e-01	1.5e-01	8.1e-02	6.4e-02	1.4e-01	3.1e-02	7.0e-01
Cholesterol esters (mmol/l)	SERUM	7.8e-02	6.6e-02	5.7e-02	4.8e-02	8.5e-02	1.8e-02	3.7e-01
Cholesterol esters (mmol/l)	PLASMA	7.6e-02	7.7e-02	4.7e-02	3.7e-02	8.6e-02	2.1e-02	3.6e-01
Free cholesterol (mmol/l)	SERUM	5.8e-02	6.5e-02	3.8e-02	2.5e-02	6.7e-02	6.6e-03	3.4e-01
Free cholesterol (mmol/l)	PLASMA	6.2e-02	7.5e-02	3.4e-02	2.9e-02	6.0e-02	1.0e-02	3.4e-01
Triglycerides (mmol/l)	SERUM	2.7e-01	2.9e-01	1.6e-01	1.3e-01	2.9e-01	3.6e-02	1.5e+00
Triglycerides (mmol/l)	PLASMA	3.0e-01	3.4e-01	1.8e-01	1.4e-01	2.9e-01	5.3e-02	1.5e+00
Small VLDL								
Particle concentration (mol/l)	SERUM	2.5e-08	1.5e-08	1.9e-08	1.7e-08	2.7e-08	9.5e-09	8.7e-08
Particle concentration (mol/l)	PLASMA	2.6e-08	1.7e-08	2.0e-08	1.8e-08	2.7e-08	1.1e-08	8.7e-08
Total lipids (mmol/l)	SERUM	4.8e-01	2.9e-01	3.8e-01	3.4e-01	5.3e-01	1.8e-01	1.7e+00
Total lipids (mmol/l)	PLASMA	5.1e-01	3.3e-01	4.0e-01	3.3e-01	5.3e-01	2.1e-01	1.7e+00
Phospholipids (mmol/l)	SERUM	1.2e-01	5.7e-02	9.7e-02	8.4e-02	1.3e-01	5.5e-02	3.5e-01
Phospholipids (mmol/l)	PLASMA	1.2e-01	6.5e-02	1.0e-01	8.7e-02	1.3e-01	6.2e-02	3.5e-01
Total cholesterol (mmol/l)	SERUM	1.6e-01	7.9e-02	1.4e-01	1.2e-01	1.8e-01	4.6e-02	4.5e-01
Total cholesterol (mmol/l)	PLASMA	1.6e-01	9.1e-02	1.3e-01	1.0e-01	1.7e-01	6.0e-02	4.5e-01
Cholesterol esters (mmol/l)	SERUM	9.6e-02	4.4e-02	8.3e-02	7.1e-02	1.1e-01	2.1e-02	2.4e-01
Cholesterol esters (mmol/l)	PLASMA	8.7e-02	5.0e-02	7.3e-02	5.5e-02	9.6e-02	2.8e-02	2.3e-01
Free cholesterol (mmol/l)	SERUM	6.7e-02	3.7e-02	5.4e-02	4.6e-02	7.5e-02	2.6e-02	2.1e-01
Free cholesterol (mmol/l)	PLASMA	7.1e-02	4.2e-02	5.7e-02	4.8e-02	7.5e-02	3.2e-02	2.1e-01
Triglycerides (mmol/l)	SERUM	2.0e-01	1.5e-01	1.5e-01	1.3e-01	2.1e-01	7.1e-02	8.5e-01
Triglycerides (mmol/l)	PLASMA	2.3e-01	1.7e-01	1.7e-01	1.4e-01	2.3e-01	8.9e-02	8.6e-01
Very Small VLDL								
Particle concentration (mol/l)	SERUM	3.1e-08	8.4e-09	3.0e-08	2.7e-08	3.4e-08	1.6e-08	5.5e-08
Particle concentration (mol/l)	PLASMA	3.0e-08	9.4e-09	2.7e-08	2.5e-08	3.2e-08	1.7e-08	5.5e-08

Metabolic traits	sample	mean	sd	median	iqr25	iqr75	min	max
Total lipids (mmol/l)	SERUM	4.0e-01	1.0e-01	3.8e-01	3.4e-01	4.3e-01	2.0e-01	6.4e-01
Total lipids (mmol/l)	PLASMA	3.7e-01	1.1e-01	3.4e-01	3.1e-01	4.1e-01	2.2e-01	6.4e-01
Phospholipids (mmol/l)	SERUM	1.3e-01	2.9e-02	1.3e-01	1.1e-01	1.5e-01	7.1e-02	2.1e-01
Phospholipids (mmol/l)	PLASMA	1.2e-01	3.0e-02	1.2e-01	1.0e-01	1.4e-01	6.9e-02	2.0e-01
Total cholesterol (mmol/l)	SERUM	1.7e-01	4.4e-02	1.7e-01	1.5e-01	2.0e-01	8.3e-02	2.9e-01
Total cholesterol (mmol/l)	PLASMA	1.5e-01	4.5e-02	1.4e-01	1.2e-01	1.8e-01	9.4e-02	2.6e-01
Cholesterol esters (mmol/l)	SERUM	1.1e-01	3.1e-02	1.1e-01	9.0e-02	1.3e-01	4.6e-02	1.9e-01
Cholesterol esters (mmol/l)	PLASMA	9.5e-02	3.1e-02	8.6e-02	7.6e-02	1.1e-01	5.6e-02	1.7e-01
Free cholesterol (mmol/l)	SERUM	6.4e-02	1.4e-02	6.2e-02	5.6e-02	7.2e-02	3.3e-02	1.0e-01
Free cholesterol (mmol/l)	PLASMA	5.7e-02	1.4e-02	5.5e-02	4.6e-02	6.8e-02	3.6e-02	9.4e-02
Triglycerides (mmol/l)	SERUM	9.1e-02	4.2e-02	7.9e-02	6.8e-02	9.3e-02	4.6e-02	2.7e-01
Triglycerides (mmol/l)	PLASMA	9.8e-02	4.8e-02	8.1e-02	7.3e-02	1.0e-01	5.1e-02	2.7e-01
IDL								
Particle concentration (mol/l)	SERUM	9.4e-08	1.9e-08	9.3e-08	8.0e-08	1.1e-07	5.2e-08	1.4e-07
Particle concentration (mol/l)	PLASMA	8.6e-08	2.0e-08	8.7e-08	7.1e-08	1.0e-07	5.5e-08	1.3e-07
Total lipids (mmol/l)	SERUM	9.5e-01	2.0e-01	9.5e-01	8.1e-01	1.1e+00	5.3e-01	1.4e+00
Total lipids (mmol/l)	PLASMA	8.6e-01	2.0e-01	8.7e-01	7.2e-01	1.0e+00	5.6e-01	1.3e+00
Phospholipids (mmol/l)	SERUM	2.6e-01	5.0e-02	2.6e-01	2.3e-01	3.0e-01	1.7e-01	3.8e-01
Phospholipids (mmol/l)	PLASMA	2.4e-01	5.0e-02	2.4e-01	2.1e-01	2.7e-01	1.6e-01	3.5e-01
Total cholesterol (mmol/l)	SERUM	5.9e-01	1.3e-01	5.9e-01	5.0e-01	6.6e-01	3.1e-01	9.0e-01
Total cholesterol (mmol/l)	PLASMA	5.2e-01	1.4e-01	5.1e-01	4.1e-01	6.3e-01	3.2e-01	7.8e-01
Cholesterol esters (mmol/l)	SERUM	4.2e-01	9.7e-02	4.2e-01	3.5e-01	4.8e-01	2.1e-01	6.4e-01
Cholesterol esters (mmol/l)	PLASMA	3.6e-01	1.1e-01	3.5e-01	2.8e-01	4.6e-01	2.2e-01	5.4e-01
Free cholesterol (mmol/l)	SERUM	1.7e-01	4.1e-02	1.7e-01	1.5e-01	2.0e-01	1.0e-01	2.7e-01
Free cholesterol (mmol/l)	PLASMA	1.6e-01	4.0e-02	1.5e-01	1.3e-01	1.7e-01	1.0e-01	2.4e-01
Triglycerides (mmol/l)	SERUM	9.7e-02	2.8e-02	9.3e-02	7.8e-02	1.1e-01	5.0e-02	2.0e-01
Triglycerides (mmol/l)	PLASMA	1.0e-01	3.2e-02	9.7e-02	8.2e-02	1.1e-01	5.2e-02	2.0e-01
Large LDL								
Particle concentration (mol/l)	SERUM	1.6e-07	3.4e-08	1.5e-07	1.3e-07	1.8e-07	9.2e-08	2.3e-07

Metabolic traits	sample	mean	sd	median	iqr25	iqr75	min	max
Particle concentration (mol/l)	PLASMA	1.5e-07	3.5e-08	1.4e-07	1.2e-07	1.7e-07	9.0e-08	2.2e-07
Total lipids (mmol/l)	SERUM	1.1e+00	2.4e-01	1.1e+00	9.5e-01	1.3e+00	6.5e-01	1.7e+00
Total lipids (mmol/l)	PLASMA	1.1e+00	2.5e-01	1.0e+00	8.7e-01	1.2e+00	6.4e-01	1.5e+00
Phospholipids (mmol/l)	SERUM	2.9e-01	5.1e-02	2.8e-01	2.5e-01	3.2e-01	1.9e-01	4.1e-01
Phospholipids (mmol/l)	PLASMA	2.7e-01	5.2e-02	2.7e-01	2.3e-01	3.1e-01	1.8e-01	3.7e-01
Total cholesterol (mmol/l)	SERUM	7.5e-01	1.8e-01	7.2e-01	6.4e-01	8.8e-01	4.1e-01	1.2e+00
Total cholesterol (mmol/l)	PLASMA	6.9e-01	1.8e-01	6.6e-01	5.6e-01	8.2e-01	4.0e-01	1.1e+00
Cholesterol esters (mmol/l)	SERUM	5.4e-01	1.4e-01	5.1e-01	4.5e-01	6.2e-01	2.7e-01	8.6e-01
Cholesterol esters (mmol/l)	PLASMA	4.9e-01	1.4e-01	4.6e-01	3.7e-01	5.8e-01	2.6e-01	7.6e-01
Free cholesterol (mmol/l)	SERUM	2.2e-01	4.6e-02	2.1e-01	1.8e-01	2.5e-01	1.4e-01	3.2e-01
Free cholesterol (mmol/l)	PLASMA	2.0e-01	4.5e-02	2.0e-01	1.7e-01	2.1e-01	1.3e-01	2.9e-01
Triglycerides (mmol/l)	SERUM	8.7e-02	2.3e-02	9.0e-02	6.7e-02	1.0e-01	4.2e-02	1.5e-01
Triglycerides (mmol/l)	PLASMA	9.1e-02	2.6e-02	9.2e-02	6.9e-02	1.0e-01	4.2e-02	1.5e-01
Medium LDL								
Particle concentration (mol/l)	SERUM	1.3e-07	3.0e-08	1.2e-07	1.1e-07	1.5e-07	6.9e-08	2.0e-07
Particle concentration (mol/l)	PLASMA	1.2e-07	3.0e-08	1.1e-07	9.8e-08	1.4e-07	6.6e-08	1.8e-07
Total lipids (mmol/l)	SERUM	6.5e-01	1.5e-01	6.2e-01	5.5e-01	7.5e-01	3.5e-01	1.0e+00
Total lipids (mmol/l)	PLASMA	6.1e-01	1.5e-01	5.8e-01	4.9e-01	7.2e-01	3.4e-01	9.0e-01
Phospholipids (mmol/l)	SERUM	1.8e-01	3.3e-02	1.7e-01	1.6e-01	2.0e-01	1.2e-01	2.4e-01
Phospholipids (mmol/l)	PLASMA	1.7e-01	3.6e-02	1.6e-01	1.4e-01	1.9e-01	1.1e-01	2.4e-01
Total cholesterol (mmol/l)	SERUM	4.3e-01	1.1e-01	4.0e-01	3.7e-01	5.0e-01	2.1e-01	7.1e-01
Total cholesterol (mmol/l)	PLASMA	3.9e-01	1.1e-01	3.5e-01	3.2e-01	4.7e-01	1.9e-01	6.3e-01
Cholesterol esters (mmol/l)	SERUM	3.1e-01	9.1e-02	2.7e-01	2.6e-01	3.6e-01	1.2e-01	5.3e-01
Cholesterol esters (mmol/l)	PLASMA	2.7e-01	9.3e-02	2.4e-01	2.2e-01	3.4e-01	1.2e-01	4.7e-01
Free cholesterol (mmol/l)	SERUM	1.3e-01	2.2e-02	1.2e-01	1.1e-01	1.4e-01	8.5e-02	1.8e-01
Free cholesterol (mmol/l)	PLASMA	1.2e-01	2.2e-02	1.1e-01	1.0e-01	1.3e-01	7.7e-02	1.6e-01
Triglycerides (mmol/l)	SERUM	4.5e-02	1.2e-02	4.6e-02	3.4e-02	5.2e-02	2.5e-02	7.6e-02
Triglycerides (mmol/l)	PLASMA	4.8e-02	1.3e-02	4.8e-02	3.8e-02	5.5e-02	2.2e-02	7.5e-02
Small LDL								

Metabolic traits	sample	mean	sd	median	iqr25	iqr75	min	max
Particle concentration (mol/l)	SERUM	1.5e-07	3.4e-08	1.4e-07	1.3e-07	1.8e-07	8.6e-08	2.3e-07
Particle concentration (mol/l)	PLASMA	1.4e-07	3.5e-08	1.3e-07	1.1e-07	1.7e-07	7.6e-08	2.1e-07
Total lipids (mmol/l)	SERUM	4.3e-01	9.6e-02	4.0e-01	3.6e-01	4.8e-01	2.4e-01	6.4e-01
Total lipids (mmol/l)	PLASMA	3.9e-01	9.8e-02	3.7e-01	3.2e-01	4.7e-01	2.1e-01	5.8e-01
Phospholipids (mmol/l)	SERUM	1.3e-01	2.3e-02	1.3e-01	1.2e-01	1.5e-01	9.3e-02	1.8e-01
Phospholipids (mmol/l)	PLASMA	1.3e-01	2.5e-02	1.2e-01	1.1e-01	1.4e-01	8.1e-02	1.7e-01
Total cholesterol (mmol/l)	SERUM	2.6e-01	7.0e-02	2.4e-01	2.2e-01	3.2e-01	1.3e-01	4.4e-01
Total cholesterol (mmol/l)	PLASMA	2.4e-01	7.0e-02	2.2e-01	1.9e-01	2.9e-01	1.1e-01	3.8e-01
Cholesterol esters (mmol/l)	SERUM	1.9e-01	5.6e-02	1.7e-01	1.5e-01	2.2e-01	8.0e-02	3.3e-01
Cholesterol esters (mmol/l)	PLASMA	1.7e-01	5.7e-02	1.5e-01	1.4e-01	2.0e-01	6.9e-02	2.9e-01
Free cholesterol (mmol/l)	SERUM	7.7e-02	1.4e-02	7.4e-02	6.7e-02	8.6e-02	5.2e-02	1.1e-01
Free cholesterol (mmol/l)	PLASMA	6.9e-02	1.4e-02	6.7e-02	5.9e-02	8.0e-02	4.2e-02	9.9e-02
Triglycerides (mmol/l)	SERUM	2.9e-02	1.1e-02	2.7e-02	2.2e-02	3.2e-02	1.5e-02	7.3e-02
Triglycerides (mmol/l)	PLASMA	3.1e-02	1.2e-02	2.9e-02	2.4e-02	3.3e-02	1.3e-02	7.1e-02
Very large HDL								
Particle concentration (mol/l)	SERUM	5.2e-07	2.5e-07	4.6e-07	3.2e-07	7.4e-07	1.4e-07	9.4e-07
Particle concentration (mol/l)	PLASMA	5.2e-07	2.5e-07	4.7e-07	3.2e-07	7.6e-07	1.3e-07	9.4e-07
Total lipids (mmol/l)	SERUM	5.2e-01	2.5e-01	4.6e-01	3.1e-01	7.4e-01	1.4e-01	9.5e-01
Total lipids (mmol/l)	PLASMA	5.2e-01	2.5e-01	4.7e-01	3.2e-01	7.6e-01	1.3e-01	9.4e-01
Phospholipids (mmol/l)	SERUM	2.8e-01	1.4e-01	2.5e-01	1.7e-01	4.1e-01	1.9e-02	5.2e-01
Phospholipids (mmol/l)	PLASMA	2.8e-01	1.5e-01	2.6e-01	2.0e-01	4.1e-01	1.6e-02	5.2e-01
Total cholesterol (mmol/l)	SERUM	2.3e-01	1.1e-01	2.0e-01	1.3e-01	3.1e-01	6.0e-02	4.2e-01
Total cholesterol (mmol/l)	PLASMA	2.3e-01	1.1e-01	2.0e-01	1.4e-01	3.2e-01	7.3e-02	4.0e-01
Cholesterol esters (mmol/l)	SERUM	1.7e-01	7.7e-02	1.5e-01	1.0e-01	2.2e-01	4.8e-02	3.0e-01
Cholesterol esters (mmol/l)	PLASMA	1.6e-01	7.6e-02	1.4e-01	1.0e-01	2.3e-01	6.1e-02	2.9e-01
Free cholesterol (mmol/l)	SERUM	6.1e-02	3.3e-02	5.1e-02	3.2e-02	8.8e-02	1.2e-02	1.2e-01
Free cholesterol (mmol/l)	PLASMA	6.1e-02	3.3e-02	5.4e-02	3.3e-02	9.2e-02	1.2e-02	1.1e-01
Triglycerides (mmol/l)	SERUM	1.8e-02	9.4e-03	1.7e-02	1.3e-02	2.4e-02	2.4e-03	5.2e-02
Triglycerides (mmol/l)	PLASMA	1.7e-02	1.1e-02	1.7e-02	8.5e-03	2.5e-02	7.2e-04	4.9e-02

Metabolic traits	sample	mean	sd	median	iqr25	iqr75	min	max
Large HDL								
Particle concentration (mol/l)	SERUM	1.6e-06	6.5e-07	1.5e-06	1.1e-06	2.3e-06	3.7e-07	2.8e-06
Particle concentration (mol/l)	PLASMA	1.6e-06	7.0e-07	1.5e-06	1.3e-06	2.2e-06	0.0e+00	2.6e-06
Total lipids (mmol/l)	SERUM	1.0e+00	4.2e-01	9.5e-01	7.0e-01	1.4e+00	2.2e-01	1.8e+00
Total lipids (mmol/l)	PLASMA	1.0e+00	4.5e-01	9.2e-01	8.0e-01	1.4e+00	0.0e+00	1.7e+00
Phospholipids (mmol/l)	SERUM	4.9e-01	1.8e-01	4.6e-01	3.5e-01	6.5e-01	1.0e-01	8.3e-01
Phospholipids (mmol/l)	PLASMA	4.8e-01	2.0e-01	4.5e-01	4.2e-01	6.5e-01	0.0e+00	7.7e-01
Total cholesterol (mmol/l)	SERUM	5.0e-01	2.3e-01	4.6e-01	3.3e-01	7.2e-01	7.1e-02	8.7e-01
Total cholesterol (mmol/l)	PLASMA	4.8e-01	2.4e-01	4.5e-01	3.5e-01	7.0e-01	0.0e+00	8.3e-01
Cholesterol esters (mmol/l)	SERUM	3.8e-01	1.7e-01	3.6e-01	2.6e-01	5.6e-01	6.8e-02	6.7e-01
Cholesterol esters (mmol/l)	PLASMA	3.7e-01	1.8e-01	3.5e-01	2.7e-01	5.4e-01	0.0e+00	6.3e-01
Free cholesterol (mmol/l)	SERUM	1.1e-01	5.5e-02	1.0e-01	7.2e-02	1.7e-01	3.4e-03	2.0e-01
Free cholesterol (mmol/l)	PLASMA	1.1e-01	5.7e-02	1.0e-01	8.0e-02	1.6e-01	0.0e+00	1.9e-01
Triglycerides (mmol/l)	SERUM	3.6e-02	1.4e-02	3.2e-02	2.4e-02	5.1e-02	1.6e-02	5.7e-02
Triglycerides (mmol/l)	PLASMA	3.5e-02	1.6e-02	3.3e-02	2.6e-02	5.3e-02	0.0e+00	5.8e-02
Medium HDL								
Particle concentration (mol/l)	SERUM	2.4e-06	3.7e-07	2.4e-06	2.1e-06	2.5e-06	1.8e-06	3.2e-06
Particle concentration (mol/l)	PLASMA	2.4e-06	3.8e-07	2.4e-06	2.1e-06	2.6e-06	1.8e-06	3.3e-06
Total lipids (mmol/l)	SERUM	1.0e+00	1.6e-01	1.0e+00	9.1e-01	1.1e+00	7.4e-01	1.4e+00
Total lipids (mmol/l)	PLASMA	1.0e+00	1.7e-01	1.0e+00	8.8e-01	1.1e+00	7.6e-01	1.4e+00
Phospholipids (mmol/l)	SERUM	4.6e-01	7.4e-02	4.5e-01	4.2e-01	4.9e-01	3.4e-01	6.3e-01
Phospholipids (mmol/l)	PLASMA	4.7e-01	7.6e-02	4.7e-01	4.1e-01	5.1e-01	3.6e-01	6.5e-01
Total cholesterol (mmol/l)	SERUM	5.1e-01	9.2e-02	5.2e-01	4.4e-01	5.5e-01	3.2e-01	7.1e-01
Total cholesterol (mmol/l)	PLASMA	5.0e-01	9.3e-02	4.9e-01	4.3e-01	5.7e-01	3.2e-01	6.6e-01
Cholesterol esters (mmol/l)	SERUM	4.1e-01	7.2e-02	4.2e-01	3.6e-01	4.5e-01	2.7e-01	5.7e-01
Cholesterol esters (mmol/l)	PLASMA	4.0e-01	7.2e-02	4.0e-01	3.5e-01	4.6e-01	2.7e-01	5.3e-01
Free cholesterol (mmol/l)	SERUM	9.4e-02	2.0e-02	9.4e-02	8.3e-02	1.0e-01	5.5e-02	1.4e-01
Free cholesterol (mmol/l)	PLASMA	9.4e-02	2.1e-02	9.4e-02	8.0e-02	1.1e-01	5.6e-02	1.4e-01
Triglycerides (mmol/l)	SERUM	4.4e-02	1.6e-02	4.1e-02	3.3e-02	5.1e-02	2.7e-02	1.0e-01

Metabolic traits	sample	mean	sd	median	iqr25	iqr75	min	max
Triglycerides (mmol/l)	PLASMA	4.9e-02	1.8e-02	4.3e-02	3.7e-02	5.1e-02	3.2e-02	1.0e-01
Small HDL								
Particle concentration (mol/l)	SERUM	4.9e-06	5.0e-07	4.9e-06	4.6e-06	5.2e-06	3.9e-06	6.4e-06
Particle concentration (mol/l)	PLASMA	5.0e-06	5.7e-07	4.9e-06	4.7e-06	5.4e-06	4.1e-06	6.3e-06
Total lipids (mmol/l)	SERUM	1.1e+00	1.1e-01	1.1e+00	1.0e+00	1.2e+00	8.8e-01	1.4e+00
Total lipids (mmol/l)	PLASMA	1.1e+00	1.2e-01	1.1e+00	1.0e+00	1.2e+00	9.0e-01	1.4e+00
Phospholipids (mmol/l)	SERUM	6.0e-01	7.0e-02	6.0e-01	5.5e-01	6.4e-01	4.6e-01	7.6e-01
Phospholipids (mmol/l)	PLASMA	6.1e-01	7.5e-02	6.1e-01	5.6e-01	6.4e-01	4.9e-01	8.0e-01
Total cholesterol (mmol/l)	SERUM	4.4e-01	6.4e-02	4.4e-01	4.1e-01	4.6e-01	2.4e-01	6.0e-01
Total cholesterol (mmol/l)	PLASMA	4.4e-01	7.8e-02	4.3e-01	4.0e-01	5.1e-01	2.6e-01	5.9e-01
Cholesterol esters (mmol/l)	SERUM	3.3e-01	6.1e-02	3.3e-01	3.0e-01	3.5e-01	1.2e-01	4.7e-01
Cholesterol esters (mmol/l)	PLASMA	3.3e-01	7.4e-02	3.2e-01	2.9e-01	4.0e-01	1.4e-01	4.6e-01
Free cholesterol (mmol/l)	SERUM	1.1e-01	1.2e-02	1.1e-01	1.0e-01	1.2e-01	8.9e-02	1.4e-01
Free cholesterol (mmol/l)	PLASMA	1.1e-01	1.3e-02	1.1e-01	1.0e-01	1.2e-01	9.3e-02	1.5e-01
Triglycerides (mmol/l)	SERUM	4.5e-02	1.9e-02	3.8e-02	3.3e-02	4.7e-02	2.7e-02	1.2e-01
Triglycerides (mmol/l)	PLASMA	5.0e-02	2.1e-02	4.3e-02	3.8e-02	5.3e-02	3.1e-02	1.2e-01
**Lipoprotein particle size**								
VLDL particle size (nm)	SERUM	3.7e+01	1.6e+00	3.6e+01	3.5e+01	3.7e+01	3.4e+01	4.1e+01
VLDL particle size (nm)	PLASMA	3.7e+01	1.6e+00	3.6e+01	3.6e+01	3.7e+01	3.4e+01	4.1e+01
LDL particle size (nm)	SERUM	2.4e+01	8.0e-02	2.4e+01	2.3e+01	2.4e+01	2.3e+01	2.4e+01
LDL particle size (nm)	PLASMA	2.4e+01	8.9e-02	2.4e+01	2.3e+01	2.4e+01	2.3e+01	2.4e+01
HDL particle size (nm)	SERUM	1.0e+01	2.8e-01	1.0e+01	9.9e+00	1.0e+01	9.6e+00	1.1e+01
HDL particle size (nm)	PLASMA	1.0e+01	3.0e-01	1.0e+01	9.9e+00	1.0e+01	9.6e+00	1.1e+01
**Cholesterol**								
Total cholesterol (mmol/l)	SERUM	4.3e+00	7.4e-01	4.3e+00	3.7e+00	4.8e+00	2.9e+00	5.7e+00
Total cholesterol (mmol/l)	PLASMA	4.0e+00	7.5e-01	4.1e+00	3.4e+00	4.7e+00	2.7e+00	5.3e+00
VLDL cholesterol (mmol/l)	SERUM	5.4e-01	3.4e-01	4.5e-01	3.7e-01	5.9e-01	1.6e-01	2.0e+00
VLDL cholesterol (mmol/l)	PLASMA	5.2e-01	4.0e-01	3.6e-01	3.1e-01	5.8e-01	2.0e-01	1.9e+00
Remnant cholesterol (mmol/l)	SERUM	1.1e+00	4.0e-01	1.0e+00	9.1e-01	1.2e+00	5.0e-01	2.6e+00

Metabolic traits	sample	mean	sd	median	iqr25	iqr75	min	max
Remnant cholesterol (mmol/l)	PLASMA	1.0e+00	4.8e-01	8.7e-01	7.4e-01	1.1e+00	5.4e-01	2.5e+00
LDL cholesterol (mmol/l)	SERUM	1.4e+00	3.6e-01	1.4e+00	1.2e+00	1.7e+00	7.5e-01	2.3e+00
LDL cholesterol (mmol/l)	PLASMA	1.3e+00	3.7e-01	1.2e+00	1.1e+00	1.6e+00	7.0e-01	2.1e+00
HDL cholesterol (mmol/l)	SERUM	1.7e+00	4.1e-01	1.6e+00	1.4e+00	2.1e+00	8.5e-01	2.5e+00
HDL cholesterol (mmol/l)	PLASMA	1.6e+00	4.1e-01	1.6e+00	1.4e+00	2.0e+00	8.2e-01	2.3e+00
HDL2 cholesterol (mmol/l)	SERUM	1.2e+00	3.8e-01	1.1e+00	9.7e-01	1.6e+00	4.5e-01	1.9e+00
HDL2 cholesterol (mmol/l)	PLASMA	1.2e+00	3.7e-01	1.1e+00	9.9e-01	1.5e+00	4.3e-01	1.8e+00
HDL3 cholesterol (mmol/l)	SERUM	4.8e-01	3.6e-02	4.8e-01	4.6e-01	5.1e-01	4.0e-01	5.5e-01
HDL3 cholesterol (mmol/l)	PLASMA	4.8e-01	3.6e-02	4.8e-01	4.4e-01	5.0e-01	3.9e-01	5.3e-01
Esterified cholesterol (mmol/l)	SERUM	2.9e+00	5.2e-01	2.9e+00	2.6e+00	3.3e+00	1.9e+00	4.1e+00
Esterified cholesterol (mmol/l)	PLASMA	2.8e+00	5.4e-01	2.7e+00	2.4e+00	3.3e+00	1.9e+00	3.8e+00
Free cholesterol (mmol/l)	SERUM	1.3e+00	2.3e-01	1.3e+00	1.1e+00	1.5e+00	9.3e-01	1.8e+00
Free cholesterol (mmol/l)	PLASMA	1.2e+00	2.2e-01	1.2e+00	1.0e+00	1.3e+00	8.3e-01	1.6e+00
**Glycerides and phospholipids**								
Triglycerides (mmol/l)	SERUM	1.2e+00	8.9e-01	8.9e-01	6.9e-01	1.3e+00	4.7e-01	5.0e+00
Triglycerides (mmol/l)	PLASMA	1.3e+00	1.0e+00	8.9e-01	7.8e-01	1.3e+00	5.3e-01	5.0e+00
VLDL triglycerides (mmol/l)	SERUM	7.8e-01	8.0e-01	5.3e-01	3.9e-01	8.6e-01	1.6e-01	4.2e+00
VLDL triglycerides (mmol/l)	PLASMA	8.6e-01	9.1e-01	5.3e-01	4.3e-01	8.3e-01	2.0e-01	4.2e+00
LDL triglycerides (mmol/l)	SERUM	1.6e-01	4.5e-02	1.6e-01	1.3e-01	1.9e-01	8.2e-02	3.0e-01
LDL triglycerides (mmol/l)	PLASMA	1.7e-01	5.0e-02	1.7e-01	1.3e-01	1.9e-01	7.7e-02	3.0e-01
HDL triglycerides (mmol/l)	SERUM	1.4e-01	4.3e-02	1.3e-01	1.1e-01	1.5e-01	8.6e-02	3.2e-01
HDL triglycerides (mmol/l)	PLASMA	1.5e-01	4.9e-02	1.4e-01	1.1e-01	1.7e-01	9.9e-02	3.1e-01
Diacylglycerol (mmol/l)	SERUM	1.6e-02	2.0e-02	1.3e-02	0.0e+00	2.2e-02	0.0e+00	9.3e-02
Diacylglycerol (mmol/l)	PLASMA	1.7e-02	2.5e-02	9.0e-03	2.3e-04	2.6e-02	0.0e+00	1.1e-01
Phosphoglycerides (mmol/l)	SERUM	2.0e+00	3.8e-01	1.9e+00	1.7e+00	2.3e+00	1.5e+00	2.8e+00
Phosphoglycerides (mmol/l)	PLASMA	1.9e+00	4.0e-01	1.9e+00	1.5e+00	2.2e+00	1.4e+00	2.7e+00
Phosphatidylcholine + other cholines (mmol/l)	SERUM	2.0e+00	3.6e-01	2.0e+00	1.7e+00	2.3e+00	1.5e+00	2.7e+00
Phosphatidylcholine + other cholines (mmol/l)	PLASMA	1.9e+00	3.7e-01	1.9e+00	1.6e+00	2.2e+00	1.5e+00	2.6e+00
Sphingomyelins (mmol/l)	SERUM	4.4e-01	7.3e-02	4.5e-01	3.8e-01	5.2e-01	3.2e-01	5.7e-01

Metabolic traits	sample	mean	sd	median	iqr25	iqr75	min	max
Sphingomyelins (mmol/l)	PLASMA	4.3e-01	8.6e-02	4.2e-01	3.7e-01	4.8e-01	2.9e-01	6.2e-01
Cholines (mmol/l)	SERUM	2.4e+00	4.2e-01	2.4e+00	2.0e+00	2.7e+00	1.3e+00	3.1e+00
Cholines (mmol/l)	PLASMA	2.3e+00	4.1e-01	2.3e+00	1.9e+00	2.6e+00	1.7e+00	3.0e+00
**Apolipoproteins**								
Apolipoprotein A-I (g/l)	SERUM	1.7e+00	2.1e-01	1.6e+00	1.5e+00	1.8e+00	1.3e+00	2.1e+00
Apolipoprotein A-I (g/l)	PLASMA	1.6e+00	1.9e-01	1.6e+00	1.5e+00	1.8e+00	1.3e+00	2.0e+00
Apolipoprotein B (g/l)	SERUM	7.8e-01	2.1e-01	7.3e-01	6.7e-01	8.1e-01	4.6e-01	1.5e+00
Apolipoprotein B (g/l)	PLASMA	7.4e-01	2.6e-01	6.5e-01	5.8e-01	7.8e-01	5.0e-01	1.6e+00
**Fatty acids**								
Total fatty acids (mmol/l)	SERUM	1.1e+01	2.7e+00	1.1e+01	8.8e+00	1.2e+01	7.2e+00	2.1e+01
Total fatty acids (mmol/l)	PLASMA	1.1e+01	3.4e+00	1.0e+01	8.3e+00	1.1e+01	6.8e+00	2.1e+01
Fatty acid chain length	SERUM	1.7e+01	2.6e-01	1.7e+01	1.7e+01	1.8e+01	1.7e+01	1.8e+01
Fatty acid chain length	PLASMA	1.7e+01	2.6e-01	1.7e+01	1.7e+01	1.8e+01	1.7e+01	1.8e+01
Degree of unsaturation	SERUM	1.2e+00	6.9e-02	1.2e+00	1.2e+00	1.3e+00	1.0e+00	1.3e+00
Degree of unsaturation	PLASMA	1.2e+00	7.3e-02	1.2e+00	1.2e+00	1.2e+00	1.1e+00	1.3e+00
Docosahexaenoic acid (mmol/l)	SERUM	1.3e-01	4.7e-02	1.2e-01	9.8e-02	1.5e-01	4.5e-02	2.3e-01
Docosahexaenoic acid (mmol/l)	PLASMA	1.3e-01	4.9e-02	1.2e-01	9.8e-02	1.7e-01	6.6e-02	2.4e-01
Linoleic acid (mmol/l)	SERUM	2.8e+00	5.4e-01	2.8e+00	2.4e+00	3.2e+00	1.9e+00	4.5e+00
Linoleic acid (mmol/l)	PLASMA	2.8e+00	6.2e-01	2.7e+00	2.2e+00	3.1e+00	1.9e+00	4.5e+00
Conjugated linoleic acid (mmol/l)	SERUM	2.7e-02	1.9e-02	2.5e-02	1.3e-02	3.3e-02	3.0e-03	8.8e-02
Conjugated linoleic acid (mmol/l)	PLASMA	3.1e-02	2.0e-02	2.7e-02	2.0e-02	3.5e-02	0.0e+00	9.5e-02
n-3 fatty acids (mmol/l)	SERUM	4.1e-01	1.2e-01	3.7e-01	3.3e-01	4.6e-01	2.4e-01	8.1e-01
n-3 fatty acids (mmol/l)	PLASMA	4.2e-01	1.5e-01	3.5e-01	3.1e-01	4.9e-01	2.3e-01	8.8e-01
n-6 fatty acids (mmol/l)	SERUM	3.6e+00	6.2e-01	3.5e+00	3.0e+00	4.0e+00	2.5e+00	5.4e+00
n-6 fatty acids (mmol/l)	PLASMA	3.4e+00	6.9e-01	3.6e+00	2.8e+00	3.8e+00	2.4e+00	5.3e+00
PUFA (mmol/l)	SERUM	4.0e+00	7.2e-01	3.8e+00	3.4e+00	4.5e+00	2.8e+00	6.2e+00
PUFA (mmol/l)	PLASMA	3.8e+00	8.3e-01	3.9e+00	3.1e+00	4.3e+00	2.7e+00	6.2e+00
MUFA (mmol/l)	SERUM	2.8e+00	1.1e+00	2.5e+00	2.0e+00	3.0e+00	1.4e+00	7.1e+00
MUFA (mmol/l)	PLASMA	2.7e+00	1.3e+00	2.3e+00	1.8e+00	2.8e+00	1.3e+00	6.9e+00

Metabolic traits	sample	mean	sd	median	iqr25	iqr75	min	max
Saturated fatty acids (mmol/l)	SERUM	4.2e+00	1.0e+00	4.0e+00	3.4e+00	4.4e+00	3.0e+00	7.5e+00
Saturated fatty acids (mmol/l)	PLASMA	4.1e+00	1.3e+00	3.9e+00	3.2e+00	4.3e+00	2.8e+00	8.0e+00
**Glycolysis related metabolites**								
Glucose (mmol/l)	SERUM	4.2e+00	5.8e-01	4.1e+00	3.8e+00	4.6e+00	2.6e+00	5.5e+00
Glucose (mmol/l)	PLASMA	4.1e+00	5.2e-01	4.2e+00	4.0e+00	4.3e+00	2.6e+00	5.4e+00
Lactate (mmol/l)	SERUM	1.5e+00	5.5e-01	1.4e+00	1.2e+00	1.6e+00	8.3e-01	4.0e+00
Lactate (mmol/l)	PLASMA	1.2e+00	3.3e-01	1.1e+00	9.7e-01	1.4e+00	6.0e-01	2.1e+00
Pyruvate (mmol/l)	SERUM	8.5e-02	3.4e-02	8.2e-02	5.9e-02	1.1e-01	2.5e-02	1.7e-01
Citrate (mmol/l)	SERUM	1.0e-01	2.7e-02	9.6e-02	8.5e-02	1.1e-01	6.3e-02	2.0e-01
Citrate (mmol/l)	PLASMA	1.7e-01	3.7e-02	1.7e-01	1.4e-01	2.0e-01	1.1e-01	2.5e-01
Glycerol (mmol/l)	SERUM	5.5e-02	1.9e-02	5.2e-02	4.6e-02	6.6e-02	1.6e-02	1.1e-01
**Amino acids**								
Alanine (mmol/l)	SERUM	4.3e-01	5.5e-02	4.3e-01	3.9e-01	4.6e-01	3.2e-01	5.9e-01
Alanine (mmol/l)	PLASMA	4.1e-01	5.3e-02	4.1e-01	3.8e-01	4.3e-01	3.5e-01	5.6e-01
Glutamine (mmol/l)	SERUM	5.2e-01	6.0e-02	5.2e-01	4.9e-01	5.6e-01	3.8e-01	6.5e-01
Glutamine (mmol/l)	PLASMA	5.1e-01	5.7e-02	5.1e-01	4.7e-01	5.4e-01	3.9e-01	6.2e-01
Glycine (mmol/l)	SERUM	2.6e-01	7.6e-02	2.3e-01	2.1e-01	2.7e-01	1.7e-01	4.8e-01
Histidine (mmol/l)	SERUM	6.4e-02	6.5e-03	6.4e-02	6.1e-02	6.9e-02	5.2e-02	8.1e-02
Histidine (mmol/l)	PLASMA	6.3e-02	8.4e-03	6.2e-02	5.8e-02	7.0e-02	4.8e-02	8.0e-02
Branched-chain amino acids								
Isoleucine (mmol/l)	SERUM	5.6e-02	2.1e-02	4.8e-02	4.2e-02	6.4e-02	3.7e-02	1.3e-01
Isoleucine (mmol/l)	PLASMA	6.0e-02	2.5e-02	5.0e-02	4.5e-02	6.6e-02	3.0e-02	1.2e-01
Leucine (mmol/l)	SERUM	7.1e-02	1.9e-02	6.4e-02	5.8e-02	7.9e-02	4.2e-02	1.2e-01
Leucine (mmol/l)	PLASMA	7.1e-02	2.2e-02	6.5e-02	5.7e-02	7.4e-02	4.1e-02	1.2e-01
Valine (mmol/l)	SERUM	1.6e-01	3.2e-02	1.5e-01	1.3e-01	1.7e-01	1.1e-01	2.5e-01
Valine (mmol/l)	PLASMA	1.5e-01	3.6e-02	1.5e-01	1.3e-01	1.6e-01	1.0e-01	2.5e-01
Aromatic amino acids								
Phenylalanine (mmol/l)	SERUM	6.1e-02	6.9e-03	5.9e-02	5.5e-02	6.5e-02	4.9e-02	7.8e-02
Phenylalanine (mmol/l)	PLASMA	5.7e-02	6.5e-03	5.6e-02	5.4e-02	5.9e-02	4.7e-02	7.2e-02

Metabolic traits	sample	mean	sd	median	iqr25	iqr75	min	max
Tyrosine (mmol/l)	SERUM	5.3e-02	1.3e-02	4.9e-02	4.5e-02	5.8e-02	3.6e-02	8.8e-02
Tyrosine (mmol/l)	PLASMA	5.5e-02	1.2e-02	5.2e-02	4.8e-02	6.2e-02	3.9e-02	9.2e-02
**Ketone bodies**								
Acetate (mmol/l)	SERUM	3.7e-02	9.7e-03	3.4e-02	3.1e-02	3.8e-02	2.7e-02	7.2e-02
Acetate (mmol/l)	PLASMA	4.7e-02	1.0e-02	4.4e-02	4.1e-02	4.8e-02	3.7e-02	7.4e-02
Beta-hydroxybutyrate (mmol/l)	SERUM	8.4e-02	2.5e-02	8.4e-02	7.0e-02	9.3e-02	5.0e-02	2.0e-01
Beta-hydroxybutyrate (mmol/l)	PLASMA	8.8e-02	1.5e-02	9.0e-02	7.9e-02	9.9e-02	5.4e-02	1.2e-01
**Fluid balance**								
Creatinine (mmol/l)	SERUM	5.6e-02	8.7e-03	5.5e-02	4.9e-02	6.2e-02	4.0e-02	7.1e-02
Creatinine (mmol/l)	PLASMA	5.7e-02	8.8e-03	5.5e-02	5.0e-02	6.5e-02	4.1e-02	7.2e-02
Albumin (signal area)	SERUM	9.3e-02	4.0e-03	9.3e-02	9.1e-02	9.5e-02	8.4e-02	1.0e-01
Albumin (signal area)	PLASMA	9.3e-02	3.6e-03	9.2e-02	9.1e-02	9.5e-02	8.6e-02	1.0e-01
**Inflammation**								
Glycoprotein acetyls (mmol/l)	SERUM	1.3e+00	2.9e-01	1.3e+00	1.1e+00	1.4e+00	1.0e+00	2.6e+00
Glycoprotein acetyls (mmol/l)	PLASMA	1.3e+00	3.3e-01	1.3e+00	1.1e+00	1.3e+00	1.0e+00	2.5e+00

Table S2. Spearman's correlation: serum, pre-storage handling effects. Spearman's rank correlation coefficients between metabolic trait concentrations (or values) in reference samples (condition: 4 ºC, 1.5 h) and samples incubated at (i) 4 ºC, 24 h; (ii) 4 ºC, 48 h; (iii) 21 ºC, 24 h; (iv) 21 ºC, 48 h, before centrifugation (see Figures 1-2, S1).

***Abbreviations**: **C**=cholesterol; **IDL**=intermediate-density lipoprotein; **LCI**=lower confidence interval; **LDL**=low-density lipoprotein; **HDL**=high-density lipoprotein; **MUFA**=monounsaturated fatty acids; **PUFA**=polyunsaturated fatty acids; **UCI**= upper confidence interval; **VLDL**=very-low-density lipoprotein.*

Metabolic traits	conditions	correlation	LCI	UCI
**Lipoprotein subclasses**				
Extremely large VLDL				
Particle concentration (mol/l)	4°C,24h	9.2e-01	7.6e-01	9.9e-01
Particle concentration (mol/l)	4°C,48h	9.1e-01	7.7e-01	9.9e-01
Particle concentration (mol/l)	21°C,24h	8.9e-01	7.0e-01	9.6e-01
Particle concentration (mol/l)	21°C,48h	7.7e-01	4.7e-01	9.3e-01
Total lipids (mmol/l)	4°C,24h	9.2e-01	7.6e-01	9.8e-01
Total lipids (mmol/l)	4°C,48h	9.1e-01	7.3e-01	9.8e-01
Total lipids (mmol/l)	21°C,24h	9.0e-01	7.1e-01	9.7e-01
Total lipids (mmol/l)	21°C,48h	7.7e-01	4.7e-01	9.4e-01
Phospholipids (mmol/l)	4°C,24h	8.9e-01	6.9e-01	9.8e-01
Phospholipids (mmol/l)	4°C,48h	9.2e-01	7.4e-01	9.9e-01
Phospholipids (mmol/l)	21°C,24h	9.1e-01	6.9e-01	9.7e-01
Phospholipids (mmol/l)	21°C,48h	7.9e-01	5.0e-01	9.4e-01
Total cholesterol (mmol/l)	4°C,24h	9.5e-01	8.2e-01	9.9e-01
Total cholesterol (mmol/l)	4°C,48h	9.5e-01	8.4e-01	9.9e-01
Total cholesterol (mmol/l)	21°C,24h	9.4e-01	7.9e-01	9.8e-01
Total cholesterol (mmol/l)	21°C,48h	8.5e-01	6.2e-01	9.5e-01
Cholesterol esters (mmol/l)	4°C,24h	9.5e-01	8.3e-01	9.9e-01
Cholesterol esters (mmol/l)	4°C,48h	9.5e-01	8.0e-01	9.9e-01
Cholesterol esters (mmol/l)	21°C,24h	9.3e-01	7.8e-01	9.9e-01
Cholesterol esters (mmol/l)	21°C,48h	8.1e-01	5.1e-01	9.3e-01
Free cholesterol (mmol/l)	4°C,24h	9.1e-01	7.3e-01	1.0e+00
Free cholesterol (mmol/l)	4°C,48h	9.3e-01	7.8e-01	9.9e-01

Metabolic traits	conditions	correlation	LCI	UCI
Free cholesterol (mmol/l)	21°C,24h	9.3e-01	7.6e-01	9.8e-01
Free cholesterol (mmol/l)	21°C,48h	8.3e-01	5.4e-01	9.5e-01
Triglycerides (mmol/l)	4°C,24h	9.1e-01	7.3e-01	9.9e-01
Triglycerides (mmol/l)	4°C,48h	9.1e-01	7.1e-01	9.8e-01
Triglycerides (mmol/l)	21°C,24h	8.8e-01	6.9e-01	9.6e-01
Triglycerides (mmol/l)	21°C,48h	7.6e-01	3.8e-01	9.3e-01
Very large VLDL				
Particle concentration (mol/l)	4°C,24h	9.6e-01	8.5e-01	1.0e+00
Particle concentration (mol/l)	4°C,48h	9.6e-01	8.8e-01	9.9e-01
Particle concentration (mol/l)	21°C,24h	9.5e-01	8.6e-01	9.9e-01
Particle concentration (mol/l)	21°C,48h	8.3e-01	5.6e-01	9.7e-01
Total lipids (mmol/l)	4°C,24h	9.6e-01	8.3e-01	9.9e-01
Total lipids (mmol/l)	4°C,48h	9.6e-01	8.7e-01	9.9e-01
Total lipids (mmol/l)	21°C,24h	9.5e-01	8.6e-01	9.9e-01
Total lipids (mmol/l)	21°C,48h	8.3e-01	5.9e-01	9.6e-01
Phospholipids (mmol/l)	4°C,24h	9.7e-01	8.7e-01	9.9e-01
Phospholipids (mmol/l)	4°C,48h	9.7e-01	9.0e-01	1.0e+00
Phospholipids (mmol/l)	21°C,24h	9.7e-01	8.9e-01	9.9e-01
Phospholipids (mmol/l)	21°C,48h	8.5e-01	5.7e-01	9.7e-01
Total cholesterol (mmol/l)	4°C,24h	9.7e-01	8.9e-01	1.0e+00
Total cholesterol (mmol/l)	4°C,48h	9.7e-01	9.0e-01	1.0e+00
Total cholesterol (mmol/l)	21°C,24h	9.7e-01	8.9e-01	9.9e-01
Total cholesterol (mmol/l)	21°C,48h	8.6e-01	6.3e-01	9.7e-01
Cholesterol esters (mmol/l)	4°C,24h	9.7e-01	8.8e-01	1.0e+00
Cholesterol esters (mmol/l)	4°C,48h	9.7e-01	8.8e-01	1.0e+00
Cholesterol esters (mmol/l)	21°C,24h	9.7e-01	8.9e-01	9.9e-01
Cholesterol esters (mmol/l)	21°C,48h	8.6e-01	6.2e-01	9.6e-01
Free cholesterol (mmol/l)	4°C,24h	9.7e-01	8.6e-01	9.9e-01
Free cholesterol (mmol/l)	4°C,48h	9.7e-01	9.0e-01	9.9e-01

Metabolic traits	conditions	correlation	LCI	UCI
Free cholesterol (mmol/l)	21°C,24h	9.7e-01	8.8e-01	9.9e-01
Free cholesterol (mmol/l)	21°C,48h	8.5e-01	6.0e-01	9.6e-01
Triglycerides (mmol/l)	4°C,24h	9.6e-01	8.7e-01	9.9e-01
Triglycerides (mmol/l)	4°C,48h	9.6e-01	8.6e-01	9.9e-01
Triglycerides (mmol/l)	21°C,24h	9.5e-01	8.3e-01	9.9e-01
Triglycerides (mmol/l)	21°C,48h	8.3e-01	5.7e-01	9.6e-01
Large VLDL				
Particle concentration (mol/l)	4°C,24h	9.9e-01	9.4e-01	1.0e+00
Particle concentration (mol/l)	4°C,48h	9.8e-01	9.1e-01	1.0e+00
Particle concentration (mol/l)	21°C,24h	9.6e-01	8.4e-01	1.0e+00
Particle concentration (mol/l)	21°C,48h	9.0e-01	6.8e-01	9.8e-01
Total lipids (mmol/l)	4°C,24h	9.8e-01	9.3e-01	1.0e+00
Total lipids (mmol/l)	4°C,48h	9.8e-01	9.0e-01	1.0e+00
Total lipids (mmol/l)	21°C,24h	9.6e-01	8.7e-01	1.0e+00
Total lipids (mmol/l)	21°C,48h	9.0e-01	6.8e-01	9.8e-01
Phospholipids (mmol/l)	4°C,24h	9.8e-01	9.1e-01	1.0e+00
Phospholipids (mmol/l)	4°C,48h	9.8e-01	9.2e-01	1.0e+00
Phospholipids (mmol/l)	21°C,24h	9.6e-01	8.6e-01	9.9e-01
Phospholipids (mmol/l)	21°C,48h	8.9e-01	6.8e-01	9.8e-01
Total cholesterol (mmol/l)	4°C,24h	9.8e-01	9.0e-01	1.0e+00
Total cholesterol (mmol/l)	4°C,48h	9.8e-01	9.4e-01	1.0e+00
Total cholesterol (mmol/l)	21°C,24h	9.7e-01	8.9e-01	9.9e-01
Total cholesterol (mmol/l)	21°C,48h	8.8e-01	6.8e-01	9.8e-01
Cholesterol esters (mmol/l)	4°C,24h	9.7e-01	8.7e-01	9.9e-01
Cholesterol esters (mmol/l)	4°C,48h	9.7e-01	8.6e-01	9.9e-01
Cholesterol esters (mmol/l)	21°C,24h	9.6e-01	8.6e-01	9.9e-01
Cholesterol esters (mmol/l)	21°C,48h	8.2e-01	5.7e-01	9.4e-01
Free cholesterol (mmol/l)	4°C,24h	9.8e-01	9.1e-01	1.0e+00
Free cholesterol (mmol/l)	4°C,48h	9.9e-01	9.2e-01	1.0e+00

Metabolic traits	conditions	correlation	LCI	UCI
Free cholesterol (mmol/l)	21°C,24h	9.8e-01	9.0e-01	1.0e+00
Free cholesterol (mmol/l)	21°C,48h	9.1e-01	7.1e-01	9.8e-01
Triglycerides (mmol/l)	4°C,24h	9.8e-01	9.1e-01	1.0e+00
Triglycerides (mmol/l)	4°C,48h	9.9e-01	9.3e-01	1.0e+00
Triglycerides (mmol/l)	21°C,24h	9.6e-01	8.6e-01	9.9e-01
Triglycerides (mmol/l)	21°C,48h	9.0e-01	6.8e-01	9.7e-01
Medium VLDL				
Particle concentration (mol/l)	4°C,24h	9.7e-01	8.8e-01	1.0e+00
Particle concentration (mol/l)	4°C,48h	9.8e-01	9.0e-01	1.0e+00
Particle concentration (mol/l)	21°C,24h	9.7e-01	8.7e-01	1.0e+00
Particle concentration (mol/l)	21°C,48h	9.0e-01	7.0e-01	9.7e-01
Total lipids (mmol/l)	4°C,24h	9.7e-01	8.6e-01	1.0e+00
Total lipids (mmol/l)	4°C,48h	9.9e-01	9.3e-01	1.0e+00
Total lipids (mmol/l)	21°C,24h	9.6e-01	8.6e-01	1.0e+00
Total lipids (mmol/l)	21°C,48h	8.9e-01	6.8e-01	9.8e-01
Phospholipids (mmol/l)	4°C,24h	9.8e-01	8.8e-01	1.0e+00
Phospholipids (mmol/l)	4°C,48h	9.8e-01	9.2e-01	1.0e+00
Phospholipids (mmol/l)	21°C,24h	9.6e-01	8.7e-01	9.9e-01
Phospholipids (mmol/l)	21°C,48h	8.9e-01	6.7e-01	9.7e-01
Total cholesterol (mmol/l)	4°C,24h	9.7e-01	8.7e-01	1.0e+00
Total cholesterol (mmol/l)	4°C,48h	9.7e-01	8.9e-01	1.0e+00
Total cholesterol (mmol/l)	21°C,24h	9.8e-01	9.0e-01	1.0e+00
Total cholesterol (mmol/l)	21°C,48h	8.8e-01	6.1e-01	9.8e-01
Cholesterol esters (mmol/l)	4°C,24h	9.7e-01	8.4e-01	1.0e+00
Cholesterol esters (mmol/l)	4°C,48h	9.7e-01	8.7e-01	1.0e+00
Cholesterol esters (mmol/l)	21°C,24h	9.4e-01	7.8e-01	1.0e+00
Cholesterol esters (mmol/l)	21°C,48h	8.6e-01	6.2e-01	9.8e-01
Free cholesterol (mmol/l)	4°C,24h	9.9e-01	9.6e-01	1.0e+00
Free cholesterol (mmol/l)	4°C,48h	9.9e-01	9.3e-01	1.0e+00

Metabolic traits	conditions	correlation	LCI	UCI
Free cholesterol (mmol/l)	21°C,24h	9.7e-01	8.9e-01	1.0e+00
Free cholesterol (mmol/l)	21°C,48h	9.1e-01	7.2e-01	9.9e-01
Triglycerides (mmol/l)	4°C,24h	9.8e-01	9.0e-01	1.0e+00
Triglycerides (mmol/l)	4°C,48h	9.9e-01	9.2e-01	1.0e+00
Triglycerides (mmol/l)	21°C,24h	9.7e-01	8.8e-01	1.0e+00
Triglycerides (mmol/l)	21°C,48h	9.2e-01	7.5e-01	9.8e-01
Small VLDL				
Particle concentration (mol/l)	4°C,24h	9.8e-01	9.1e-01	1.0e+00
Particle concentration (mol/l)	4°C,48h	9.8e-01	9.1e-01	1.0e+00
Particle concentration (mol/l)	21°C,24h	9.6e-01	8.5e-01	9.9e-01
Particle concentration (mol/l)	21°C,48h	9.2e-01	7.5e-01	9.8e-01
Total lipids (mmol/l)	4°C,24h	9.7e-01	8.6e-01	1.0e+00
Total lipids (mmol/l)	4°C,48h	9.8e-01	9.2e-01	1.0e+00
Total lipids (mmol/l)	21°C,24h	9.7e-01	8.8e-01	9.9e-01
Total lipids (mmol/l)	21°C,48h	9.3e-01	7.5e-01	9.9e-01
Phospholipids (mmol/l)	4°C,24h	9.7e-01	8.9e-01	1.0e+00
Phospholipids (mmol/l)	4°C,48h	9.7e-01	8.8e-01	1.0e+00
Phospholipids (mmol/l)	21°C,24h	9.9e-01	9.4e-01	1.0e+00
Phospholipids (mmol/l)	21°C,48h	9.5e-01	8.6e-01	9.9e-01
Total cholesterol (mmol/l)	4°C,24h	9.8e-01	8.7e-01	1.0e+00
Total cholesterol (mmol/l)	4°C,48h	9.7e-01	8.7e-01	1.0e+00
Total cholesterol (mmol/l)	21°C,24h	9.8e-01	8.9e-01	1.0e+00
Total cholesterol (mmol/l)	21°C,48h	9.2e-01	7.6e-01	9.8e-01
Cholesterol esters (mmol/l)	4°C,24h	9.8e-01	9.3e-01	1.0e+00
Cholesterol esters (mmol/l)	4°C,48h	9.7e-01	8.8e-01	1.0e+00
Cholesterol esters (mmol/l)	21°C,24h	9.6e-01	8.1e-01	9.9e-01
Cholesterol esters (mmol/l)	21°C,48h	9.0e-01	7.0e-01	9.7e-01
Free cholesterol (mmol/l)	4°C,24h	9.9e-01	9.4e-01	1.0e+00
Free cholesterol (mmol/l)	4°C,48h	9.9e-01	9.5e-01	1.0e+00

Metabolic traits	conditions	correlation	LCI	UCI
Free cholesterol (mmol/l)	21°C,24h	9.9e-01	9.5e-01	1.0e+00
Free cholesterol (mmol/l)	21°C,48h	9.5e-01	8.1e-01	9.9e-01
Triglycerides (mmol/l)	4°C,24h	9.7e-01	8.5e-01	1.0e+00
Triglycerides (mmol/l)	4°C,48h	9.6e-01	8.6e-01	9.9e-01
Triglycerides (mmol/l)	21°C,24h	9.6e-01	8.5e-01	9.9e-01
Triglycerides (mmol/l)	21°C,48h	9.0e-01	6.9e-01	9.7e-01
Very Small VLDL				
Particle concentration (mol/l)	4°C,24h	1.0e+00	9.6e-01	1.0e+00
Particle concentration (mol/l)	4°C,48h	9.9e-01	9.4e-01	1.0e+00
Particle concentration (mol/l)	21°C,24h	9.7e-01	8.6e-01	1.0e+00
Particle concentration (mol/l)	21°C,48h	9.2e-01	7.6e-01	9.8e-01
Total lipids (mmol/l)	4°C,24h	9.9e-01	9.6e-01	1.0e+00
Total lipids (mmol/l)	4°C,48h	9.8e-01	9.2e-01	1.0e+00
Total lipids (mmol/l)	21°C,24h	9.7e-01	8.6e-01	1.0e+00
Total lipids (mmol/l)	21°C,48h	9.2e-01	7.7e-01	9.8e-01
Phospholipids (mmol/l)	4°C,24h	9.8e-01	9.1e-01	1.0e+00
Phospholipids (mmol/l)	4°C,48h	9.6e-01	8.4e-01	9.8e-01
Phospholipids (mmol/l)	21°C,24h	9.5e-01	8.5e-01	9.9e-01
Phospholipids (mmol/l)	21°C,48h	9.0e-01	7.2e-01	9.6e-01
Total cholesterol (mmol/l)	4°C,24h	9.7e-01	8.8e-01	9.9e-01
Total cholesterol (mmol/l)	4°C,48h	9.4e-01	7.8e-01	9.8e-01
Total cholesterol (mmol/l)	21°C,24h	9.4e-01	8.2e-01	9.9e-01
Total cholesterol (mmol/l)	21°C,48h	8.2e-01	5.4e-01	9.3e-01
Cholesterol esters (mmol/l)	4°C,24h	9.7e-01	9.1e-01	9.9e-01
Cholesterol esters (mmol/l)	4°C,48h	9.5e-01	8.1e-01	9.8e-01
Cholesterol esters (mmol/l)	21°C,24h	9.5e-01	8.3e-01	9.8e-01
Cholesterol esters (mmol/l)	21°C,48h	7.7e-01	4.8e-01	9.4e-01
Free cholesterol (mmol/l)	4°C,24h	9.7e-01	8.9e-01	9.9e-01
Free cholesterol (mmol/l)	4°C,48h	9.3e-01	8.0e-01	9.8e-01

Metabolic traits	conditions	correlation	LCI	UCI
Free cholesterol (mmol/l)	21°C,24h	9.4e-01	8.0e-01	9.8e-01
Free cholesterol (mmol/l)	21°C,48h	9.1e-01	7.3e-01	9.6e-01
Triglycerides (mmol/l)	4°C,24h	9.9e-01	9.4e-01	1.0e+00
Triglycerides (mmol/l)	4°C,48h	9.8e-01	9.3e-01	1.0e+00
Triglycerides (mmol/l)	21°C,24h	9.9e-01	9.2e-01	1.0e+00
Triglycerides (mmol/l)	21°C,48h	9.4e-01	7.9e-01	9.9e-01
IDL				
Particle concentration (mol/l)	4°C,24h	9.7e-01	9.1e-01	9.9e-01
Particle concentration (mol/l)	4°C,48h	9.7e-01	8.7e-01	9.9e-01
Particle concentration (mol/l)	21°C,24h	9.5e-01	8.5e-01	9.9e-01
Particle concentration (mol/l)	21°C,48h	9.2e-01	7.7e-01	9.8e-01
Total lipids (mmol/l)	4°C,24h	9.7e-01	8.9e-01	1.0e+00
Total lipids (mmol/l)	4°C,48h	9.7e-01	8.6e-01	9.9e-01
Total lipids (mmol/l)	21°C,24h	9.3e-01	8.1e-01	9.8e-01
Total lipids (mmol/l)	21°C,48h	9.0e-01	7.1e-01	9.8e-01
Phospholipids (mmol/l)	4°C,24h	9.5e-01	8.7e-01	9.8e-01
Phospholipids (mmol/l)	4°C,48h	9.4e-01	8.3e-01	9.9e-01
Phospholipids (mmol/l)	21°C,24h	8.9e-01	6.7e-01	9.8e-01
Phospholipids (mmol/l)	21°C,48h	8.8e-01	6.7e-01	9.7e-01
Total cholesterol (mmol/l)	4°C,24h	9.3e-01	8.0e-01	9.8e-01
Total cholesterol (mmol/l)	4°C,48h	9.3e-01	7.6e-01	9.8e-01
Total cholesterol (mmol/l)	21°C,24h	9.3e-01	7.5e-01	9.8e-01
Total cholesterol (mmol/l)	21°C,48h	8.8e-01	6.7e-01	9.6e-01
Cholesterol esters (mmol/l)	4°C,24h	9.7e-01	8.8e-01	9.9e-01
Cholesterol esters (mmol/l)	4°C,48h	9.5e-01	8.2e-01	9.9e-01
Cholesterol esters (mmol/l)	21°C,24h	9.5e-01	8.3e-01	9.9e-01
Cholesterol esters (mmol/l)	21°C,48h	9.0e-01	7.0e-01	9.7e-01
Free cholesterol (mmol/l)	4°C,24h	9.3e-01	7.9e-01	9.7e-01
Free cholesterol (mmol/l)	4°C,48h	9.2e-01	7.4e-01	9.8e-01

Metabolic traits	conditions	correlation	LCI	UCI
Free cholesterol (mmol/l)	21°C,24h	8.4e-01	6.0e-01	9.6e-01
Free cholesterol (mmol/l)	21°C,48h	8.0e-01	5.3e-01	9.1e-01
Triglycerides (mmol/l)	4°C,24h	1.0e+00	1.0e+00	1.0e+00
Triglycerides (mmol/l)	4°C,48h	9.9e-01	9.5e-01	1.0e+00
Triglycerides (mmol/l)	21°C,24h	9.7e-01	8.9e-01	9.9e-01
Triglycerides (mmol/l)	21°C,48h	9.5e-01	8.6e-01	9.8e-01
Large LDL				
Particle concentration (mol/l)	4°C,24h	9.5e-01	8.4e-01	9.8e-01
Particle concentration (mol/l)	4°C,48h	9.3e-01	8.0e-01	9.8e-01
Particle concentration (mol/l)	21°C,24h	9.2e-01	7.7e-01	9.7e-01
Particle concentration (mol/l)	21°C,48h	9.0e-01	7.6e-01	9.6e-01
Total lipids (mmol/l)	4°C,24h	9.5e-01	8.2e-01	9.8e-01
Total lipids (mmol/l)	4°C,48h	9.1e-01	7.4e-01	9.7e-01
Total lipids (mmol/l)	21°C,24h	9.1e-01	7.6e-01	9.8e-01
Total lipids (mmol/l)	21°C,48h	8.7e-01	7.1e-01	9.5e-01
Phospholipids (mmol/l)	4°C,24h	9.4e-01	8.5e-01	9.8e-01
Phospholipids (mmol/l)	4°C,48h	9.3e-01	7.9e-01	9.7e-01
Phospholipids (mmol/l)	21°C,24h	9.1e-01	7.5e-01	9.7e-01
Phospholipids (mmol/l)	21°C,48h	8.8e-01	7.3e-01	9.5e-01
Total cholesterol (mmol/l)	4°C,24h	9.7e-01	8.9e-01	9.9e-01
Total cholesterol (mmol/l)	4°C,48h	9.3e-01	7.9e-01	9.8e-01
Total cholesterol (mmol/l)	21°C,24h	9.1e-01	7.3e-01	9.7e-01
Total cholesterol (mmol/l)	21°C,48h	8.8e-01	6.9e-01	9.5e-01
Cholesterol esters (mmol/l)	4°C,24h	9.4e-01	7.9e-01	9.8e-01
Cholesterol esters (mmol/l)	4°C,48h	9.3e-01	7.9e-01	9.8e-01
Cholesterol esters (mmol/l)	21°C,24h	8.8e-01	7.1e-01	9.6e-01
Cholesterol esters (mmol/l)	21°C,48h	9.0e-01	7.0e-01	9.6e-01
Free cholesterol (mmol/l)	4°C,24h	9.5e-01	8.3e-01	9.9e-01
Free cholesterol (mmol/l)	4°C,48h	9.0e-01	7.1e-01	9.8e-01

Metabolic traits	conditions	correlation	LCI	UCI
Free cholesterol (mmol/l)	21°C,24h	8.2e-01	5.5e-01	9.6e-01
Free cholesterol (mmol/l)	21°C,48h	7.7e-01	4.6e-01	9.3e-01
Triglycerides (mmol/l)	4°C,24h	9.9e-01	9.6e-01	1.0e+00
Triglycerides (mmol/l)	4°C,48h	9.9e-01	9.4e-01	1.0e+00
Triglycerides (mmol/l)	21°C,24h	9.6e-01	8.8e-01	9.9e-01
Triglycerides (mmol/l)	21°C,48h	9.6e-01	8.6e-01	9.8e-01
Medium LDL				
Particle concentration (mol/l)	4°C,24h	9.3e-01	7.9e-01	9.8e-01
Particle concentration (mol/l)	4°C,48h	9.2e-01	7.6e-01	9.7e-01
Particle concentration (mol/l)	21°C,24h	8.8e-01	6.6e-01	9.6e-01
Particle concentration (mol/l)	21°C,48h	8.9e-01	7.0e-01	9.6e-01
Total lipids (mmol/l)	4°C,24h	9.4e-01	8.1e-01	9.9e-01
Total lipids (mmol/l)	4°C,48h	9.3e-01	7.8e-01	9.8e-01
Total lipids (mmol/l)	21°C,24h	8.7e-01	6.5e-01	9.5e-01
Total lipids (mmol/l)	21°C,48h	9.1e-01	7.3e-01	9.7e-01
Phospholipids (mmol/l)	4°C,24h	9.7e-01	8.7e-01	9.8e-01
Phospholipids (mmol/l)	4°C,48h	9.5e-01	8.3e-01	9.8e-01
Phospholipids (mmol/l)	21°C,24h	9.4e-01	8.2e-01	9.9e-01
Phospholipids (mmol/l)	21°C,48h	9.1e-01	7.5e-01	9.7e-01
Total cholesterol (mmol/l)	4°C,24h	9.5e-01	8.2e-01	9.9e-01
Total cholesterol (mmol/l)	4°C,48h	9.2e-01	7.6e-01	9.8e-01
Total cholesterol (mmol/l)	21°C,24h	8.5e-01	5.9e-01	9.3e-01
Total cholesterol (mmol/l)	21°C,48h	8.7e-01	6.7e-01	9.3e-01
Cholesterol esters (mmol/l)	4°C,24h	9.6e-01	8.6e-01	9.9e-01
Cholesterol esters (mmol/l)	4°C,48h	9.3e-01	7.8e-01	9.8e-01
Cholesterol esters (mmol/l)	21°C,24h	8.6e-01	6.2e-01	9.4e-01
Cholesterol esters (mmol/l)	21°C,48h	8.7e-01	6.9e-01	9.4e-01
Free cholesterol (mmol/l)	4°C,24h	9.4e-01	8.3e-01	9.8e-01
Free cholesterol (mmol/l)	4°C,48h	9.1e-01	7.6e-01	9.8e-01

Metabolic traits	conditions	correlation	LCI	UCI
Free cholesterol (mmol/l)	21°C,24h	8.7e-01	6.7e-01	9.4e-01
Free cholesterol (mmol/l)	21°C,48h	8.8e-01	6.7e-01	9.5e-01
Triglycerides (mmol/l)	4°C,24h	9.8e-01	9.3e-01	1.0e+00
Triglycerides (mmol/l)	4°C,48h	9.8e-01	9.3e-01	1.0e+00
Triglycerides (mmol/l)	21°C,24h	9.5e-01	8.4e-01	9.8e-01
Triglycerides (mmol/l)	21°C,48h	9.4e-01	7.9e-01	9.8e-01
Small LDL				
Particle concentration (mol/l)	4°C,24h	9.5e-01	8.3e-01	9.9e-01
Particle concentration (mol/l)	4°C,48h	9.3e-01	7.7e-01	9.8e-01
Particle concentration (mol/l)	21°C,24h	9.1e-01	7.4e-01	9.8e-01
Particle concentration (mol/l)	21°C,48h	9.2e-01	7.4e-01	9.8e-01
Total lipids (mmol/l)	4°C,24h	9.5e-01	8.0e-01	9.9e-01
Total lipids (mmol/l)	4°C,48h	9.2e-01	7.8e-01	9.8e-01
Total lipids (mmol/l)	21°C,24h	8.9e-01	6.9e-01	9.6e-01
Total lipids (mmol/l)	21°C,48h	9.3e-01	7.7e-01	9.8e-01
Phospholipids (mmol/l)	4°C,24h	9.9e-01	9.5e-01	1.0e+00
Phospholipids (mmol/l)	4°C,48h	9.7e-01	8.7e-01	9.9e-01
Phospholipids (mmol/l)	21°C,24h	9.6e-01	8.4e-01	9.9e-01
Phospholipids (mmol/l)	21°C,48h	9.4e-01	8.3e-01	9.8e-01
Total cholesterol (mmol/l)	4°C,24h	9.4e-01	8.3e-01	9.9e-01
Total cholesterol (mmol/l)	4°C,48h	9.1e-01	7.6e-01	9.8e-01
Total cholesterol (mmol/l)	21°C,24h	8.4e-01	5.9e-01	9.4e-01
Total cholesterol (mmol/l)	21°C,48h	8.2e-01	5.4e-01	9.3e-01
Cholesterol esters (mmol/l)	4°C,24h	9.6e-01	8.1e-01	9.9e-01
Cholesterol esters (mmol/l)	4°C,48h	9.0e-01	7.2e-01	9.6e-01
Cholesterol esters (mmol/l)	21°C,24h	8.3e-01	5.5e-01	9.5e-01
Cholesterol esters (mmol/l)	21°C,48h	7.9e-01	4.4e-01	9.2e-01
Free cholesterol (mmol/l)	4°C,24h	9.5e-01	8.0e-01	9.9e-01
Free cholesterol (mmol/l)	4°C,48h	9.2e-01	7.7e-01	9.7e-01

Metabolic traits	conditions	correlation	LCI	UCI
Free cholesterol (mmol/l)	21°C,24h	8.8e-01	7.1e-01	9.5e-01
Free cholesterol (mmol/l)	21°C,48h	9.1e-01	7.3e-01	9.6e-01
Triglycerides (mmol/l)	4°C,24h	9.9e-01	9.6e-01	1.0e+00
Triglycerides (mmol/l)	4°C,48h	9.9e-01	9.4e-01	1.0e+00
Triglycerides (mmol/l)	21°C,24h	9.9e-01	9.4e-01	1.0e+00
Triglycerides (mmol/l)	21°C,48h	9.7e-01	8.4e-01	1.0e+00
Very large HDL				
Particle concentration (mol/l)	4°C,24h	9.8e-01	9.2e-01	1.0e+00
Particle concentration (mol/l)	4°C,48h	9.8e-01	9.3e-01	1.0e+00
Particle concentration (mol/l)	21°C,24h	9.8e-01	9.1e-01	1.0e+00
Particle concentration (mol/l)	21°C,48h	9.0e-01	7.3e-01	9.5e-01
Total lipids (mmol/l)	4°C,24h	9.8e-01	8.9e-01	1.0e+00
Total lipids (mmol/l)	4°C,48h	9.8e-01	9.1e-01	1.0e+00
Total lipids (mmol/l)	21°C,24h	9.8e-01	9.1e-01	1.0e+00
Total lipids (mmol/l)	21°C,48h	9.0e-01	7.4e-01	9.5e-01
Phospholipids (mmol/l)	4°C,24h	9.9e-01	9.5e-01	1.0e+00
Phospholipids (mmol/l)	4°C,48h	9.9e-01	9.5e-01	1.0e+00
Phospholipids (mmol/l)	21°C,24h	9.8e-01	9.0e-01	9.9e-01
Phospholipids (mmol/l)	21°C,48h	9.6e-01	8.6e-01	9.8e-01
Total cholesterol (mmol/l)	4°C,24h	9.7e-01	8.8e-01	1.0e+00
Total cholesterol (mmol/l)	4°C,48h	9.8e-01	9.1e-01	1.0e+00
Total cholesterol (mmol/l)	21°C,24h	9.8e-01	9.0e-01	1.0e+00
Total cholesterol (mmol/l)	21°C,48h	8.5e-01	5.9e-01	9.5e-01
Cholesterol esters (mmol/l)	4°C,24h	9.7e-01	8.9e-01	1.0e+00
Cholesterol esters (mmol/l)	4°C,48h	9.8e-01	9.0e-01	1.0e+00
Cholesterol esters (mmol/l)	21°C,24h	9.8e-01	9.2e-01	9.9e-01
Cholesterol esters (mmol/l)	21°C,48h	8.4e-01	6.1e-01	9.5e-01
Free cholesterol (mmol/l)	4°C,24h	9.8e-01	9.2e-01	1.0e+00
Free cholesterol (mmol/l)	4°C,48h	9.8e-01	9.2e-01	1.0e+00

Metabolic traits	conditions	correlation	LCI	UCI
Free cholesterol (mmol/l)	21°C,24h	9.7e-01	9.0e-01	9.9e-01
Free cholesterol (mmol/l)	21°C,48h	8.9e-01	7.1e-01	9.5e-01
Triglycerides (mmol/l)	4°C,24h	9.9e-01	9.4e-01	1.0e+00
Triglycerides (mmol/l)	4°C,48h	9.8e-01	9.1e-01	9.9e-01
Triglycerides (mmol/l)	21°C,24h	9.8e-01	8.9e-01	1.0e+00
Triglycerides (mmol/l)	21°C,48h	9.1e-01	7.3e-01	9.7e-01
Large HDL				
Particle concentration (mol/l)	4°C,24h	9.8e-01	9.0e-01	1.0e+00
Particle concentration (mol/l)	4°C,48h	9.8e-01	9.0e-01	1.0e+00
Particle concentration (mol/l)	21°C,24h	9.5e-01	8.2e-01	9.9e-01
Particle concentration (mol/l)	21°C,48h	9.4e-01	8.3e-01	9.9e-01
Total lipids (mmol/l)	4°C,24h	9.8e-01	9.4e-01	1.0e+00
Total lipids (mmol/l)	4°C,48h	9.9e-01	9.4e-01	1.0e+00
Total lipids (mmol/l)	21°C,24h	9.5e-01	8.3e-01	1.0e+00
Total lipids (mmol/l)	21°C,48h	9.4e-01	8.3e-01	9.8e-01
Phospholipids (mmol/l)	4°C,24h	9.8e-01	9.1e-01	1.0e+00
Phospholipids (mmol/l)	4°C,48h	9.9e-01	9.3e-01	1.0e+00
Phospholipids (mmol/l)	21°C,24h	9.4e-01	8.1e-01	9.9e-01
Phospholipids (mmol/l)	21°C,48h	9.1e-01	7.6e-01	9.7e-01
Total cholesterol (mmol/l)	4°C,24h	9.9e-01	9.3e-01	1.0e+00
Total cholesterol (mmol/l)	4°C,48h	1.0e+00	9.7e-01	1.0e+00
Total cholesterol (mmol/l)	21°C,24h	9.6e-01	8.3e-01	9.9e-01
Total cholesterol (mmol/l)	21°C,48h	9.5e-01	8.5e-01	9.8e-01
Cholesterol esters (mmol/l)	4°C,24h	9.9e-01	9.4e-01	1.0e+00
Cholesterol esters (mmol/l)	4°C,48h	9.9e-01	9.6e-01	1.0e+00
Cholesterol esters (mmol/l)	21°C,24h	9.6e-01	8.6e-01	1.0e+00
Cholesterol esters (mmol/l)	21°C,48h	9.5e-01	8.6e-01	9.8e-01
Free cholesterol (mmol/l)	4°C,24h	9.9e-01	9.4e-01	1.0e+00
Free cholesterol (mmol/l)	4°C,48h	9.9e-01	9.6e-01	1.0e+00

Metabolic traits	conditions	correlation	LCI	UCI
Free cholesterol (mmol/l)	21°C,24h	9.6e-01	8.4e-01	9.9e-01
Free cholesterol (mmol/l)	21°C,48h	9.5e-01	8.4e-01	9.8e-01
Triglycerides (mmol/l)	4°C,24h	8.9e-01	6.3e-01	1.0e+00
Triglycerides (mmol/l)	4°C,48h	8.8e-01	6.2e-01	9.9e-01
Triglycerides (mmol/l)	21°C,24h	8.5e-01	5.5e-01	9.8e-01
Triglycerides (mmol/l)	21°C,48h	7.9e-01	4.8e-01	9.4e-01
Medium HDL				
Particle concentration (mol/l)	4°C,24h	9.8e-01	9.2e-01	1.0e+00
Particle concentration (mol/l)	4°C,48h	9.7e-01	8.9e-01	1.0e+00
Particle concentration (mol/l)	21°C,24h	9.4e-01	8.3e-01	9.8e-01
Particle concentration (mol/l)	21°C,48h	8.0e-01	5.4e-01	9.1e-01
Total lipids (mmol/l)	4°C,24h	9.9e-01	9.4e-01	1.0e+00
Total lipids (mmol/l)	4°C,48h	9.7e-01	8.8e-01	9.9e-01
Total lipids (mmol/l)	21°C,24h	9.5e-01	8.4e-01	9.8e-01
Total lipids (mmol/l)	21°C,48h	8.0e-01	5.7e-01	9.2e-01
Phospholipids (mmol/l)	4°C,24h	9.9e-01	9.5e-01	1.0e+00
Phospholipids (mmol/l)	4°C,48h	9.7e-01	8.8e-01	1.0e+00
Phospholipids (mmol/l)	21°C,24h	9.5e-01	8.6e-01	9.8e-01
Phospholipids (mmol/l)	21°C,48h	8.1e-01	5.8e-01	9.2e-01
Total cholesterol (mmol/l)	4°C,24h	9.9e-01	9.3e-01	1.0e+00
Total cholesterol (mmol/l)	4°C,48h	9.8e-01	9.3e-01	1.0e+00
Total cholesterol (mmol/l)	21°C,24h	9.7e-01	8.7e-01	9.9e-01
Total cholesterol (mmol/l)	21°C,48h	7.6e-01	4.4e-01	9.4e-01
Cholesterol esters (mmol/l)	4°C,24h	9.8e-01	9.2e-01	9.9e-01
Cholesterol esters (mmol/l)	4°C,48h	9.9e-01	9.3e-01	1.0e+00
Cholesterol esters (mmol/l)	21°C,24h	9.6e-01	8.5e-01	9.9e-01
Cholesterol esters (mmol/l)	21°C,48h	7.8e-01	4.9e-01	9.4e-01
Free cholesterol (mmol/l)	4°C,24h	9.9e-01	9.5e-01	1.0e+00
Free cholesterol (mmol/l)	4°C,48h	9.7e-01	8.9e-01	1.0e+00

Metabolic traits	conditions	correlation	LCI	UCI
Free cholesterol (mmol/l)	21°C,24h	9.7e-01	8.9e-01	9.9e-01
Free cholesterol (mmol/l)	21°C,48h	7.6e-01	4.2e-01	9.3e-01
Triglycerides (mmol/l)	4°C,24h	9.8e-01	9.2e-01	1.0e+00
Triglycerides (mmol/l)	4°C,48h	9.7e-01	8.6e-01	9.9e-01
Triglycerides (mmol/l)	21°C,24h	9.7e-01	8.8e-01	9.9e-01
Triglycerides (mmol/l)	21°C,48h	9.3e-01	7.6e-01	9.9e-01
Small HDL				
Particle concentration (mol/l)	4°C,24h	9.5e-01	8.2e-01	9.9e-01
Particle concentration (mol/l)	4°C,48h	9.5e-01	8.0e-01	1.0e+00
Particle concentration (mol/l)	21°C,24h	9.5e-01	8.3e-01	9.9e-01
Particle concentration (mol/l)	21°C,48h	6.5e-01	2.4e-01	8.7e-01
Total lipids (mmol/l)	4°C,24h	9.6e-01	8.5e-01	9.9e-01
Total lipids (mmol/l)	4°C,48h	9.5e-01	8.4e-01	9.9e-01
Total lipids (mmol/l)	21°C,24h	9.3e-01	8.1e-01	9.7e-01
Total lipids (mmol/l)	21°C,48h	7.0e-01	3.3e-01	9.0e-01
Phospholipids (mmol/l)	4°C,24h	9.7e-01	8.6e-01	1.0e+00
Phospholipids (mmol/l)	4°C,48h	9.6e-01	8.8e-01	9.9e-01
Phospholipids (mmol/l)	21°C,24h	9.2e-01	7.7e-01	9.7e-01
Phospholipids (mmol/l)	21°C,48h	6.1e-01	2.1e-01	8.4e-01
Total cholesterol (mmol/l)	4°C,24h	8.9e-01	6.5e-01	9.8e-01
Total cholesterol (mmol/l)	4°C,48h	8.4e-01	5.3e-01	9.8e-01
Total cholesterol (mmol/l)	21°C,24h	8.5e-01	5.9e-01	9.7e-01
Total cholesterol (mmol/l)	21°C,48h	6.2e-01	2.6e-01	8.7e-01
Cholesterol esters (mmol/l)	4°C,24h	9.0e-01	7.1e-01	9.8e-01
Cholesterol esters (mmol/l)	4°C,48h	9.0e-01	6.7e-01	9.8e-01
Cholesterol esters (mmol/l)	21°C,24h	8.3e-01	5.5e-01	9.6e-01
Cholesterol esters (mmol/l)	21°C,48h	6.7e-01	2.7e-01	8.9e-01
Free cholesterol (mmol/l)	4°C,24h	9.5e-01	8.1e-01	9.9e-01
Free cholesterol (mmol/l)	4°C,48h	9.8e-01	8.9e-01	9.9e-01

Metabolic traits	conditions	correlation	LCI	UCI
Free cholesterol (mmol/l)	21°C,24h	9.1e-01	7.5e-01	9.7e-01
Free cholesterol (mmol/l)	21°C,48h	6.0e-01	2.3e-01	8.6e-01
Triglycerides (mmol/l)	4°C,24h	9.9e-01	9.7e-01	1.0e+00
Triglycerides (mmol/l)	4°C,48h	9.8e-01	9.0e-01	1.0e+00
Triglycerides (mmol/l)	21°C,24h	9.8e-01	9.3e-01	1.0e+00
Triglycerides (mmol/l)	21°C,48h	9.7e-01	8.9e-01	9.9e-01
**Lipoprotein particle size**				
VLDL particle size (nm)	4°C,24h	9.8e-01	9.2e-01	1.0e+00
VLDL particle size (nm)	4°C,48h	9.7e-01	8.9e-01	9.9e-01
VLDL particle size (nm)	21°C,24h	9.5e-01	8.2e-01	9.9e-01
VLDL particle size (nm)	21°C,48h	9.1e-01	7.4e-01	9.8e-01
LDL particle size (nm)	4°C,24h	9.5e-01	8.6e-01	9.8e-01
LDL particle size (nm)	4°C,48h	9.4e-01	8.4e-01	9.8e-01
LDL particle size (nm)	21°C,24h	9.4e-01	8.3e-01	9.7e-01
LDL particle size (nm)	21°C,48h	7.0e-01	4.1e-01	8.6e-01
HDL particle size (nm)	4°C,24h	9.9e-01	9.5e-01	1.0e+00
HDL particle size (nm)	4°C,48h	9.9e-01	9.5e-01	1.0e+00
HDL particle size (nm)	21°C,24h	9.9e-01	9.5e-01	1.0e+00
HDL particle size (nm)	21°C,48h	9.7e-01	8.8e-01	9.9e-01
**Cholesterol**				
Total cholesterol (mmol/l)	4°C,24h	9.7e-01	8.6e-01	9.9e-01
Total cholesterol (mmol/l)	4°C,48h	9.5e-01	8.6e-01	9.9e-01
Total cholesterol (mmol/l)	21°C,24h	9.3e-01	7.9e-01	9.8e-01
Total cholesterol (mmol/l)	21°C,48h	9.3e-01	7.9e-01	9.7e-01
VLDL cholesterol (mmol/l)	4°C,24h	9.8e-01	9.1e-01	1.0e+00
VLDL cholesterol (mmol/l)	4°C,48h	9.8e-01	8.9e-01	1.0e+00
VLDL cholesterol (mmol/l)	21°C,24h	9.7e-01	8.8e-01	1.0e+00
VLDL cholesterol (mmol/l)	21°C,48h	8.9e-01	6.5e-01	9.7e-01
Remnant cholesterol (mmol/l)	4°C,24h	9.8e-01	9.0e-01	1.0e+00

Metabolic traits	conditions	correlation	LCI	UCI
Remnant cholesterol (mmol/l)	4°C,48h	9.7e-01	8.7e-01	1.0e+00
Remnant cholesterol (mmol/l)	21°C,24h	9.7e-01	8.8e-01	1.0e+00
Remnant cholesterol (mmol/l)	21°C,48h	9.2e-01	7.8e-01	9.8e-01
LDL cholesterol (mmol/l)	4°C,24h	9.6e-01	8.6e-01	9.9e-01
LDL cholesterol (mmol/l)	4°C,48h	9.3e-01	7.7e-01	9.8e-01
LDL cholesterol (mmol/l)	21°C,24h	8.6e-01	6.3e-01	9.5e-01
LDL cholesterol (mmol/l)	21°C,48h	9.0e-01	7.4e-01	9.6e-01
HDL cholesterol (mmol/l)	4°C,24h	9.9e-01	9.1e-01	1.0e+00
HDL cholesterol (mmol/l)	4°C,48h	9.8e-01	9.2e-01	1.0e+00
HDL cholesterol (mmol/l)	21°C,24h	9.5e-01	8.1e-01	9.9e-01
HDL cholesterol (mmol/l)	21°C,48h	8.7e-01	6.9e-01	9.6e-01
HDL2 cholesterol (mmol/l)	4°C,24h	9.9e-01	9.2e-01	1.0e+00
HDL2 cholesterol (mmol/l)	4°C,48h	9.8e-01	9.0e-01	9.9e-01
HDL2 cholesterol (mmol/l)	21°C,24h	9.5e-01	8.2e-01	9.9e-01
HDL2 cholesterol (mmol/l)	21°C,48h	9.0e-01	7.2e-01	9.7e-01
HDL3 cholesterol (mmol/l)	4°C,24h	9.8e-01	9.4e-01	1.0e+00
HDL3 cholesterol (mmol/l)	4°C,48h	9.5e-01	8.6e-01	9.8e-01
HDL3 cholesterol (mmol/l)	21°C,24h	8.7e-01	6.4e-01	9.5e-01
HDL3 cholesterol (mmol/l)	21°C,48h	4.7e-01	-4.5e-03	8.1e-01
Esterified cholesterol (mmol/l)	4°C,24h	9.5e-01	8.3e-01	9.9e-01
Esterified cholesterol (mmol/l)	4°C,48h	9.2e-01	7.2e-01	9.7e-01
Esterified cholesterol (mmol/l)	21°C,24h	9.1e-01	7.5e-01	9.8e-01
Esterified cholesterol (mmol/l)	21°C,48h	8.6e-01	6.8e-01	9.4e-01
Free cholesterol (mmol/l)	4°C,24h	9.2e-01	6.9e-01	9.9e-01
Free cholesterol (mmol/l)	4°C,48h	9.8e-01	9.1e-01	1.0e+00
Free cholesterol (mmol/l)	21°C,24h	9.5e-01	8.0e-01	1.0e+00
Free cholesterol (mmol/l)	21°C,48h	9.6e-01	8.6e-01	9.9e-01
**Glycerides and phospholipids**				
Triglycerides (mmol/l)	4°C,24h	9.9e-01	9.5e-01	1.0e+00

Metabolic traits	conditions	correlation	LCI	UCI
Triglycerides (mmol/l)	4°C,48h	9.9e-01	9.5e-01	1.0e+00
Triglycerides (mmol/l)	21°C,24h	9.8e-01	9.0e-01	1.0e+00
Triglycerides (mmol/l)	21°C,48h	9.2e-01	7.7e-01	9.9e-01
VLDL triglycerides (mmol/l)	4°C,24h	9.9e-01	9.6e-01	1.0e+00
VLDL triglycerides (mmol/l)	4°C,48h	9.8e-01	9.0e-01	1.0e+00
VLDL triglycerides (mmol/l)	21°C,24h	9.6e-01	8.3e-01	1.0e+00
VLDL triglycerides (mmol/l)	21°C,48h	8.8e-01	6.4e-01	9.7e-01
LDL triglycerides (mmol/l)	4°C,24h	1.0e+00	9.7e-01	1.0e+00
LDL triglycerides (mmol/l)	4°C,48h	9.9e-01	9.4e-01	1.0e+00
LDL triglycerides (mmol/l)	21°C,24h	9.7e-01	9.0e-01	9.9e-01
LDL triglycerides (mmol/l)	21°C,48h	9.5e-01	8.2e-01	9.8e-01
HDL triglycerides (mmol/l)	4°C,24h	1.0e+00	9.6e-01	1.0e+00
HDL triglycerides (mmol/l)	4°C,48h	1.0e+00	9.7e-01	1.0e+00
HDL triglycerides (mmol/l)	21°C,24h	9.9e-01	9.3e-01	1.0e+00
HDL triglycerides (mmol/l)	21°C,48h	9.8e-01	9.0e-01	1.0e+00
Diacylglycerol (mmol/l)	4°C,24h	3.2e-01	-2.2e-01	7.4e-01
Diacylglycerol (mmol/l)	4°C,48h	1.9e-01	-3.7e-01	6.3e-01
Diacylglycerol (mmol/l)	21°C,24h	2.8e-01	-3.3e-01	7.1e-01
Diacylglycerol (mmol/l)	21°C,48h	5.3e-01	1.3e-02	8.2e-01
Phosphoglycerides (mmol/l)	4°C,24h	9.5e-01	8.5e-01	9.9e-01
Phosphoglycerides (mmol/l)	4°C,48h	9.5e-01	8.1e-01	9.9e-01
Phosphoglycerides (mmol/l)	21°C,24h	9.1e-01	7.6e-01	9.8e-01
Phosphoglycerides (mmol/l)	21°C,48h	8.9e-01	6.8e-01	9.5e-01
Phosphatidylcholine + other cholines (mmol/l)	4°C,24h	9.5e-01	8.1e-01	9.9e-01
Phosphatidylcholine + other cholines (mmol/l)	4°C,48h	9.5e-01	8.1e-01	9.9e-01
Phosphatidylcholine + other cholines (mmol/l)	21°C,24h	9.4e-01	8.1e-01	9.8e-01
Phosphatidylcholine + other cholines (mmol/l)	21°C,48h	8.9e-01	6.8e-01	9.7e-01
Sphingomyelins (mmol/l)	4°C,24h	6.9e-01	3.0e-01	8.9e-01
Sphingomyelins (mmol/l)	4°C,48h	6.9e-01	4.3e-01	8.5e-01

Metabolic traits	conditions	correlation	LCI	UCI
Sphingomyelins (mmol/l)	21°C,24h	6.6e-01	2.1e-01	8.7e-01
Sphingomyelins (mmol/l)	21°C,48h	6.5e-01	1.8e-01	8.7e-01
Cholines (mmol/l)	4°C,24h	8.5e-01	6.2e-01	9.6e-01
Cholines (mmol/l)	4°C,48h	8.3e-01	5.8e-01	9.5e-01
Cholines (mmol/l)	21°C,24h	7.8e-01	4.8e-01	9.3e-01
Cholines (mmol/l)	21°C,48h	6.7e-01	2.0e-01	9.1e-01
**Apolipoproteins**				
Apolipoprotein A-I (g/l)	4°C,24h	9.8e-01	9.2e-01	1.0e+00
Apolipoprotein A-I (g/l)	4°C,48h	9.8e-01	9.3e-01	9.9e-01
Apolipoprotein A-I (g/l)	21°C,24h	9.7e-01	8.7e-01	1.0e+00
Apolipoprotein A-I (g/l)	21°C,48h	8.5e-01	6.8e-01	9.5e-01
Apolipoprotein B (g/l)	4°C,24h	9.9e-01	9.4e-01	1.0e+00
Apolipoprotein B (g/l)	4°C,48h	9.8e-01	9.0e-01	1.0e+00
Apolipoprotein B (g/l)	21°C,24h	9.7e-01	8.9e-01	9.9e-01
Apolipoprotein B (g/l)	21°C,48h	9.5e-01	8.2e-01	9.9e-01
**Fatty acids**				
Total fatty acids (mmol/l)	4°C,24h	9.5e-01	8.0e-01	9.9e-01
Total fatty acids (mmol/l)	4°C,48h	9.7e-01	8.5e-01	1.0e+00
Total fatty acids (mmol/l)	21°C,24h	9.6e-01	8.7e-01	1.0e+00
Total fatty acids (mmol/l)	21°C,48h	9.6e-01	8.7e-01	1.0e+00
Fatty acid chain length	4°C,24h	7.6e-01	4.0e-01	9.4e-01
Fatty acid chain length	4°C,48h	7.9e-01	4.8e-01	9.6e-01
Fatty acid chain length	21°C,24h	8.8e-01	6.7e-01	9.8e-01
Fatty acid chain length	21°C,48h	8.5e-01	5.5e-01	9.5e-01
Degree of unsaturation	4°C,24h	9.2e-01	7.3e-01	9.9e-01
Degree of unsaturation	4°C,48h	9.5e-01	8.1e-01	9.9e-01
Degree of unsaturation	21°C,24h	9.0e-01	6.8e-01	9.7e-01
Degree of unsaturation	21°C,48h	9.1e-01	7.2e-01	9.7e-01
Docosahexaenoic acid (mmol/l)	4°C,24h	9.1e-01	6.6e-01	1.0e+00

Metabolic traits	conditions	correlation	LCI	UCI
Docosahexaenoic acid (mmol/l)	4°C,48h	9.1e-01	6.9e-01	9.9e-01
Docosahexaenoic acid (mmol/l)	21°C,24h	9.5e-01	8.1e-01	9.9e-01
Docosahexaenoic acid (mmol/l)	21°C,48h	9.4e-01	8.1e-01	9.8e-01
Linoleic acid (mmol/l)	4°C,24h	9.7e-01	8.8e-01	1.0e+00
Linoleic acid (mmol/l)	4°C,48h	9.7e-01	8.7e-01	9.9e-01
Linoleic acid (mmol/l)	21°C,24h	9.7e-01	8.7e-01	1.0e+00
Linoleic acid (mmol/l)	21°C,48h	9.5e-01	8.4e-01	9.8e-01
Conjugated linoleic acid (mmol/l)	4°C,24h	5.4e-01	-4.6e-03	8.6e-01
Conjugated linoleic acid (mmol/l)	4°C,48h	5.5e-01	1.5e-03	8.5e-01
Conjugated linoleic acid (mmol/l)	21°C,24h	7.1e-01	2.8e-01	9.3e-01
Conjugated linoleic acid (mmol/l)	21°C,48h	6.3e-01	1.4e-01	9.0e-01
n-3 fatty acids (mmol/l)	4°C,24h	8.8e-01	5.7e-01	9.9e-01
n-3 fatty acids (mmol/l)	4°C,48h	9.1e-01	6.7e-01	9.8e-01
n-3 fatty acids (mmol/l)	21°C,24h	9.7e-01	8.7e-01	1.0e+00
n-3 fatty acids (mmol/l)	21°C,48h	9.5e-01	8.3e-01	9.9e-01
n-6 fatty acids (mmol/l)	4°C,24h	9.4e-01	7.7e-01	9.9e-01
n-6 fatty acids (mmol/l)	4°C,48h	9.5e-01	8.5e-01	9.9e-01
n-6 fatty acids (mmol/l)	21°C,24h	9.6e-01	8.5e-01	9.9e-01
n-6 fatty acids (mmol/l)	21°C,48h	9.5e-01	8.5e-01	9.8e-01
PUFA (mmol/l)	4°C,24h	9.5e-01	7.9e-01	1.0e+00
PUFA (mmol/l)	4°C,48h	9.7e-01	8.9e-01	9.9e-01
PUFA (mmol/l)	21°C,24h	9.8e-01	8.9e-01	1.0e+00
PUFA (mmol/l)	21°C,48h	9.6e-01	8.6e-01	9.8e-01
MUFA (mmol/l)	4°C,24h	9.8e-01	8.9e-01	1.0e+00
MUFA (mmol/l)	4°C,48h	9.9e-01	9.3e-01	1.0e+00
MUFA (mmol/l)	21°C,24h	9.7e-01	8.6e-01	1.0e+00
MUFA (mmol/l)	21°C,48h	9.7e-01	8.5e-01	1.0e+00
Saturated fatty acids (mmol/l)	4°C,24h	9.7e-01	8.2e-01	1.0e+00
Saturated fatty acids (mmol/l)	4°C,48h	9.8e-01	9.2e-01	1.0e+00

Metabolic traits	conditions	correlation	LCI	UCI
Saturated fatty acids (mmol/l)	21°C,24h	9.5e-01	8.2e-01	1.0e+00
Saturated fatty acids (mmol/l)	21°C,48h	9.7e-01	8.7e-01	9.9e-01
**Glycolysis related metabolites**				
Glucose (mmol/l)	4°C,24h	9.7e-01	9.1e-01	9.9e-01
Glucose (mmol/l)	4°C,48h	9.1e-01	7.3e-01	9.7e-01
Glucose (mmol/l)	21°C,24h	7.0e-01	3.5e-01	9.3e-01
Glucose (mmol/l)	21°C,48h	4.8e-01	1.0e-01	7.4e-01
Lactate (mmol/l)	4°C,24h	7.3e-01	3.7e-01	9.0e-01
Lactate (mmol/l)	4°C,48h	5.5e-01	1.9e-01	8.3e-01
Lactate (mmol/l)	21°C,24h	3.3e-01	-1.3e-01	6.6e-01
Lactate (mmol/l)	21°C,48h	5.2e-01	1.0e-01	7.7e-01
Pyruvate (mmol/l)	4°C,24h	4.4e-01	1.4e-02	7.7e-01
Pyruvate (mmol/l)	4°C,48h	2.8e-01	-9.7e-02	6.2e-01
Pyruvate (mmol/l)	21°C,24h	-6.5e-02	-5.5e-01	4.5e-01
Pyruvate (mmol/l)	21°C,48h	-6.6e-02	-5.7e-01	4.1e-01
Citrate (mmol/l)	4°C,24h	8.6e-01	6.3e-01	9.5e-01
Citrate (mmol/l)	4°C,48h	8.2e-01	5.3e-01	9.5e-01
Citrate (mmol/l)	21°C,24h	8.0e-01	4.7e-01	9.5e-01
Citrate (mmol/l)	21°C,48h	8.7e-01	6.3e-01	9.8e-01
Glycerol (mmol/l)	4°C,24h	9.0e-01	7.2e-01	9.6e-01
Glycerol (mmol/l)	4°C,48h	4.9e-01	5.1e-02	8.0e-01
Glycerol (mmol/l)	21°C,24h	6.7e-01	3.1e-01	8.6e-01
Glycerol (mmol/l)	21°C,48h	5.1e-01	8.3e-02	8.1e-01
**Amino acids**				
Alanine (mmol/l)	4°C,24h	9.5e-01	8.3e-01	9.8e-01
Alanine (mmol/l)	4°C,48h	9.2e-01	7.9e-01	9.6e-01
Alanine (mmol/l)	21°C,24h	8.6e-01	6.1e-01	9.6e-01
Alanine (mmol/l)	21°C,48h	5.6e-01	8.3e-02	8.5e-01
Glutamine (mmol/l)	4°C,24h	9.6e-01	8.6e-01	9.9e-01

Metabolic traits	conditions	correlation	LCI	UCI
Glutamine (mmol/l)	4°C,48h	9.4e-01	7.9e-01	9.9e-01
Glutamine (mmol/l)	21°C,24h	9.2e-01	7.5e-01	9.7e-01
Glutamine (mmol/l)	21°C,48h	8.1e-01	4.4e-01	9.5e-01
Histidine (mmol/l)	4°C,24h	3.9e-01	-1.2e-01	7.5e-01
Histidine (mmol/l)	4°C,48h	5.6e-01	1.3e-01	8.4e-01
Histidine (mmol/l)	21°C,24h	5.0e-01	7.4e-03	8.3e-01
Histidine (mmol/l)	21°C,48h	4.8e-01	8.2e-02	8.0e-01
Glycine (mmol/l)	4°C,24h	8.9e-01	6.9e-01	9.8e-01
Glycine (mmol/l)	4°C,48h	8.0e-01	4.0e-01	9.5e-01
Glycine (mmol/l)	21°C,24h	8.9e-01	6.8e-01	9.8e-01
Glycine (mmol/l)	21°C,48h	7.7e-01	4.8e-01	9.3e-01
Branched-chain amino acids				
Isoleucine (mmol/l)	4°C,24h	9.8e-01	9.0e-01	9.9e-01
Isoleucine (mmol/l)	4°C,48h	9.8e-01	9.0e-01	1.0e+00
Isoleucine (mmol/l)	21°C,24h	9.2e-01	7.7e-01	9.9e-01
Isoleucine (mmol/l)	21°C,48h	9.4e-01	7.8e-01	1.0e+00
Leucine (mmol/l)	4°C,24h	9.8e-01	9.1e-01	1.0e+00
Leucine (mmol/l)	4°C,48h	9.7e-01	9.0e-01	1.0e+00
Leucine (mmol/l)	21°C,24h	9.1e-01	7.4e-01	9.8e-01
Leucine (mmol/l)	21°C,48h	9.2e-01	7.8e-01	9.8e-01
Valine (mmol/l)	4°C,24h	9.8e-01	9.2e-01	1.0e+00
Valine (mmol/l)	4°C,48h	9.8e-01	9.2e-01	1.0e+00
Valine (mmol/l)	21°C,24h	9.6e-01	8.6e-01	9.9e-01
Valine (mmol/l)	21°C,48h	9.5e-01	8.4e-01	9.9e-01
Aromatic amino acids				
Phenylalanine (mmol/l)	4°C,24h	7.4e-01	4.5e-01	9.1e-01
Phenylalanine (mmol/l)	4°C,48h	7.3e-01	4.9e-01	8.6e-01
Phenylalanine (mmol/l)	21°C,24h	7.7e-01	5.1e-01	9.0e-01
Phenylalanine (mmol/l)	21°C,48h	6.5e-01	3.4e-01	8.3e-01

Metabolic traits	conditions	correlation	LCI	UCI
Tyrosine (mmol/l)	4°C,24h	8.9e-01	6.6e-01	9.8e-01
Tyrosine (mmol/l)	4°C,48h	8.9e-01	6.6e-01	9.8e-01
Tyrosine (mmol/l)	21°C,24h	8.9e-01	6.4e-01	9.7e-01
Tyrosine (mmol/l)	21°C,48h	8.6e-01	6.3e-01	9.7e-01
**Ketone bodies**				
Acetate (mmol/l)	4°C,24h	8.3e-01	5.5e-01	9.6e-01
Acetate (mmol/l)	4°C,48h	7.0e-01	3.9e-01	8.7e-01
Acetate (mmol/l)	21°C,24h	4.5e-01	4.2e-02	7.9e-01
Acetate (mmol/l)	21°C,48h	6.0e-01	2.5e-01	8.2e-01
Beta-hydroxybutyrate (mmol/l)	4°C,24h	8.7e-01	5.6e-01	9.7e-01
Beta-hydroxybutyrate (mmol/l)	4°C,48h	7.3e-01	3.4e-01	9.5e-01
Beta-hydroxybutyrate (mmol/l)	21°C,24h	8.6e-01	6.4e-01	9.6e-01
Beta-hydroxybutyrate (mmol/l)	21°C,48h	7.3e-01	3.8e-01	9.0e-01
**Fluid balance**				
Creatinine (mmol/l)	4°C,24h	9.3e-01	8.1e-01	9.7e-01
Creatinine (mmol/l)	4°C,48h	9.6e-01	8.8e-01	9.9e-01
Creatinine (mmol/l)	21°C,24h	9.4e-01	8.3e-01	9.8e-01
Creatinine (mmol/l)	21°C,48h	9.5e-01	8.5e-01	9.7e-01
Albumin (signal area)	4°C,24h	9.6e-01	8.5e-01	9.8e-01
Albumin (signal area)	4°C,48h	9.1e-01	7.3e-01	9.8e-01
Albumin (signal area)	21°C,24h	8.5e-01	5.3e-01	9.6e-01
Albumin (signal area)	21°C,48h	9.0e-01	6.9e-01	9.8e-01
**Inflammation**				
Glycoprotein acetyls (mmol/l)	4°C,24h	9.9e-01	9.4e-01	1.0e+00
Glycoprotein acetyls (mmol/l)	4°C,48h	9.9e-01	9.5e-01	1.0e+00
Glycoprotein acetyls (mmol/l)	21°C,24h	9.9e-01	9.4e-01	1.0e+00
Glycoprotein acetyls (mmol/l)	21°C,48h	9.7e-01	8.9e-01	9.9e-01

*Table S3. Spearman's correlation: EDTA-plasma, pre-storage handling effects. Spearman's rank correlation coefficients between metabolic concentrations (or values) in reference samples (condition: 4 °C, 1.5 h) and samples incubated at (i) 4 °C, 24 h; (ii) 4 °C, 48 h; (iii) 21 °C, 24 h; (iv) 21 °C, 48 h, before centrifugation (see Figures 3-4, S2). Pyruvate, glycerol and glycine are not quantified in EDTA-plasma samples due to the interfering resonances of EDTA on their signals. **Abbreviations**: **C**=cholesterol; **IDL**=intermediate-density lipoprotein; **LCI**=lower confidence interval; **LDL**=low-density lipoprotein; **HDL**=high-density lipoprotein; **MUFA**=monounsaturated fatty acids; **PUFA**=polyunsaturated fatty acids; **UCI**= upper confidence interval; **VLDL**=very-low-density lipoprotein.*

Metabolic traits	conditions	correlation	LCI	UCI
**Lipoprotein subclasses**				
Extremely large VLDL				
Particle concentration (mol/l)	4°C,24h	8.5e-01	5.7e-01	9.6e-01
Particle concentration (mol/l)	4°C,48h	9.5e-01	8.4e-01	1.0e+00
Particle concentration (mol/l)	21°C,24h	9.7e-01	8.9e-01	9.9e-01
Particle concentration (mol/l)	21°C,48h	7.3e-01	3.3e-01	9.4e-01
Total lipids (mmol/l)	4°C,24h	8.5e-01	5.5e-01	9.7e-01
Total lipids (mmol/l)	4°C,48h	9.5e-01	8.2e-01	9.9e-01
Total lipids (mmol/l)	21°C,24h	9.7e-01	9.0e-01	1.0e+00
Total lipids (mmol/l)	21°C,48h	7.3e-01	3.4e-01	9.4e-01
Phospholipids (mmol/l)	4°C,24h	8.9e-01	6.7e-01	9.7e-01
Phospholipids (mmol/l)	4°C,48h	9.7e-01	8.9e-01	1.0e+00
Phospholipids (mmol/l)	21°C,24h	9.8e-01	9.4e-01	1.0e+00
Phospholipids (mmol/l)	21°C,48h	7.9e-01	4.6e-01	9.5e-01
Total cholesterol (mmol/l)	4°C,24h	8.5e-01	5.3e-01	9.7e-01
Total cholesterol (mmol/l)	4°C,48h	9.5e-01	7.9e-01	9.9e-01
Total cholesterol (mmol/l)	21°C,24h	9.7e-01	8.9e-01	9.9e-01
Total cholesterol (mmol/l)	21°C,48h	7.5e-01	3.9e-01	9.4e-01
Cholesterol esters (mmol/l)	4°C,24h	8.3e-01	5.4e-01	9.7e-01
Cholesterol esters (mmol/l)	4°C,48h	9.4e-01	7.7e-01	9.9e-01
Cholesterol esters (mmol/l)	21°C,24h	9.5e-01	8.0e-01	9.9e-01
Cholesterol esters (mmol/l)	21°C,48h	7.4e-01	3.6e-01	9.3e-01
Free cholesterol (mmol/l)	4°C,24h	9.0e-01	6.8e-01	9.8e-01

Metabolic traits	conditions	correlation	LCI	UCI
Free cholesterol (mmol/l)	4°C,48h	9.7e-01	8.9e-01	1.0e+00
Free cholesterol (mmol/l)	21°C,24h	9.8e-01	9.3e-01	1.0e+00
Free cholesterol (mmol/l)	21°C,48h	8.0e-01	5.2e-01	9.5e-01
Triglycerides (mmol/l)	4°C,24h	8.5e-01	5.4e-01	9.6e-01
Triglycerides (mmol/l)	4°C,48h	9.6e-01	8.6e-01	1.0e+00
Triglycerides (mmol/l)	21°C,24h	9.7e-01	8.9e-01	9.9e-01
Triglycerides (mmol/l)	21°C,48h	7.2e-01	3.5e-01	9.2e-01
Very large VLDL				
Particle concentration (mol/l)	4°C,24h	9.3e-01	7.6e-01	9.9e-01
Particle concentration (mol/l)	4°C,48h	9.6e-01	8.7e-01	1.0e+00
Particle concentration (mol/l)	21°C,24h	9.9e-01	9.6e-01	1.0e+00
Particle concentration (mol/l)	21°C,48h	8.6e-01	6.4e-01	9.7e-01
Total lipids (mmol/l)	4°C,24h	9.3e-01	7.6e-01	9.8e-01
Total lipids (mmol/l)	4°C,48h	9.6e-01	8.7e-01	1.0e+00
Total lipids (mmol/l)	21°C,24h	9.9e-01	9.6e-01	1.0e+00
Total lipids (mmol/l)	21°C,48h	8.7e-01	6.2e-01	9.7e-01
Phospholipids (mmol/l)	4°C,24h	9.2e-01	7.2e-01	9.8e-01
Phospholipids (mmol/l)	4°C,48h	9.6e-01	8.8e-01	9.9e-01
Phospholipids (mmol/l)	21°C,24h	9.9e-01	9.6e-01	1.0e+00
Phospholipids (mmol/l)	21°C,48h	8.6e-01	5.7e-01	9.6e-01
Total cholesterol (mmol/l)	4°C,24h	9.0e-01	6.9e-01	9.9e-01
Total cholesterol (mmol/l)	4°C,48h	9.5e-01	8.1e-01	9.9e-01
Total cholesterol (mmol/l)	21°C,24h	9.9e-01	9.5e-01	1.0e+00
Total cholesterol (mmol/l)	21°C,48h	8.1e-01	5.2e-01	9.6e-01
Cholesterol esters (mmol/l)	4°C,24h	8.8e-01	6.7e-01	9.8e-01
Cholesterol esters (mmol/l)	4°C,48h	9.5e-01	8.6e-01	9.9e-01
Cholesterol esters (mmol/l)	21°C,24h	9.9e-01	9.2e-01	1.0e+00
Cholesterol esters (mmol/l)	21°C,48h	8.0e-01	5.1e-01	9.6e-01
Free cholesterol (mmol/l)	4°C,24h	9.2e-01	7.6e-01	9.8e-01

Metabolic traits	conditions	correlation	LCI	UCI
Free cholesterol (mmol/l)	4°C,48h	9.5e-01	8.4e-01	9.9e-01
Free cholesterol (mmol/l)	21°C,24h	9.9e-01	9.6e-01	1.0e+00
Free cholesterol (mmol/l)	21°C,48h	8.6e-01	6.6e-01	9.6e-01
Triglycerides (mmol/l)	4°C,24h	9.3e-01	7.6e-01	9.9e-01
Triglycerides (mmol/l)	4°C,48h	9.7e-01	8.8e-01	1.0e+00
Triglycerides (mmol/l)	21°C,24h	9.9e-01	9.5e-01	1.0e+00
Triglycerides (mmol/l)	21°C,48h	8.7e-01	6.2e-01	9.7e-01
Large VLDL				
Particle concentration (mol/l)	4°C,24h	9.7e-01	8.9e-01	1.0e+00
Particle concentration (mol/l)	4°C,48h	9.7e-01	8.8e-01	1.0e+00
Particle concentration (mol/l)	21°C,24h	9.6e-01	8.5e-01	1.0e+00
Particle concentration (mol/l)	21°C,48h	8.6e-01	6.2e-01	9.6e-01
Total lipids (mmol/l)	4°C,24h	9.6e-01	8.5e-01	9.9e-01
Total lipids (mmol/l)	4°C,48h	9.7e-01	9.1e-01	1.0e+00
Total lipids (mmol/l)	21°C,24h	9.7e-01	8.8e-01	1.0e+00
Total lipids (mmol/l)	21°C,48h	8.7e-01	6.5e-01	9.6e-01
Phospholipids (mmol/l)	4°C,24h	9.5e-01	8.2e-01	9.9e-01
Phospholipids (mmol/l)	4°C,48h	9.8e-01	9.3e-01	1.0e+00
Phospholipids (mmol/l)	21°C,24h	9.6e-01	8.3e-01	1.0e+00
Phospholipids (mmol/l)	21°C,48h	8.8e-01	6.7e-01	9.7e-01
Total cholesterol (mmol/l)	4°C,24h	9.5e-01	8.4e-01	9.9e-01
Total cholesterol (mmol/l)	4°C,48h	9.7e-01	9.0e-01	9.9e-01
Total cholesterol (mmol/l)	21°C,24h	9.8e-01	9.1e-01	1.0e+00
Total cholesterol (mmol/l)	21°C,48h	8.8e-01	6.4e-01	9.6e-01
Cholesterol esters (mmol/l)	4°C,24h	9.2e-01	7.0e-01	1.0e+00
Cholesterol esters (mmol/l)	4°C,48h	9.5e-01	8.2e-01	9.9e-01
Cholesterol esters (mmol/l)	21°C,24h	9.7e-01	9.1e-01	9.9e-01
Cholesterol esters (mmol/l)	21°C,48h	8.5e-01	5.9e-01	9.6e-01
Free cholesterol (mmol/l)	4°C,24h	9.5e-01	8.2e-01	9.9e-01

Metabolic traits	conditions	correlation	LCI	UCI
Free cholesterol (mmol/l)	4°C,48h	9.7e-01	8.8e-01	9.9e-01
Free cholesterol (mmol/l)	21°C,24h	9.9e-01	9.5e-01	1.0e+00
Free cholesterol (mmol/l)	21°C,48h	8.8e-01	6.9e-01	9.8e-01
Triglycerides (mmol/l)	4°C,24h	9.8e-01	9.1e-01	1.0e+00
Triglycerides (mmol/l)	4°C,48h	9.7e-01	8.3e-01	1.0e+00
Triglycerides (mmol/l)	21°C,24h	9.4e-01	7.9e-01	9.9e-01
Triglycerides (mmol/l)	21°C,48h	8.6e-01	6.2e-01	9.6e-01
Medium VLDL				
Particle concentration (mol/l)	4°C,24h	9.2e-01	6.8e-01	9.9e-01
Particle concentration (mol/l)	4°C,48h	9.8e-01	8.9e-01	1.0e+00
Particle concentration (mol/l)	21°C,24h	9.4e-01	8.0e-01	9.9e-01
Particle concentration (mol/l)	21°C,48h	8.4e-01	5.2e-01	9.7e-01
Total lipids (mmol/l)	4°C,24h	9.3e-01	7.7e-01	9.9e-01
Total lipids (mmol/l)	4°C,48h	9.9e-01	9.5e-01	1.0e+00
Total lipids (mmol/l)	21°C,24h	9.4e-01	7.9e-01	9.9e-01
Total lipids (mmol/l)	21°C,48h	8.3e-01	5.2e-01	9.7e-01
Phospholipids (mmol/l)	4°C,24h	9.2e-01	7.5e-01	9.9e-01
Phospholipids (mmol/l)	4°C,48h	9.9e-01	9.6e-01	1.0e+00
Phospholipids (mmol/l)	21°C,24h	9.3e-01	7.7e-01	9.9e-01
Phospholipids (mmol/l)	21°C,48h	8.3e-01	4.8e-01	9.6e-01
Total cholesterol (mmol/l)	4°C,24h	9.1e-01	6.9e-01	9.9e-01
Total cholesterol (mmol/l)	4°C,48h	9.5e-01	8.3e-01	1.0e+00
Total cholesterol (mmol/l)	21°C,24h	9.7e-01	8.6e-01	1.0e+00
Total cholesterol (mmol/l)	21°C,48h	8.8e-01	6.4e-01	9.7e-01
Cholesterol esters (mmol/l)	4°C,24h	9.3e-01	7.4e-01	9.9e-01
Cholesterol esters (mmol/l)	4°C,48h	9.4e-01	7.9e-01	1.0e+00
Cholesterol esters (mmol/l)	21°C,24h	9.5e-01	7.9e-01	9.8e-01
Cholesterol esters (mmol/l)	21°C,48h	8.6e-01	6.2e-01	9.7e-01
Free cholesterol (mmol/l)	4°C,24h	9.6e-01	8.4e-01	1.0e+00

Metabolic traits	conditions	correlation	LCI	UCI
Free cholesterol (mmol/l)	4°C,48h	9.7e-01	8.8e-01	1.0e+00
Free cholesterol (mmol/l)	21°C,24h	9.6e-01	8.6e-01	1.0e+00
Free cholesterol (mmol/l)	21°C,48h	8.7e-01	6.0e-01	9.8e-01
Triglycerides (mmol/l)	4°C,24h	9.6e-01	8.5e-01	9.9e-01
Triglycerides (mmol/l)	4°C,48h	9.9e-01	9.4e-01	1.0e+00
Triglycerides (mmol/l)	21°C,24h	9.6e-01	8.6e-01	1.0e+00
Triglycerides (mmol/l)	21°C,48h	9.0e-01	6.6e-01	9.8e-01
Small VLDL				
Particle concentration (mol/l)	4°C,24h	9.5e-01	8.2e-01	1.0e+00
Particle concentration (mol/l)	4°C,48h	9.8e-01	9.0e-01	1.0e+00
Particle concentration (mol/l)	21°C,24h	9.8e-01	8.9e-01	1.0e+00
Particle concentration (mol/l)	21°C,48h	9.1e-01	7.0e-01	9.9e-01
Total lipids (mmol/l)	4°C,24h	9.4e-01	7.6e-01	1.0e+00
Total lipids (mmol/l)	4°C,48h	9.8e-01	8.9e-01	1.0e+00
Total lipids (mmol/l)	21°C,24h	9.7e-01	8.7e-01	1.0e+00
Total lipids (mmol/l)	21°C,48h	9.1e-01	7.1e-01	9.9e-01
Phospholipids (mmol/l)	4°C,24h	9.6e-01	8.3e-01	9.9e-01
Phospholipids (mmol/l)	4°C,48h	9.8e-01	9.0e-01	1.0e+00
Phospholipids (mmol/l)	21°C,24h	9.6e-01	8.7e-01	9.9e-01
Phospholipids (mmol/l)	21°C,48h	9.2e-01	7.3e-01	9.8e-01
Total cholesterol (mmol/l)	4°C,24h	9.4e-01	7.9e-01	9.9e-01
Total cholesterol (mmol/l)	4°C,48h	9.8e-01	9.2e-01	1.0e+00
Total cholesterol (mmol/l)	21°C,24h	9.8e-01	9.0e-01	1.0e+00
Total cholesterol (mmol/l)	21°C,48h	9.7e-01	8.9e-01	9.9e-01
Cholesterol esters (mmol/l)	4°C,24h	9.4e-01	7.5e-01	9.9e-01
Cholesterol esters (mmol/l)	4°C,48h	9.6e-01	8.2e-01	1.0e+00
Cholesterol esters (mmol/l)	21°C,24h	9.7e-01	8.8e-01	1.0e+00
Cholesterol esters (mmol/l)	21°C,48h	9.5e-01	8.3e-01	9.8e-01
Free cholesterol (mmol/l)	4°C,24h	9.8e-01	9.1e-01	1.0e+00

Metabolic traits	conditions	correlation	LCI	UCI
Free cholesterol (mmol/l)	4°C,48h	9.8e-01	9.1e-01	1.0e+00
Free cholesterol (mmol/l)	21°C,24h	9.8e-01	9.1e-01	1.0e+00
Free cholesterol (mmol/l)	21°C,48h	9.5e-01	8.0e-01	9.9e-01
Triglycerides (mmol/l)	4°C,24h	9.6e-01	8.3e-01	9.9e-01
Triglycerides (mmol/l)	4°C,48h	9.9e-01	9.5e-01	1.0e+00
Triglycerides (mmol/l)	21°C,24h	9.7e-01	8.9e-01	1.0e+00
Triglycerides (mmol/l)	21°C,48h	8.9e-01	6.6e-01	9.8e-01
Very Small VLDL				
Particle concentration (mol/l)	4°C,24h	9.6e-01	8.4e-01	9.9e-01
Particle concentration (mol/l)	4°C,48h	9.7e-01	9.0e-01	9.9e-01
Particle concentration (mol/l)	21°C,24h	9.7e-01	9.0e-01	9.9e-01
Particle concentration (mol/l)	21°C,48h	9.3e-01	8.1e-01	9.8e-01
Total lipids (mmol/l)	4°C,24h	9.6e-01	8.3e-01	1.0e+00
Total lipids (mmol/l)	4°C,48h	9.8e-01	9.0e-01	1.0e+00
Total lipids (mmol/l)	21°C,24h	9.7e-01	8.6e-01	9.9e-01
Total lipids (mmol/l)	21°C,48h	9.4e-01	8.0e-01	9.8e-01
Phospholipids (mmol/l)	4°C,24h	9.2e-01	7.3e-01	9.8e-01
Phospholipids (mmol/l)	4°C,48h	9.8e-01	9.1e-01	1.0e+00
Phospholipids (mmol/l)	21°C,24h	9.5e-01	8.5e-01	9.9e-01
Phospholipids (mmol/l)	21°C,48h	9.4e-01	8.2e-01	9.9e-01
Total cholesterol (mmol/l)	4°C,24h	8.8e-01	6.3e-01	9.8e-01
Total cholesterol (mmol/l)	4°C,48h	9.5e-01	8.3e-01	9.9e-01
Total cholesterol (mmol/l)	21°C,24h	9.1e-01	7.0e-01	9.8e-01
Total cholesterol (mmol/l)	21°C,48h	8.7e-01	6.7e-01	9.6e-01
Cholesterol esters (mmol/l)	4°C,24h	8.3e-01	5.2e-01	9.5e-01
Cholesterol esters (mmol/l)	4°C,48h	9.3e-01	7.9e-01	9.9e-01
Cholesterol esters (mmol/l)	21°C,24h	8.8e-01	6.8e-01	9.7e-01
Cholesterol esters (mmol/l)	21°C,48h	8.1e-01	5.5e-01	9.5e-01
Free cholesterol (mmol/l)	4°C,24h	9.2e-01	7.0e-01	9.9e-01

Metabolic traits	conditions	correlation	LCI	UCI
Free cholesterol (mmol/l)	4°C,48h	9.9e-01	9.4e-01	1.0e+00
Free cholesterol (mmol/l)	21°C,24h	9.8e-01	9.2e-01	9.9e-01
Free cholesterol (mmol/l)	21°C,48h	9.7e-01	9.0e-01	9.9e-01
Triglycerides (mmol/l)	4°C,24h	9.5e-01	7.9e-01	9.9e-01
Triglycerides (mmol/l)	4°C,48h	9.6e-01	8.0e-01	1.0e+00
Triglycerides (mmol/l)	21°C,24h	9.4e-01	7.8e-01	9.9e-01
Triglycerides (mmol/l)	21°C,48h	9.3e-01	7.4e-01	9.9e-01
IDL				
Particle concentration (mol/l)	4°C,24h	9.5e-01	8.0e-01	9.9e-01
Particle concentration (mol/l)	4°C,48h	9.9e-01	9.5e-01	1.0e+00
Particle concentration (mol/l)	21°C,24h	9.6e-01	8.0e-01	9.9e-01
Particle concentration (mol/l)	21°C,48h	9.4e-01	8.0e-01	9.8e-01
Total lipids (mmol/l)	4°C,24h	9.4e-01	8.2e-01	9.9e-01
Total lipids (mmol/l)	4°C,48h	9.8e-01	9.2e-01	1.0e+00
Total lipids (mmol/l)	21°C,24h	9.5e-01	8.1e-01	9.8e-01
Total lipids (mmol/l)	21°C,48h	9.3e-01	8.2e-01	9.8e-01
Phospholipids (mmol/l)	4°C,24h	9.4e-01	7.8e-01	9.9e-01
Phospholipids (mmol/l)	4°C,48h	9.7e-01	8.7e-01	1.0e+00
Phospholipids (mmol/l)	21°C,24h	9.2e-01	7.5e-01	9.8e-01
Phospholipids (mmol/l)	21°C,48h	8.8e-01	6.8e-01	9.7e-01
Total cholesterol (mmol/l)	4°C,24h	9.0e-01	7.2e-01	9.9e-01
Total cholesterol (mmol/l)	4°C,48h	9.5e-01	8.4e-01	9.9e-01
Total cholesterol (mmol/l)	21°C,24h	9.1e-01	7.5e-01	9.7e-01
Total cholesterol (mmol/l)	21°C,48h	9.0e-01	6.9e-01	9.7e-01
Cholesterol esters (mmol/l)	4°C,24h	9.0e-01	7.4e-01	9.7e-01
Cholesterol esters (mmol/l)	4°C,48h	9.4e-01	8.0e-01	9.9e-01
Cholesterol esters (mmol/l)	21°C,24h	9.0e-01	7.1e-01	9.7e-01
Cholesterol esters (mmol/l)	21°C,48h	8.9e-01	6.8e-01	9.7e-01
Free cholesterol (mmol/l)	4°C,24h	9.3e-01	7.7e-01	9.8e-01

Metabolic traits	conditions	correlation	LCI	UCI
Free cholesterol (mmol/l)	4°C,48h	9.5e-01	8.2e-01	9.9e-01
Free cholesterol (mmol/l)	21°C,24h	8.9e-01	7.3e-01	9.6e-01
Free cholesterol (mmol/l)	21°C,48h	8.5e-01	6.1e-01	9.5e-01
Triglycerides (mmol/l)	4°C,24h	9.7e-01	8.8e-01	9.9e-01
Triglycerides (mmol/l)	4°C,48h	9.8e-01	8.7e-01	1.0e+00
Triglycerides (mmol/l)	21°C,24h	9.6e-01	8.5e-01	1.0e+00
Triglycerides (mmol/l)	21°C,48h	9.5e-01	8.5e-01	9.9e-01
Large LDL				
Particle concentration (mol/l)	4°C,24h	9.6e-01	8.6e-01	1.0e+00
Particle concentration (mol/l)	4°C,48h	9.7e-01	8.9e-01	9.9e-01
Particle concentration (mol/l)	21°C,24h	9.4e-01	7.9e-01	9.8e-01
Particle concentration (mol/l)	21°C,48h	9.3e-01	7.5e-01	9.9e-01
Total lipids (mmol/l)	4°C,24h	9.5e-01	8.4e-01	1.0e+00
Total lipids (mmol/l)	4°C,48h	9.6e-01	8.8e-01	9.9e-01
Total lipids (mmol/l)	21°C,24h	9.3e-01	7.6e-01	9.8e-01
Total lipids (mmol/l)	21°C,48h	9.1e-01	7.2e-01	9.8e-01
Phospholipids (mmol/l)	4°C,24h	9.4e-01	7.8e-01	9.9e-01
Phospholipids (mmol/l)	4°C,48h	9.6e-01	8.6e-01	9.9e-01
Phospholipids (mmol/l)	21°C,24h	9.4e-01	7.9e-01	9.9e-01
Phospholipids (mmol/l)	21°C,48h	9.3e-01	7.8e-01	9.9e-01
Total cholesterol (mmol/l)	4°C,24h	9.4e-01	7.8e-01	9.9e-01
Total cholesterol (mmol/l)	4°C,48h	9.6e-01	8.3e-01	9.9e-01
Total cholesterol (mmol/l)	21°C,24h	9.4e-01	8.1e-01	9.9e-01
Total cholesterol (mmol/l)	21°C,48h	9.2e-01	7.1e-01	9.8e-01
Cholesterol esters (mmol/l)	4°C,24h	9.4e-01	8.0e-01	9.9e-01
Cholesterol esters (mmol/l)	4°C,48h	9.6e-01	8.5e-01	9.9e-01
Cholesterol esters (mmol/l)	21°C,24h	9.5e-01	7.7e-01	9.9e-01
Cholesterol esters (mmol/l)	21°C,48h	9.2e-01	7.3e-01	9.8e-01
Free cholesterol (mmol/l)	4°C,24h	9.3e-01	8.0e-01	9.8e-01

Metabolic traits	conditions	correlation	LCI	UCI
Free cholesterol (mmol/l)	4°C,48h	9.5e-01	8.6e-01	9.8e-01
Free cholesterol (mmol/l)	21°C,24h	9.0e-01	7.1e-01	9.6e-01
Free cholesterol (mmol/l)	21°C,48h	8.0e-01	5.0e-01	9.6e-01
Triglycerides (mmol/l)	4°C,24h	9.8e-01	8.9e-01	9.9e-01
Triglycerides (mmol/l)	4°C,48h	9.8e-01	9.0e-01	1.0e+00
Triglycerides (mmol/l)	21°C,24h	9.7e-01	8.7e-01	9.9e-01
Triglycerides (mmol/l)	21°C,48h	9.7e-01	8.9e-01	9.9e-01
Medium LDL				
Particle concentration (mol/l)	4°C,24h	9.6e-01	8.7e-01	9.9e-01
Particle concentration (mol/l)	4°C,48h	9.7e-01	8.9e-01	1.0e+00
Particle concentration (mol/l)	21°C,24h	9.6e-01	8.5e-01	9.9e-01
Particle concentration (mol/l)	21°C,48h	9.3e-01	7.8e-01	9.8e-01
Total lipids (mmol/l)	4°C,24h	9.6e-01	8.5e-01	9.9e-01
Total lipids (mmol/l)	4°C,48h	9.6e-01	8.2e-01	9.9e-01
Total lipids (mmol/l)	21°C,24h	9.5e-01	8.4e-01	9.9e-01
Total lipids (mmol/l)	21°C,48h	9.1e-01	7.0e-01	9.8e-01
Phospholipids (mmol/l)	4°C,24h	9.4e-01	8.2e-01	9.9e-01
Phospholipids (mmol/l)	4°C,48h	9.7e-01	8.8e-01	1.0e+00
Phospholipids (mmol/l)	21°C,24h	9.7e-01	8.8e-01	9.9e-01
Phospholipids (mmol/l)	21°C,48h	9.5e-01	8.2e-01	9.9e-01
Total cholesterol (mmol/l)	4°C,24h	9.5e-01	8.4e-01	9.9e-01
Total cholesterol (mmol/l)	4°C,48h	9.7e-01	8.7e-01	1.0e+00
Total cholesterol (mmol/l)	21°C,24h	9.3e-01	7.6e-01	9.8e-01
Total cholesterol (mmol/l)	21°C,48h	9.1e-01	7.1e-01	9.9e-01
Cholesterol esters (mmol/l)	4°C,24h	9.6e-01	8.6e-01	9.9e-01
Cholesterol esters (mmol/l)	4°C,48h	9.7e-01	9.0e-01	1.0e+00
Cholesterol esters (mmol/l)	21°C,24h	9.4e-01	7.8e-01	9.9e-01
Cholesterol esters (mmol/l)	21°C,48h	9.1e-01	7.0e-01	9.8e-01
Free cholesterol (mmol/l)	4°C,24h	9.5e-01	8.3e-01	9.9e-01

Metabolic traits	conditions	correlation	LCI	UCI
Free cholesterol (mmol/l)	4°C,48h	9.7e-01	8.9e-01	1.0e+00
Free cholesterol (mmol/l)	21°C,24h	9.4e-01	8.0e-01	9.8e-01
Free cholesterol (mmol/l)	21°C,48h	8.8e-01	6.6e-01	9.7e-01
Triglycerides (mmol/l)	4°C,24h	9.4e-01	8.2e-01	9.8e-01
Triglycerides (mmol/l)	4°C,48h	9.8e-01	9.1e-01	1.0e+00
Triglycerides (mmol/l)	21°C,24h	9.6e-01	8.8e-01	9.9e-01
Triglycerides (mmol/l)	21°C,48h	9.4e-01	8.5e-01	9.8e-01
Small LDL				
Particle concentration (mol/l)	4°C,24h	9.6e-01	8.6e-01	9.9e-01
Particle concentration (mol/l)	4°C,48h	9.8e-01	9.0e-01	1.0e+00
Particle concentration (mol/l)	21°C,24h	9.5e-01	8.4e-01	9.9e-01
Particle concentration (mol/l)	21°C,48h	9.2e-01	7.2e-01	9.8e-01
Total lipids (mmol/l)	4°C,24h	9.6e-01	8.8e-01	9.9e-01
Total lipids (mmol/l)	4°C,48h	9.8e-01	9.1e-01	1.0e+00
Total lipids (mmol/l)	21°C,24h	9.5e-01	8.0e-01	9.9e-01
Total lipids (mmol/l)	21°C,48h	9.1e-01	6.9e-01	9.8e-01
Phospholipids (mmol/l)	4°C,24h	9.3e-01	7.8e-01	9.9e-01
Phospholipids (mmol/l)	4°C,48h	9.7e-01	8.8e-01	1.0e+00
Phospholipids (mmol/l)	21°C,24h	9.6e-01	8.7e-01	9.9e-01
Phospholipids (mmol/l)	21°C,48h	9.1e-01	7.2e-01	9.8e-01
Total cholesterol (mmol/l)	4°C,24h	9.4e-01	8.5e-01	9.9e-01
Total cholesterol (mmol/l)	4°C,48h	9.8e-01	9.3e-01	1.0e+00
Total cholesterol (mmol/l)	21°C,24h	9.2e-01	7.5e-01	9.8e-01
Total cholesterol (mmol/l)	21°C,48h	8.9e-01	6.8e-01	9.8e-01
Cholesterol esters (mmol/l)	4°C,24h	9.7e-01	8.8e-01	9.9e-01
Cholesterol esters (mmol/l)	4°C,48h	9.9e-01	9.3e-01	1.0e+00
Cholesterol esters (mmol/l)	21°C,24h	9.5e-01	8.1e-01	9.9e-01
Cholesterol esters (mmol/l)	21°C,48h	8.9e-01	6.8e-01	9.7e-01
Free cholesterol (mmol/l)	4°C,24h	9.5e-01	8.2e-01	9.9e-01

Metabolic traits	conditions	correlation	LCI	UCI
Free cholesterol (mmol/l)	4°C,48h	9.8e-01	9.0e-01	9.9e-01
Free cholesterol (mmol/l)	21°C,24h	9.3e-01	8.1e-01	9.8e-01
Free cholesterol (mmol/l)	21°C,48h	8.3e-01	5.5e-01	9.5e-01
Triglycerides (mmol/l)	4°C,24h	9.7e-01	8.8e-01	9.9e-01
Triglycerides (mmol/l)	4°C,48h	9.9e-01	9.4e-01	1.0e+00
Triglycerides (mmol/l)	21°C,24h	9.7e-01	8.8e-01	1.0e+00
Triglycerides (mmol/l)	21°C,48h	9.6e-01	8.7e-01	9.9e-01
Very large HDL				
Particle concentration (mol/l)	4°C,24h	9.9e-01	9.5e-01	1.0e+00
Particle concentration (mol/l)	4°C,48h	9.9e-01	9.3e-01	1.0e+00
Particle concentration (mol/l)	21°C,24h	9.8e-01	9.2e-01	1.0e+00
Particle concentration (mol/l)	21°C,48h	9.8e-01	9.1e-01	9.9e-01
Total lipids (mmol/l)	4°C,24h	9.9e-01	9.6e-01	1.0e+00
Total lipids (mmol/l)	4°C,48h	9.8e-01	9.1e-01	1.0e+00
Total lipids (mmol/l)	21°C,24h	9.8e-01	9.1e-01	9.9e-01
Total lipids (mmol/l)	21°C,48h	9.7e-01	9.1e-01	9.9e-01
Phospholipids (mmol/l)	4°C,24h	1.0e+00	9.7e-01	1.0e+00
Phospholipids (mmol/l)	4°C,48h	9.9e-01	9.6e-01	1.0e+00
Phospholipids (mmol/l)	21°C,24h	9.9e-01	9.5e-01	1.0e+00
Phospholipids (mmol/l)	21°C,48h	9.9e-01	9.6e-01	1.0e+00
Total cholesterol (mmol/l)	4°C,24h	9.7e-01	8.8e-01	9.9e-01
Total cholesterol (mmol/l)	4°C,48h	9.6e-01	8.4e-01	9.9e-01
Total cholesterol (mmol/l)	21°C,24h	9.7e-01	9.0e-01	9.9e-01
Total cholesterol (mmol/l)	21°C,48h	9.6e-01	8.5e-01	9.9e-01
Cholesterol esters (mmol/l)	4°C,24h	9.6e-01	8.5e-01	9.9e-01
Cholesterol esters (mmol/l)	4°C,48h	9.7e-01	8.4e-01	9.9e-01
Cholesterol esters (mmol/l)	21°C,24h	9.6e-01	8.7e-01	9.9e-01
Cholesterol esters (mmol/l)	21°C,48h	9.7e-01	8.9e-01	1.0e+00
Free cholesterol (mmol/l)	4°C,24h	1.0e+00	9.7e-01	1.0e+00

Metabolic traits	conditions	correlation	LCI	UCI
Free cholesterol (mmol/l)	4°C,48h	9.8e-01	9.1e-01	1.0e+00
Free cholesterol (mmol/l)	21°C,24h	9.7e-01	8.9e-01	9.9e-01
Free cholesterol (mmol/l)	21°C,48h	9.7e-01	8.9e-01	9.9e-01
Triglycerides (mmol/l)	4°C,24h	9.2e-01	7.7e-01	9.7e-01
Triglycerides (mmol/l)	4°C,48h	9.5e-01	8.7e-01	9.8e-01
Triglycerides (mmol/l)	21°C,24h	8.8e-01	6.7e-01	9.6e-01
Triglycerides (mmol/l)	21°C,48h	8.9e-01	7.0e-01	9.6e-01
Large HDL				
Particle concentration (mol/l)	4°C,24h	9.6e-01	8.7e-01	9.9e-01
Particle concentration (mol/l)	4°C,48h	9.7e-01	8.8e-01	1.0e+00
Particle concentration (mol/l)	21°C,24h	9.8e-01	9.1e-01	9.9e-01
Particle concentration (mol/l)	21°C,48h	9.5e-01	8.0e-01	9.9e-01
Total lipids (mmol/l)	4°C,24h	9.6e-01	8.6e-01	9.9e-01
Total lipids (mmol/l)	4°C,48h	9.7e-01	8.7e-01	1.0e+00
Total lipids (mmol/l)	21°C,24h	9.8e-01	9.3e-01	9.9e-01
Total lipids (mmol/l)	21°C,48h	9.6e-01	7.8e-01	9.9e-01
Phospholipids (mmol/l)	4°C,24h	9.2e-01	7.8e-01	9.8e-01
Phospholipids (mmol/l)	4°C,48h	9.7e-01	8.7e-01	1.0e+00
Phospholipids (mmol/l)	21°C,24h	9.8e-01	9.2e-01	1.0e+00
Phospholipids (mmol/l)	21°C,48h	9.4e-01	8.3e-01	9.8e-01
Total cholesterol (mmol/l)	4°C,24h	9.8e-01	9.0e-01	1.0e+00
Total cholesterol (mmol/l)	4°C,48h	9.8e-01	9.1e-01	1.0e+00
Total cholesterol (mmol/l)	21°C,24h	9.8e-01	9.2e-01	1.0e+00
Total cholesterol (mmol/l)	21°C,48h	9.6e-01	8.7e-01	9.9e-01
Cholesterol esters (mmol/l)	4°C,24h	9.8e-01	8.8e-01	1.0e+00
Cholesterol esters (mmol/l)	4°C,48h	9.8e-01	8.8e-01	1.0e+00
Cholesterol esters (mmol/l)	21°C,24h	9.9e-01	9.5e-01	1.0e+00
Cholesterol esters (mmol/l)	21°C,48h	9.7e-01	8.6e-01	1.0e+00
Free cholesterol (mmol/l)	4°C,24h	9.6e-01	8.8e-01	9.9e-01

Metabolic traits	conditions	correlation	LCI	UCI
Free cholesterol (mmol/l)	4°C,48h	9.8e-01	8.8e-01	1.0e+00
Free cholesterol (mmol/l)	21°C,24h	9.9e-01	9.2e-01	1.0e+00
Free cholesterol (mmol/l)	21°C,48h	9.7e-01	8.6e-01	9.9e-01
Triglycerides (mmol/l)	4°C,24h	8.7e-01	5.6e-01	9.9e-01
Triglycerides (mmol/l)	4°C,48h	8.7e-01	5.6e-01	9.9e-01
Triglycerides (mmol/l)	21°C,24h	8.3e-01	5.6e-01	9.8e-01
Triglycerides (mmol/l)	21°C,48h	9.5e-01	8.5e-01	9.8e-01
Medium HDL				
Particle concentration (mol/l)	4°C,24h	8.1e-01	5.7e-01	9.4e-01
Particle concentration (mol/l)	4°C,48h	9.3e-01	7.7e-01	9.8e-01
Particle concentration (mol/l)	21°C,24h	9.1e-01	7.5e-01	9.7e-01
Particle concentration (mol/l)	21°C,48h	7.0e-01	3.0e-01	8.9e-01
Total lipids (mmol/l)	4°C,24h	8.1e-01	5.3e-01	9.4e-01
Total lipids (mmol/l)	4°C,48h	9.3e-01	7.5e-01	9.8e-01
Total lipids (mmol/l)	21°C,24h	9.1e-01	7.2e-01	9.8e-01
Total lipids (mmol/l)	21°C,48h	7.0e-01	2.7e-01	9.0e-01
Phospholipids (mmol/l)	4°C,24h	8.4e-01	6.0e-01	9.5e-01
Phospholipids (mmol/l)	4°C,48h	9.4e-01	8.1e-01	9.8e-01
Phospholipids (mmol/l)	21°C,24h	9.2e-01	7.8e-01	9.7e-01
Phospholipids (mmol/l)	21°C,48h	7.2e-01	3.8e-01	9.0e-01
Total cholesterol (mmol/l)	4°C,24h	8.0e-01	4.9e-01	9.5e-01
Total cholesterol (mmol/l)	4°C,48h	9.3e-01	8.0e-01	9.8e-01
Total cholesterol (mmol/l)	21°C,24h	9.0e-01	7.1e-01	9.7e-01
Total cholesterol (mmol/l)	21°C,48h	7.4e-01	3.7e-01	9.3e-01
Cholesterol esters (mmol/l)	4°C,24h	7.8e-01	4.9e-01	9.4e-01
Cholesterol esters (mmol/l)	4°C,48h	9.3e-01	7.8e-01	9.8e-01
Cholesterol esters (mmol/l)	21°C,24h	9.0e-01	7.0e-01	9.7e-01
Cholesterol esters (mmol/l)	21°C,48h	7.4e-01	3.6e-01	9.3e-01
Free cholesterol (mmol/l)	4°C,24h	8.3e-01	5.3e-01	9.5e-01

Metabolic traits	conditions	correlation	LCI	UCI
Free cholesterol (mmol/l)	4°C,48h	9.4e-01	7.9e-01	9.8e-01
Free cholesterol (mmol/l)	21°C,24h	8.9e-01	6.8e-01	9.7e-01
Free cholesterol (mmol/l)	21°C,48h	7.1e-01	3.4e-01	9.1e-01
Triglycerides (mmol/l)	4°C,24h	9.6e-01	8.2e-01	9.9e-01
Triglycerides (mmol/l)	4°C,48h	9.8e-01	9.0e-01	1.0e+00
Triglycerides (mmol/l)	21°C,24h	9.5e-01	8.5e-01	9.8e-01
Triglycerides (mmol/l)	21°C,48h	9.0e-01	7.1e-01	9.6e-01
Small HDL				
Particle concentration (mol/l)	4°C,24h	7.9e-01	4.6e-01	9.2e-01
Particle concentration (mol/l)	4°C,48h	8.8e-01	6.9e-01	9.8e-01
Particle concentration (mol/l)	21°C,24h	8.6e-01	6.8e-01	9.6e-01
Particle concentration (mol/l)	21°C,48h	4.9e-01	6.2e-02	7.8e-01
Total lipids (mmol/l)	4°C,24h	8.0e-01	5.4e-01	9.1e-01
Total lipids (mmol/l)	4°C,48h	8.7e-01	6.1e-01	9.7e-01
Total lipids (mmol/l)	21°C,24h	8.7e-01	6.8e-01	9.6e-01
Total lipids (mmol/l)	21°C,48h	4.7e-01	8.4e-02	7.7e-01
Phospholipids (mmol/l)	4°C,24h	8.6e-01	5.5e-01	9.8e-01
Phospholipids (mmol/l)	4°C,48h	9.7e-01	8.9e-01	9.9e-01
Phospholipids (mmol/l)	21°C,24h	9.6e-01	8.8e-01	9.9e-01
Phospholipids (mmol/l)	21°C,48h	6.1e-01	2.3e-01	8.3e-01
Total cholesterol (mmol/l)	4°C,24h	7.4e-01	4.3e-01	8.9e-01
Total cholesterol (mmol/l)	4°C,48h	8.8e-01	6.8e-01	9.6e-01
Total cholesterol (mmol/l)	21°C,24h	8.2e-01	5.2e-01	9.4e-01
Total cholesterol (mmol/l)	21°C,48h	4.8e-01	3.2e-02	7.9e-01
Cholesterol esters (mmol/l)	4°C,24h	7.1e-01	3.4e-01	9.0e-01
Cholesterol esters (mmol/l)	4°C,48h	9.0e-01	7.1e-01	9.7e-01
Cholesterol esters (mmol/l)	21°C,24h	7.9e-01	4.6e-01	9.5e-01
Cholesterol esters (mmol/l)	21°C,48h	5.8e-01	1.1e-01	8.2e-01
Free cholesterol (mmol/l)	4°C,24h	7.9e-01	4.1e-01	9.7e-01

Metabolic traits	conditions	correlation	LCI	UCI
Free cholesterol (mmol/l)	4°C,48h	9.7e-01	8.9e-01	9.9e-01
Free cholesterol (mmol/l)	21°C,24h	9.4e-01	8.6e-01	9.7e-01
Free cholesterol (mmol/l)	21°C,48h	5.7e-01	2.3e-01	7.9e-01
Triglycerides (mmol/l)	4°C,24h	9.5e-01	8.3e-01	9.9e-01
Triglycerides (mmol/l)	4°C,48h	9.7e-01	8.7e-01	1.0e+00
Triglycerides (mmol/l)	21°C,24h	9.8e-01	9.2e-01	1.0e+00
Triglycerides (mmol/l)	21°C,48h	9.8e-01	9.2e-01	1.0e+00
**Lipoprotein particle size**				
VLDL particle size (nm)	4°C,24h	9.7e-01	8.7e-01	1.0e+00
VLDL particle size (nm)	4°C,48h	9.9e-01	9.5e-01	1.0e+00
VLDL particle size (nm)	21°C,24h	9.8e-01	8.9e-01	1.0e+00
VLDL particle size (nm)	21°C,48h	8.7e-01	6.1e-01	9.7e-01
LDL particle size (nm)	4°C,24h	7.6e-01	4.3e-01	9.1e-01
LDL particle size (nm)	4°C,48h	8.4e-01	6.0e-01	9.6e-01
LDL particle size (nm)	21°C,24h	8.0e-01	5.8e-01	9.0e-01
LDL particle size (nm)	21°C,48h	3.4e-01	-1.8e-01	7.6e-01
HDL particle size (nm)	4°C,24h	9.8e-01	9.0e-01	9.9e-01
HDL particle size (nm)	4°C,48h	9.8e-01	9.4e-01	1.0e+00
HDL particle size (nm)	21°C,24h	9.9e-01	9.6e-01	1.0e+00
HDL particle size (nm)	21°C,48h	9.9e-01	9.4e-01	1.0e+00
**Cholesterol**				
Total cholesterol (mmol/l)	4°C,24h	9.6e-01	8.5e-01	9.9e-01
Total cholesterol (mmol/l)	4°C,48h	9.8e-01	9.4e-01	1.0e+00
Total cholesterol (mmol/l)	21°C,24h	9.8e-01	9.0e-01	1.0e+00
Total cholesterol (mmol/l)	21°C,48h	9.6e-01	8.8e-01	9.9e-01
VLDL cholesterol (mmol/l)	4°C,24h	9.1e-01	7.2e-01	9.8e-01
VLDL cholesterol (mmol/l)	4°C,48h	9.3e-01	7.6e-01	1.0e+00
VLDL cholesterol (mmol/l)	21°C,24h	9.6e-01	8.4e-01	9.9e-01
VLDL cholesterol (mmol/l)	21°C,48h	9.2e-01	7.5e-01	9.8e-01

Metabolic traits	conditions	correlation	LCI	UCI
Remnant cholesterol (mmol/l)	4°C,24h	9.3e-01	7.3e-01	9.8e-01
Remnant cholesterol (mmol/l)	4°C,48h	9.7e-01	8.9e-01	9.9e-01
Remnant cholesterol (mmol/l)	21°C,24h	9.3e-01	7.7e-01	9.9e-01
Remnant cholesterol (mmol/l)	21°C,48h	9.0e-01	6.5e-01	9.7e-01
LDL cholesterol (mmol/l)	4°C,24h	9.5e-01	8.6e-01	9.9e-01
LDL cholesterol (mmol/l)	4°C,48h	9.6e-01	8.7e-01	9.9e-01
LDL cholesterol (mmol/l)	21°C,24h	9.4e-01	8.1e-01	9.8e-01
LDL cholesterol (mmol/l)	21°C,48h	9.1e-01	7.0e-01	9.8e-01
HDL cholesterol (mmol/l)	4°C,24h	9.3e-01	8.0e-01	9.8e-01
HDL cholesterol (mmol/l)	4°C,48h	9.8e-01	9.2e-01	1.0e+00
HDL cholesterol (mmol/l)	21°C,24h	9.5e-01	8.2e-01	9.9e-01
HDL cholesterol (mmol/l)	21°C,48h	9.2e-01	7.9e-01	9.7e-01
HDL2 cholesterol (mmol/l)	4°C,24h	9.3e-01	8.0e-01	9.9e-01
HDL2 cholesterol (mmol/l)	4°C,48h	9.8e-01	9.2e-01	1.0e+00
HDL2 cholesterol (mmol/l)	21°C,24h	9.8e-01	9.1e-01	1.0e+00
HDL2 cholesterol (mmol/l)	21°C,48h	9.4e-01	8.2e-01	9.7e-01
HDL3 cholesterol (mmol/l)	4°C,24h	9.2e-01	7.9e-01	9.8e-01
HDL3 cholesterol (mmol/l)	4°C,48h	9.7e-01	8.8e-01	9.9e-01
HDL3 cholesterol (mmol/l)	21°C,24h	8.2e-01	4.8e-01	9.7e-01
HDL3 cholesterol (mmol/l)	21°C,48h	6.8e-01	3.4e-01	9.0e-01
Esterified cholesterol (mmol/l)	4°C,24h	9.6e-01	8.6e-01	9.8e-01
Esterified cholesterol (mmol/l)	4°C,48h	9.7e-01	8.8e-01	1.0e+00
Esterified cholesterol (mmol/l)	21°C,24h	9.5e-01	7.9e-01	9.9e-01
Esterified cholesterol (mmol/l)	21°C,48h	9.4e-01	7.8e-01	9.9e-01
Free cholesterol (mmol/l)	4°C,24h	9.1e-01	7.1e-01	9.7e-01
Free cholesterol (mmol/l)	4°C,48h	9.6e-01	8.3e-01	1.0e+00
Free cholesterol (mmol/l)	21°C,24h	9.5e-01	8.3e-01	9.9e-01
Free cholesterol (mmol/l)	21°C,48h	9.5e-01	8.1e-01	9.9e-01
**Glycerides and phospholipids**				

Metabolic traits	conditions	correlation	LCI	UCI
Triglycerides (mmol/l)	4°C,24h	9.7e-01	8.6e-01	1.0e+00
Triglycerides (mmol/l)	4°C,48h	1.0e+00	9.6e-01	1.0e+00
Triglycerides (mmol/l)	21°C,24h	9.6e-01	8.1e-01	1.0e+00
Triglycerides (mmol/l)	21°C,48h	8.9e-01	6.8e-01	9.8e-01
VLDL triglycerides (mmol/l)	4°C,24h	9.6e-01	8.4e-01	1.0e+00
VLDL triglycerides (mmol/l)	4°C,48h	9.9e-01	9.4e-01	1.0e+00
VLDL triglycerides (mmol/l)	21°C,24h	9.4e-01	7.8e-01	1.0e+00
VLDL triglycerides (mmol/l)	21°C,48h	8.8e-01	6.6e-01	9.8e-01
LDL triglycerides (mmol/l)	4°C,24h	9.6e-01	8.7e-01	9.9e-01
LDL triglycerides (mmol/l)	4°C,48h	9.8e-01	8.9e-01	1.0e+00
LDL triglycerides (mmol/l)	21°C,24h	9.7e-01	8.7e-01	9.9e-01
LDL triglycerides (mmol/l)	21°C,48h	9.6e-01	8.4e-01	9.9e-01
HDL triglycerides (mmol/l)	4°C,24h	9.9e-01	9.3e-01	1.0e+00
HDL triglycerides (mmol/l)	4°C,48h	9.9e-01	9.5e-01	1.0e+00
HDL triglycerides (mmol/l)	21°C,24h	9.9e-01	9.4e-01	1.0e+00
HDL triglycerides (mmol/l)	21°C,48h	9.5e-01	8.2e-01	9.9e-01
Diacylglycerol (mmol/l)	4°C,24h	5.9e-01	1.2e-01	8.8e-01
Diacylglycerol (mmol/l)	4°C,48h	4.4e-01	-1.3e-01	7.8e-01
Diacylglycerol (mmol/l)	21°C,24h	1.1e-01	-4.5e-01	6.1e-01
Diacylglycerol (mmol/l)	21°C,48h	3.6e-01	-1.8e-01	7.2e-01
Phosphoglycerides (mmol/l)	4°C,24h	9.1e-01	7.5e-01	9.7e-01
Phosphoglycerides (mmol/l)	4°C,48h	9.3e-01	7.7e-01	9.9e-01
Phosphoglycerides (mmol/l)	21°C,24h	9.7e-01	8.9e-01	9.9e-01
Phosphoglycerides (mmol/l)	21°C,48h	8.9e-01	7.6e-01	9.6e-01
Phosphatidylcholine + other cholines (mmol/l)	4°C,24h	8.7e-01	6.6e-01	9.5e-01
Phosphatidylcholine + other cholines (mmol/l)	4°C,48h	9.2e-01	7.1e-01	9.8e-01
Phosphatidylcholine + other cholines (mmol/l)	21°C,24h	9.7e-01	8.7e-01	1.0e+00
Phosphatidylcholine + other cholines (mmol/l)	21°C,48h	9.1e-01	7.1e-01	9.7e-01
Sphingomyelins (mmol/l)	4°C,24h	8.3e-01	5.8e-01	9.3e-01

Metabolic traits	conditions	correlation	LCI	UCI
Sphingomyelins (mmol/l)	4°C,48h	6.7e-01	3.2e-01	8.7e-01
Sphingomyelins (mmol/l)	21°C,24h	5.4e-01	1.3e-01	7.9e-01
Sphingomyelins (mmol/l)	21°C,48h	8.6e-01	6.6e-01	9.5e-01
Cholines (mmol/l)	4°C,24h	9.1e-01	7.2e-01	9.8e-01
Cholines (mmol/l)	4°C,48h	9.2e-01	7.4e-01	9.8e-01
Cholines (mmol/l)	21°C,24h	9.5e-01	8.2e-01	9.8e-01
Cholines (mmol/l)	21°C,48h	8.8e-01	6.7e-01	9.6e-01
**Apolipoproteins**				
Apolipoprotein A-I (g/l)	4°C,24h	9.3e-01	7.9e-01	9.8e-01
Apolipoprotein A-I (g/l)	4°C,48h	9.8e-01	9.1e-01	1.0e+00
Apolipoprotein A-I (g/l)	21°C,24h	9.8e-01	9.0e-01	1.0e+00
Apolipoprotein A-I (g/l)	21°C,48h	8.7e-01	7.2e-01	9.4e-01
Apolipoprotein B (g/l)	4°C,24h	9.1e-01	7.1e-01	9.8e-01
Apolipoprotein B (g/l)	4°C,48h	9.4e-01	8.3e-01	9.9e-01
Apolipoprotein B (g/l)	21°C,24h	9.3e-01	7.5e-01	9.8e-01
Apolipoprotein B (g/l)	21°C,48h	9.0e-01	6.7e-01	9.7e-01
**Fatty acids**				
Total fatty acids (mmol/l)	4°C,24h	9.4e-01	7.9e-01	9.9e-01
Total fatty acids (mmol/l)	4°C,48h	9.8e-01	9.0e-01	1.0e+00
Total fatty acids (mmol/l)	21°C,24h	9.7e-01	8.5e-01	1.0e+00
Total fatty acids (mmol/l)	21°C,48h	9.6e-01	8.2e-01	9.9e-01
Fatty acid chain length	4°C,24h	7.2e-01	4.3e-01	8.9e-01
Fatty acid chain length	4°C,48h	7.3e-01	4.2e-01	9.2e-01
Fatty acid chain length	21°C,24h	7.3e-01	3.9e-01	9.1e-01
Fatty acid chain length	21°C,48h	7.1e-01	3.6e-01	9.1e-01
Degree of unsaturation	4°C,24h	9.1e-01	7.1e-01	9.8e-01
Degree of unsaturation	4°C,48h	9.6e-01	8.5e-01	1.0e+00
Degree of unsaturation	21°C,24h	8.3e-01	4.8e-01	9.7e-01
Degree of unsaturation	21°C,48h	7.3e-01	2.7e-01	9.4e-01

Metabolic traits	conditions	correlation	LCI	UCI
Docosahexaenoic acid (mmol/l)	4°C,24h	9.8e-01	8.9e-01	1.0e+00
Docosahexaenoic acid (mmol/l)	4°C,48h	9.7e-01	8.8e-01	1.0e+00
Docosahexaenoic acid (mmol/l)	21°C,24h	9.8e-01	8.8e-01	1.0e+00
Docosahexaenoic acid (mmol/l)	21°C,48h	9.2e-01	7.4e-01	9.8e-01
Linoleic acid (mmol/l)	4°C,24h	9.4e-01	8.1e-01	9.8e-01
Linoleic acid (mmol/l)	4°C,48h	9.6e-01	8.4e-01	9.9e-01
Linoleic acid (mmol/l)	21°C,24h	9.5e-01	8.3e-01	9.9e-01
Linoleic acid (mmol/l)	21°C,48h	9.5e-01	8.2e-01	9.9e-01
Conjugated linoleic acid (mmol/l)	4°C,24h	8.6e-01	5.8e-01	9.7e-01
Conjugated linoleic acid (mmol/l)	4°C,48h	5.6e-01	8.3e-02	8.4e-01
Conjugated linoleic acid (mmol/l)	21°C,24h	9.1e-01	7.0e-01	9.8e-01
Conjugated linoleic acid (mmol/l)	21°C,48h	5.8e-01	4.6e-02	8.7e-01
n-3 fatty acids (mmol/l)	4°C,24h	9.6e-01	8.4e-01	9.9e-01
n-3 fatty acids (mmol/l)	4°C,48h	9.5e-01	8.1e-01	1.0e+00
n-3 fatty acids (mmol/l)	21°C,24h	9.8e-01	9.0e-01	1.0e+00
n-3 fatty acids (mmol/l)	21°C,48h	9.2e-01	7.0e-01	9.8e-01
n-6 fatty acids (mmol/l)	4°C,24h	9.6e-01	8.3e-01	1.0e+00
n-6 fatty acids (mmol/l)	4°C,48h	9.6e-01	8.5e-01	9.9e-01
n-6 fatty acids (mmol/l)	21°C,24h	9.6e-01	7.8e-01	1.0e+00
n-6 fatty acids (mmol/l)	21°C,48h	9.4e-01	7.8e-01	9.9e-01
PUFA (mmol/l)	4°C,24h	9.5e-01	7.7e-01	1.0e+00
PUFA (mmol/l)	4°C,48h	9.8e-01	8.8e-01	9.9e-01
PUFA (mmol/l)	21°C,24h	9.6e-01	8.2e-01	1.0e+00
PUFA (mmol/l)	21°C,48h	9.3e-01	7.8e-01	9.8e-01
MUFA (mmol/l)	4°C,24h	9.8e-01	9.0e-01	1.0e+00
MUFA (mmol/l)	4°C,48h	9.9e-01	9.3e-01	1.0e+00
MUFA (mmol/l)	21°C,24h	9.9e-01	9.5e-01	1.0e+00
MUFA (mmol/l)	21°C,48h	9.9e-01	9.3e-01	1.0e+00
Saturated fatty acids (mmol/l)	4°C,24h	9.1e-01	7.4e-01	9.7e-01

Metabolic traits	conditions	correlation	LCI	UCI
Saturated fatty acids (mmol/l)	4°C,48h	9.6e-01	8.1e-01	1.0e+00
Saturated fatty acids (mmol/l)	21°C,24h	9.5e-01	8.1e-01	9.9e-01
Saturated fatty acids (mmol/l)	21°C,48h	9.4e-01	7.8e-01	1.0e+00
**Glycolysis related metabolites**				
Glucose (mmol/l)	4°C,24h	9.1e-01	7.2e-01	9.7e-01
Glucose (mmol/l)	4°C,48h	7.2e-01	4.1e-01	8.7e-01
Glucose (mmol/l)	21°C,24h	7.7e-01	4.7e-01	9.3e-01
Glucose (mmol/l)	21°C,48h	4.8e-01	6.1e-02	7.5e-01
Lactate (mmol/l)	4°C,24h	7.5e-01	3.8e-01	9.3e-01
Lactate (mmol/l)	4°C,48h	3.3e-02	-4.8e-01	4.9e-01
Lactate (mmol/l)	21°C,24h	2.6e-01	-2.7e-01	6.6e-01
Lactate (mmol/l)	21°C,48h	5.2e-01	1.8e-01	7.5e-01
Citrate (mmol/l)	4°C,24h	8.5e-01	6.1e-01	9.6e-01
Citrate (mmol/l)	4°C,48h	8.8e-01	6.2e-01	9.5e-01
Citrate (mmol/l)	21°C,24h	9.0e-01	7.1e-01	9.8e-01
Citrate (mmol/l)	21°C,48h	7.8e-01	4.7e-01	9.3e-01
**Amino acids**				
Alanine (mmol/l)	4°C,24h	9.5e-01	8.4e-01	9.9e-01
Alanine (mmol/l)	4°C,48h	9.7e-01	8.7e-01	9.9e-01
Alanine (mmol/l)	21°C,24h	8.0e-01	5.3e-01	9.3e-01
Alanine (mmol/l)	21°C,48h	4.4e-01	1.3e-02	8.1e-01
Glutamine (mmol/l)	4°C,24h	9.5e-01	8.0e-01	9.8e-01
Glutamine (mmol/l)	4°C,48h	9.0e-01	7.5e-01	9.7e-01
Glutamine (mmol/l)	21°C,24h	9.7e-01	8.9e-01	9.9e-01
Glutamine (mmol/l)	21°C,48h	8.5e-01	6.2e-01	9.5e-01
Histidine (mmol/l)	4°C,24h	7.9e-01	5.3e-01	9.1e-01
Histidine (mmol/l)	4°C,48h	3.0e-01	-1.8e-01	7.0e-01
Histidine (mmol/l)	21°C,24h	6.5e-01	3.0e-01	8.4e-01
Histidine (mmol/l)	21°C,48h	1.6e-01	-3.7e-01	6.2e-01

Metabolic traits	conditions	correlation	LCI	UCI
Branched-chain amino acids				
Isoleucine (mmol/l)	4°C,24h	9.1e-01	7.1e-01	9.8e-01
Isoleucine (mmol/l)	4°C,48h	9.4e-01	7.8e-01	1.0e+00
Isoleucine (mmol/l)	21°C,24h	9.2e-01	7.4e-01	9.9e-01
Isoleucine (mmol/l)	21°C,48h	8.6e-01	5.8e-01	9.7e-01
Leucine (mmol/l)	4°C,24h	9.8e-01	9.3e-01	1.0e+00
Leucine (mmol/l)	4°C,48h	9.7e-01	9.0e-01	1.0e+00
Leucine (mmol/l)	21°C,24h	9.5e-01	8.4e-01	9.8e-01
Leucine (mmol/l)	21°C,48h	9.0e-01	6.9e-01	9.8e-01
Valine (mmol/l)	4°C,24h	9.7e-01	8.8e-01	1.0e+00
Valine (mmol/l)	4°C,48h	9.8e-01	9.2e-01	1.0e+00
Valine (mmol/l)	21°C,24h	9.8e-01	9.0e-01	1.0e+00
Valine (mmol/l)	21°C,48h	9.8e-01	9.1e-01	1.0e+00
Aromatic amino acids				
Phenylalanine (mmol/l)	4°C,24h	6.2e-01	1.8e-01	8.7e-01
Phenylalanine (mmol/l)	4°C,48h	5.4e-01	1.4e-01	8.5e-01
Phenylalanine (mmol/l)	21°C,24h	6.8e-01	2.8e-01	8.8e-01
Phenylalanine (mmol/l)	21°C,48h	3.2e-01	-1.1e-01	7.1e-01
Tyrosine (mmol/l)	4°C,24h	9.2e-01	7.6e-01	9.8e-01
Tyrosine (mmol/l)	4°C,48h	9.1e-01	7.1e-01	9.8e-01
Tyrosine (mmol/l)	21°C,24h	9.4e-01	7.9e-01	9.9e-01
Tyrosine (mmol/l)	21°C,48h	8.9e-01	6.7e-01	9.8e-01
**Ketone bodies**				
Acetate (mmol/l)	4°C,24h	4.9e-01	9.1e-02	8.0e-01
Acetate (mmol/l)	4°C,48h	2.8e-01	-1.4e-01	6.1e-01
Acetate (mmol/l)	21°C,24h	2.8e-01	-2.4e-01	6.4e-01
Acetate (mmol/l)	21°C,48h	4.1e-01	-2.5e-02	7.3e-01
Beta-hydroxybutyrate (mmol/l)	4°C,24h	9.7e-01	8.9e-01	9.9e-01
Beta-hydroxybutyrate (mmol/l)	4°C,48h	8.9e-01	6.5e-01	9.8e-01

Metabolic traits	conditions	correlation	LCI	UCI
Beta-hydroxybutyrate (mmol/l)	21°C,24h	8.8e-01	7.1e-01	9.6e-01
Beta-hydroxybutyrate (mmol/l)	21°C,48h	6.7e-01	2.9e-01	8.9e-01
**Fluid balance**				
Creatinine (mmol/l)	4°C,24h	9.4e-01	8.4e-01	9.8e-01
Creatinine (mmol/l)	4°C,48h	9.3e-01	7.8e-01	9.7e-01
Creatinine (mmol/l)	21°C,24h	9.3e-01	8.3e-01	9.8e-01
Creatinine (mmol/l)	21°C,48h	9.0e-01	7.1e-01	9.7e-01
Albumin (signal area)	4°C,24h	8.6e-01	6.7e-01	9.4e-01
Albumin (signal area)	4°C,48h	9.1e-01	7.5e-01	9.6e-01
Albumin (signal area)	21°C,24h	8.7e-01	6.8e-01	9.5e-01
Albumin (signal area)	21°C,48h	7.2e-01	3.8e-01	9.0e-01
**Inflammation**				
Glycoprotein acetyls (mmol/l)	4°C,24h	9.8e-01	8.9e-01	9.9e-01
Glycoprotein acetyls (mmol/l)	4°C,48h	9.9e-01	9.4e-01	1.0e+00
Glycoprotein acetyls (mmol/l)	21°C,24h	9.8e-01	9.2e-01	9.9e-01
Glycoprotein acetyls (mmol/l)	21°C,48h	9.6e-01	8.7e-01	9.9e-01

Table S4. Spearman's correlation: serum, post-storage handling effects. Spearman's rank correlation coefficients between metabolic concentrations (or values) in reference conditions samples (i.e. no buffer addition delay or NMR analysis delay) and samples (i) left for 24 h before addition of sodium buffer followed by immediate NMR analysis (i.e. buffer delay); and (ii) addition of sodium buffer, then left for 24 h before NMR analysis (i.e. NMR delay); (see Figures 5-6, S3).

***Abbreviations**: **C**=cholesterol; **IDL**=intermediate-density lipoprotein; **LCI**=lower confidence interval; **LDL**=low-density lipoprotein; **HDL**=high-density lipoprotein; **MUFA**=monounsaturated fatty acids; **PUFA**=polyunsaturated fatty acids; **UCI**= upper confidence interval; **VLDL**=very-low-density lipoprotein.*

Metabolic traits	conditions	correlation	LCI	UCI
**Lipoprotein subclasses**				
Extremely large VLDL				
Particle concentration (mol/l)	Buffer delay	8.9e-01	7.1e-01	9.7e-01
Particle concentration (mol/l)	NMR delay	9.7e-01	8.9e-01	1.0e+00
Total lipids (mmol/l)	Buffer delay	8.9e-01	6.9e-01	9.7e-01
Total lipids (mmol/l)	NMR delay	9.7e-01	8.9e-01	1.0e+00
Phospholipids (mmol/l)	Buffer delay	9.0e-01	7.4e-01	9.8e-01
Phospholipids (mmol/l)	NMR delay	9.7e-01	8.9e-01	1.0e+00
Total cholesterol (mmol/l)	Buffer delay	8.9e-01	6.8e-01	9.8e-01
Total cholesterol (mmol/l)	NMR delay	9.7e-01	8.7e-01	1.0e+00
Cholesterol esters (mmol/l)	Buffer delay	8.6e-01	5.9e-01	9.8e-01
Cholesterol esters (mmol/l)	NMR delay	9.2e-01	7.6e-01	9.9e-01
Free cholesterol (mmol/l)	Buffer delay	9.1e-01	7.4e-01	9.7e-01
Free cholesterol (mmol/l)	NMR delay	9.7e-01	8.8e-01	1.0e+00
Triglycerides (mmol/l)	Buffer delay	9.0e-01	7.4e-01	9.7e-01
Triglycerides (mmol/l)	NMR delay	9.7e-01	9.0e-01	9.9e-01
Very large VLDL				
Particle concentration (mol/l)	Buffer delay	9.1e-01	7.2e-01	9.9e-01
Particle concentration (mol/l)	NMR delay	9.8e-01	9.1e-01	1.0e+00
Total lipids (mmol/l)	Buffer delay	9.1e-01	7.1e-01	9.9e-01
Total lipids (mmol/l)	NMR delay	9.8e-01	9.0e-01	1.0e+00
Phospholipids (mmol/l)	Buffer delay	9.2e-01	7.3e-01	9.8e-01
Phospholipids (mmol/l)	NMR delay	9.7e-01	8.9e-01	9.9e-01

Metabolic traits	conditions	correlation	LCI	UCI
Total cholesterol (mmol/l)	Buffer delay	9.3e-01	8.0e-01	9.8e-01
Total cholesterol (mmol/l)	NMR delay	9.8e-01	9.1e-01	9.9e-01
Cholesterol esters (mmol/l)	Buffer delay	9.4e-01	7.9e-01	9.9e-01
Cholesterol esters (mmol/l)	NMR delay	9.7e-01	8.9e-01	9.9e-01
Free cholesterol (mmol/l)	Buffer delay	9.3e-01	8.1e-01	9.8e-01
Free cholesterol (mmol/l)	NMR delay	9.8e-01	8.9e-01	1.0e+00
Triglycerides (mmol/l)	Buffer delay	9.0e-01	6.9e-01	9.9e-01
Triglycerides (mmol/l)	NMR delay	9.8e-01	9.1e-01	1.0e+00
Large VLDL				
Particle concentration (mol/l)	Buffer delay	9.9e-01	9.4e-01	1.0e+00
Particle concentration (mol/l)	NMR delay	9.9e-01	9.5e-01	1.0e+00
Total lipids (mmol/l)	Buffer delay	9.9e-01	9.3e-01	1.0e+00
Total lipids (mmol/l)	NMR delay	9.9e-01	9.5e-01	1.0e+00
Phospholipids (mmol/l)	Buffer delay	9.8e-01	9.4e-01	1.0e+00
Phospholipids (mmol/l)	NMR delay	9.9e-01	9.7e-01	1.0e+00
Total cholesterol (mmol/l)	Buffer delay	9.8e-01	9.4e-01	1.0e+00
Total cholesterol (mmol/l)	NMR delay	9.9e-01	9.6e-01	1.0e+00
Cholesterol esters (mmol/l)	Buffer delay	9.7e-01	8.9e-01	9.9e-01
Cholesterol esters (mmol/l)	NMR delay	9.9e-01	9.5e-01	1.0e+00
Free cholesterol (mmol/l)	Buffer delay	9.8e-01	8.9e-01	1.0e+00
Free cholesterol (mmol/l)	NMR delay	1.0e+00	9.9e-01	1.0e+00
Triglycerides (mmol/l)	Buffer delay	9.7e-01	8.9e-01	1.0e+00
Triglycerides (mmol/l)	NMR delay	9.9e-01	9.4e-01	1.0e+00
Medium VLDL				
Particle concentration (mol/l)	Buffer delay	1.0e+00	9.8e-01	1.0e+00
Particle concentration (mol/l)	NMR delay	1.0e+00	9.6e-01	1.0e+00
Total lipids (mmol/l)	Buffer delay	9.9e-01	9.7e-01	1.0e+00
Total lipids (mmol/l)	NMR delay	9.9e-01	9.7e-01	1.0e+00
Phospholipids (mmol/l)	Buffer delay	9.9e-01	9.5e-01	1.0e+00

Metabolic traits	conditions	correlation	LCI	UCI
Phospholipids (mmol/l)	NMR delay	9.9e-01	9.6e-01	1.0e+00
Total cholesterol (mmol/l)	Buffer delay	9.6e-01	8.6e-01	9.9e-01
Total cholesterol (mmol/l)	NMR delay	9.9e-01	9.5e-01	1.0e+00
Cholesterol esters (mmol/l)	Buffer delay	9.6e-01	8.4e-01	9.9e-01
Cholesterol esters (mmol/l)	NMR delay	9.8e-01	9.2e-01	1.0e+00
Free cholesterol (mmol/l)	Buffer delay	9.9e-01	9.5e-01	1.0e+00
Free cholesterol (mmol/l)	NMR delay	1.0e+00	9.9e-01	1.0e+00
Triglycerides (mmol/l)	Buffer delay	9.9e-01	9.6e-01	1.0e+00
Triglycerides (mmol/l)	NMR delay	1.0e+00	9.9e-01	1.0e+00
Small VLDL				
Particle concentration (mol/l)	Buffer delay	9.8e-01	9.1e-01	1.0e+00
Particle concentration (mol/l)	NMR delay	9.9e-01	9.7e-01	1.0e+00
Total lipids (mmol/l)	Buffer delay	9.8e-01	9.1e-01	1.0e+00
Total lipids (mmol/l)	NMR delay	9.9e-01	9.5e-01	1.0e+00
Phospholipids (mmol/l)	Buffer delay	9.9e-01	9.6e-01	1.0e+00
Phospholipids (mmol/l)	NMR delay	9.9e-01	9.4e-01	1.0e+00
Total cholesterol (mmol/l)	Buffer delay	9.6e-01	8.4e-01	9.9e-01
Total cholesterol (mmol/l)	NMR delay	9.8e-01	9.1e-01	1.0e+00
Cholesterol esters (mmol/l)	Buffer delay	9.7e-01	8.9e-01	9.9e-01
Cholesterol esters (mmol/l)	NMR delay	9.7e-01	8.9e-01	1.0e+00
Free cholesterol (mmol/l)	Buffer delay	9.9e-01	9.4e-01	1.0e+00
Free cholesterol (mmol/l)	NMR delay	9.9e-01	9.4e-01	1.0e+00
Triglycerides (mmol/l)	Buffer delay	9.9e-01	9.5e-01	1.0e+00
Triglycerides (mmol/l)	NMR delay	9.9e-01	9.3e-01	1.0e+00
Very Small VLDL				
Particle concentration (mol/l)	Buffer delay	9.5e-01	8.3e-01	9.9e-01
Particle concentration (mol/l)	NMR delay	9.9e-01	9.3e-01	1.0e+00
Total lipids (mmol/l)	Buffer delay	9.4e-01	7.6e-01	9.9e-01
Total lipids (mmol/l)	NMR delay	9.9e-01	9.2e-01	1.0e+00

Metabolic traits	conditions	correlation	LCI	UCI
Phospholipids (mmol/l)	Buffer delay	9.6e-01	8.7e-01	9.9e-01
Phospholipids (mmol/l)	NMR delay	9.9e-01	9.5e-01	1.0e+00
Total cholesterol (mmol/l)	Buffer delay	8.6e-01	5.4e-01	9.8e-01
Total cholesterol (mmol/l)	NMR delay	9.3e-01	7.9e-01	9.8e-01
Cholesterol esters (mmol/l)	Buffer delay	8.6e-01	5.6e-01	9.8e-01
Cholesterol esters (mmol/l)	NMR delay	9.1e-01	7.1e-01	9.8e-01
Free cholesterol (mmol/l)	Buffer delay	9.4e-01	7.6e-01	9.9e-01
Free cholesterol (mmol/l)	NMR delay	9.8e-01	9.0e-01	9.9e-01
Triglycerides (mmol/l)	Buffer delay	9.9e-01	9.4e-01	1.0e+00
Triglycerides (mmol/l)	NMR delay	9.9e-01	9.5e-01	1.0e+00
IDL				
Particle concentration (mol/l)	Buffer delay	9.7e-01	8.9e-01	9.9e-01
Particle concentration (mol/l)	NMR delay	9.9e-01	9.5e-01	1.0e+00
Total lipids (mmol/l)	Buffer delay	9.7e-01	8.8e-01	9.9e-01
Total lipids (mmol/l)	NMR delay	9.8e-01	9.2e-01	1.0e+00
Phospholipids (mmol/l)	Buffer delay	9.8e-01	9.0e-01	9.9e-01
Phospholipids (mmol/l)	NMR delay	9.9e-01	9.4e-01	1.0e+00
Total cholesterol (mmol/l)	Buffer delay	9.6e-01	8.8e-01	9.9e-01
Total cholesterol (mmol/l)	NMR delay	9.7e-01	8.9e-01	9.9e-01
Cholesterol esters (mmol/l)	Buffer delay	9.4e-01	8.1e-01	9.8e-01
Cholesterol esters (mmol/l)	NMR delay	9.8e-01	9.1e-01	1.0e+00
Free cholesterol (mmol/l)	Buffer delay	9.6e-01	8.8e-01	9.8e-01
Free cholesterol (mmol/l)	NMR delay	9.8e-01	9.3e-01	9.9e-01
Triglycerides (mmol/l)	Buffer delay	9.9e-01	9.5e-01	1.0e+00
Triglycerides (mmol/l)	NMR delay	9.9e-01	9.6e-01	1.0e+00
Large LDL				
Particle concentration (mol/l)	Buffer delay	9.8e-01	9.1e-01	9.9e-01
Particle concentration (mol/l)	NMR delay	9.9e-01	9.4e-01	1.0e+00
Total lipids (mmol/l)	Buffer delay	9.7e-01	9.1e-01	9.9e-01

Metabolic traits	conditions	correlation	LCI	UCI
Total lipids (mmol/l)	NMR delay	9.9e-01	9.3e-01	1.0e+00
Phospholipids (mmol/l)	Buffer delay	9.7e-01	9.0e-01	9.9e-01
Phospholipids (mmol/l)	NMR delay	9.8e-01	9.2e-01	9.9e-01
Total cholesterol (mmol/l)	Buffer delay	9.7e-01	9.0e-01	9.9e-01
Total cholesterol (mmol/l)	NMR delay	9.9e-01	9.4e-01	1.0e+00
Cholesterol esters (mmol/l)	Buffer delay	9.7e-01	9.0e-01	9.9e-01
Cholesterol esters (mmol/l)	NMR delay	9.9e-01	9.3e-01	1.0e+00
Free cholesterol (mmol/l)	Buffer delay	9.8e-01	9.3e-01	9.9e-01
Free cholesterol (mmol/l)	NMR delay	9.8e-01	9.4e-01	1.0e+00
Triglycerides (mmol/l)	Buffer delay	9.9e-01	9.6e-01	1.0e+00
Triglycerides (mmol/l)	NMR delay	9.9e-01	9.7e-01	1.0e+00
Medium LDL				
Particle concentration (mol/l)	Buffer delay	9.8e-01	9.1e-01	9.9e-01
Particle concentration (mol/l)	NMR delay	9.7e-01	9.0e-01	9.9e-01
Total lipids (mmol/l)	Buffer delay	9.7e-01	9.0e-01	9.9e-01
Total lipids (mmol/l)	NMR delay	9.9e-01	9.4e-01	1.0e+00
Phospholipids (mmol/l)	Buffer delay	9.7e-01	9.1e-01	9.9e-01
Phospholipids (mmol/l)	NMR delay	9.8e-01	9.2e-01	1.0e+00
Total cholesterol (mmol/l)	Buffer delay	9.7e-01	9.1e-01	9.9e-01
Total cholesterol (mmol/l)	NMR delay	9.9e-01	9.3e-01	1.0e+00
Cholesterol esters (mmol/l)	Buffer delay	9.8e-01	8.9e-01	1.0e+00
Cholesterol esters (mmol/l)	NMR delay	9.8e-01	9.2e-01	9.9e-01
Free cholesterol (mmol/l)	Buffer delay	9.8e-01	9.1e-01	9.9e-01
Free cholesterol (mmol/l)	NMR delay	9.8e-01	8.9e-01	9.9e-01
Triglycerides (mmol/l)	Buffer delay	9.8e-01	9.2e-01	1.0e+00
Triglycerides (mmol/l)	NMR delay	9.8e-01	9.2e-01	1.0e+00
Small LDL				
Particle concentration (mol/l)	Buffer delay	9.8e-01	9.3e-01	1.0e+00
Particle concentration (mol/l)	NMR delay	9.8e-01	8.9e-01	1.0e+00

Metabolic traits	conditions	correlation	LCI	UCI
Total lipids (mmol/l)	Buffer delay	9.7e-01	8.8e-01	9.9e-01
Total lipids (mmol/l)	NMR delay	9.8e-01	9.1e-01	1.0e+00
Phospholipids (mmol/l)	Buffer delay	9.8e-01	9.3e-01	9.9e-01
Phospholipids (mmol/l)	NMR delay	9.8e-01	9.3e-01	1.0e+00
Total cholesterol (mmol/l)	Buffer delay	9.8e-01	9.0e-01	1.0e+00
Total cholesterol (mmol/l)	NMR delay	9.8e-01	9.3e-01	1.0e+00
Cholesterol esters (mmol/l)	Buffer delay	9.4e-01	8.0e-01	9.9e-01
Cholesterol esters (mmol/l)	NMR delay	9.9e-01	9.5e-01	1.0e+00
Free cholesterol (mmol/l)	Buffer delay	9.8e-01	9.3e-01	9.9e-01
Free cholesterol (mmol/l)	NMR delay	9.8e-01	9.0e-01	9.9e-01
Triglycerides (mmol/l)	Buffer delay	9.9e-01	9.6e-01	1.0e+00
Triglycerides (mmol/l)	NMR delay	9.9e-01	9.6e-01	1.0e+00
Very large HDL				
Particle concentration (mol/l)	Buffer delay	9.8e-01	9.1e-01	1.0e+00
Particle concentration (mol/l)	NMR delay	9.9e-01	9.7e-01	1.0e+00
Total lipids (mmol/l)	Buffer delay	9.8e-01	9.1e-01	1.0e+00
Total lipids (mmol/l)	NMR delay	9.9e-01	9.6e-01	1.0e+00
Phospholipids (mmol/l)	Buffer delay	9.9e-01	9.6e-01	1.0e+00
Phospholipids (mmol/l)	NMR delay	1.0e+00	9.7e-01	1.0e+00
Total cholesterol (mmol/l)	Buffer delay	9.7e-01	8.6e-01	1.0e+00
Total cholesterol (mmol/l)	NMR delay	9.8e-01	9.2e-01	1.0e+00
Cholesterol esters (mmol/l)	Buffer delay	9.7e-01	8.4e-01	1.0e+00
Cholesterol esters (mmol/l)	NMR delay	9.9e-01	9.3e-01	1.0e+00
Free cholesterol (mmol/l)	Buffer delay	9.8e-01	9.2e-01	1.0e+00
Free cholesterol (mmol/l)	NMR delay	9.9e-01	9.3e-01	1.0e+00
Triglycerides (mmol/l)	Buffer delay	9.5e-01	8.0e-01	9.9e-01
Triglycerides (mmol/l)	NMR delay	9.9e-01	9.3e-01	1.0e+00
Large HDL				
Particle concentration (mol/l)	Buffer delay	9.9e-01	9.4e-01	1.0e+00

Metabolic traits	conditions	correlation	LCI	UCI
Particle concentration (mol/l)	NMR delay	9.9e-01	9.6e-01	1.0e+00
Total lipids (mmol/l)	Buffer delay	9.9e-01	9.6e-01	1.0e+00
Total lipids (mmol/l)	NMR delay	9.9e-01	9.7e-01	1.0e+00
Phospholipids (mmol/l)	Buffer delay	9.9e-01	9.5e-01	1.0e+00
Phospholipids (mmol/l)	NMR delay	9.9e-01	9.5e-01	1.0e+00
Total cholesterol (mmol/l)	Buffer delay	1.0e+00	9.7e-01	1.0e+00
Total cholesterol (mmol/l)	NMR delay	1.0e+00	9.7e-01	1.0e+00
Cholesterol esters (mmol/l)	Buffer delay	9.9e-01	9.6e-01	1.0e+00
Cholesterol esters (mmol/l)	NMR delay	1.0e+00	9.7e-01	1.0e+00
Free cholesterol (mmol/l)	Buffer delay	9.9e-01	9.5e-01	1.0e+00
Free cholesterol (mmol/l)	NMR delay	9.9e-01	9.7e-01	1.0e+00
Triglycerides (mmol/l)	Buffer delay	9.8e-01	9.4e-01	1.0e+00
Triglycerides (mmol/l)	NMR delay	9.9e-01	9.5e-01	1.0e+00
Medium HDL				
Particle concentration (mol/l)	Buffer delay	8.9e-01	7.1e-01	9.8e-01
Particle concentration (mol/l)	NMR delay	9.7e-01	8.8e-01	9.9e-01
Total lipids (mmol/l)	Buffer delay	8.9e-01	7.1e-01	9.8e-01
Total lipids (mmol/l)	NMR delay	9.7e-01	8.9e-01	9.9e-01
Phospholipids (mmol/l)	Buffer delay	9.3e-01	7.8e-01	9.8e-01
Phospholipids (mmol/l)	NMR delay	9.7e-01	9.0e-01	9.9e-01
Total cholesterol (mmol/l)	Buffer delay	9.1e-01	7.3e-01	9.9e-01
Total cholesterol (mmol/l)	NMR delay	9.6e-01	8.4e-01	1.0e+00
Cholesterol esters (mmol/l)	Buffer delay	9.0e-01	6.7e-01	9.8e-01
Cholesterol esters (mmol/l)	NMR delay	9.5e-01	8.2e-01	9.9e-01
Free cholesterol (mmol/l)	Buffer delay	9.2e-01	7.6e-01	9.8e-01
Free cholesterol (mmol/l)	NMR delay	9.6e-01	8.6e-01	9.9e-01
Triglycerides (mmol/l)	Buffer delay	9.8e-01	8.9e-01	1.0e+00
Triglycerides (mmol/l)	NMR delay	9.8e-01	9.1e-01	1.0e+00
Small HDL				

Metabolic traits	conditions	correlation	LCI	UCI
Particle concentration (mol/l)	Buffer delay	8.8e-01	7.1e-01	9.7e-01
Particle concentration (mol/l)	NMR delay	9.3e-01	7.7e-01	9.9e-01
Total lipids (mmol/l)	Buffer delay	8.6e-01	6.9e-01	9.6e-01
Total lipids (mmol/l)	NMR delay	9.3e-01	7.9e-01	9.9e-01
Phospholipids (mmol/l)	Buffer delay	9.8e-01	8.9e-01	1.0e+00
Phospholipids (mmol/l)	NMR delay	9.8e-01	9.2e-01	1.0e+00
Total cholesterol (mmol/l)	Buffer delay	7.4e-01	3.3e-01	9.5e-01
Total cholesterol (mmol/l)	NMR delay	7.8e-01	4.4e-01	9.8e-01
Cholesterol esters (mmol/l)	Buffer delay	8.7e-01	6.1e-01	9.7e-01
Cholesterol esters (mmol/l)	NMR delay	8.7e-01	6.2e-01	9.9e-01
Free cholesterol (mmol/l)	Buffer delay	9.7e-01	8.8e-01	9.9e-01
Free cholesterol (mmol/l)	NMR delay	9.8e-01	9.1e-01	9.9e-01
Triglycerides (mmol/l)	Buffer delay	9.8e-01	9.1e-01	1.0e+00
Triglycerides (mmol/l)	NMR delay	9.6e-01	8.8e-01	9.9e-01
**Lipoprotein particle size**				
VLDL particle size (nm)	Buffer delay	9.9e-01	9.5e-01	1.0e+00
VLDL particle size (nm)	NMR delay	9.9e-01	9.5e-01	1.0e+00
LDL particle size (nm)	Buffer delay	8.2e-01	5.6e-01	9.5e-01
LDL particle size (nm)	NMR delay	9.3e-01	7.9e-01	9.8e-01
HDL particle size (nm)	Buffer delay	9.9e-01	9.5e-01	1.0e+00
HDL particle size (nm)	NMR delay	9.9e-01	9.5e-01	1.0e+00
**Cholesterol**				
Total cholesterol (mmol/l)	Buffer delay	9.9e-01	9.4e-01	1.0e+00
Total cholesterol (mmol/l)	NMR delay	9.9e-01	9.4e-01	1.0e+00
VLDL cholesterol (mmol/l)	Buffer delay	9.4e-01	7.9e-01	9.9e-01
VLDL cholesterol (mmol/l)	NMR delay	9.9e-01	9.3e-01	1.0e+00
Remnant cholesterol (mmol/l)	Buffer delay	9.5e-01	8.2e-01	1.0e+00
Remnant cholesterol (mmol/l)	NMR delay	9.9e-01	9.4e-01	1.0e+00
LDL cholesterol (mmol/l)	Buffer delay	9.7e-01	8.7e-01	9.9e-01

Metabolic traits	conditions	correlation	LCI	UCI
LDL cholesterol (mmol/l)	NMR delay	9.9e-01	9.6e-01	1.0e+00
HDL cholesterol (mmol/l)	Buffer delay	9.9e-01	9.5e-01	1.0e+00
HDL cholesterol (mmol/l)	NMR delay	9.9e-01	9.4e-01	1.0e+00
HDL2 cholesterol (mmol/l)	Buffer delay	9.9e-01	9.3e-01	1.0e+00
HDL2 cholesterol (mmol/l)	NMR delay	9.8e-01	9.1e-01	1.0e+00
HDL3 cholesterol (mmol/l)	Buffer delay	9.8e-01	9.0e-01	1.0e+00
HDL3 cholesterol (mmol/l)	NMR delay	9.8e-01	9.3e-01	1.0e+00
Esterified cholesterol (mmol/l)	Buffer delay	9.8e-01	9.3e-01	1.0e+00
Esterified cholesterol (mmol/l)	NMR delay	9.9e-01	9.3e-01	1.0e+00
Free cholesterol (mmol/l)	Buffer delay	9.7e-01	8.4e-01	9.9e-01
Free cholesterol (mmol/l)	NMR delay	9.7e-01	8.6e-01	1.0e+00
**Glycerides and phospholipids**				
Triglycerides (mmol/l)	Buffer delay	9.9e-01	9.4e-01	1.0e+00
Triglycerides (mmol/l)	NMR delay	1.0e+00	9.7e-01	1.0e+00
VLDL triglycerides (mmol/l)	Buffer delay	9.9e-01	9.4e-01	1.0e+00
VLDL triglycerides (mmol/l)	NMR delay	9.9e-01	9.4e-01	1.0e+00
LDL triglycerides (mmol/l)	Buffer delay	1.0e+00	9.8e-01	1.0e+00
LDL triglycerides (mmol/l)	NMR delay	9.9e-01	9.5e-01	1.0e+00
HDL triglycerides (mmol/l)	Buffer delay	9.9e-01	9.5e-01	1.0e+00
HDL triglycerides (mmol/l)	NMR delay	9.9e-01	9.6e-01	1.0e+00
Diacylglycerol (mmol/l)	Buffer delay	4.1e-01	-1.4e-01	7.5e-01
Diacylglycerol (mmol/l)	NMR delay	1.9e-01	-5.2e-01	6.9e-01
Phosphoglycerides (mmol/l)	Buffer delay	9.2e-01	7.6e-01	9.7e-01
Phosphoglycerides (mmol/l)	NMR delay	9.2e-01	7.2e-01	9.7e-01
Phosphatidylcholine + other cholines (mmol/l)	Buffer delay	9.2e-01	7.5e-01	9.7e-01
Phosphatidylcholine + other cholines (mmol/l)	NMR delay	9.3e-01	7.8e-01	9.9e-01
Sphingomyelins (mmol/l)	Buffer delay	8.6e-01	6.6e-01	9.4e-01
Sphingomyelins (mmol/l)	NMR delay	8.6e-01	5.8e-01	9.6e-01
Cholines (mmol/l)	Buffer delay	8.8e-01	6.6e-01	9.7e-01

Metabolic traits	conditions	correlation	LCI	UCI
Cholines (mmol/l)	NMR delay	8.7e-01	6.3e-01	9.5e-01
**Apolipoproteins**				
Apolipoprotein A-I (g/l)	Buffer delay	9.8e-01	9.1e-01	9.9e-01
Apolipoprotein A-I (g/l)	NMR delay	9.8e-01	9.1e-01	9.9e-01
Apolipoprotein B (g/l)	Buffer delay	9.4e-01	8.3e-01	9.8e-01
Apolipoprotein B (g/l)	NMR delay	9.9e-01	9.6e-01	1.0e+00
**Fatty acids**				
Total fatty acids (mmol/l)	Buffer delay	9.4e-01	8.0e-01	9.9e-01
Total fatty acids (mmol/l)	NMR delay	9.8e-01	8.8e-01	9.9e-01
Fatty acid chain length	Buffer delay	9.3e-01	7.9e-01	9.8e-01
Fatty acid chain length	NMR delay	9.0e-01	7.3e-01	9.7e-01
Degree of unsaturation	Buffer delay	8.8e-01	6.8e-01	9.7e-01
Degree of unsaturation	NMR delay	9.6e-01	8.2e-01	9.9e-01
Docosahexaenoic acid (mmol/l)	Buffer delay	9.7e-01	8.9e-01	9.9e-01
Docosahexaenoic acid (mmol/l)	NMR delay	9.8e-01	9.0e-01	1.0e+00
Linoleic acid (mmol/l)	Buffer delay	9.6e-01	8.6e-01	9.9e-01
Linoleic acid (mmol/l)	NMR delay	9.6e-01	8.3e-01	9.9e-01
Conjugated linoleic acid (mmol/l)	Buffer delay	5.8e-01	1.3e-01	8.5e-01
Conjugated linoleic acid (mmol/l)	NMR delay	7.9e-01	4.0e-01	9.4e-01
n-3 fatty acids (mmol/l)	Buffer delay	9.6e-01	8.7e-01	9.9e-01
n-3 fatty acids (mmol/l)	NMR delay	9.8e-01	9.1e-01	9.9e-01
n-6 fatty acids (mmol/l)	Buffer delay	9.6e-01	8.5e-01	9.9e-01
n-6 fatty acids (mmol/l)	NMR delay	9.6e-01	8.5e-01	9.9e-01
PUFA (mmol/l)	Buffer delay	9.7e-01	8.8e-01	9.9e-01
PUFA (mmol/l)	NMR delay	9.6e-01	8.7e-01	9.9e-01
MUFA (mmol/l)	Buffer delay	1.0e+00	9.7e-01	1.0e+00
MUFA (mmol/l)	NMR delay	9.9e-01	9.6e-01	1.0e+00
Saturated fatty acids (mmol/l)	Buffer delay	9.3e-01	7.5e-01	9.9e-01
Saturated fatty acids (mmol/l)	NMR delay	9.8e-01	9.4e-01	1.0e+00

Metabolic traits	conditions	correlation	LCI	UCI
**Glycolysis related metabolites**				
Glucose (mmol/l)	Buffer delay	9.7e-01	8.6e-01	1.0e+00
Glucose (mmol/l)	NMR delay	9.8e-01	9.0e-01	1.0e+00
Lactate (mmol/l)	Buffer delay	9.8e-01	9.2e-01	1.0e+00
Lactate (mmol/l)	NMR delay	9.9e-01	9.4e-01	1.0e+00
Pyruvate (mmol/l)	Buffer delay	9.8e-01	9.4e-01	9.9e-01
Pyruvate (mmol/l)	NMR delay	9.9e-01	9.5e-01	9.9e-01
Citrate (mmol/l)	Buffer delay	7.7e-01	5.3e-01	9.2e-01
Citrate (mmol/l)	NMR delay	8.9e-01	7.2e-01	9.6e-01
Glycerol (mmol/l)	Buffer delay	8.9e-01	7.8e-01	9.5e-01
Glycerol (mmol/l)	NMR delay	9.2e-01	8.0e-01	9.7e-01
**Amino acids**				
Alanine (mmol/l)	Buffer delay	9.7e-01	8.9e-01	9.9e-01
Alanine (mmol/l)	NMR delay	9.8e-01	9.2e-01	1.0e+00
Glutamine (mmol/l)	Buffer delay	9.6e-01	8.5e-01	9.8e-01
Glutamine (mmol/l)	NMR delay	9.5e-01	8.5e-01	9.8e-01
Histidine (mmol/l)	Buffer delay	4.8e-01	9.2e-02	7.9e-01
Histidine (mmol/l)	NMR delay	7.7e-01	4.6e-01	9.0e-01
Glycine (mmol/l)	Buffer delay	9.7e-01	9.1e-01	9.9e-01
Glycine (mmol/l)	NMR delay	9.6e-01	8.7e-01	9.9e-01
Branched-chain amino acids				
Isoleucine (mmol/l)	Buffer delay	9.6e-01	8.9e-01	9.9e-01
Isoleucine (mmol/l)	NMR delay	9.6e-01	8.9e-01	9.9e-01
Leucine (mmol/l)	Buffer delay	9.8e-01	9.1e-01	1.0e+00
Leucine (mmol/l)	NMR delay	9.9e-01	9.7e-01	1.0e+00
Valine (mmol/l)	Buffer delay	9.9e-01	9.5e-01	1.0e+00
Valine (mmol/l)	NMR delay	9.9e-01	9.4e-01	1.0e+00
Aromatic amino acids				
Phenylalanine (mmol/l)	Buffer delay	7.7e-01	5.5e-01	8.8e-01

Metabolic traits	conditions	correlation	LCI	UCI
Phenylalanine (mmol/l)	NMR delay	9.0e-01	6.9e-01	9.6e-01
Tyrosine (mmol/l)	Buffer delay	9.6e-01	8.6e-01	9.9e-01
Tyrosine (mmol/l)	NMR delay	9.5e-01	8.2e-01	9.9e-01
**Ketone bodies**				
Acetate (mmol/l)	Buffer delay	9.5e-01	8.4e-01	9.8e-01
Acetate (mmol/l)	NMR delay	9.7e-01	9.1e-01	1.0e+00
Beta-hydroxybutyrate (mmol/l)	Buffer delay	9.6e-01	8.8e-01	9.9e-01
Beta-hydroxybutyrate (mmol/l)	NMR delay	9.7e-01	8.9e-01	9.9e-01
**Fluid balance**				
Creatinine (mmol/l)	Buffer delay	9.2e-01	7.9e-01	9.7e-01
Creatinine (mmol/l)	NMR delay	9.2e-01	8.1e-01	9.7e-01
Albumin (signal area)	Buffer delay	9.2e-01	7.8e-01	9.7e-01
Albumin (signal area)	NMR delay	9.5e-01	8.4e-01	9.8e-01
**Inflammation**				
Glycoprotein acetyls (mmol/l)	Buffer delay	9.8e-01	9.3e-01	1.0e+00
Glycoprotein acetyls (mmol/l)	NMR delay	9.8e-01	9.3e-01	1.0e+00

Table S5. Spearman's correlation: EDTA-plasma, post-storage handling effects. Spearman's rank correlation coefficients between metabolic concentrations (or values) in reference conditions samples (i.e. no buffer addition delay or NMR analysis delay) and samples (i) left for 24 h before addition of sodium buffer followed by immediate NMR analysis (i.e. buffer delay); and (ii) addition of sodium buffer, then left for 24 h before NMR analysis (i.e. NMR delay); (see Figures 7-8, S4). Pyruvate, glycerol and glycine are not quantified in EDTA-plasma samples due to the interfering resonances of EDTA on their signals.

***Abbreviations**: **C**=cholesterol; **IDL**=intermediate-density lipoprotein; **LCI**=lower confidence interval; **LDL**=low-density lipoprotein; **HDL**=high-density lipoprotein; **MUFA**=monounsaturated fatty acids; **PUFA**=polyunsaturated fatty acids; **UCI**= upper confidence interval; **VLDL**=very-low-density lipoprotein.*

Metabolic traits	conditions	correlation	LCI	UCI
**Lipoprotein subclasses**				
Extremely large VLDL				
Particle concentration (mol/l)	Buffer delay	9.6e-01	8.6e-01	9.9e-01
Particle concentration (mol/l)	NMR delay	9.1e-01	7.6e-01	9.7e-01
Total lipids (mmol/l)	Buffer delay	9.6e-01	8.6e-01	9.9e-01
Total lipids (mmol/l)	NMR delay	9.2e-01	7.8e-01	9.7e-01
Phospholipids (mmol/l)	Buffer delay	9.6e-01	8.6e-01	1.0e+00
Phospholipids (mmol/l)	NMR delay	9.3e-01	7.9e-01	9.9e-01
Total cholesterol (mmol/l)	Buffer delay	9.6e-01	8.9e-01	9.9e-01
Total cholesterol (mmol/l)	NMR delay	9.4e-01	8.5e-01	9.8e-01
Cholesterol esters (mmol/l)	Buffer delay	9.6e-01	8.4e-01	9.9e-01
Cholesterol esters (mmol/l)	NMR delay	9.3e-01	8.2e-01	9.8e-01
Free cholesterol (mmol/l)	Buffer delay	9.7e-01	8.7e-01	1.0e+00
Free cholesterol (mmol/l)	NMR delay	9.4e-01	8.1e-01	9.9e-01
Triglycerides (mmol/l)	Buffer delay	9.6e-01	8.6e-01	9.9e-01
Triglycerides (mmol/l)	NMR delay	9.1e-01	7.9e-01	9.7e-01
Very large VLDL				
Particle concentration (mol/l)	Buffer delay	9.8e-01	9.3e-01	1.0e+00
Particle concentration (mol/l)	NMR delay	9.3e-01	8.2e-01	9.8e-01
Total lipids (mmol/l)	Buffer delay	9.8e-01	9.3e-01	1.0e+00
Total lipids (mmol/l)	NMR delay	9.4e-01	8.1e-01	9.8e-01
Phospholipids (mmol/l)	Buffer delay	9.8e-01	9.3e-01	1.0e+00

Metabolic traits	conditions	correlation	LCI	UCI
Phospholipids (mmol/l)	NMR delay	9.4e-01	8.3e-01	9.9e-01
Total cholesterol (mmol/l)	Buffer delay	9.7e-01	9.2e-01	1.0e+00
Total cholesterol (mmol/l)	NMR delay	9.6e-01	8.8e-01	9.8e-01
Cholesterol esters (mmol/l)	Buffer delay	9.8e-01	9.2e-01	1.0e+00
Cholesterol esters (mmol/l)	NMR delay	9.6e-01	8.9e-01	9.9e-01
Free cholesterol (mmol/l)	Buffer delay	9.8e-01	9.2e-01	1.0e+00
Free cholesterol (mmol/l)	NMR delay	9.6e-01	8.7e-01	9.9e-01
Triglycerides (mmol/l)	Buffer delay	9.8e-01	9.3e-01	1.0e+00
Triglycerides (mmol/l)	NMR delay	9.3e-01	7.8e-01	9.9e-01
Large VLDL				
Particle concentration (mol/l)	Buffer delay	9.7e-01	9.1e-01	1.0e+00
Particle concentration (mol/l)	NMR delay	9.8e-01	9.3e-01	1.0e+00
Total lipids (mmol/l)	Buffer delay	9.7e-01	8.9e-01	9.9e-01
Total lipids (mmol/l)	NMR delay	9.8e-01	9.3e-01	1.0e+00
Phospholipids (mmol/l)	Buffer delay	9.8e-01	9.2e-01	9.9e-01
Phospholipids (mmol/l)	NMR delay	9.8e-01	9.2e-01	1.0e+00
Total cholesterol (mmol/l)	Buffer delay	9.7e-01	9.0e-01	9.9e-01
Total cholesterol (mmol/l)	NMR delay	9.7e-01	8.9e-01	9.9e-01
Cholesterol esters (mmol/l)	Buffer delay	9.5e-01	8.3e-01	9.8e-01
Cholesterol esters (mmol/l)	NMR delay	9.4e-01	8.1e-01	9.9e-01
Free cholesterol (mmol/l)	Buffer delay	9.7e-01	9.0e-01	9.9e-01
Free cholesterol (mmol/l)	NMR delay	9.7e-01	8.8e-01	9.9e-01
Triglycerides (mmol/l)	Buffer delay	9.8e-01	9.0e-01	1.0e+00
Triglycerides (mmol/l)	NMR delay	9.9e-01	9.5e-01	1.0e+00
Medium VLDL				
Particle concentration (mol/l)	Buffer delay	9.7e-01	8.7e-01	1.0e+00
Particle concentration (mol/l)	NMR delay	9.8e-01	9.1e-01	1.0e+00
Total lipids (mmol/l)	Buffer delay	9.7e-01	8.8e-01	9.9e-01
Total lipids (mmol/l)	NMR delay	9.8e-01	9.1e-01	1.0e+00

Metabolic traits	conditions	correlation	LCI	UCI
Phospholipids (mmol/l)	Buffer delay	9.6e-01	8.6e-01	9.9e-01
Phospholipids (mmol/l)	NMR delay	9.8e-01	9.2e-01	1.0e+00
Total cholesterol (mmol/l)	Buffer delay	9.4e-01	7.9e-01	9.9e-01
Total cholesterol (mmol/l)	NMR delay	9.7e-01	8.9e-01	9.9e-01
Cholesterol esters (mmol/l)	Buffer delay	9.7e-01	8.9e-01	9.9e-01
Cholesterol esters (mmol/l)	NMR delay	9.8e-01	9.0e-01	1.0e+00
Free cholesterol (mmol/l)	Buffer delay	9.8e-01	9.2e-01	1.0e+00
Free cholesterol (mmol/l)	NMR delay	9.8e-01	9.2e-01	1.0e+00
Triglycerides (mmol/l)	Buffer delay	9.9e-01	9.4e-01	1.0e+00
Triglycerides (mmol/l)	NMR delay	9.9e-01	9.6e-01	1.0e+00
Small VLDL				
Particle concentration (mol/l)	Buffer delay	9.9e-01	9.5e-01	1.0e+00
Particle concentration (mol/l)	NMR delay	9.8e-01	9.1e-01	9.9e-01
Total lipids (mmol/l)	Buffer delay	9.8e-01	9.2e-01	1.0e+00
Total lipids (mmol/l)	NMR delay	9.8e-01	9.3e-01	1.0e+00
Phospholipids (mmol/l)	Buffer delay	9.8e-01	9.1e-01	9.9e-01
Phospholipids (mmol/l)	NMR delay	9.8e-01	9.0e-01	9.9e-01
Total cholesterol (mmol/l)	Buffer delay	9.8e-01	9.0e-01	1.0e+00
Total cholesterol (mmol/l)	NMR delay	9.7e-01	9.0e-01	9.9e-01
Cholesterol esters (mmol/l)	Buffer delay	9.6e-01	8.5e-01	9.9e-01
Cholesterol esters (mmol/l)	NMR delay	9.6e-01	8.8e-01	9.9e-01
Free cholesterol (mmol/l)	Buffer delay	9.8e-01	9.3e-01	1.0e+00
Free cholesterol (mmol/l)	NMR delay	9.7e-01	8.8e-01	1.0e+00
Triglycerides (mmol/l)	Buffer delay	9.9e-01	9.6e-01	1.0e+00
Triglycerides (mmol/l)	NMR delay	9.9e-01	9.3e-01	1.0e+00
Very Small VLDL				
Particle concentration (mol/l)	Buffer delay	9.6e-01	8.5e-01	9.9e-01
Particle concentration (mol/l)	NMR delay	9.6e-01	8.4e-01	9.9e-01
Total lipids (mmol/l)	Buffer delay	9.6e-01	8.4e-01	9.9e-01

Metabolic traits	conditions	correlation	LCI	UCI
Total lipids (mmol/l)	NMR delay	9.5e-01	7.9e-01	9.9e-01
Phospholipids (mmol/l)	Buffer delay	9.5e-01	8.3e-01	9.9e-01
Phospholipids (mmol/l)	NMR delay	9.8e-01	9.1e-01	9.9e-01
Total cholesterol (mmol/l)	Buffer delay	9.3e-01	7.6e-01	9.9e-01
Total cholesterol (mmol/l)	NMR delay	9.1e-01	7.1e-01	9.8e-01
Cholesterol esters (mmol/l)	Buffer delay	9.1e-01	7.0e-01	9.8e-01
Cholesterol esters (mmol/l)	NMR delay	8.9e-01	6.6e-01	9.7e-01
Free cholesterol (mmol/l)	Buffer delay	9.6e-01	8.9e-01	9.9e-01
Free cholesterol (mmol/l)	NMR delay	9.7e-01	9.0e-01	1.0e+00
Triglycerides (mmol/l)	Buffer delay	9.7e-01	8.9e-01	9.9e-01
Triglycerides (mmol/l)	NMR delay	9.7e-01	8.8e-01	1.0e+00
IDL				
Particle concentration (mol/l)	Buffer delay	9.8e-01	9.3e-01	1.0e+00
Particle concentration (mol/l)	NMR delay	9.8e-01	9.2e-01	9.9e-01
Total lipids (mmol/l)	Buffer delay	9.8e-01	9.2e-01	9.9e-01
Total lipids (mmol/l)	NMR delay	9.7e-01	8.9e-01	9.9e-01
Phospholipids (mmol/l)	Buffer delay	9.8e-01	9.1e-01	9.9e-01
Phospholipids (mmol/l)	NMR delay	9.7e-01	8.8e-01	9.9e-01
Total cholesterol (mmol/l)	Buffer delay	9.7e-01	9.1e-01	9.9e-01
Total cholesterol (mmol/l)	NMR delay	9.6e-01	8.7e-01	9.8e-01
Cholesterol esters (mmol/l)	Buffer delay	9.7e-01	8.9e-01	9.9e-01
Cholesterol esters (mmol/l)	NMR delay	9.4e-01	8.5e-01	9.7e-01
Free cholesterol (mmol/l)	Buffer delay	9.7e-01	8.9e-01	9.9e-01
Free cholesterol (mmol/l)	NMR delay	9.7e-01	8.8e-01	9.9e-01
Triglycerides (mmol/l)	Buffer delay	9.8e-01	9.1e-01	1.0e+00
Triglycerides (mmol/l)	NMR delay	9.8e-01	9.2e-01	1.0e+00
Large LDL				
Particle concentration (mol/l)	Buffer delay	9.9e-01	9.4e-01	1.0e+00
Particle concentration (mol/l)	NMR delay	9.8e-01	9.2e-01	9.9e-01

Metabolic traits	conditions	correlation	LCI	UCI
Total lipids (mmol/l)	Buffer delay	9.8e-01	9.3e-01	1.0e+00
Total lipids (mmol/l)	NMR delay	9.8e-01	9.1e-01	9.9e-01
Phospholipids (mmol/l)	Buffer delay	9.8e-01	9.3e-01	1.0e+00
Phospholipids (mmol/l)	NMR delay	9.8e-01	9.0e-01	1.0e+00
Total cholesterol (mmol/l)	Buffer delay	9.9e-01	9.4e-01	1.0e+00
Total cholesterol (mmol/l)	NMR delay	9.7e-01	9.0e-01	9.9e-01
Cholesterol esters (mmol/l)	Buffer delay	9.7e-01	9.1e-01	9.9e-01
Cholesterol esters (mmol/l)	NMR delay	9.7e-01	9.0e-01	9.9e-01
Free cholesterol (mmol/l)	Buffer delay	9.8e-01	8.9e-01	1.0e+00
Free cholesterol (mmol/l)	NMR delay	9.6e-01	8.7e-01	9.9e-01
Triglycerides (mmol/l)	Buffer delay	9.7e-01	8.9e-01	9.9e-01
Triglycerides (mmol/l)	NMR delay	9.7e-01	9.1e-01	9.9e-01
Medium LDL				
Particle concentration (mol/l)	Buffer delay	9.8e-01	9.1e-01	9.9e-01
Particle concentration (mol/l)	NMR delay	9.8e-01	9.3e-01	1.0e+00
Total lipids (mmol/l)	Buffer delay	9.8e-01	9.2e-01	1.0e+00
Total lipids (mmol/l)	NMR delay	9.8e-01	9.3e-01	1.0e+00
Phospholipids (mmol/l)	Buffer delay	9.9e-01	9.4e-01	1.0e+00
Phospholipids (mmol/l)	NMR delay	9.9e-01	9.5e-01	1.0e+00
Total cholesterol (mmol/l)	Buffer delay	9.8e-01	9.2e-01	9.9e-01
Total cholesterol (mmol/l)	NMR delay	9.7e-01	9.1e-01	9.9e-01
Cholesterol esters (mmol/l)	Buffer delay	9.8e-01	9.2e-01	9.9e-01
Cholesterol esters (mmol/l)	NMR delay	9.8e-01	9.3e-01	9.9e-01
Free cholesterol (mmol/l)	Buffer delay	9.9e-01	9.4e-01	1.0e+00
Free cholesterol (mmol/l)	NMR delay	9.7e-01	9.1e-01	9.9e-01
Triglycerides (mmol/l)	Buffer delay	9.4e-01	8.4e-01	9.8e-01
Triglycerides (mmol/l)	NMR delay	9.5e-01	8.2e-01	9.8e-01
Small LDL				
Particle concentration (mol/l)	Buffer delay	9.8e-01	9.0e-01	9.9e-01

Metabolic traits	conditions	correlation	LCI	UCI
Particle concentration (mol/l)	NMR delay	9.8e-01	9.3e-01	1.0e+00
Total lipids (mmol/l)	Buffer delay	9.7e-01	9.0e-01	9.9e-01
Total lipids (mmol/l)	NMR delay	9.8e-01	9.3e-01	1.0e+00
Phospholipids (mmol/l)	Buffer delay	9.9e-01	9.6e-01	1.0e+00
Phospholipids (mmol/l)	NMR delay	9.9e-01	9.5e-01	1.0e+00
Total cholesterol (mmol/l)	Buffer delay	9.8e-01	9.3e-01	1.0e+00
Total cholesterol (mmol/l)	NMR delay	9.7e-01	9.0e-01	9.9e-01
Cholesterol esters (mmol/l)	Buffer delay	9.6e-01	8.8e-01	9.9e-01
Cholesterol esters (mmol/l)	NMR delay	9.7e-01	9.0e-01	9.9e-01
Free cholesterol (mmol/l)	Buffer delay	9.7e-01	8.9e-01	1.0e+00
Free cholesterol (mmol/l)	NMR delay	9.9e-01	9.5e-01	1.0e+00
Triglycerides (mmol/l)	Buffer delay	9.8e-01	9.0e-01	9.9e-01
Triglycerides (mmol/l)	NMR delay	9.8e-01	9.2e-01	1.0e+00
Very large HDL				
Particle concentration (mol/l)	Buffer delay	9.9e-01	9.6e-01	1.0e+00
Particle concentration (mol/l)	NMR delay	9.9e-01	9.4e-01	1.0e+00
Total lipids (mmol/l)	Buffer delay	9.9e-01	9.6e-01	1.0e+00
Total lipids (mmol/l)	NMR delay	9.9e-01	9.3e-01	1.0e+00
Phospholipids (mmol/l)	Buffer delay	9.9e-01	9.6e-01	1.0e+00
Phospholipids (mmol/l)	NMR delay	9.9e-01	9.6e-01	1.0e+00
Total cholesterol (mmol/l)	Buffer delay	9.8e-01	9.2e-01	1.0e+00
Total cholesterol (mmol/l)	NMR delay	9.7e-01	9.0e-01	9.9e-01
Cholesterol esters (mmol/l)	Buffer delay	9.8e-01	9.3e-01	1.0e+00
Cholesterol esters (mmol/l)	NMR delay	9.8e-01	9.2e-01	1.0e+00
Free cholesterol (mmol/l)	Buffer delay	9.9e-01	9.6e-01	1.0e+00
Free cholesterol (mmol/l)	NMR delay	9.9e-01	9.3e-01	1.0e+00
Triglycerides (mmol/l)	Buffer delay	9.5e-01	8.6e-01	9.7e-01
Triglycerides (mmol/l)	NMR delay	9.4e-01	8.2e-01	9.7e-01
Large HDL				

Metabolic traits	conditions	correlation	LCI	UCI
Particle concentration (mol/l)	Buffer delay	9.9e-01	9.6e-01	1.0e+00
Particle concentration (mol/l)	NMR delay	9.9e-01	9.6e-01	1.0e+00
Total lipids (mmol/l)	Buffer delay	9.9e-01	9.6e-01	1.0e+00
Total lipids (mmol/l)	NMR delay	9.9e-01	9.6e-01	1.0e+00
Phospholipids (mmol/l)	Buffer delay	9.9e-01	9.4e-01	1.0e+00
Phospholipids (mmol/l)	NMR delay	9.9e-01	9.4e-01	1.0e+00
Total cholesterol (mmol/l)	Buffer delay	9.9e-01	9.6e-01	1.0e+00
Total cholesterol (mmol/l)	NMR delay	9.9e-01	9.7e-01	1.0e+00
Cholesterol esters (mmol/l)	Buffer delay	1.0e+00	9.8e-01	1.0e+00
Cholesterol esters (mmol/l)	NMR delay	1.0e+00	9.8e-01	1.0e+00
Free cholesterol (mmol/l)	Buffer delay	9.9e-01	9.7e-01	1.0e+00
Free cholesterol (mmol/l)	NMR delay	9.9e-01	9.6e-01	1.0e+00
Triglycerides (mmol/l)	Buffer delay	8.8e-01	6.3e-01	9.9e-01
Triglycerides (mmol/l)	NMR delay	8.8e-01	6.4e-01	9.8e-01
Medium HDL				
Particle concentration (mol/l)	Buffer delay	8.6e-01	6.4e-01	9.6e-01
Particle concentration (mol/l)	NMR delay	8.8e-01	6.5e-01	9.7e-01
Total lipids (mmol/l)	Buffer delay	8.6e-01	6.0e-01	9.5e-01
Total lipids (mmol/l)	NMR delay	8.6e-01	6.5e-01	9.6e-01
Phospholipids (mmol/l)	Buffer delay	9.0e-01	6.7e-01	9.7e-01
Phospholipids (mmol/l)	NMR delay	9.0e-01	7.0e-01	9.7e-01
Total cholesterol (mmol/l)	Buffer delay	8.8e-01	6.1e-01	9.6e-01
Total cholesterol (mmol/l)	NMR delay	8.5e-01	6.0e-01	9.6e-01
Cholesterol esters (mmol/l)	Buffer delay	8.9e-01	6.8e-01	9.7e-01
Cholesterol esters (mmol/l)	NMR delay	8.4e-01	5.8e-01	9.5e-01
Free cholesterol (mmol/l)	Buffer delay	8.6e-01	6.2e-01	9.7e-01
Free cholesterol (mmol/l)	NMR delay	8.6e-01	6.4e-01	9.7e-01
Triglycerides (mmol/l)	Buffer delay	9.5e-01	8.8e-01	9.8e-01
Triglycerides (mmol/l)	NMR delay	9.6e-01	8.7e-01	9.9e-01

Metabolic traits	conditions	correlation	LCI	UCI
Small HDL				
Particle concentration (mol/l)	Buffer delay	8.2e-01	5.9e-01	9.4e-01
Particle concentration (mol/l)	NMR delay	8.3e-01	5.9e-01	9.2e-01
Total lipids (mmol/l)	Buffer delay	8.5e-01	6.1e-01	9.6e-01
Total lipids (mmol/l)	NMR delay	8.5e-01	6.5e-01	9.4e-01
Phospholipids (mmol/l)	Buffer delay	9.8e-01	9.1e-01	1.0e+00
Phospholipids (mmol/l)	NMR delay	9.8e-01	9.1e-01	9.9e-01
Total cholesterol (mmol/l)	Buffer delay	7.3e-01	4.4e-01	9.0e-01
Total cholesterol (mmol/l)	NMR delay	7.0e-01	4.0e-01	8.9e-01
Cholesterol esters (mmol/l)	Buffer delay	8.3e-01	6.0e-01	9.4e-01
Cholesterol esters (mmol/l)	NMR delay	7.3e-01	4.2e-01	8.7e-01
Free cholesterol (mmol/l)	Buffer delay	9.4e-01	8.0e-01	9.9e-01
Free cholesterol (mmol/l)	NMR delay	9.5e-01	8.4e-01	9.8e-01
Triglycerides (mmol/l)	Buffer delay	9.7e-01	9.0e-01	9.9e-01
Triglycerides (mmol/l)	NMR delay	9.6e-01	8.6e-01	9.9e-01
**Lipoprotein particle size**				
VLDL particle size (nm)	Buffer delay	9.9e-01	9.4e-01	1.0e+00
VLDL particle size (nm)	NMR delay	9.7e-01	8.6e-01	1.0e+00
LDL particle size (nm)	Buffer delay	7.8e-01	5.1e-01	9.2e-01
LDL particle size (nm)	NMR delay	7.8e-01	5.6e-01	9.1e-01
HDL particle size (nm)	Buffer delay	9.9e-01	9.5e-01	1.0e+00
HDL particle size (nm)	NMR delay	9.8e-01	9.3e-01	1.0e+00
**Cholesterol**				
Total cholesterol (mmol/l)	Buffer delay	9.8e-01	9.2e-01	1.0e+00
Total cholesterol (mmol/l)	NMR delay	9.8e-01	9.4e-01	1.0e+00
VLDL cholesterol (mmol/l)	Buffer delay	9.5e-01	8.2e-01	9.9e-01
VLDL cholesterol (mmol/l)	NMR delay	9.5e-01	8.3e-01	1.0e+00
Remnant cholesterol (mmol/l)	Buffer delay	9.7e-01	8.7e-01	9.9e-01
Remnant cholesterol (mmol/l)	NMR delay	9.6e-01	8.6e-01	9.9e-01

Metabolic traits	conditions	correlation	LCI	UCI
LDL cholesterol (mmol/l)	Buffer delay	9.9e-01	9.5e-01	1.0e+00
LDL cholesterol (mmol/l)	NMR delay	9.6e-01	8.9e-01	9.9e-01
HDL cholesterol (mmol/l)	Buffer delay	9.8e-01	9.2e-01	1.0e+00
HDL cholesterol (mmol/l)	NMR delay	9.7e-01	9.0e-01	9.9e-01
HDL2 cholesterol (mmol/l)	Buffer delay	9.7e-01	8.9e-01	9.9e-01
HDL2 cholesterol (mmol/l)	NMR delay	9.7e-01	8.8e-01	9.9e-01
HDL3 cholesterol (mmol/l)	Buffer delay	9.5e-01	8.5e-01	9.8e-01
HDL3 cholesterol (mmol/l)	NMR delay	9.5e-01	8.7e-01	9.8e-01
Esterified cholesterol (mmol/l)	Buffer delay	9.8e-01	8.8e-01	1.0e+00
Esterified cholesterol (mmol/l)	NMR delay	9.9e-01	9.4e-01	1.0e+00
Free cholesterol (mmol/l)	Buffer delay	9.7e-01	8.7e-01	1.0e+00
Free cholesterol (mmol/l)	NMR delay	9.6e-01	8.2e-01	9.9e-01
**Glycerides and phospholipids**				
Triglycerides (mmol/l)	Buffer delay	9.9e-01	9.6e-01	1.0e+00
Triglycerides (mmol/l)	NMR delay	9.9e-01	9.2e-01	1.0e+00
VLDL triglycerides (mmol/l)	Buffer delay	9.8e-01	9.0e-01	1.0e+00
VLDL triglycerides (mmol/l)	NMR delay	9.8e-01	9.3e-01	1.0e+00
LDL triglycerides (mmol/l)	Buffer delay	9.7e-01	8.9e-01	9.9e-01
LDL triglycerides (mmol/l)	NMR delay	9.7e-01	9.0e-01	9.9e-01
HDL triglycerides (mmol/l)	Buffer delay	9.9e-01	9.5e-01	1.0e+00
HDL triglycerides (mmol/l)	NMR delay	9.8e-01	9.2e-01	9.9e-01
Diacylglycerol (mmol/l)	Buffer delay	7.2e-01	3.7e-01	8.9e-01
Diacylglycerol (mmol/l)	NMR delay	7.7e-01	4.0e-01	9.4e-01
Phosphoglycerides (mmol/l)	Buffer delay	9.3e-01	8.0e-01	9.7e-01
Phosphoglycerides (mmol/l)	NMR delay	9.4e-01	7.2e-01	1.0e+00
Phosphatidylcholine + other cholines (mmol/l)	Buffer delay	9.1e-01	7.6e-01	9.6e-01
Phosphatidylcholine + other cholines (mmol/l)	NMR delay	9.2e-01	7.4e-01	9.8e-01
Sphingomyelins (mmol/l)	Buffer delay	7.7e-01	3.7e-01	9.3e-01
Sphingomyelins (mmol/l)	NMR delay	8.1e-01	5.3e-01	9.2e-01

Metabolic traits	conditions	correlation	LCI	UCI
Cholines (mmol/l)	Buffer delay	8.3e-01	5.3e-01	9.5e-01
Cholines (mmol/l)	NMR delay	8.6e-01	6.4e-01	9.5e-01
**Apolipoproteins**				
Apolipoprotein A-I (g/l)	Buffer delay	9.7e-01	9.0e-01	1.0e+00
Apolipoprotein A-I (g/l)	NMR delay	9.8e-01	9.1e-01	9.9e-01
Apolipoprotein B (g/l)	Buffer delay	9.7e-01	8.8e-01	9.9e-01
Apolipoprotein B (g/l)	NMR delay	9.5e-01	8.4e-01	9.9e-01
**Fatty acids**				
Total fatty acids (mmol/l)	Buffer delay	9.6e-01	8.5e-01	1.0e+00
Total fatty acids (mmol/l)	NMR delay	9.7e-01	8.9e-01	9.9e-01
Fatty acid chain length	Buffer delay	8.8e-01	6.3e-01	9.8e-01
Fatty acid chain length	NMR delay	5.3e-01	1.2e-01	8.1e-01
Degree of unsaturation	Buffer delay	9.4e-01	7.5e-01	9.9e-01
Degree of unsaturation	NMR delay	8.3e-01	5.5e-01	9.7e-01
Docosahexaenoic acid (mmol/l)	Buffer delay	9.8e-01	9.0e-01	1.0e+00
Docosahexaenoic acid (mmol/l)	NMR delay	9.8e-01	8.8e-01	1.0e+00
Linoleic acid (mmol/l)	Buffer delay	9.8e-01	9.2e-01	1.0e+00
Linoleic acid (mmol/l)	NMR delay	9.5e-01	8.4e-01	9.9e-01
Conjugated linoleic acid (mmol/l)	Buffer delay	7.3e-01	3.7e-01	9.3e-01
Conjugated linoleic acid (mmol/l)	NMR delay	7.8e-01	4.4e-01	9.4e-01
n-3 fatty acids (mmol/l)	Buffer delay	9.7e-01	8.9e-01	9.9e-01
n-3 fatty acids (mmol/l)	NMR delay	9.8e-01	8.9e-01	1.0e+00
n-6 fatty acids (mmol/l)	Buffer delay	9.6e-01	8.7e-01	9.9e-01
n-6 fatty acids (mmol/l)	NMR delay	9.6e-01	8.3e-01	9.9e-01
PUFA (mmol/l)	Buffer delay	9.7e-01	8.7e-01	1.0e+00
PUFA (mmol/l)	NMR delay	9.7e-01	8.6e-01	9.9e-01
MUFA (mmol/l)	Buffer delay	9.8e-01	9.1e-01	1.0e+00
MUFA (mmol/l)	NMR delay	9.6e-01	8.7e-01	9.9e-01
Saturated fatty acids (mmol/l)	Buffer delay	9.8e-01	9.2e-01	1.0e+00

Metabolic traits	conditions	correlation	LCI	UCI
Saturated fatty acids (mmol/l)	NMR delay	9.7e-01	8.9e-01	9.9e-01
**Glycolysis related metabolites**				
Glucose (mmol/l)	Buffer delay	9.7e-01	9.0e-01	1.0e+00
Glucose (mmol/l)	NMR delay	9.7e-01	8.7e-01	9.9e-01
Lactate (mmol/l)	Buffer delay	9.9e-01	9.6e-01	1.0e+00
Lactate (mmol/l)	NMR delay	9.9e-01	9.4e-01	1.0e+00
Citrate (mmol/l)	Buffer delay	8.8e-01	6.9e-01	9.6e-01
Citrate (mmol/l)	NMR delay	8.5e-01	6.4e-01	9.4e-01
**Amino acids**				
Alanine (mmol/l)	Buffer delay	9.7e-01	8.8e-01	9.9e-01
Alanine (mmol/l)	NMR delay	9.6e-01	8.8e-01	9.8e-01
Glutamine (mmol/l)	Buffer delay	8.8e-01	7.2e-01	9.6e-01
Glutamine (mmol/l)	NMR delay	9.6e-01	8.6e-01	9.9e-01
Histidine (mmol/l)	Buffer delay	3.6e-01	-9.9e-02	7.3e-01
Histidine (mmol/l)	NMR delay	5.7e-01	1.5e-01	8.1e-01
Branched-chain amino acids				
Isoleucine (mmol/l)	Buffer delay	9.0e-01	6.9e-01	9.8e-01
Isoleucine (mmol/l)	NMR delay	9.2e-01	7.5e-01	9.9e-01
Leucine (mmol/l)	Buffer delay	9.9e-01	9.5e-01	1.0e+00
Leucine (mmol/l)	NMR delay	9.8e-01	9.0e-01	1.0e+00
Valine (mmol/l)	Buffer delay	9.8e-01	9.1e-01	1.0e+00
Valine (mmol/l)	NMR delay	9.9e-01	9.5e-01	1.0e+00
Aromatic amino acids				
Phenylalanine (mmol/l)	Buffer delay	7.5e-01	4.8e-01	9.2e-01
Phenylalanine (mmol/l)	NMR delay	7.4e-01	4.8e-01	8.9e-01
Tyrosine (mmol/l)	Buffer delay	9.1e-01	7.4e-01	9.8e-01
Tyrosine (mmol/l)	NMR delay	9.6e-01	8.7e-01	9.9e-01
**Ketone bodies**				
Acetate (mmol/l)	Buffer delay	8.3e-01	5.9e-01	9.5e-01

Metabolic traits	conditions	correlation	LCI	UCI
Acetate (mmol/l)	NMR delay	9.0e-01	7.3e-01	9.7e-01
Beta-hydroxybutyrate (mmol/l)	Buffer delay	9.8e-01	9.2e-01	9.9e-01
Beta-hydroxybutyrate (mmol/l)	NMR delay	9.1e-01	7.2e-01	9.7e-01
**Fluid balance**				
Creatinine (mmol/l)	Buffer delay	9.7e-01	9.0e-01	9.9e-01
Creatinine (mmol/l)	NMR delay	9.3e-01	7.8e-01	9.7e-01
Albumin (signal area)	Buffer delay	9.0e-01	7.2e-01	9.7e-01
Albumin (signal area)	NMR delay	8.9e-01	7.5e-01	9.5e-01
**Inflammation**				
Glycoprotein acetyls (mmol/l)	Buffer delay	9.7e-01	8.6e-01	9.9e-01
Glycoprotein acetyls (mmol/l)	NMR delay	9.9e-01	9.6e-01	1.0e+00

Table S6. Serum, pre-storage handling effects (differences in mean levels): mean differences in metabolite concentrations (or trait value) per 24 h increment in incubation duration at 4 °C and 21 °C, for serum samples.

# Associations in Figure S5 are presented in SD-units. These SD point estimates can be obtained by dividing the point estimate (beta) in absolute (clinically meaningful) concentration by the metabolic trait standard deviation (SD), both provided in the below table.

***Abbreviations**: **C**=cholesterol; **IDL**=intermediate-density lipoprotein; **LCI**=lower confidence interval; **LDL**=low-density lipoprotein; **HDL**=high-density lipoprotein; **MUFA**=monounsaturated fatty acids; **N.obs**= number of observations (samples); **N.indiv**=number of individuals; **PUFA**=polyunsaturated fatty acids; **SD**=standard deviation; **UCI**= upper confidence interval; **VLDL**=very-low-density lipoprotein.*

Metabolic traits	temperature	N.obs	N.indiv	Beta	LCI	UCI	Pvalue	SD
**Lipoprotein subclasses**								
Extremely large VLDL								
Particle concentration (mol/l)	4°C	69	23	-1.1e-11	-1.6e-11	-6.1e-12	8.5e-06	2.2e-10
Particle concentration (mol/l)	21°C	69	23	-6.3e-12	-1.7e-11	4.3e-12	2.4e-01	2.2e-10
Total lipids (mmol/l)	4°C	69	23	-2.3e-03	-3.3e-03	-1.3e-03	8.5e-06	4.7e-02
Total lipids (mmol/l)	21°C	69	23	-1.3e-03	-3.5e-03	9.3e-04	2.5e-01	4.7e-02
Phospholipids (mmol/l)	4°C	69	23	-2.7e-04	-4.0e-04	-1.4e-04	3.6e-05	5.8e-03
Phospholipids (mmol/l)	21°C	69	23	-1.8e-04	-4.4e-04	8.5e-05	1.9e-01	5.8e-03
Total cholesterol (mmol/l)	4°C	69	23	-3.4e-04	-4.9e-04	-1.8e-04	1.6e-05	8.8e-03
Total cholesterol (mmol/l)	21°C	69	23	-4.5e-05	-4.2e-04	3.3e-04	8.2e-01	8.8e-03
Cholesterol esters (mmol/l)	4°C	69	23	-1.8e-04	-2.7e-04	-8.9e-05	1.1e-04	5.0e-03
Cholesterol esters (mmol/l)	21°C	69	23	8.4e-05	-1.7e-04	3.4e-04	5.1e-01	5.0e-03
Free cholesterol (mmol/l)	4°C	69	23	-1.6e-04	-2.3e-04	-8.3e-05	3.9e-05	3.8e-03
Free cholesterol (mmol/l)	21°C	69	23	-1.3e-04	-2.7e-04	1.2e-05	7.3e-02	3.8e-03
Triglycerides (mmol/l)	4°C	69	23	-1.7e-03	-2.5e-03	-9.5e-04	8.9e-06	3.2e-02
Triglycerides (mmol/l)	21°C	69	23	-1.1e-03	-2.7e-03	5.3e-04	1.9e-01	3.2e-02
Very large VLDL								
Particle concentration (mol/l)	4°C	69	23	-4.0e-11	-6.3e-11	-1.7e-11	5.2e-04	1.3e-09
Particle concentration (mol/l)	21°C	69	23	-4.0e-11	-8.2e-11	2.8e-12	6.7e-02	1.3e-09
Total lipids (mmol/l)	4°C	69	23	-3.9e-03	-6.1e-03	-1.7e-03	4.2e-04	1.3e-01
Total lipids (mmol/l)	21°C	69	23	-3.6e-03	-7.8e-03	5.3e-04	8.7e-02	1.3e-01

Metabolic traits	temperature	N.obs	N.indiv	Beta	LCI	UCI	Pvalue	SD
Phospholipids (mmol/l)	4°C	69	23	-6.7e-04	-1.0e-03	-3.1e-04	2.5e-04	2.1e-02
Phospholipids (mmol/l)	21°C	69	23	-5.2e-04	-1.2e-03	1.9e-04	1.5e-01	2.1e-02
Total cholesterol (mmol/l)	4°C	69	23	-8.2e-04	-1.2e-03	-4.0e-04	1.0e-04	2.6e-02
Total cholesterol (mmol/l)	21°C	69	23	-1.7e-04	-1.1e-03	7.7e-04	7.2e-01	2.6e-02
Cholesterol esters (mmol/l)	4°C	69	23	-4.3e-04	-6.5e-04	-2.0e-04	2.3e-04	1.4e-02
Cholesterol esters (mmol/l)	21°C	69	23	6.3e-06	-5.3e-04	5.4e-04	9.8e-01	1.4e-02
Free cholesterol (mmol/l)	4°C	69	23	-3.9e-04	-5.8e-04	-2.0e-04	5.2e-05	1.2e-02
Free cholesterol (mmol/l)	21°C	69	23	-1.8e-04	-6.0e-04	2.3e-04	3.9e-01	1.2e-02
Triglycerides (mmol/l)	4°C	69	23	-2.4e-03	-3.9e-03	-1.0e-03	9.1e-04	8.1e-02
Triglycerides (mmol/l)	21°C	69	23	-2.9e-03	-5.5e-03	-3.2e-04	2.8e-02	8.1e-02
Large VLDL								
Particle concentration (mol/l)	4°C	69	23	-1.8e-10	-3.2e-10	-3.8e-11	1.3e-02	7.5e-09
Particle concentration (mol/l)	21°C	69	23	-2.1e-10	-4.3e-10	8.7e-12	6.0e-02	7.5e-09
Total lipids (mmol/l)	4°C	69	23	-1.0e-02	-1.8e-02	-2.3e-03	1.2e-02	4.4e-01
Total lipids (mmol/l)	21°C	69	23	-1.2e-02	-2.5e-02	9.7e-04	7.0e-02	4.4e-01
Phospholipids (mmol/l)	4°C	69	23	-1.8e-03	-3.3e-03	-3.7e-04	1.4e-02	7.9e-02
Phospholipids (mmol/l)	21°C	69	23	-1.9e-03	-4.2e-03	4.3e-04	1.1e-01	7.9e-02
Total cholesterol (mmol/l)	4°C	69	23	-2.3e-03	-3.9e-03	-6.8e-04	5.2e-03	1.0e-01
Total cholesterol (mmol/l)	21°C	69	23	-1.1e-03	-4.1e-03	1.9e-03	4.6e-01	1.0e-01
Cholesterol esters (mmol/l)	4°C	69	23	-1.0e-03	-1.9e-03	-2.1e-04	1.4e-02	5.1e-02
Cholesterol esters (mmol/l)	21°C	69	23	1.9e-04	-1.6e-03	1.9e-03	8.3e-01	5.1e-02
Free cholesterol (mmol/l)	4°C	69	23	-1.2e-03	-2.1e-03	-4.2e-04	2.9e-03	5.0e-02
Free cholesterol (mmol/l)	21°C	69	23	-1.3e-03	-2.6e-03	1.4e-05	5.3e-02	5.0e-02
Triglycerides (mmol/l)	4°C	69	23	-6.2e-03	-1.1e-02	-1.2e-03	1.6e-02	2.6e-01
Triglycerides (mmol/l)	21°C	69	23	-8.8e-03	-1.7e-02	-1.1e-03	2.5e-02	2.6e-01
Medium VLDL								
Particle concentration (mol/l)	4°C	69	23	-3.1e-10	-6.4e-10	2.4e-11	6.9e-02	1.9e-08

Metabolic traits	temperature	N.obs	N.indiv	Beta	LCI	UCI	Pvalue	SD
Particle concentration (mol/l)	21°C	69	23	-2.1e-10	-7.7e-10	3.5e-10	4.6e-01	1.9e-08
Total lipids (mmol/l)	4°C	69	23	-1.0e-02	-2.1e-02	9.1e-04	7.2e-02	6.3e-01
Total lipids (mmol/l)	21°C	69	23	-5.8e-03	-2.4e-02	1.3e-02	5.4e-01	6.3e-01
Phospholipids (mmol/l)	4°C	69	23	-1.8e-03	-3.9e-03	2.4e-04	8.4e-02	1.2e-01
Phospholipids (mmol/l)	21°C	69	23	-5.7e-04	-4.1e-03	3.0e-03	7.6e-01	1.2e-01
Total cholesterol (mmol/l)	4°C	69	23	-1.9e-03	-4.5e-03	7.0e-04	1.5e-01	1.6e-01
Total cholesterol (mmol/l)	21°C	69	23	2.4e-03	-2.5e-03	7.4e-03	3.4e-01	1.6e-01
Cholesterol esters (mmol/l)	4°C	69	23	-6.7e-04	-2.2e-03	8.3e-04	3.8e-01	8.2e-02
Cholesterol esters (mmol/l)	21°C	69	23	3.5e-03	2.0e-04	6.7e-03	3.8e-02	8.2e-02
Free cholesterol (mmol/l)	4°C	69	23	-1.2e-03	-2.5e-03	3.5e-07	5.0e-02	7.7e-02
Free cholesterol (mmol/l)	21°C	69	23	-1.0e-03	-3.0e-03	9.1e-04	3.0e-01	7.7e-02
Triglycerides (mmol/l)	4°C	69	23	-6.3e-03	-1.3e-02	4.6e-05	5.2e-02	3.5e-01
Triglycerides (mmol/l)	21°C	69	23	-7.6e-03	-1.8e-02	2.8e-03	1.5e-01	3.5e-01
Small VLDL								
Particle concentration (mol/l)	4°C	69	23	2.3e-10	-1.3e-10	5.8e-10	2.1e-01	1.9e-08
Particle concentration (mol/l)	21°C	69	23	8.7e-10	3.4e-10	1.4e-09	1.4e-03	1.9e-08
Total lipids (mmol/l)	4°C	69	23	5.4e-03	-1.4e-03	1.2e-02	1.2e-01	3.5e-01
Total lipids (mmol/l)	21°C	69	23	1.9e-02	9.0e-03	3.0e-02	2.4e-04	3.5e-01
Phospholipids (mmol/l)	4°C	69	23	2.1e-03	7.5e-04	3.5e-03	2.5e-03	7.1e-02
Phospholipids (mmol/l)	21°C	69	23	5.3e-03	3.5e-03	7.0e-03	5.6e-09	7.1e-02
Total cholesterol (mmol/l)	4°C	69	23	3.7e-03	1.2e-03	6.2e-03	3.5e-03	1.0e-01
Total cholesterol (mmol/l)	21°C	69	23	1.3e-02	8.0e-03	1.7e-02	5.7e-08	1.0e-01
Cholesterol esters (mmol/l)	4°C	69	23	2.5e-03	7.7e-04	4.3e-03	4.9e-03	5.6e-02
Cholesterol esters (mmol/l)	21°C	69	23	9.6e-03	6.1e-03	1.3e-02	6.7e-08	5.6e-02
Free cholesterol (mmol/l)	4°C	69	23	1.2e-03	3.1e-04	2.0e-03	7.7e-03	4.6e-02
Free cholesterol (mmol/l)	21°C	69	23	2.9e-03	1.8e-03	4.1e-03	8.3e-07	4.6e-02
Triglycerides (mmol/l)	4°C	69	23	-4.2e-04	-3.8e-03	3.0e-03	8.1e-01	1.9e-01

Metabolic traits	temperature	N.obs	N.indiv	Beta	LCI	UCI	Pvalue	SD
Triglycerides (mmol/l)	21°C	69	23	1.4e-03	-3.5e-03	6.3e-03	5.7e-01	1.9e-01
Very Small VLDL								
Particle concentration (mol/l)	4°C	69	23	5.9e-10	3.0e-10	8.8e-10	6.7e-05	1.1e-08
Particle concentration (mol/l)	21°C	69	23	2.1e-09	1.4e-09	2.8e-09	1.4e-09	1.1e-08
Total lipids (mmol/l)	4°C	69	23	7.5e-03	3.7e-03	1.1e-02	1.1e-04	1.3e-01
Total lipids (mmol/l)	21°C	69	23	2.7e-02	1.8e-02	3.6e-02	2.1e-09	1.3e-01
Phospholipids (mmol/l)	4°C	69	23	2.3e-03	9.8e-04	3.5e-03	5.6e-04	3.3e-02
Phospholipids (mmol/l)	21°C	69	23	8.3e-03	6.0e-03	1.1e-02	7.7e-13	3.3e-02
Total cholesterol (mmol/l)	4°C	69	23	3.5e-03	1.1e-03	5.8e-03	4.0e-03	5.1e-02
Total cholesterol (mmol/l)	21°C	69	23	1.4e-02	8.3e-03	1.9e-02	5.3e-07	5.1e-02
Cholesterol esters (mmol/l)	4°C	69	23	2.0e-03	1.7e-04	3.8e-03	3.2e-02	3.6e-02
Cholesterol esters (mmol/l)	21°C	69	23	9.7e-03	5.5e-03	1.4e-02	7.1e-06	3.6e-02
Free cholesterol (mmol/l)	4°C	69	23	1.5e-03	8.0e-04	2.2e-03	1.8e-05	1.6e-02
Free cholesterol (mmol/l)	21°C	69	23	3.9e-03	2.7e-03	5.1e-03	1.1e-10	1.6e-02
Triglycerides (mmol/l)	4°C	69	23	1.8e-03	9.3e-04	2.7e-03	4.8e-05	5.4e-02
Triglycerides (mmol/l)	21°C	69	23	5.1e-03	3.3e-03	6.8e-03	1.1e-08	5.4e-02
IDL								
Particle concentration (mol/l)	4°C	69	23	4.9e-10	-3.5e-10	1.3e-09	2.6e-01	2.1e-08
Particle concentration (mol/l)	21°C	69	23	3.5e-09	1.8e-09	5.2e-09	6.8e-05	2.1e-08
Total lipids (mmol/l)	4°C	69	23	4.3e-03	-4.5e-03	1.3e-02	3.4e-01	2.1e-01
Total lipids (mmol/l)	21°C	69	23	3.4e-02	1.6e-02	5.1e-02	1.5e-04	2.1e-01
Phospholipids (mmol/l)	4°C	69	23	1.6e-03	-5.3e-04	3.8e-03	1.4e-01	4.9e-02
Phospholipids (mmol/l)	21°C	69	23	9.2e-03	5.2e-03	1.3e-02	7.7e-06	4.9e-02
Total cholesterol (mmol/l)	4°C	69	23	6.1e-04	-5.7e-03	6.9e-03	8.5e-01	1.3e-01
Total cholesterol (mmol/l)	21°C	69	23	1.8e-02	6.1e-03	3.0e-02	3.0e-03	1.3e-01
Cholesterol esters (mmol/l)	4°C	69	23	-2.3e-04	-4.9e-03	4.4e-03	9.2e-01	1.0e-01
Cholesterol esters (mmol/l)	21°C	69	23	1.2e-02	3.6e-03	2.1e-02	5.8e-03	1.0e-01

Metabolic traits	temperature	N.obs	N.indiv	Beta	LCI	UCI	Pvalue	SD
Free cholesterol (mmol/l)	4°C	69	23	8.5e-04	-8.7e-04	2.6e-03	3.3e-01	3.8e-02
Free cholesterol (mmol/l)	21°C	69	23	5.5e-03	2.3e-03	8.8e-03	8.7e-04	3.8e-02
Triglycerides (mmol/l)	4°C	69	23	2.0e-03	1.3e-03	2.8e-03	4.1e-08	3.9e-02
Triglycerides (mmol/l)	21°C	69	23	6.5e-03	4.2e-03	8.9e-03	4.1e-08	3.9e-02
Large LDL								
Particle concentration (mol/l)	4°C	69	23	9.6e-10	-4.0e-10	2.3e-09	1.7e-01	3.5e-08
Particle concentration (mol/l)	21°C	69	23	5.1e-09	2.5e-09	7.7e-09	1.2e-04	3.5e-08
Total lipids (mmol/l)	4°C	69	23	6.2e-03	-3.5e-03	1.6e-02	2.1e-01	2.4e-01
Total lipids (mmol/l)	21°C	69	23	3.5e-02	1.7e-02	5.4e-02	2.0e-04	2.4e-01
Phospholipids (mmol/l)	4°C	69	23	1.2e-03	-8.2e-04	3.2e-03	2.4e-01	5.1e-02
Phospholipids (mmol/l)	21°C	69	23	7.1e-03	3.6e-03	1.1e-02	7.3e-05	5.1e-02
Total cholesterol (mmol/l)	4°C	69	23	3.0e-03	-4.3e-03	1.0e-02	4.2e-01	1.7e-01
Total cholesterol (mmol/l)	21°C	69	23	2.2e-02	8.9e-03	3.6e-02	1.1e-03	1.7e-01
Cholesterol esters (mmol/l)	4°C	69	23	2.1e-03	-3.4e-03	7.6e-03	4.5e-01	1.4e-01
Cholesterol esters (mmol/l)	21°C	69	23	1.7e-02	6.7e-03	2.6e-02	9.9e-04	1.4e-01
Free cholesterol (mmol/l)	4°C	69	23	9.2e-04	-9.0e-04	2.7e-03	3.2e-01	4.2e-02
Free cholesterol (mmol/l)	21°C	69	23	5.8e-03	2.1e-03	9.4e-03	1.8e-03	4.2e-02
Triglycerides (mmol/l)	4°C	69	23	2.0e-03	1.2e-03	2.7e-03	9.4e-08	3.1e-02
Triglycerides (mmol/l)	21°C	69	23	5.8e-03	3.4e-03	8.2e-03	1.8e-06	3.1e-02
Medium LDL								
Particle concentration (mol/l)	4°C	69	23	1.5e-09	2.8e-10	2.7e-09	1.6e-02	3.0e-08
Particle concentration (mol/l)	21°C	69	23	5.1e-09	2.8e-09	7.4e-09	1.0e-05	3.0e-08
Total lipids (mmol/l)	4°C	69	23	7.5e-03	1.2e-03	1.4e-02	1.9e-02	1.5e-01
Total lipids (mmol/l)	21°C	69	23	2.5e-02	1.4e-02	3.7e-02	1.4e-05	1.5e-01
Phospholipids (mmol/l)	4°C	69	23	1.5e-03	2.8e-04	2.7e-03	1.6e-02	3.7e-02
Phospholipids (mmol/l)	21°C	69	23	5.3e-03	3.2e-03	7.4e-03	7.5e-07	3.7e-02
Total cholesterol (mmol/l)	4°C	69	23	4.9e-03	1.0e-04	9.8e-03	4.5e-02	1.1e-01

Metabolic traits	temperature	N.obs	N.indiv	Beta	LCI	UCI	Pvalue	SD
Total cholesterol (mmol/l)	21°C	69	23	1.7e-02	8.6e-03	2.6e-02	7.4e-05	1.1e-01
Cholesterol esters (mmol/l)	4°C	69	23	3.5e-03	-4.2e-04	7.3e-03	8.0e-02	8.9e-02
Cholesterol esters (mmol/l)	21°C	69	23	1.3e-02	6.3e-03	2.0e-02	1.2e-04	8.9e-02
Free cholesterol (mmol/l)	4°C	69	23	1.5e-03	5.1e-04	2.5e-03	2.9e-03	2.2e-02
Free cholesterol (mmol/l)	21°C	69	23	4.2e-03	2.2e-03	6.1e-03	2.7e-05	2.2e-02
Triglycerides (mmol/l)	4°C	69	23	1.0e-03	6.0e-04	1.5e-03	3.0e-06	1.6e-02
Triglycerides (mmol/l)	21°C	69	23	2.9e-03	1.6e-03	4.3e-03	1.6e-05	1.6e-02
Small LDL								
Particle concentration (mol/l)	4°C	69	23	2.3e-09	8.3e-10	3.7e-09	2.1e-03	3.5e-08
Particle concentration (mol/l)	21°C	69	23	6.3e-09	3.7e-09	8.9e-09	2.4e-06	3.5e-08
Total lipids (mmol/l)	4°C	69	23	6.5e-03	2.3e-03	1.1e-02	2.2e-03	9.8e-02
Total lipids (mmol/l)	21°C	69	23	1.8e-02	1.0e-02	2.5e-02	3.2e-06	9.8e-02
Phospholipids (mmol/l)	4°C	69	23	1.9e-03	9.1e-04	2.8e-03	1.3e-04	2.6e-02
Phospholipids (mmol/l)	21°C	69	23	4.4e-03	2.7e-03	6.1e-03	2.2e-07	2.6e-02
Total cholesterol (mmol/l)	4°C	69	23	4.1e-03	1.0e-03	7.1e-03	9.4e-03	6.8e-02
Total cholesterol (mmol/l)	21°C	69	23	1.2e-02	6.5e-03	1.7e-02	1.8e-05	6.8e-02
Cholesterol esters (mmol/l)	4°C	69	23	2.7e-03	2.8e-04	5.1e-03	2.8e-02	5.4e-02
Cholesterol esters (mmol/l)	21°C	69	23	8.7e-03	4.5e-03	1.3e-02	3.9e-05	5.4e-02
Free cholesterol (mmol/l)	4°C	69	23	1.4e-03	6.9e-04	2.1e-03	9.2e-05	1.4e-02
Free cholesterol (mmol/l)	21°C	69	23	3.2e-03	1.8e-03	4.6e-03	7.0e-06	1.4e-02
Triglycerides (mmol/l)	4°C	69	23	5.4e-04	2.7e-04	8.1e-04	1.0e-04	1.4e-02
Triglycerides (mmol/l)	21°C	69	23	1.5e-03	8.8e-04	2.0e-03	9.1e-07	1.4e-02
Very large HDL								
Particle concentration (mol/l)	4°C	69	23	-1.1e-08	-1.6e-08	-6.7e-09	1.3e-06	2.3e-07
Particle concentration (mol/l)	21°C	69	23	-1.0e-08	-2.4e-08	4.0e-09	1.6e-01	2.3e-07
Total lipids (mmol/l)	4°C	69	23	-1.1e-02	-1.6e-02	-6.7e-03	1.5e-06	2.3e-01
Total lipids (mmol/l)	21°C	69	23	-1.0e-02	-2.4e-02	4.1e-03	1.6e-01	2.3e-01

Metabolic traits	temperature	N.obs	N.indiv	Beta	LCI	UCI	Pvalue	SD
Phospholipids (mmol/l)	4°C	69	23	-5.3e-03	-7.6e-03	-3.0e-03	6.3e-06	1.4e-01
Phospholipids (mmol/l)	21°C	69	23	-5.1e-03	-1.2e-02	1.8e-03	1.5e-01	1.4e-01
Total cholesterol (mmol/l)	4°C	69	23	-5.2e-03	-7.5e-03	-2.9e-03	1.2e-05	9.7e-02
Total cholesterol (mmol/l)	21°C	69	23	-4.3e-03	-1.2e-02	3.1e-03	2.5e-01	9.7e-02
Cholesterol esters (mmol/l)	4°C	69	23	-3.6e-03	-5.3e-03	-1.9e-03	2.8e-05	6.8e-02
Cholesterol esters (mmol/l)	21°C	69	23	-2.7e-03	-8.2e-03	2.9e-03	3.5e-01	6.8e-02
Free cholesterol (mmol/l)	4°C	69	23	-1.6e-03	-2.3e-03	-9.2e-04	3.6e-06	3.0e-02
Free cholesterol (mmol/l)	21°C	69	23	-1.7e-03	-3.6e-03	2.3e-04	8.5e-02	3.0e-02
Triglycerides (mmol/l)	4°C	69	23	-7.6e-04	-1.1e-03	-4.5e-04	1.1e-06	1.1e-02
Triglycerides (mmol/l)	21°C	69	23	-7.8e-04	-1.5e-03	-8.7e-05	2.7e-02	1.1e-02
Large HDL								
Particle concentration (mol/l)	4°C	69	23	-1.6e-08	-3.4e-08	2.3e-09	8.6e-02	6.7e-07
Particle concentration (mol/l)	21°C	69	23	-4.4e-08	-8.7e-08	-9.4e-10	4.5e-02	6.7e-07
Total lipids (mmol/l)	4°C	69	23	-9.8e-03	-2.1e-02	1.4e-03	8.7e-02	4.3e-01
Total lipids (mmol/l)	21°C	69	23	-2.7e-02	-5.5e-02	-3.8e-04	4.7e-02	4.3e-01
Phospholipids (mmol/l)	4°C	69	23	-2.1e-03	-7.6e-03	3.4e-03	4.5e-01	1.9e-01
Phospholipids (mmol/l)	21°C	69	23	-9.7e-03	-2.3e-02	3.5e-03	1.5e-01	1.9e-01
Total cholesterol (mmol/l)	4°C	69	23	-5.8e-03	-1.1e-02	-7.6e-04	2.4e-02	2.3e-01
Total cholesterol (mmol/l)	21°C	69	23	-1.5e-02	-2.8e-02	-2.1e-03	2.3e-02	2.3e-01
Cholesterol esters (mmol/l)	4°C	69	23	-5.1e-03	-9.2e-03	-1.1e-03	1.3e-02	1.7e-01
Cholesterol esters (mmol/l)	21°C	69	23	-1.2e-02	-2.2e-02	-2.5e-03	1.4e-02	1.7e-01
Free cholesterol (mmol/l)	4°C	69	23	-6.4e-04	-1.7e-03	4.5e-04	2.5e-01	5.4e-02
Free cholesterol (mmol/l)	21°C	69	23	-2.8e-03	-6.2e-03	4.7e-04	9.3e-02	5.4e-02
Triglycerides (mmol/l)	4°C	69	23	-1.8e-03	-3.2e-03	-4.3e-04	1.0e-02	1.4e-02
Triglycerides (mmol/l)	21°C	69	23	-2.5e-03	-4.2e-03	-7.6e-04	4.9e-03	1.4e-02
Medium HDL								
Particle concentration (mol/l)	4°C	69	23	3.0e-08	9.6e-09	5.0e-08	3.8e-03	4.4e-07

Metabolic traits	temperature	N.obs	N.indiv	Beta	LCI	UCI	Pvalue	SD
Particle concentration (mol/l)	21°C	69	23	-1.9e-08	-7.7e-08	3.8e-08	5.1e-01	4.4e-07
Total lipids (mmol/l)	4°C	69	23	1.3e-02	3.9e-03	2.2e-02	4.6e-03	1.9e-01
Total lipids (mmol/l)	21°C	69	23	-8.6e-03	-3.4e-02	1.6e-02	5.0e-01	1.9e-01
Phospholipids (mmol/l)	4°C	69	23	6.0e-03	2.1e-03	9.8e-03	2.4e-03	8.6e-02
Phospholipids (mmol/l)	21°C	69	23	-3.8e-03	-1.5e-02	7.4e-03	5.1e-01	8.6e-02
Total cholesterol (mmol/l)	4°C	69	23	6.3e-03	1.3e-03	1.1e-02	1.3e-02	1.1e-01
Total cholesterol (mmol/l)	21°C	69	23	-5.3e-03	-1.9e-02	8.7e-03	4.6e-01	1.1e-01
Cholesterol esters (mmol/l)	4°C	69	23	4.6e-03	6.6e-04	8.5e-03	2.2e-02	8.8e-02
Cholesterol esters (mmol/l)	21°C	69	23	-4.6e-03	-1.6e-02	6.4e-03	4.1e-01	8.8e-02
Free cholesterol (mmol/l)	4°C	69	23	1.7e-03	6.6e-04	2.8e-03	1.5e-03	2.4e-02
Free cholesterol (mmol/l)	21°C	69	23	-6.8e-04	-3.7e-03	2.3e-03	6.6e-01	2.4e-02
Triglycerides (mmol/l)	4°C	69	23	5.9e-04	1.6e-04	1.0e-03	7.0e-03	2.0e-02
Triglycerides (mmol/l)	21°C	69	23	4.7e-04	-1.0e-04	1.0e-03	1.1e-01	2.0e-02
Small HDL								
Particle concentration (mol/l)	4°C	69	23	9.0e-08	5.7e-08	1.2e-07	7.1e-08	5.6e-07
Particle concentration (mol/l)	21°C	69	23	1.2e-08	-7.7e-08	1.0e-07	7.9e-01	5.6e-07
Total lipids (mmol/l)	4°C	69	23	2.0e-02	1.3e-02	2.8e-02	9.0e-08	1.2e-01
Total lipids (mmol/l)	21°C	69	23	2.9e-03	-1.7e-02	2.3e-02	7.8e-01	1.2e-01
Phospholipids (mmol/l)	4°C	69	23	8.9e-03	5.3e-03	1.2e-02	1.2e-06	7.6e-02
Phospholipids (mmol/l)	21°C	69	23	-7.5e-03	-1.9e-02	3.5e-03	1.8e-01	7.6e-02
Total cholesterol (mmol/l)	4°C	69	23	1.1e-02	6.0e-03	1.5e-02	6.5e-06	7.0e-02
Total cholesterol (mmol/l)	21°C	69	23	8.9e-03	-1.4e-03	1.9e-02	9.1e-02	7.0e-02
Cholesterol esters (mmol/l)	4°C	69	23	8.4e-03	4.3e-03	1.3e-02	6.7e-05	6.5e-02
Cholesterol esters (mmol/l)	21°C	69	23	9.6e-03	1.2e-03	1.8e-02	2.6e-02	6.5e-02
Free cholesterol (mmol/l)	4°C	69	23	2.1e-03	1.4e-03	2.9e-03	3.7e-08	1.4e-02
Free cholesterol (mmol/l)	21°C	69	23	-6.2e-04	-2.9e-03	1.7e-03	6.0e-01	1.4e-02
Triglycerides (mmol/l)	4°C	69	23	8.5e-04	4.6e-04	1.2e-03	2.1e-05	2.4e-02

Metabolic traits	temperature	N.obs	N.indiv	Beta	LCI	UCI	Pvalue	SD
Triglycerides (mmol/l)	21°C	69	23	1.5e-03	8.5e-04	2.2e-03	6.4e-06	2.4e-02
**Lipoprotein particle size**								
VLDL particle size (nm)	4°C	69	23	-1.2e-01	-1.6e-01	-7.6e-02	8.5e-09	1.6e+00
VLDL particle size (nm)	21°C	69	23	-2.0e-01	-2.8e-01	-1.1e-01	8.0e-06	1.6e+00
LDL particle size (nm)	4°C	69	23	-1.3e-02	-2.0e-02	-6.0e-03	2.5e-04	8.5e-02
LDL particle size (nm)	21°C	69	23	-9.6e-03	-2.4e-02	4.4e-03	1.8e-01	8.5e-02
HDL particle size (nm)	4°C	69	23	-1.9e-02	-2.4e-02	-1.3e-02	4.9e-11	2.8e-01
HDL particle size (nm)	21°C	69	23	-1.9e-02	-3.4e-02	-5.1e-03	7.9e-03	2.8e-01
**Cholesterol**								
Total cholesterol (mmol/l)	4°C	69	23	2.1e-02	-7.4e-03	4.9e-02	1.5e-01	7.2e-01
Total cholesterol (mmol/l)	21°C	69	23	8.1e-02	3.2e-02	1.3e-01	1.2e-03	7.2e-01
VLDL cholesterol (mmol/l)	4°C	69	23	1.9e-03	-5.6e-03	9.4e-03	6.1e-01	4.3e-01
VLDL cholesterol (mmol/l)	21°C	69	23	2.8e-02	1.1e-02	4.4e-02	1.3e-03	4.3e-01
Remnant cholesterol (mmol/l)	4°C	69	23	2.6e-03	-9.4e-03	1.5e-02	6.8e-01	5.0e-01
Remnant cholesterol (mmol/l)	21°C	69	23	4.5e-02	1.9e-02	7.2e-02	8.6e-04	5.0e-01
LDL cholesterol (mmol/l)	4°C	69	23	1.2e-02	-3.1e-03	2.7e-02	1.2e-01	3.5e-01
LDL cholesterol (mmol/l)	21°C	69	23	5.1e-02	2.4e-02	7.8e-02	1.8e-04	3.5e-01
HDL cholesterol (mmol/l)	4°C	69	23	6.2e-03	-5.0e-03	1.7e-02	2.8e-01	4.2e-01
HDL cholesterol (mmol/l)	21°C	69	23	-1.6e-02	-5.2e-02	2.0e-02	3.9e-01	4.2e-01
HDL2 cholesterol (mmol/l)	4°C	69	23	2.8e-03	-7.2e-03	1.3e-02	5.9e-01	3.9e-01
HDL2 cholesterol (mmol/l)	21°C	69	23	-2.0e-02	-5.2e-02	1.2e-02	2.1e-01	3.9e-01
HDL3 cholesterol (mmol/l)	4°C	69	23	3.3e-03	1.6e-03	5.1e-03	2.3e-04	3.4e-02
HDL3 cholesterol (mmol/l)	21°C	69	23	4.4e-03	-1.6e-03	1.0e-02	1.5e-01	3.4e-02
Esterified cholesterol (mmol/l)	4°C	60	20	3.5e-02	8.9e-03	6.0e-02	8.2e-03	5.0e-01
Esterified cholesterol (mmol/l)	21°C	60	20	1.5e-01	1.1e-01	1.9e-01	8.6e-12	5.0e-01
Free cholesterol (mmol/l)	4°C	60	20	-2.2e-02	-3.7e-02	-6.6e-03	5.1e-03	2.2e-01
Free cholesterol (mmol/l)	21°C	60	20	-8.3e-02	-9.6e-02	-7.0e-02	< 0.1e-26	2.2e-01

Metabolic traits	temperature	N.obs	N.indiv	Beta	LCI	UCI	Pvalue	SD
**Glycerides and phospholipids**								
Triglycerides (mmol/l)	4°C	69	23	-8.9e-03	-2.7e-02	8.9e-03	3.3e-01	1.1e+00
Triglycerides (mmol/l)	21°C	69	23	4.1e-03	-2.0e-02	2.8e-02	7.4e-01	1.1e+00
VLDL triglycerides (mmol/l)	4°C	69	23	-1.4e-02	-3.1e-02	2.3e-03	9.2e-02	9.5e-01
VLDL triglycerides (mmol/l)	21°C	69	23	-1.2e-02	-3.8e-02	1.4e-02	3.7e-01	9.5e-01
LDL triglycerides (mmol/l)	4°C	69	23	3.5e-03	2.2e-03	4.9e-03	5.0e-07	6.1e-02
LDL triglycerides (mmol/l)	21°C	69	23	1.0e-02	5.9e-03	1.4e-02	2.8e-06	6.1e-02
HDL triglycerides (mmol/l)	4°C	69	23	-1.8e-04	-1.1e-03	7.3e-04	7.0e-01	5.7e-02
HDL triglycerides (mmol/l)	21°C	69	23	-5.9e-04	-2.6e-03	1.4e-03	5.7e-01	5.7e-02
Diacylglycerol (mmol/l)	4°C	57	19	5.9e-04	-3.7e-03	4.9e-03	7.9e-01	3.0e-02
Diacylglycerol (mmol/l)	21°C	57	19	2.4e-03	-1.7e-03	6.6e-03	2.5e-01	3.0e-02
Phosphoglycerides (mmol/l)	4°C	60	20	-5.1e-03	-2.9e-02	1.9e-02	6.7e-01	3.7e-01
Phosphoglycerides (mmol/l)	21°C	60	20	-6.3e-02	-9.6e-02	-2.9e-02	2.4e-04	3.7e-01
Phosphatidylcholine + other cholines (mmol/l)	4°C	60	20	-1.3e-02	-3.3e-02	7.0e-03	2.0e-01	3.5e-01
Phosphatidylcholine + other cholines (mmol/l)	21°C	60	20	-6.0e-02	-8.8e-02	-3.2e-02	3.2e-05	3.5e-01
Sphingomyelins (mmol/l)	4°C	60	20	2.4e-03	-8.8e-03	1.4e-02	6.7e-01	6.2e-02
Sphingomyelins (mmol/l)	21°C	60	20	3.3e-03	-7.9e-03	1.5e-02	5.6e-01	6.2e-02
Cholines (mmol/l)	4°C	60	20	5.4e-03	-2.8e-02	3.9e-02	7.5e-01	3.7e-01
Cholines (mmol/l)	21°C	60	20	-4.4e-02	-9.3e-02	3.7e-03	7.0e-02	3.7e-01
**Apolipoproteins**								
Apolipoprotein A-I (g/l)	4°C	69	23	2.5e-03	-4.3e-03	9.4e-03	4.6e-01	2.0e-01
Apolipoprotein A-I (g/l)	21°C	69	23	-9.3e-03	-2.8e-02	9.8e-03	3.4e-01	2.0e-01
Apolipoprotein B (g/l)	4°C	69	23	9.5e-04	-5.4e-03	7.3e-03	7.7e-01	2.6e-01
Apolipoprotein B (g/l)	21°C	69	23	1.9e-02	6.0e-03	3.1e-02	3.9e-03	2.6e-01
**Fatty acids**								
Total fatty acids (mmol/l)	4°C	60	20	-1.4e-03	-1.2e-01	1.2e-01	9.8e-01	3.6e+00

metabolites MDPI

Metabolic traits	temperature	N.obs	N.indiv	Beta	LCI	UCI	Pvalue	SD
Total fatty acids (mmol/l)	21°C	60	20	4.5e-02	-1.2e-01	2.1e-01	5.9e-01	3.6e+00
Fatty acid chain length	4°C	60	20	1.1e-02	-2.2e-02	4.5e-02	5.1e-01	2.9e-01
Fatty acid chain length	21°C	60	20	1.5e-03	-3.7e-02	4.0e-02	9.4e-01	2.9e-01
Degree of unsaturation	4°C	60	20	1.0e-02	-1.8e-03	2.2e-02	9.6e-02	8.7e-02
Degree of unsaturation	21°C	60	20	8.6e-03	2.9e-03	1.4e-02	3.0e-03	8.7e-02
Docosahexaenoic acid (mmol/l)	4°C	60	20	2.1e-03	-1.1e-03	5.2e-03	1.9e-01	4.8e-02
Docosahexaenoic acid (mmol/l)	21°C	60	20	3.3e-03	5.6e-04	6.0e-03	1.8e-02	4.8e-02
Linoleic acid (mmol/l)	4°C	60	20	5.9e-03	-1.9e-02	3.1e-02	6.4e-01	6.4e-01
Linoleic acid (mmol/l)	21°C	60	20	3.1e-02	-6.0e-03	6.8e-02	1.0e-01	6.4e-01
Conjugated linoleic acid (mmol/l)	4°C	60	20	-2.8e-03	-6.0e-03	3.9e-04	8.5e-02	2.4e-02
Conjugated linoleic acid (mmol/l)	21°C	60	20	-2.1e-03	-4.8e-03	5.6e-04	1.2e-01	2.4e-02
n-3 fatty acids (mmol/l)	4°C	60	20	4.5e-03	-3.5e-03	1.2e-02	2.7e-01	1.5e-01
n-3 fatty acids (mmol/l)	21°C	60	20	9.8e-03	2.6e-03	1.7e-02	7.8e-03	1.5e-01
n-6 fatty acids (mmol/l)	4°C	60	20	1.2e-02	-2.2e-02	4.5e-02	5.0e-01	7.4e-01
n-6 fatty acids (mmol/l)	21°C	60	20	3.7e-02	-1.1e-02	8.4e-02	1.3e-01	7.4e-01
PUFA (mmol/l)	4°C	60	20	1.6e-02	-2.3e-02	5.5e-02	4.2e-01	8.7e-01
PUFA (mmol/l)	21°C	60	20	4.6e-02	-7.0e-03	1.0e-01	8.9e-02	8.7e-01
MUFA (mmol/l)	4°C	60	20	-1.8e-02	-5.2e-02	1.7e-02	3.3e-01	1.5e+00
MUFA (mmol/l)	21°C	60	20	1.1e-03	-4.8e-02	5.0e-02	9.6e-01	1.5e+00
Saturated fatty acids (mmol/l)	4°C	60	20	-7.5e-05	-5.2e-02	5.2e-02	1.0e+00	1.3e+00
Saturated fatty acids (mmol/l)	21°C	60	20	-2.0e-03	-7.2e-02	6.8e-02	9.6e-01	1.3e+00
**Glycolysis related metabolites**								
Glucose (mmol/l)	4°C	69	23	-9.1e-01	-9.5e-01	-8.7e-01	< 0.1e-26	1.4e+00
Glucose (mmol/l)	21°C	69	23	-1.9e+00	-2.1e+00	-1.7e+00	< 0.1e-26	1.4e+00
Lactate (mmol/l)	4°C	69	23	1.6e+00	1.5e+00	1.7e+00	< 0.1e-26	2.6e+00
Lactate (mmol/l)	21°C	69	23	3.7e+00	3.4e+00	3.9e+00	< 0.1e-26	2.6e+00

Metabolic traits	temperature	N.obs	N.indiv	Beta	LCI	UCI	Pvalue	SD
Pyruvate (mmol/l)	4°C	60	20	-1.3e-02	-1.8e-02	-7.9e-03	6.0e-07	6.0e-01
Pyruvate (mmol/l)	21°C	60	20	7.3e-01	6.4e-01	8.2e-01	< 0.1e-26	6.0e-01
Citrate (mmol/l)	4°C	69	23	4.9e-03	1.8e-03	8.0e-03	2.2e-03	2.0e-02
Citrate (mmol/l)	21°C	69	23	1.9e-03	-9.8e-04	4.8e-03	1.9e-01	2.0e-02
Glycerol (mmol/l)	4°C	69	23	9.1e-03	7.0e-03	1.1e-02	< 0.1e-26	1.9e-02
Glycerol (mmol/l)	21°C	69	23	2.7e-03	-4.3e-04	5.7e-03	9.1e-02	1.9e-02
**Amino acids**								
Alanine (mmol/l)	4°C	69	23	3.1e-02	2.8e-02	3.3e-02	< 0.1e-26	1.2e-01
Alanine (mmol/l)	21°C	69	23	1.5e-01	1.4e-01	1.6e-01	< 0.1e-26	1.2e-01
Glutamine (mmol/l)	4°C	69	23	-7.5e-03	-1.0e-02	-4.7e-03	1.0e-07	6.5e-02
Glutamine (mmol/l)	21°C	69	23	-4.6e-02	-5.1e-02	-4.1e-02	< 0.1e-26	6.5e-02
Histidine (mmol/l)	4°C	69	23	6.8e-03	5.7e-03	7.9e-03	< 0.1e-26	1.4e-02
Histidine (mmol/l)	21°C	69	23	1.7e-02	1.5e-02	1.8e-02	< 0.1e-26	1.4e-02
Glycine (mmol/l)	4°C	69	23	4.0e-02	3.7e-02	4.2e-02	< 0.1e-26	8.6e-02
Glycine (mmol/l)	21°C	69	23	8.1e-02	7.5e-02	8.6e-02	< 0.1e-26	8.6e-02
Branched-chain amino acids								
Isoleucine (mmol/l)	4°C	69	23	5.0e-03	4.0e-03	6.0e-03	< 0.1e-26	2.9e-02
Isoleucine (mmol/l)	21°C	69	23	1.1e-02	9.3e-03	1.3e-02	< 0.1e-26	2.9e-02
Leucine (mmol/l)	4°C	69	23	9.4e-03	8.7e-03	1.0e-02	< 0.1e-26	2.7e-02
Leucine (mmol/l)	21°C	69	23	2.0e-02	1.9e-02	2.2e-02	< 0.1e-26	2.7e-02
Valine (mmol/l)	4°C	69	23	1.3e-02	1.2e-02	1.4e-02	< 0.1e-26	4.1e-02
Valine (mmol/l)	21°C	69	23	2.8e-02	2.6e-02	3.0e-02	< 0.1e-26	4.1e-02
Aromatic amino acids								
Phenylalanine (mmol/l)	4°C	69	23	1.1e-02	9.4e-03	1.2e-02	< 0.1e-26	1.5e-02
Phenylalanine (mmol/l)	21°C	69	23	1.8e-02	1.6e-02	2.0e-02	< 0.1e-26	1.5e-02
Tyrosine (mmol/l)	4°C	69	23	3.4e-03	2.8e-03	4.0e-03	< 0.1e-26	1.3e-02
Tyrosine (mmol/l)	21°C	69	23	7.3e-03	6.5e-03	8.1e-03	< 0.1e-26	1.3e-02

Metabolic traits	temperature	N.obs	N.indiv	Beta	LCI	UCI	Pvalue	SD
**Ketone bodies**								
Acetate (mmol/l)	4°C	69	23	9.3e-03	8.0e-03	1.1e-02	< 0.1e-26	1.2e-02
Acetate (mmol/l)	21°C	69	23	8.7e-03	6.0e-03	1.1e-02	4.8e-10	1.2e-02
Beta-hydroxybutyrate (mmol/l)	4°C	69	23	1.9e-04	-1.2e-03	1.6e-03	7.8e-01	2.0e-02
Beta-hydroxybutyrate (mmol/l)	21°C	69	23	3.0e-03	-7.9e-05	6.0e-03	5.6e-02	2.0e-02
**Fluid balance**								
Creatinine (mmol/l)	4°C	69	23	4.9e-04	-1.7e-04	1.1e-03	1.4e-01	1.0e-02
Creatinine (mmol/l)	21°C	69	23	3.3e-04	-4.9e-04	1.1e-03	4.3e-01	1.0e-02
Albumin (signal area)	4°C	69	23	6.8e-04	3.8e-04	9.9e-04	1.2e-05	4.6e-03
Albumin (signal area)	21°C	69	23	2.6e-03	2.1e-03	3.1e-03	< 0.1e-26	4.6e-03
**Inflammation**								
Glycoprotein acetyls (mmol/l)	4°C	69	23	7.1e-03	2.4e-03	1.2e-02	3.2e-03	3.4e-01
Glycoprotein acetyls (mmol/l)	21°C	69	23	3.2e-02	2.6e-02	3.9e-02	< 0.1e-26	3.4e-01

Table S7. EDTA-Plasma, pre-storage handling effects (differences in mean levels): mean differences in metabolite concentrations (or trait value) per 24 h increment in incubation duration at 4 °C and 21 °C, for EDTA-samples samples. Pyruvate, glycerol and glycine are not quantified in EDTA-plasma samples due to the interfering resonances of EDTA on their signals.

# Associations in Figure S5 are presented in SD-units. These SD point estimate can be obtained by dividing the point estimate in absolute (clinically meaningful) concentration by the metabolic trait standard deviation (SD), both provided in the below table.

***Abbreviations**: **C**=cholesterol; **IDL**=intermediate-density lipoprotein; **LCI**=lower confidence interval; **LDL**=low-density lipoprotein; **HDL**=high-density lipoprotein; **MUFA**=monounsaturated fatty acids; **N.obs**= number of observations (samples); **N.indiv**=number of individuals; **PUFA**=polyunsaturated fatty acids; **SD**=standard deviation; **UCI**= upper confidence interval; **VLDL**=very-low-density lipoprotein.*

Metabolic traits	temperature	N.obs	N.indiv	Beta	LCI	UCI	Pvalue	SD
**Lipoprotein subclasses**								
Extremely large VLDL								
Particle concentration (mol/l)	4°C	69	23	-4.8e-12	-1.3e-11	2.8e-12	2.2e-01	2.0e-10
Particle concentration (mol/l)	21°C	69	23	-2.8e-12	-1.5e-11	9.5e-12	6.5e-01	2.0e-10
Total lipids (mmol/l)	4°C	69	23	-1.0e-03	-2.7e-03	6.0e-04	2.1e-01	4.2e-02
Total lipids (mmol/l)	21°C	69	23	-6.0e-04	-3.2e-03	2.0e-03	6.5e-01	4.2e-02
Phospholipids (mmol/l)	4°C	69	23	-1.5e-04	-3.4e-04	3.9e-05	1.2e-01	5.3e-03
Phospholipids (mmol/l)	21°C	69	23	-1.4e-04	-4.5e-04	1.7e-04	3.7e-01	5.3e-03
Total cholesterol (mmol/l)	4°C	69	23	-1.3e-04	-4.1e-04	1.6e-04	3.9e-01	7.9e-03
Total cholesterol (mmol/l)	21°C	69	23	5.1e-05	-4.2e-04	5.3e-04	8.3e-01	7.9e-03
Cholesterol esters (mmol/l)	4°C	69	23	-2.4e-05	-2.0e-04	1.5e-04	7.9e-01	4.5e-03
Cholesterol esters (mmol/l)	21°C	69	23	1.7e-04	-1.2e-04	4.7e-04	2.6e-01	4.5e-03
Free cholesterol (mmol/l)	4°C	69	23	-1.0e-04	-2.1e-04	1.2e-05	8.1e-02	3.5e-03
Free cholesterol (mmol/l)	21°C	69	23	-1.2e-04	-3.1e-04	6.9e-05	2.1e-01	3.5e-03
Triglycerides (mmol/l)	4°C	69	23	-7.5e-04	-1.9e-03	4.1e-04	2.0e-01	2.9e-02
Triglycerides (mmol/l)	21°C	69	23	-5.1e-04	-2.4e-03	1.3e-03	5.9e-01	2.9e-02
Very large VLDL								
Particle concentration (mol/l)	4°C	69	23	-3.3e-11	-6.7e-11	1.6e-12	6.2e-02	1.2e-09
Particle concentration (mol/l)	21°C	69	23	-3.7e-11	-9.9e-11	2.4e-11	2.3e-01	1.2e-09
Total lipids (mmol/l)	4°C	69	23	-3.1e-03	-6.5e-03	2.4e-04	6.8e-02	1.1e-01

Metabolic traits	temperature	N.obs	N.indiv	Beta	LCI	UCI	Pvalue	SD
Total lipids (mmol/l)	21°C	69	23	-3.4e-03	-9.4e-03	2.6e-03	2.7e-01	1.1e-01
Phospholipids (mmol/l)	4°C	69	23	-5.5e-04	-1.1e-03	-9.3e-06	4.6e-02	1.9e-02
Phospholipids (mmol/l)	21°C	69	23	-4.8e-04	-1.5e-03	5.0e-04	3.4e-01	1.9e-02
Total cholesterol (mmol/l)	4°C	69	23	-4.2e-04	-1.2e-03	3.4e-04	2.8e-01	2.3e-02
Total cholesterol (mmol/l)	21°C	69	23	-8.9e-05	-1.4e-03	1.2e-03	8.9e-01	2.3e-02
Cholesterol esters (mmol/l)	4°C	69	23	-1.7e-04	-5.9e-04	2.6e-04	4.5e-01	1.3e-02
Cholesterol esters (mmol/l)	21°C	69	23	8.4e-05	-6.3e-04	8.0e-04	8.2e-01	1.3e-02
Free cholesterol (mmol/l)	4°C	69	23	-2.5e-04	-5.8e-04	7.8e-05	1.3e-01	1.1e-02
Free cholesterol (mmol/l)	21°C	69	23	-1.7e-04	-7.3e-04	3.9e-04	5.5e-01	1.1e-02
Triglycerides (mmol/l)	4°C	69	23	-2.2e-03	-4.3e-03	-1.5e-05	4.8e-02	7.2e-02
Triglycerides (mmol/l)	21°C	69	23	-2.8e-03	-6.6e-03	9.8e-04	1.5e-01	7.2e-02
Large VLDL								
Particle concentration (mol/l)	4°C	69	23	-2.8e-10	-4.6e-10	-9.8e-11	2.6e-03	6.7e-09
Particle concentration (mol/l)	21°C	69	23	-3.7e-10	-7.0e-10	-4.1e-11	2.8e-02	6.7e-09
Total lipids (mmol/l)	4°C	69	23	-1.6e-02	-2.6e-02	-5.2e-03	3.4e-03	3.9e-01
Total lipids (mmol/l)	21°C	69	23	-2.1e-02	-4.0e-02	-1.5e-03	3.5e-02	3.9e-01
Phospholipids (mmol/l)	4°C	69	23	-2.9e-03	-4.7e-03	-1.0e-03	2.2e-03	7.0e-02
Phospholipids (mmol/l)	21°C	69	23	-3.6e-03	-7.0e-03	-1.7e-04	4.0e-02	7.0e-02
Total cholesterol (mmol/l)	4°C	69	23	-2.1e-03	-4.5e-03	3.4e-04	9.2e-02	9.1e-02
Total cholesterol (mmol/l)	21°C	69	23	-2.3e-03	-6.8e-03	2.2e-03	3.2e-01	9.1e-02
Cholesterol esters (mmol/l)	4°C	69	23	-6.3e-04	-1.9e-03	6.9e-04	3.5e-01	4.6e-02
Cholesterol esters (mmol/l)	21°C	69	23	-1.5e-04	-2.5e-03	2.2e-03	9.0e-01	4.6e-02
Free cholesterol (mmol/l)	4°C	69	23	-1.5e-03	-2.6e-03	-3.0e-04	1.4e-02	4.5e-02
Free cholesterol (mmol/l)	21°C	69	23	-2.1e-03	-4.3e-03	4.4e-05	5.5e-02	4.5e-02
Triglycerides (mmol/l)	4°C	69	23	-1.1e-02	-1.7e-02	-4.4e-03	9.6e-04	2.3e-01
Triglycerides (mmol/l)	21°C	69	23	-1.5e-02	-2.6e-02	-3.4e-03	1.1e-02	2.3e-01
Medium VLDL								

Metabolic traits	temperature	N.obs	N.indiv	Beta	LCI	UCI	Pvalue	SD
Particle concentration (mol/l)	4°C	69	23	-4.9e-10	-9.2e-10	-6.7e-11	2.3e-02	1.7e-08
Particle concentration (mol/l)	21°C	69	23	-4.9e-10	-1.3e-09	3.2e-10	2.4e-01	1.7e-08
Total lipids (mmol/l)	4°C	69	23	-1.6e-02	-3.0e-02	-1.8e-03	2.7e-02	5.5e-01
Total lipids (mmol/l)	21°C	69	23	-1.5e-02	-4.2e-02	1.2e-02	2.8e-01	5.5e-01
Phospholipids (mmol/l)	4°C	69	23	-3.0e-03	-5.6e-03	-3.5e-04	2.6e-02	1.1e-01
Phospholipids (mmol/l)	21°C	69	23	-2.5e-03	-7.6e-03	2.6e-03	3.3e-01	1.1e-01
Total cholesterol (mmol/l)	4°C	69	23	-2.3e-03	-5.8e-03	1.2e-03	2.0e-01	1.4e-01
Total cholesterol (mmol/l)	21°C	69	23	1.9e-03	-5.1e-03	8.8e-03	6.0e-01	1.4e-01
Cholesterol esters (mmol/l)	4°C	69	23	-1.6e-04	-2.1e-03	1.8e-03	8.7e-01	7.3e-02
Cholesterol esters (mmol/l)	21°C	69	23	4.4e-03	5.1e-04	8.3e-03	2.7e-02	7.3e-02
Free cholesterol (mmol/l)	4°C	69	23	-2.1e-03	-3.8e-03	-4.7e-04	1.2e-02	6.9e-02
Free cholesterol (mmol/l)	21°C	69	23	-2.6e-03	-5.7e-03	6.0e-04	1.1e-01	6.9e-02
Triglycerides (mmol/l)	4°C	69	23	-1.1e-02	-1.9e-02	-2.5e-03	1.0e-02	3.1e-01
Triglycerides (mmol/l)	21°C	69	23	-1.4e-02	-2.9e-02	1.0e-03	6.8e-02	3.1e-01
Small VLDL								
Particle concentration (mol/l)	4°C	69	23	-2.6e-10	-5.9e-10	6.6e-11	1.2e-01	1.6e-08
Particle concentration (mol/l)	21°C	69	23	-1.4e-11	-6.6e-10	6.3e-10	9.7e-01	1.6e-08
Total lipids (mmol/l)	4°C	69	23	-4.3e-03	-1.0e-02	1.8e-03	1.6e-01	3.1e-01
Total lipids (mmol/l)	21°C	69	23	2.3e-03	-9.7e-03	1.4e-02	7.1e-01	3.1e-01
Phospholipids (mmol/l)	4°C	69	23	-6.5e-04	-1.9e-03	6.4e-04	3.2e-01	6.2e-02
Phospholipids (mmol/l)	21°C	69	23	-8.7e-04	-3.0e-03	1.3e-03	4.2e-01	6.2e-02
Total cholesterol (mmol/l)	4°C	69	23	6.5e-04	-1.5e-03	2.8e-03	5.6e-01	9.0e-02
Total cholesterol (mmol/l)	21°C	69	23	9.9e-03	6.1e-03	1.4e-02	2.6e-07	9.0e-02
Cholesterol esters (mmol/l)	4°C	69	23	1.2e-03	-4.6e-04	2.9e-03	1.5e-01	5.1e-02
Cholesterol esters (mmol/l)	21°C	69	23	9.9e-03	7.2e-03	1.3e-02	1.3e-12	5.1e-02
Free cholesterol (mmol/l)	4°C	69	23	-5.9e-04	-1.3e-03	1.1e-04	1.0e-01	4.0e-02
Free cholesterol (mmol/l)	21°C	69	23	-5.6e-05	-1.3e-03	1.2e-03	9.3e-01	4.0e-02

Metabolic traits	temperature	N.obs	N.indiv	Beta	LCI	UCI	Pvalue	SD
Triglycerides (mmol/l)	4°C	69	23	-4.3e-03	-8.0e-03	-6.1e-04	2.2e-02	1.6e-01
Triglycerides (mmol/l)	21°C	69	23	-6.7e-03	-1.4e-02	1.7e-04	5.6e-02	1.6e-01
Very Small VLDL								
Particle concentration (mol/l)	4°C	69	23	2.4e-10	-6.9e-11	5.5e-10	1.3e-01	9.9e-09
Particle concentration (mol/l)	21°C	69	23	2.1e-09	1.6e-09	2.7e-09	1.1e-15	9.9e-09
Total lipids (mmol/l)	4°C	69	23	3.5e-03	-7.0e-04	7.6e-03	1.0e-01	1.2e-01
Total lipids (mmol/l)	21°C	69	23	2.9e-02	2.2e-02	3.6e-02	< 0.1e-26	1.2e-01
Phospholipids (mmol/l)	4°C	69	23	6.2e-04	-7.4e-04	2.0e-03	3.7e-01	3.2e-02
Phospholipids (mmol/l)	21°C	69	23	9.6e-03	7.8e-03	1.2e-02	< 0.1e-26	3.2e-02
Total cholesterol (mmol/l)	4°C	69	23	3.0e-03	3.8e-06	6.0e-03	5.0e-02	4.9e-02
Total cholesterol (mmol/l)	21°C	69	23	1.9e-02	1.4e-02	2.3e-02	< 0.1e-26	4.9e-02
Cholesterol esters (mmol/l)	4°C	69	23	2.5e-03	2.1e-05	4.9e-03	4.8e-02	3.5e-02
Cholesterol esters (mmol/l)	21°C	69	23	1.4e-02	1.1e-02	1.8e-02	6.2e-15	3.5e-02
Free cholesterol (mmol/l)	4°C	69	23	5.4e-04	-1.4e-04	1.2e-03	1.2e-01	1.5e-02
Free cholesterol (mmol/l)	21°C	69	23	4.6e-03	3.8e-03	5.5e-03	< 0.1e-26	1.5e-02
Triglycerides (mmol/l)	4°C	69	23	-1.7e-04	-1.1e-03	7.4e-04	7.1e-01	4.8e-02
Triglycerides (mmol/l)	21°C	69	23	6.3e-04	-7.2e-04	2.0e-03	3.6e-01	4.8e-02
IDL								
Particle concentration (mol/l)	4°C	69	23	2.2e-11	-1.1e-09	1.1e-09	9.7e-01	2.0e-08
Particle concentration (mol/l)	21°C	69	23	6.3e-09	5.0e-09	7.5e-09	< 0.1e-26	2.0e-08
Total lipids (mmol/l)	4°C	69	23	1.9e-04	-1.2e-02	1.2e-02	9.7e-01	2.0e-01
Total lipids (mmol/l)	21°C	69	23	6.5e-02	5.3e-02	7.8e-02	< 0.1e-26	2.0e-01
Phospholipids (mmol/l)	4°C	69	23	2.7e-04	-2.3e-03	2.9e-03	8.4e-01	4.8e-02
Phospholipids (mmol/l)	21°C	69	23	1.6e-02	1.4e-02	1.9e-02	< 0.1e-26	4.8e-02
Total cholesterol (mmol/l)	4°C	69	23	-2.1e-04	-9.6e-03	9.2e-03	9.7e-01	1.3e-01
Total cholesterol (mmol/l)	21°C	69	23	4.6e-02	3.7e-02	5.6e-02	< 0.1e-26	1.3e-01
Cholesterol esters (mmol/l)	4°C	69	23	-3.9e-04	-7.6e-03	6.8e-03	9.2e-01	1.0e-01

Metabolic traits	temperature	N.obs	N.indiv	Beta	LCI	UCI	Pvalue	SD
Cholesterol esters (mmol/l)	21°C	69	23	3.4e-02	2.7e-02	4.1e-02	< 0.1e-26	1.0e-01
Free cholesterol (mmol/l)	4°C	69	23	1.7e-04	-2.1e-03	2.5e-03	8.8e-01	3.6e-02
Free cholesterol (mmol/l)	21°C	69	23	1.2e-02	1.0e-02	1.5e-02	< 0.1e-26	3.6e-02
Triglycerides (mmol/l)	4°C	69	23	1.3e-04	-1.0e-03	1.3e-03	8.2e-01	3.5e-02
Triglycerides (mmol/l)	21°C	69	23	2.5e-03	9.8e-04	4.1e-03	1.4e-03	3.5e-02
Large LDL								
Particle concentration (mol/l)	4°C	69	23	-5.4e-10	-2.3e-09	1.2e-09	5.5e-01	3.4e-08
Particle concentration (mol/l)	21°C	69	23	9.3e-09	7.7e-09	1.1e-08	< 0.1e-26	3.4e-08
Total lipids (mmol/l)	4°C	69	23	-3.6e-03	-1.7e-02	9.4e-03	5.9e-01	2.4e-01
Total lipids (mmol/l)	21°C	69	23	6.8e-02	5.7e-02	8.0e-02	< 0.1e-26	2.4e-01
Phospholipids (mmol/l)	4°C	69	23	-1.0e-03	-3.9e-03	1.8e-03	4.9e-01	5.0e-02
Phospholipids (mmol/l)	21°C	69	23	1.4e-02	1.2e-02	1.6e-02	< 0.1e-26	5.0e-02
Total cholesterol (mmol/l)	4°C	69	23	-2.6e-03	-1.3e-02	7.4e-03	6.1e-01	1.7e-01
Total cholesterol (mmol/l)	21°C	69	23	5.2e-02	4.3e-02	6.1e-02	< 0.1e-26	1.7e-01
Cholesterol esters (mmol/l)	4°C	69	23	-2.4e-03	-9.9e-03	5.1e-03	5.3e-01	1.3e-01
Cholesterol esters (mmol/l)	21°C	69	23	3.9e-02	3.2e-02	4.5e-02	< 0.1e-26	1.3e-01
Free cholesterol (mmol/l)	4°C	69	23	-1.3e-04	-2.6e-03	2.4e-03	9.2e-01	4.1e-02
Free cholesterol (mmol/l)	21°C	69	23	1.3e-02	1.1e-02	1.6e-02	< 0.1e-26	4.1e-02
Triglycerides (mmol/l)	4°C	69	23	-5.7e-05	-1.3e-03	1.2e-03	9.3e-01	2.8e-02
Triglycerides (mmol/l)	21°C	69	23	2.0e-03	4.6e-04	3.5e-03	1.1e-02	2.8e-02
Medium LDL								
Particle concentration (mol/l)	4°C	69	23	-4.5e-10	-2.0e-09	1.1e-09	5.7e-01	2.9e-08
Particle concentration (mol/l)	21°C	69	23	7.8e-09	6.4e-09	9.2e-09	< 0.1e-26	2.9e-08
Total lipids (mmol/l)	4°C	69	23	-2.1e-03	-9.9e-03	5.8e-03	6.1e-01	1.5e-01
Total lipids (mmol/l)	21°C	69	23	4.1e-02	3.3e-02	4.8e-02	< 0.1e-26	1.5e-01
Phospholipids (mmol/l)	4°C	69	23	-6.1e-04	-2.3e-03	1.1e-03	4.8e-01	3.6e-02
Phospholipids (mmol/l)	21°C	69	23	7.4e-03	6.1e-03	8.8e-03	< 0.1e-26	3.6e-02

Metabolic traits	temperature	N.obs	N.indiv	Beta	LCI	UCI	Pvalue	SD
Total cholesterol (mmol/l)	4°C	69	23	-1.2e-03	-7.2e-03	4.8e-03	6.9e-01	1.1e-01
Total cholesterol (mmol/l)	21°C	69	23	3.3e-02	2.7e-02	3.8e-02	< 0.1e-26	1.1e-01
Cholesterol esters (mmol/l)	4°C	69	23	-1.1e-03	-5.8e-03	3.6e-03	6.4e-01	8.6e-02
Cholesterol esters (mmol/l)	21°C	69	23	2.6e-02	2.2e-02	3.0e-02	< 0.1e-26	8.6e-02
Free cholesterol (mmol/l)	4°C	69	23	-9.2e-05	-1.4e-03	1.2e-03	8.9e-01	2.1e-02
Free cholesterol (mmol/l)	21°C	69	23	6.5e-03	5.1e-03	7.9e-03	< 0.1e-26	2.1e-02
Triglycerides (mmol/l)	4°C	69	23	-2.6e-04	-1.2e-03	6.5e-04	5.8e-01	1.4e-02
Triglycerides (mmol/l)	21°C	69	23	4.3e-04	-5.4e-04	1.4e-03	3.9e-01	1.4e-02
Small LDL								
Particle concentration (mol/l)	4°C	69	23	-6.0e-10	-2.5e-09	1.3e-09	5.4e-01	3.4e-08
Particle concentration (mol/l)	21°C	69	23	8.5e-09	6.7e-09	1.0e-08	< 0.1e-26	3.4e-08
Total lipids (mmol/l)	4°C	69	23	-1.5e-03	-6.8e-03	3.9e-03	6.0e-01	9.5e-02
Total lipids (mmol/l)	21°C	69	23	2.5e-02	2.0e-02	3.0e-02	< 0.1e-26	9.5e-02
Phospholipids (mmol/l)	4°C	69	23	-5.2e-04	-2.0e-03	9.2e-04	4.8e-01	2.5e-02
Phospholipids (mmol/l)	21°C	69	23	4.7e-03	3.3e-03	6.1e-03	6.2e-11	2.5e-02
Total cholesterol (mmol/l)	4°C	69	23	-5.6e-04	-4.4e-03	3.3e-03	7.8e-01	6.6e-02
Total cholesterol (mmol/l)	21°C	69	23	2.0e-02	1.7e-02	2.4e-02	< 0.1e-26	6.6e-02
Cholesterol esters (mmol/l)	4°C	69	23	-5.5e-04	-3.5e-03	2.4e-03	7.2e-01	5.2e-02
Cholesterol esters (mmol/l)	21°C	69	23	1.6e-02	1.3e-02	1.9e-02	< 0.1e-26	5.2e-02
Free cholesterol (mmol/l)	4°C	69	23	-7.4e-06	-9.0e-04	8.8e-04	9.9e-01	1.4e-02
Free cholesterol (mmol/l)	21°C	69	23	4.2e-03	3.1e-03	5.3e-03	7.1e-14	1.4e-02
Triglycerides (mmol/l)	4°C	69	23	-3.8e-04	-8.0e-04	3.8e-05	7.5e-02	1.3e-02
Triglycerides (mmol/l)	21°C	69	23	-1.6e-04	-5.3e-04	2.2e-04	4.2e-01	1.3e-02
Very large HDL								
Particle concentration (mol/l)	4°C	69	23	-9.8e-09	-1.6e-08	-3.9e-09	1.2e-03	2.2e-07
Particle concentration (mol/l)	21°C	69	23	5.2e-09	-4.5e-09	1.5e-08	2.9e-01	2.2e-07
Total lipids (mmol/l)	4°C	69	23	-9.8e-03	-1.6e-02	-3.7e-03	1.7e-03	2.2e-01

Metabolic traits	temperature	N.obs	N.indiv	Beta	LCI	UCI	Pvalue	SD
Total lipids (mmol/l)	21°C	69	23	5.6e-03	-4.3e-03	1.6e-02	2.7e-01	2.2e-01
Phospholipids (mmol/l)	4°C	69	23	-6.2e-03	-9.3e-03	-3.1e-03	1.1e-04	1.3e-01
Phospholipids (mmol/l)	21°C	69	23	-1.6e-04	-5.2e-03	4.9e-03	9.5e-01	1.3e-01
Total cholesterol (mmol/l)	4°C	69	23	-3.2e-03	-7.0e-03	5.9e-04	9.8e-02	9.3e-02
Total cholesterol (mmol/l)	21°C	69	23	5.7e-03	3.8e-04	1.1e-02	3.5e-02	9.3e-02
Cholesterol esters (mmol/l)	4°C	69	23	-2.0e-03	-4.9e-03	8.5e-04	1.7e-01	6.5e-02
Cholesterol esters (mmol/l)	21°C	69	23	5.1e-03	1.2e-03	9.0e-03	1.1e-02	6.5e-02
Free cholesterol (mmol/l)	4°C	69	23	-1.2e-03	-2.1e-03	-2.0e-04	1.8e-02	2.8e-02
Free cholesterol (mmol/l)	21°C	69	23	5.9e-04	-8.2e-04	2.0e-03	4.1e-01	2.8e-02
Triglycerides (mmol/l)	4°C	69	23	-3.7e-04	-1.0e-03	3.0e-04	2.8e-01	1.0e-02
Triglycerides (mmol/l)	21°C	69	23	1.1e-04	-7.1e-04	9.3e-04	7.9e-01	1.0e-02
Large HDL								
Particle concentration (mol/l)	4°C	69	23	-3.6e-08	-6.5e-08	-6.0e-09	1.8e-02	6.3e-07
Particle concentration (mol/l)	21°C	69	23	-6.4e-08	-9.7e-08	-3.1e-08	1.7e-04	6.3e-07
Total lipids (mmol/l)	4°C	69	23	-2.3e-02	-4.1e-02	-4.6e-03	1.4e-02	4.0e-01
Total lipids (mmol/l)	21°C	69	23	-4.0e-02	-6.1e-02	-1.9e-02	1.9e-04	4.0e-01
Phospholipids (mmol/l)	4°C	69	23	-1.1e-02	-2.1e-02	-7.5e-04	3.6e-02	1.8e-01
Phospholipids (mmol/l)	21°C	69	23	-2.1e-02	-3.3e-02	-9.8e-03	2.7e-04	1.8e-01
Total cholesterol (mmol/l)	4°C	69	23	-1.2e-02	-2.0e-02	-4.0e-03	3.1e-03	2.1e-01
Total cholesterol (mmol/l)	21°C	69	23	-1.8e-02	-2.7e-02	-7.9e-03	3.6e-04	2.1e-01
Cholesterol esters (mmol/l)	4°C	69	23	-8.8e-03	-1.5e-02	-2.7e-03	4.4e-03	1.6e-01
Cholesterol esters (mmol/l)	21°C	69	23	-1.3e-02	-2.1e-02	-6.0e-03	3.5e-04	1.6e-01
Free cholesterol (mmol/l)	4°C	69	23	-3.0e-03	-4.9e-03	-1.1e-03	1.5e-03	5.2e-02
Free cholesterol (mmol/l)	21°C	69	23	-4.4e-03	-6.9e-03	-1.9e-03	6.5e-04	5.2e-02
Triglycerides (mmol/l)	4°C	69	23	-8.7e-05	-1.6e-03	1.5e-03	9.1e-01	1.4e-02
Triglycerides (mmol/l)	21°C	69	23	-1.4e-03	-3.0e-03	2.9e-04	1.1e-01	1.4e-02
Medium HDL								

Metabolic traits	temperature	N.obs	N.indiv	Beta	LCI	UCI	Pvalue	SD
Particle concentration (mol/l)	4°C	69	23	-4.4e-08	-9.7e-08	9.1e-09	1.0e-01	4.4e-07
Particle concentration (mol/l)	21°C	69	23	-1.3e-07	-1.8e-07	-6.7e-08	3.0e-05	4.4e-07
Total lipids (mmol/l)	4°C	69	23	-1.9e-02	-4.2e-02	4.0e-03	1.0e-01	1.9e-01
Total lipids (mmol/l)	21°C	69	23	-5.3e-02	-7.9e-02	-2.8e-02	5.0e-05	1.9e-01
Phospholipids (mmol/l)	4°C	69	23	-8.2e-03	-1.8e-02	1.9e-03	1.1e-01	8.8e-02
Phospholipids (mmol/l)	21°C	69	23	-2.7e-02	-3.8e-02	-1.5e-02	5.0e-06	8.8e-02
Total cholesterol (mmol/l)	4°C	69	23	-1.1e-02	-2.3e-02	2.0e-03	9.9e-02	1.1e-01
Total cholesterol (mmol/l)	21°C	69	23	-2.4e-02	-3.8e-02	-9.9e-03	9.3e-04	1.1e-01
Cholesterol esters (mmol/l)	4°C	69	23	-8.6e-03	-1.9e-02	1.4e-03	9.4e-02	8.2e-02
Cholesterol esters (mmol/l)	21°C	69	23	-1.8e-02	-3.0e-02	-7.2e-03	1.3e-03	8.2e-02
Free cholesterol (mmol/l)	4°C	69	23	-2.2e-03	-4.9e-03	5.8e-04	1.2e-01	2.3e-02
Free cholesterol (mmol/l)	21°C	69	23	-5.8e-03	-8.9e-03	-2.7e-03	2.6e-04	2.3e-02
Triglycerides (mmol/l)	4°C	69	23	-1.8e-04	-6.1e-04	2.5e-04	4.2e-01	1.7e-02
Triglycerides (mmol/l)	21°C	69	23	-2.5e-03	-3.4e-03	-1.7e-03	1.9e-09	1.7e-02
Small HDL								
Particle concentration (mol/l)	4°C	69	23	-3.0e-08	-1.1e-07	5.3e-08	4.8e-01	6.2e-07
Particle concentration (mol/l)	21°C	69	23	-2.0e-07	-3.0e-07	-9.5e-08	1.5e-04	6.2e-07
Total lipids (mmol/l)	4°C	69	23	-6.8e-03	-2.6e-02	1.2e-02	4.8e-01	1.4e-01
Total lipids (mmol/l)	21°C	69	23	-4.3e-02	-6.6e-02	-2.0e-02	2.9e-04	1.4e-01
Phospholipids (mmol/l)	4°C	69	23	-3.8e-03	-1.2e-02	4.0e-03	3.4e-01	8.1e-02
Phospholipids (mmol/l)	21°C	69	23	-3.5e-02	-4.6e-02	-2.4e-02	6.5e-10	8.1e-02
Total cholesterol (mmol/l)	4°C	69	23	-3.3e-03	-1.5e-02	8.6e-03	5.9e-01	7.6e-02
Total cholesterol (mmol/l)	21°C	69	23	-6.8e-03	-2.0e-02	6.7e-03	3.2e-01	7.6e-02
Cholesterol esters (mmol/l)	4°C	69	23	-2.5e-03	-1.3e-02	8.0e-03	6.4e-01	7.0e-02
Cholesterol esters (mmol/l)	21°C	69	23	-9.3e-04	-1.2e-02	1.1e-02	8.7e-01	7.0e-02
Free cholesterol (mmol/l)	4°C	69	23	-7.4e-04	-2.3e-03	8.4e-04	3.6e-01	1.5e-02
Free cholesterol (mmol/l)	21°C	69	23	-5.9e-03	-8.2e-03	-3.6e-03	6.2e-07	1.5e-02

Metabolic traits	temperature	N.obs	N.indiv	Beta	LCI	UCI	Pvalue	SD
Triglycerides (mmol/l)	4°C	69	23	2.0e-04	-2.8e-04	6.7e-04	4.2e-01	2.1e-02
Triglycerides (mmol/l)	21°C	69	23	-1.7e-03	-2.2e-03	-1.2e-03	3.7e-11	2.1e-02
**Lipoprotein particle size**								
VLDL particle size (nm)	4°C	69	23	-5.5e-02	-1.1e-01	1.5e-03	5.7e-02	1.5e+00
VLDL particle size (nm)	21°C	69	23	-2.0e-01	-3.0e-01	-1.0e-01	5.6e-05	1.5e+00
LDL particle size (nm)	4°C	69	23	5.7e-03	-9.1e-03	2.0e-02	4.5e-01	9.8e-02
LDL particle size (nm)	21°C	69	23	9.8e-03	-8.5e-03	2.8e-02	3.0e-01	9.8e-02
HDL particle size (nm)	4°C	69	23	-1.0e-02	-1.8e-02	-2.5e-03	9.8e-03	2.7e-01
HDL particle size (nm)	21°C	69	23	4.3e-03	-5.4e-03	1.4e-02	3.9e-01	2.7e-01
**Cholesterol**								
Total cholesterol (mmol/l)	4°C	69	23	-3.5e-02	-8.0e-02	9.2e-03	1.2e-01	7.3e-01
Total cholesterol (mmol/l)	21°C	69	23	1.4e-01	1.0e-01	1.7e-01	8.9e-16	7.3e-01
VLDL cholesterol (mmol/l)	4°C	69	23	-1.0e-03	-1.0e-02	8.2e-03	8.3e-01	3.8e-01
VLDL cholesterol (mmol/l)	21°C	69	23	2.8e-02	1.0e-02	4.7e-02	2.5e-03	3.8e-01
Remnant cholesterol (mmol/l)	4°C	69	23	-1.2e-03	-1.7e-02	1.5e-02	8.8e-01	4.7e-01
Remnant cholesterol (mmol/l)	21°C	69	23	7.5e-02	5.0e-02	1.0e-01	3.6e-09	4.7e-01
LDL cholesterol (mmol/l)	4°C	69	23	-4.2e-03	-2.4e-02	1.5e-02	6.7e-01	3.4e-01
LDL cholesterol (mmol/l)	21°C	69	23	1.1e-01	8.8e-02	1.2e-01	< 0.1e-26	3.4e-01
HDL cholesterol (mmol/l)	4°C	69	23	-3.0e-02	-5.8e-02	-2.3e-03	3.4e-02	3.9e-01
HDL cholesterol (mmol/l)	21°C	69	23	-4.3e-02	-7.6e-02	-8.8e-03	1.4e-02	3.9e-01
HDL2 cholesterol (mmol/l)	4°C	69	23	-2.7e-02	-5.2e-02	-2.8e-03	2.9e-02	3.6e-01
HDL2 cholesterol (mmol/l)	21°C	69	23	-4.0e-02	-6.9e-02	-1.1e-02	7.1e-03	3.6e-01
HDL3 cholesterol (mmol/l)	4°C	69	23	-2.6e-03	-5.9e-03	7.2e-04	1.2e-01	3.4e-02
HDL3 cholesterol (mmol/l)	21°C	69	23	-2.5e-03	-7.5e-03	2.6e-03	3.4e-01	3.4e-02
Esterified cholesterol (mmol/l)	4°C	60	20	-2.0e-02	-5.5e-02	1.4e-02	2.4e-01	5.3e-01
Esterified cholesterol (mmol/l)	21°C	60	20	1.5e-01	1.2e-01	1.9e-01	< 0.1e-26	5.3e-01
Free cholesterol (mmol/l)	4°C	60	20	-2.0e-02	-4.0e-02	-5.4e-04	4.4e-02	2.0e-01

Metabolic traits	temperature	N.obs	N.indiv	Beta	LCI	UCI	Pvalue	SD
Free cholesterol (mmol/l)	21°C	60	20	-1.9e-02	-3.6e-02	-1.6e-03	3.2e-02	2.0e-01
**Glycerides and phospholipids**								
Triglycerides (mmol/l)	4°C	69	23	-2.9e-02	-4.8e-02	-9.4e-03	3.6e-03	9.5e-01
Triglycerides (mmol/l)	21°C	69	23	-3.8e-02	-7.7e-02	3.9e-04	5.2e-02	9.5e-01
VLDL triglycerides (mmol/l)	4°C	69	23	-2.7e-02	-4.7e-02	-6.4e-03	1.0e-02	8.4e-01
VLDL triglycerides (mmol/l)	21°C	69	23	-3.8e-02	-7.7e-02	1.4e-03	5.8e-02	8.4e-01
LDL triglycerides (mmol/l)	4°C	69	23	-6.9e-04	-3.2e-03	1.8e-03	5.9e-01	5.5e-02
LDL triglycerides (mmol/l)	21°C	69	23	2.3e-03	-4.9e-04	5.0e-03	1.1e-01	5.5e-02
HDL triglycerides (mmol/l)	4°C	69	23	-1.3e-03	-2.3e-03	-3.2e-04	9.0e-03	4.9e-02
HDL triglycerides (mmol/l)	21°C	69	23	-5.4e-03	-7.1e-03	-3.7e-03	6.3e-10	4.9e-02
Diacylglycerol (mmol/l)	4°C	57	19	-1.4e-03	-3.9e-03	1.2e-03	3.0e-01	2.5e-02
Diacylglycerol (mmol/l)	21°C	57	19	-5.0e-04	-3.4e-03	2.4e-03	7.4e-01	2.5e-02
Phosphoglycerides (mmol/l)	4°C	60	20	-2.2e-02	-5.2e-02	7.3e-03	1.4e-01	3.8e-01
Phosphoglycerides (mmol/l)	21°C	60	20	-1.1e-02	-3.4e-02	1.2e-02	3.6e-01	3.8e-01
Phosphatidylcholine + other cholines (mmol/l)	4°C	60	20	-3.1e-02	-6.0e-02	-2.3e-03	3.4e-02	3.5e-01
Phosphatidylcholine + other cholines (mmol/l)	21°C	60	20	-5.2e-02	-7.4e-02	-3.0e-02	2.7e-06	3.5e-01
Sphingomyelins (mmol/l)	4°C	60	20	-1.1e-03	-1.3e-02	1.1e-02	8.5e-01	7.1e-02
Sphingomyelins (mmol/l)	21°C	60	20	4.3e-03	-7.2e-03	1.6e-02	4.6e-01	7.1e-02
Cholines (mmol/l)	4°C	60	20	-2.5e-02	-5.7e-02	6.6e-03	1.2e-01	3.7e-01
Cholines (mmol/l)	21°C	60	20	-4.0e-03	-3.0e-02	2.2e-02	7.7e-01	3.7e-01
**Apolipoproteins**								
Apolipoprotein A-I (g/l)	4°C	69	23	-2.1e-02	-3.8e-02	-4.8e-03	1.1e-02	2.0e-01
Apolipoprotein A-I (g/l)	21°C	69	23	-2.0e-02	-3.7e-02	-1.9e-03	3.0e-02	2.0e-01
Apolipoprotein B (g/l)	4°C	69	23	-4.1e-03	-1.3e-02	4.5e-03	3.5e-01	2.4e-01
Apolipoprotein B (g/l)	21°C	69	23	3.3e-02	2.1e-02	4.5e-02	1.2e-07	2.4e-01
**Fatty acids**								

Metabolic traits	temperature	N.obs	N.indiv	Beta	LCI	UCI	Pvalue	SD
Total fatty acids (mmol/l)	4°C	60	20	-2.1e-01	-3.7e-01	-3.8e-02	1.6e-02	3.4e+00
Total fatty acids (mmol/l)	21°C	60	20	-2.9e-02	-1.7e-01	1.1e-01	6.8e-01	3.4e+00
Fatty acid chain length	4°C	60	20	-9.0e-03	-5.1e-02	3.3e-02	6.7e-01	2.6e-01
Fatty acid chain length	21°C	60	20	-2.2e-02	-6.8e-02	2.3e-02	3.4e-01	2.6e-01
Degree of unsaturation	4°C	60	20	2.1e-03	-3.6e-03	7.7e-03	4.7e-01	7.4e-02
Degree of unsaturation	21°C	60	20	7.7e-03	9.9e-04	1.4e-02	2.5e-02	7.4e-02
Docosahexaenoic acid (mmol/l)	4°C	60	20	-5.2e-04	-4.3e-03	3.2e-03	7.8e-01	4.8e-02
Docosahexaenoic acid (mmol/l)	21°C	60	20	1.8e-03	-5.1e-04	4.0e-03	1.3e-01	4.8e-02
Linoleic acid (mmol/l)	4°C	60	20	-3.9e-02	-7.7e-02	-1.4e-03	4.2e-02	6.3e-01
Linoleic acid (mmol/l)	21°C	60	20	2.7e-02	-2.2e-03	5.5e-02	7.1e-02	6.3e-01
Conjugated linoleic acid (mmol/l)	4°C	60	20	-2.3e-03	-4.7e-03	-1.4e-05	4.9e-02	2.2e-02
Conjugated linoleic acid (mmol/l)	21°C	60	20	-2.1e-03	-4.7e-03	6.0e-04	1.3e-01	2.2e-02
n-3 fatty acids (mmol/l)	4°C	60	20	-2.0e-03	-1.1e-02	6.7e-03	6.5e-01	1.6e-01
n-3 fatty acids (mmol/l)	21°C	60	20	6.7e-03	1.7e-03	1.2e-02	8.9e-03	1.6e-01
n-6 fatty acids (mmol/l)	4°C	60	20	-5.6e-02	-1.0e-01	-9.5e-03	1.8e-02	7.1e-01
n-6 fatty acids (mmol/l)	21°C	60	20	3.1e-02	-4.2e-03	6.6e-02	8.5e-02	7.1e-01
PUFA (mmol/l)	4°C	60	20	-5.8e-02	-1.1e-01	-5.7e-03	3.0e-02	8.5e-01
PUFA (mmol/l)	21°C	60	20	3.7e-02	-1.6e-03	7.6e-02	6.0e-02	8.5e-01
MUFA (mmol/l)	4°C	60	20	-6.0e-02	-1.0e-01	-1.5e-02	8.4e-03	1.3e+00
MUFA (mmol/l)	21°C	60	20	-3.2e-02	-8.1e-02	1.6e-02	1.9e-01	1.3e+00
Saturated fatty acids (mmol/l)	4°C	60	20	-8.8e-02	-1.7e-01	-4.9e-03	3.8e-02	1.3e+00
Saturated fatty acids (mmol/l)	21°C	60	20	-3.4e-02	-9.9e-02	3.0e-02	3.0e-01	1.3e+00
**Glycolysis related metabolites**								
Glucose (mmol/l)	4°C	69	23	-1.2e+00	-1.3e+00	-1.2e+00	< 0.1e-26	1.5e+00
Glucose (mmol/l)	21°C	69	23	-1.9e+00	-2.2e+00	-1.7e+00	< 0.1e-26	1.5e+00
Lactate (mmol/l)	4°C	69	23	2.1e+00	2.0e+00	2.2e+00	< 0.1e-26	2.5e+00

Metabolic traits	temperature	N.obs	N.indiv	Beta	LCI	UCI	Pvalue	SD
Lactate (mmol/l)	21°C	69	23	3.3e+00	2.9e+00	3.7e+00	< 0.1e-26	2.5e+00
Pyruvate (mmol/l)	4°C			NA	NA	NA	NA	NA
Pyruvate (mmol/l)	21°C			NA	NA	NA	NA	NA
Citrate (mmol/l)	4°C	69	23	-2.0e-03	-5.5e-03	1.4e-03	2.4e-01	3.4e-02
Citrate (mmol/l)	21°C	69	23	-7.5e-03	-1.1e-02	-4.0e-03	2.1e-05	3.4e-02
Glycerol (mmol/l)	4°C			NA	NA	NA	NA	NA
Glycerol (mmol/l)	21°C			NA	NA	NA	NA	NA
**Amino acids**								
Alanine (mmol/l)	4°C	69	23	6.4e-03	3.3e-03	9.4e-03	3.6e-05	1.2e-01
Alanine (mmol/l)	21°C	69	23	1.3e-01	1.2e-01	1.4e-01	< 0.1e-26	1.2e-01
Glutamine (mmol/l)	4°C	69	23	-1.4e-02	-1.9e-02	-9.5e-03	2.5e-09	6.7e-02
Glutamine (mmol/l)	21°C	69	23	-5.8e-02	-6.4e-02	-5.3e-02	< 0.1e-26	6.7e-02
Histidine (mmol/l)	4°C	69	23	-3.5e-03	-4.9e-03	-2.2e-03	3.0e-07	7.9e-03
Histidine (mmol/l)	21°C	69	23	-5.6e-03	-7.3e-03	-3.9e-03	1.2e-10	7.9e-03
Glycine (mmol/l)	4°C			NA	NA	NA	NA	NA
Glycine (mmol/l)	21°C			NA	NA	NA	NA	NA
Branched-chain amino acids								
Isoleucine (mmol/l)	4°C	69	23	8.2e-04	-1.9e-04	1.8e-03	1.1e-01	2.4e-02
Isoleucine (mmol/l)	21°C	69	23	-1.4e-04	-1.3e-03	1.0e-03	8.1e-01	2.4e-02
Leucine (mmol/l)	4°C	69	23	3.8e-03	3.3e-03	4.3e-03	< 0.1e-26	2.2e-02
Leucine (mmol/l)	21°C	69	23	6.9e-03	5.9e-03	7.9e-03	< 0.1e-26	2.2e-02
Valine (mmol/l)	4°C	69	23	4.0e-03	2.9e-03	5.1e-03	5.7e-13	3.7e-02
Valine (mmol/l)	21°C	69	23	9.8e-03	8.4e-03	1.1e-02	< 0.1e-26	3.7e-02
Aromatic amino acids								
Phenylalanine (mmol/l)	4°C	69	23	8.3e-04	-1.4e-05	1.7e-03	5.4e-02	6.2e-03
Phenylalanine (mmol/l)	21°C	69	23	4.2e-03	3.2e-03	5.2e-03	4.4e-16	6.2e-03
Tyrosine (mmol/l)	4°C	69	23	4.4e-04	-2.8e-04	1.2e-03	2.3e-01	1.2e-02

Metabolic traits	temperature	N.obs	N.indiv	Beta	LCI	UCI	Pvalue	SD
Tyrosine (mmol/l)	21°C	69	23	2.3e-03	1.7e-03	3.0e-03	1.3e-11	1.2e-02
**Ketone bodies**								
Acetate (mmol/l)	4°C	69	23	-8.0e-03	-9.7e-03	-6.2e-03	< 0.1e-26	9.4e-03
Acetate (mmol/l)	21°C	69	23	-7.5e-03	-1.0e-02	-5.0e-03	6.5e-09	9.4e-03
Beta-hydroxybutyrate (mmol/l)	4°C	69	23	-5.4e-04	-1.5e-03	4.3e-04	2.7e-01	1.8e-02
Beta-hydroxybutyrate (mmol/l)	21°C	69	23	2.9e-03	4.2e-04	5.3e-03	2.2e-02	1.8e-02
**Fluid balance**								
Creatinine (mmol/l)	4°C	69	23	-5.6e-04	-1.2e-03	9.9e-05	9.6e-02	9.1e-03
Creatinine (mmol/l)	21°C	69	23	-4.1e-04	-1.1e-03	3.1e-04	2.6e-01	9.1e-03
Albumin (signal area)	4°C	69	23	-4.8e-04	-1.0e-03	7.2e-05	8.8e-02	4.3e-03
Albumin (signal area)	21°C	69	23	1.8e-03	1.3e-03	2.3e-03	2.3e-12	4.3e-03
**Inflammation**								
Glycoprotein acetyls (mmol/l)	4°C	69	23	-1.0e-02	-1.8e-02	-3.0e-03	5.8e-03	3.2e-01
Glycoprotein acetyls (mmol/l)	21°C	69	23	1.8e-02	7.5e-03	2.8e-02	7.4e-04	3.2e-01

metabolites

Table S8. Serum, post-storage handling effects (differences in mean levels): mean differences in metabolite concentrations (or trait value) comparing 24 h delays in buffer addition (i.e. buffer delay) or NMR profiling (i.e. NMR delay) to the reference (no delays), for serum samples.

# Associations in Figure S6 are presented in SD-units. These SD point estimate can be obtained by dividing the point estimate (beta) in absolute (clinically meaningful) concentration by the metabolic trait standard deviation (SD), both provided in the below table.

***Abbreviations**: **C**=cholesterol; **IDL**=intermediate-density lipoprotein; **LCI**=lower confidence interval; **LDL**=low-density lipoprotein; **HDL**=high-density lipoprotein; **MUFA**=monounsaturated fatty acids; **N.obs**= number of observations (samples); **N.indiv**=number of individuals; **PUFA**=polyunsaturated fatty acids; **SD**=standard deviation; **UCI**= upper confidence interval; **VLDL**=very-low-density lipoprotein.*

Metabolic traits	delay	N.obs	N.indiv	Beta	LCI	UCI	Pvalue	SD
**Lipoprotein subclasses**								
Extremely large VLDL								
Particle concentration (mol/l)	Buffer delay	50	25	-1.9e-11	-3.1e-11	-6.8e-12	2.2e-03	2.1e-10
Particle concentration (mol/l)	NMR delay	50	25	-6.8e-12	-1.3e-11	-1.0e-13	4.7e-02	2.1e-10
Total lipids (mmol/l)	Buffer delay	50	25	-4.0e-03	-6.6e-03	-1.4e-03	2.3e-03	4.6e-02
Total lipids (mmol/l)	NMR delay	50	25	-1.5e-03	-2.9e-03	-5.7e-05	4.1e-02	4.6e-02
Phospholipids (mmol/l)	Buffer delay	50	25	-5.3e-04	-8.3e-04	-2.3e-04	5.7e-04	5.7e-03
Phospholipids (mmol/l)	NMR delay	50	25	-2.1e-04	-3.8e-04	-4.0e-05	1.6e-02	5.7e-03
Total cholesterol (mmol/l)	Buffer delay	50	25	-5.2e-04	-9.8e-04	-6.4e-05	2.6e-02	8.5e-03
Total cholesterol (mmol/l)	NMR delay	50	25	-3.1e-04	-5.7e-04	-5.5e-05	1.7e-02	8.5e-03
Cholesterol esters (mmol/l)	Buffer delay	50	25	-1.9e-04	-4.9e-04	1.1e-04	2.1e-01	4.7e-03
Cholesterol esters (mmol/l)	NMR delay	50	25	-1.7e-04	-3.4e-04	-6.0e-06	4.2e-02	4.7e-03
Free cholesterol (mmol/l)	Buffer delay	50	25	-3.3e-04	-5.1e-04	-1.6e-04	1.6e-04	3.8e-03
Free cholesterol (mmol/l)	NMR delay	50	25	-1.4e-04	-2.4e-04	-4.1e-05	5.7e-03	3.8e-03
Triglycerides (mmol/l)	Buffer delay	50	25	-3.0e-03	-4.8e-03	-1.1e-03	1.6e-03	3.1e-02
Triglycerides (mmol/l)	NMR delay	50	25	-9.6e-04	-2.0e-03	5.0e-05	6.3e-02	3.1e-02
Very large VLDL								
Particle concentration (mol/l)	Buffer delay	50	25	-9.1e-11	-1.4e-10	-4.2e-11	2.8e-04	1.3e-09
Particle concentration (mol/l)	NMR delay	50	25	-3.9e-11	-6.6e-11	-1.2e-11	4.9e-03	1.3e-09
Total lipids (mmol/l)	Buffer delay	50	25	-9.1e-03	-1.4e-02	-4.2e-03	2.5e-04	1.2e-01
Total lipids (mmol/l)	NMR delay	50	25	-3.9e-03	-6.5e-03	-1.2e-03	4.5e-03	1.2e-01

Metabolic traits	delay	N.obs	N.indiv	Beta	LCI	UCI	Pvalue	SD
Phospholipids (mmol/l)	Buffer delay	50	25	-1.7e-03	-2.5e-03	-8.5e-04	5.9e-05	2.1e-02
Phospholipids (mmol/l)	NMR delay	50	25	-7.1e-04	-1.2e-03	-2.4e-04	3.4e-03	2.1e-02
Total cholesterol (mmol/l)	Buffer delay	50	25	-1.9e-03	-3.0e-03	-7.4e-04	1.3e-03	2.5e-02
Total cholesterol (mmol/l)	NMR delay	50	25	-8.9e-04	-1.5e-03	-2.6e-04	5.3e-03	2.5e-02
Cholesterol esters (mmol/l)	Buffer delay	50	25	-8.3e-04	-1.5e-03	-2.0e-04	9.8e-03	1.4e-02
Cholesterol esters (mmol/l)	NMR delay	50	25	-4.6e-04	-8.0e-04	-1.1e-04	8.9e-03	1.4e-02
Free cholesterol (mmol/l)	Buffer delay	50	25	-1.1e-03	-1.6e-03	-5.2e-04	1.0e-04	1.1e-02
Free cholesterol (mmol/l)	NMR delay	50	25	-4.3e-04	-7.2e-04	-1.4e-04	3.4e-03	1.1e-02
Triglycerides (mmol/l)	Buffer delay	50	25	-5.5e-03	-8.5e-03	-2.5e-03	3.4e-04	7.8e-02
Triglycerides (mmol/l)	NMR delay	50	25	-2.3e-03	-3.9e-03	-6.5e-04	6.1e-03	7.8e-02
Large VLDL								
Particle concentration (mol/l)	Buffer delay	50	25	-2.3e-10	-4.5e-10	-1.7e-11	3.4e-02	7.2e-09
Particle concentration (mol/l)	NMR delay	50	25	-1.3e-10	-2.7e-10	2.8e-12	5.5e-02	7.2e-09
Total lipids (mmol/l)	Buffer delay	50	25	-1.4e-02	-2.6e-02	-1.8e-03	2.5e-02	4.2e-01
Total lipids (mmol/l)	NMR delay	50	25	-8.1e-03	-1.6e-02	-6.4e-05	4.8e-02	4.2e-01
Phospholipids (mmol/l)	Buffer delay	50	25	-2.5e-03	-4.7e-03	-2.1e-04	3.2e-02	7.6e-02
Phospholipids (mmol/l)	NMR delay	50	25	-1.5e-03	-2.9e-03	-5.5e-06	4.9e-02	7.6e-02
Total cholesterol (mmol/l)	Buffer delay	50	25	-4.7e-03	-7.8e-03	-1.5e-03	3.5e-03	9.7e-02
Total cholesterol (mmol/l)	NMR delay	50	25	-2.3e-03	-4.3e-03	-3.4e-04	2.2e-02	9.7e-02
Cholesterol esters (mmol/l)	Buffer delay	50	25	-2.4e-03	-4.2e-03	-5.6e-04	1.1e-02	4.8e-02
Cholesterol esters (mmol/l)	NMR delay	50	25	-1.1e-03	-2.1e-03	-7.1e-05	3.6e-02	4.8e-02
Free cholesterol (mmol/l)	Buffer delay	50	25	-2.3e-03	-3.6e-03	-8.9e-04	1.2e-03	4.9e-02
Free cholesterol (mmol/l)	NMR delay	50	25	-1.2e-03	-2.2e-03	-2.4e-04	1.5e-02	4.9e-02
Triglycerides (mmol/l)	Buffer delay	50	25	-7.0e-03	-1.4e-02	3.6e-04	6.2e-02	2.5e-01
Triglycerides (mmol/l)	NMR delay	50	25	-4.3e-03	-9.0e-03	3.6e-04	7.1e-02	2.5e-01
Medium VLDL								
Particle concentration (mol/l)	Buffer delay	50	25	-1.0e-10	-5.4e-10	3.3e-10	6.4e-01	1.8e-08

Metabolic traits	delay	N.obs	N.indiv	Beta	LCI	UCI	Pvalue	SD
Particle concentration (mol/l)	NMR delay	50	25	-1.7e-10	-5.2e-10	1.7e-10	3.3e-01	1.8e-08
Total lipids (mmol/l)	Buffer delay	50	25	-5.0e-03	-2.0e-02	9.7e-03	5.0e-01	5.9e-01
Total lipids (mmol/l)	NMR delay	50	25	-6.1e-03	-1.7e-02	5.4e-03	3.0e-01	5.9e-01
Phospholipids (mmol/l)	Buffer delay	50	25	-1.1e-03	-3.9e-03	1.7e-03	4.5e-01	1.1e-01
Phospholipids (mmol/l)	NMR delay	50	25	-1.3e-03	-3.5e-03	9.1e-04	2.5e-01	1.1e-01
Total cholesterol (mmol/l)	Buffer delay	50	25	-5.6e-03	-1.0e-02	-1.1e-03	1.4e-02	1.5e-01
Total cholesterol (mmol/l)	NMR delay	50	25	-2.5e-03	-5.5e-03	5.8e-04	1.1e-01	1.5e-01
Cholesterol esters (mmol/l)	Buffer delay	50	25	-4.7e-03	-7.5e-03	-1.9e-03	9.8e-04	7.4e-02
Cholesterol esters (mmol/l)	NMR delay	50	25	-1.7e-03	-3.5e-03	1.5e-04	7.3e-02	7.4e-02
Free cholesterol (mmol/l)	Buffer delay	50	25	-8.9e-04	-2.7e-03	9.1e-04	3.3e-01	7.3e-02
Free cholesterol (mmol/l)	NMR delay	50	25	-7.8e-04	-2.2e-03	6.4e-04	2.8e-01	7.3e-02
Triglycerides (mmol/l)	Buffer delay	50	25	1.7e-03	-6.1e-03	9.5e-03	6.7e-01	3.3e-01
Triglycerides (mmol/l)	NMR delay	50	25	-2.4e-03	-8.9e-03	4.2e-03	4.8e-01	3.3e-01
Small VLDL								
Particle concentration (mol/l)	Buffer delay	50	25	-7.3e-10	-1.1e-09	-3.7e-10	7.2e-05	1.7e-08
Particle concentration (mol/l)	NMR delay	50	25	-3.2e-11	-3.6e-10	3.0e-10	8.5e-01	1.7e-08
Total lipids (mmol/l)	Buffer delay	50	25	-1.7e-02	-2.4e-02	-1.0e-02	1.4e-06	3.2e-01
Total lipids (mmol/l)	NMR delay	50	25	-8.8e-04	-7.3e-03	5.5e-03	7.9e-01	3.2e-01
Phospholipids (mmol/l)	Buffer delay	50	25	-4.7e-03	-6.2e-03	-3.1e-03	4.5e-09	6.5e-02
Phospholipids (mmol/l)	NMR delay	50	25	-3.0e-04	-1.9e-03	1.3e-03	7.2e-01	6.5e-02
Total cholesterol (mmol/l)	Buffer delay	50	25	-1.4e-02	-1.7e-02	-1.0e-02	2.0e-15	8.7e-02
Total cholesterol (mmol/l)	NMR delay	50	25	-1.2e-03	-4.1e-03	1.7e-03	4.1e-01	8.7e-02
Cholesterol esters (mmol/l)	Buffer delay	50	25	-1.1e-02	-1.4e-02	-8.2e-03	4.4e-15	4.7e-02
Cholesterol esters (mmol/l)	NMR delay	50	25	-1.1e-03	-3.4e-03	1.3e-03	3.8e-01	4.7e-02
Free cholesterol (mmol/l)	Buffer delay	50	25	-2.8e-03	-3.6e-03	-1.9e-03	1.2e-09	4.1e-02
Free cholesterol (mmol/l)	NMR delay	50	25	-1.8e-04	-1.0e-03	6.6e-04	6.8e-01	4.1e-02
Triglycerides (mmol/l)	Buffer delay	50	25	1.2e-03	-2.2e-03	4.6e-03	4.9e-01	1.7e-01

Metabolic traits	delay	N.obs	N.indiv	Beta	LCI	UCI	Pvalue	SD
Triglycerides (mmol/l)	NMR delay	50	25	5.8e-04	-2.6e-03	3.8e-03	7.2e-01	1.7e-01
Very Small VLDL								
Particle concentration (mol/l)	Buffer delay	50	25	-1.4e-09	-2.1e-09	-7.4e-10	3.7e-05	9.1e-09
Particle concentration (mol/l)	NMR delay	50	25	1.4e-10	-2.3e-10	5.0e-10	4.6e-01	9.1e-09
Total lipids (mmol/l)	Buffer delay	50	25	-2.0e-02	-2.9e-02	-1.1e-02	2.2e-05	1.1e-01
Total lipids (mmol/l)	NMR delay	50	25	1.6e-03	-3.6e-03	6.7e-03	5.5e-01	1.1e-01
Phospholipids (mmol/l)	Buffer delay	50	25	4.2e-04	-2.5e-03	3.3e-03	7.8e-01	3.0e-02
Phospholipids (mmol/l)	NMR delay	50	25	-1.4e-03	-2.8e-03	1.3e-04	7.4e-02	3.0e-02
Total cholesterol (mmol/l)	Buffer delay	50	25	-2.1e-02	-2.8e-02	-1.5e-02	3.2e-10	4.6e-02
Total cholesterol (mmol/l)	NMR delay	50	25	2.3e-03	-2.2e-03	6.7e-03	3.2e-01	4.6e-02
Cholesterol esters (mmol/l)	Buffer delay	50	25	-1.9e-02	-2.4e-02	-1.4e-02	2.0e-13	3.2e-02
Cholesterol esters (mmol/l)	NMR delay	50	25	2.6e-03	-1.0e-03	6.2e-03	1.6e-01	3.2e-02
Free cholesterol (mmol/l)	Buffer delay	50	25	-2.1e-03	-3.7e-03	-4.1e-04	1.5e-02	1.4e-02
Free cholesterol (mmol/l)	NMR delay	50	25	-3.2e-04	-1.3e-03	6.3e-04	5.1e-01	1.4e-02
Triglycerides (mmol/l)	Buffer delay	50	25	7.0e-04	-2.1e-04	1.6e-03	1.3e-01	4.8e-02
Triglycerides (mmol/l)	NMR delay	50	25	6.8e-04	-2.1e-04	1.6e-03	1.3e-01	4.8e-02
IDL								
Particle concentration (mol/l)	Buffer delay	50	25	-8.4e-10	-3.1e-09	1.4e-09	4.6e-01	2.0e-08
Particle concentration (mol/l)	NMR delay	50	25	-4.8e-10	-1.8e-09	8.6e-10	4.9e-01	2.0e-08
Total lipids (mmol/l)	Buffer delay	50	25	-9.7e-03	-3.3e-02	1.4e-02	4.2e-01	2.0e-01
Total lipids (mmol/l)	NMR delay	50	25	-5.3e-03	-2.0e-02	9.1e-03	4.7e-01	2.0e-01
Phospholipids (mmol/l)	Buffer delay	50	25	-8.4e-04	-6.3e-03	4.6e-03	7.6e-01	5.1e-02
Phospholipids (mmol/l)	NMR delay	50	25	-2.3e-03	-5.3e-03	7.5e-04	1.4e-01	5.1e-02
Total cholesterol (mmol/l)	Buffer delay	50	25	-1.2e-02	-3.0e-02	6.0e-03	1.9e-01	1.3e-01
Total cholesterol (mmol/l)	NMR delay	50	25	-3.5e-03	-1.5e-02	8.1e-03	5.6e-01	1.3e-01
Cholesterol esters (mmol/l)	Buffer delay	50	25	-1.1e-02	-2.5e-02	2.1e-03	1.0e-01	9.8e-02
Cholesterol esters (mmol/l)	NMR delay	50	25	-2.0e-03	-1.1e-02	7.0e-03	6.6e-01	9.8e-02

Metabolic traits	delay	N.obs	N.indiv	Beta	LCI	UCI	Pvalue	SD
Free cholesterol (mmol/l)	Buffer delay	50	25	-5.4e-04	-5.1e-03	4.0e-03	8.1e-01	4.1e-02
Free cholesterol (mmol/l)	NMR delay	50	25	-1.4e-03	-4.1e-03	1.2e-03	3.0e-01	4.1e-02
Triglycerides (mmol/l)	Buffer delay	50	25	2.8e-03	1.7e-03	4.0e-03	1.6e-06	3.2e-02
Triglycerides (mmol/l)	NMR delay	50	25	4.8e-04	-3.7e-04	1.3e-03	2.7e-01	3.2e-02
Large LDL								
Particle concentration (mol/l)	Buffer delay	50	25	9.8e-10	-2.2e-09	4.2e-09	5.5e-01	3.4e-08
Particle concentration (mol/l)	NMR delay	50	25	-1.3e-09	-3.2e-09	6.1e-10	1.8e-01	3.4e-08
Total lipids (mmol/l)	Buffer delay	50	25	6.7e-03	-1.7e-02	3.0e-02	5.7e-01	2.5e-01
Total lipids (mmol/l)	NMR delay	50	25	-9.1e-03	-2.3e-02	5.0e-03	2.1e-01	2.5e-01
Phospholipids (mmol/l)	Buffer delay	50	25	1.6e-04	-4.6e-03	4.9e-03	9.5e-01	5.1e-02
Phospholipids (mmol/l)	NMR delay	50	25	-2.2e-03	-5.2e-03	8.5e-04	1.6e-01	5.1e-02
Total cholesterol (mmol/l)	Buffer delay	50	25	5.0e-03	-1.3e-02	2.3e-02	5.8e-01	1.8e-01
Total cholesterol (mmol/l)	NMR delay	50	25	-6.7e-03	-1.8e-02	4.4e-03	2.3e-01	1.8e-01
Cholesterol esters (mmol/l)	Buffer delay	50	25	4.8e-03	-8.7e-03	1.8e-02	4.9e-01	1.4e-01
Cholesterol esters (mmol/l)	NMR delay	50	25	-5.4e-03	-1.4e-02	3.0e-03	2.1e-01	1.4e-01
Free cholesterol (mmol/l)	Buffer delay	50	25	2.5e-04	-4.2e-03	4.7e-03	9.1e-01	4.7e-02
Free cholesterol (mmol/l)	NMR delay	50	25	-1.3e-03	-4.0e-03	1.4e-03	3.4e-01	4.7e-02
Triglycerides (mmol/l)	Buffer delay	50	25	1.3e-03	1.6e-04	2.5e-03	2.5e-02	2.6e-02
Triglycerides (mmol/l)	NMR delay	50	25	-2.7e-04	-1.3e-03	7.2e-04	6.0e-01	2.6e-02
Medium LDL								
Particle concentration (mol/l)	Buffer delay	50	25	4.2e-09	1.6e-09	6.7e-09	1.3e-03	3.0e-08
Particle concentration (mol/l)	NMR delay	50	25	-1.0e-09	-2.6e-09	5.4e-10	2.0e-01	3.0e-08
Total lipids (mmol/l)	Buffer delay	50	25	2.0e-02	7.1e-03	3.4e-02	2.6e-03	1.5e-01
Total lipids (mmol/l)	NMR delay	50	25	-5.0e-03	-1.3e-02	3.1e-03	2.3e-01	1.5e-01
Phospholipids (mmol/l)	Buffer delay	50	25	2.1e-03	-4.3e-04	4.7e-03	1.0e-01	3.4e-02
Phospholipids (mmol/l)	NMR delay	50	25	-7.0e-04	-2.4e-03	1.0e-03	4.3e-01	3.4e-02
Total cholesterol (mmol/l)	Buffer delay	50	25	1.7e-02	6.1e-03	2.7e-02	2.1e-03	1.1e-01

Metabolic traits	delay	N.obs	N.indiv	Beta	LCI	UCI	Pvalue	SD
Total cholesterol (mmol/l)	NMR delay	50	25	-3.8e-03	-1.0e-02	2.7e-03	2.5e-01	1.1e-01
Cholesterol esters (mmol/l)	Buffer delay	50	25	1.6e-02	7.2e-03	2.4e-02	3.1e-04	9.3e-02
Cholesterol esters (mmol/l)	NMR delay	50	25	-3.3e-03	-8.4e-03	1.9e-03	2.1e-01	9.3e-02
Free cholesterol (mmol/l)	Buffer delay	50	25	9.3e-04	-1.2e-03	3.0e-03	3.8e-01	2.2e-02
Free cholesterol (mmol/l)	NMR delay	50	25	-5.3e-04	-1.9e-03	8.7e-04	4.6e-01	2.2e-02
Triglycerides (mmol/l)	Buffer delay	50	25	1.6e-03	8.3e-04	2.3e-03	3.8e-05	1.3e-02
Triglycerides (mmol/l)	NMR delay	50	25	-5.7e-04	-1.4e-03	3.0e-04	2.0e-01	1.3e-02
Small LDL								
Particle concentration (mol/l)	Buffer delay	50	25	4.9e-09	2.0e-09	7.8e-09	9.8e-04	3.4e-08
Particle concentration (mol/l)	NMR delay	50	25	-1.4e-09	-3.3e-09	4.8e-10	1.4e-01	3.4e-08
Total lipids (mmol/l)	Buffer delay	50	25	1.3e-02	4.9e-03	2.2e-02	1.9e-03	9.6e-02
Total lipids (mmol/l)	NMR delay	50	25	-3.8e-03	-9.3e-03	1.6e-03	1.7e-01	9.6e-02
Phospholipids (mmol/l)	Buffer delay	50	25	1.9e-03	8.9e-05	3.7e-03	4.0e-02	2.4e-02
Phospholipids (mmol/l)	NMR delay	50	25	-9.0e-04	-2.2e-03	4.3e-04	1.8e-01	2.4e-02
Total cholesterol (mmol/l)	Buffer delay	50	25	1.1e-02	4.2e-03	1.7e-02	1.2e-03	7.1e-02
Total cholesterol (mmol/l)	NMR delay	50	25	-2.4e-03	-6.5e-03	1.7e-03	2.4e-01	7.1e-02
Cholesterol esters (mmol/l)	Buffer delay	50	25	1.0e-02	5.2e-03	1.5e-02	8.0e-05	5.8e-02
Cholesterol esters (mmol/l)	NMR delay	50	25	-2.0e-03	-5.1e-03	1.1e-03	2.1e-01	5.8e-02
Free cholesterol (mmol/l)	Buffer delay	50	25	4.0e-04	-9.8e-04	1.8e-03	5.7e-01	1.4e-02
Free cholesterol (mmol/l)	NMR delay	50	25	-4.5e-04	-1.5e-03	5.5e-04	3.8e-01	1.4e-02
Triglycerides (mmol/l)	Buffer delay	50	25	6.4e-04	2.3e-04	1.0e-03	2.2e-03	1.3e-02
Triglycerides (mmol/l)	NMR delay	50	25	-4.8e-04	-9.4e-04	-1.8e-05	4.2e-02	1.3e-02
Very large HDL								
Particle concentration (mol/l)	Buffer delay	50	25	-6.0e-08	-7.3e-08	-4.8e-08	< 0.1e-26	2.4e-07
Particle concentration (mol/l)	NMR delay	50	25	-2.3e-08	-3.2e-08	-1.4e-08	1.4e-06	2.4e-07
Total lipids (mmol/l)	Buffer delay	50	25	-6.4e-02	-7.7e-02	-5.1e-02	< 0.1e-26	2.4e-01
Total lipids (mmol/l)	NMR delay	50	25	-2.3e-02	-3.3e-02	-1.3e-02	3.6e-06	2.4e-01

Metabolic traits	delay	N.obs	N.indiv	Beta	LCI	UCI	Pvalue	SD
Phospholipids (mmol/l)	Buffer delay	50	25	-2.3e-02	-2.8e-02	-1.7e-02	2.2e-16	1.4e-01
Phospholipids (mmol/l)	NMR delay	50	25	-1.3e-02	-1.8e-02	-8.7e-03	6.4e-09	1.4e-01
Total cholesterol (mmol/l)	Buffer delay	50	25	-4.1e-02	-4.8e-02	-3.3e-02	< 0.1e-26	1.0e-01
Total cholesterol (mmol/l)	NMR delay	50	25	-9.2e-03	-1.5e-02	-3.5e-03	1.6e-03	1.0e-01
Cholesterol esters (mmol/l)	Buffer delay	50	25	-2.9e-02	-3.5e-02	-2.4e-02	< 0.1e-26	7.3e-02
Cholesterol esters (mmol/l)	NMR delay	50	25	-6.7e-03	-1.1e-02	-2.6e-03	1.3e-03	7.3e-02
Free cholesterol (mmol/l)	Buffer delay	50	25	-1.1e-02	-1.3e-02	-9.2e-03	< 0.1e-26	3.2e-02
Free cholesterol (mmol/l)	NMR delay	50	25	-2.5e-03	-4.2e-03	-8.4e-04	3.2e-03	3.2e-02
Triglycerides (mmol/l)	Buffer delay	50	25	-6.4e-04	-1.7e-03	3.9e-04	2.2e-01	9.9e-03
Triglycerides (mmol/l)	NMR delay	50	25	-6.0e-04	-1.2e-03	3.4e-05	6.3e-02	9.9e-03
Large HDL								
Particle concentration (mol/l)	Buffer delay	50	25	-2.2e-08	-4.6e-08	3.2e-09	8.7e-02	6.4e-07
Particle concentration (mol/l)	NMR delay	50	25	-6.2e-08	-8.1e-08	-4.3e-08	1.6e-10	6.4e-07
Total lipids (mmol/l)	Buffer delay	50	25	-1.5e-02	-3.1e-02	1.3e-03	7.1e-02	4.1e-01
Total lipids (mmol/l)	NMR delay	50	25	-4.0e-02	-5.2e-02	-2.7e-02	2.2e-10	4.1e-01
Phospholipids (mmol/l)	Buffer delay	50	25	-8.4e-03	-1.7e-02	4.2e-05	5.1e-02	1.8e-01
Phospholipids (mmol/l)	NMR delay	50	25	-1.9e-02	-2.5e-02	-1.3e-02	4.8e-09	1.8e-01
Total cholesterol (mmol/l)	Buffer delay	50	25	-8.3e-03	-1.7e-02	1.8e-04	5.5e-02	2.2e-01
Total cholesterol (mmol/l)	NMR delay	50	25	-2.0e-02	-2.7e-02	-1.3e-02	4.0e-09	2.2e-01
Cholesterol esters (mmol/l)	Buffer delay	50	25	-6.3e-03	-1.3e-02	1.0e-04	5.4e-02	1.7e-01
Cholesterol esters (mmol/l)	NMR delay	50	25	-1.5e-02	-2.0e-02	-1.0e-02	3.5e-09	1.7e-01
Free cholesterol (mmol/l)	Buffer delay	50	25	-2.0e-03	-4.1e-03	1.0e-04	6.2e-02	5.4e-02
Free cholesterol (mmol/l)	NMR delay	50	25	-5.0e-03	-6.7e-03	-3.3e-03	1.1e-08	5.4e-02
Triglycerides (mmol/l)	Buffer delay	50	25	2.0e-03	1.3e-03	2.7e-03	5.9e-09	1.3e-02
Triglycerides (mmol/l)	NMR delay	50	25	-6.9e-04	-1.1e-03	-3.2e-04	3.2e-04	1.3e-02
Medium HDL								
Particle concentration (mol/l)	Buffer delay	50	25	-2.3e-08	-7.6e-08	3.0e-08	3.9e-01	3.7e-07

Metabolic traits	delay	N.obs	N.indiv	Beta	LCI	UCI	Pvalue	SD
Particle concentration (mol/l)	NMR delay	50	25	-3.5e-08	-7.2e-08	7.0e-10	5.5e-02	3.7e-07
Total lipids (mmol/l)	Buffer delay	50	25	-9.4e-03	-3.3e-02	1.4e-02	4.3e-01	1.6e-01
Total lipids (mmol/l)	NMR delay	50	25	-1.5e-02	-3.1e-02	3.5e-04	5.5e-02	1.6e-01
Phospholipids (mmol/l)	Buffer delay	50	25	-7.8e-03	-1.8e-02	2.1e-03	1.2e-01	7.3e-02
Phospholipids (mmol/l)	NMR delay	50	25	-6.9e-03	-1.4e-02	-2.3e-04	4.3e-02	7.3e-02
Total cholesterol (mmol/l)	Buffer delay	50	25	-6.0e-04	-1.4e-02	1.3e-02	9.3e-01	8.9e-02
Total cholesterol (mmol/l)	NMR delay	50	25	-7.9e-03	-1.7e-02	7.7e-04	7.4e-02	8.9e-02
Cholesterol esters (mmol/l)	Buffer delay	50	25	7.3e-04	-9.8e-03	1.1e-02	8.9e-01	6.9e-02
Cholesterol esters (mmol/l)	NMR delay	50	25	-6.2e-03	-1.3e-02	7.7e-04	8.1e-02	6.9e-02
Free cholesterol (mmol/l)	Buffer delay	50	25	-1.3e-03	-4.1e-03	1.4e-03	3.5e-01	2.0e-02
Free cholesterol (mmol/l)	NMR delay	50	25	-1.7e-03	-3.5e-03	5.5e-05	5.8e-02	2.0e-02
Triglycerides (mmol/l)	Buffer delay	50	25	-1.1e-03	-1.7e-03	-4.9e-04	3.4e-04	1.9e-02
Triglycerides (mmol/l)	NMR delay	50	25	-4.1e-04	-1.0e-03	2.2e-04	2.0e-01	1.9e-02
Small HDL								
Particle concentration (mol/l)	Buffer delay	50	25	5.7e-08	-2.7e-08	1.4e-07	1.9e-01	4.7e-07
Particle concentration (mol/l)	NMR delay	50	25	-1.1e-08	-6.8e-08	4.5e-08	7.0e-01	4.7e-07
Total lipids (mmol/l)	Buffer delay	50	25	1.3e-02	-6.0e-03	3.2e-02	1.8e-01	1.0e-01
Total lipids (mmol/l)	NMR delay	50	25	-2.9e-03	-1.6e-02	9.9e-03	6.5e-01	1.0e-01
Phospholipids (mmol/l)	Buffer delay	50	25	-4.6e-03	-1.2e-02	2.5e-03	2.1e-01	7.2e-02
Phospholipids (mmol/l)	NMR delay	50	25	-6.2e-04	-4.6e-03	3.3e-03	7.6e-01	7.2e-02
Total cholesterol (mmol/l)	Buffer delay	50	25	1.8e-02	5.4e-03	3.0e-02	4.8e-03	6.1e-02
Total cholesterol (mmol/l)	NMR delay	50	25	-2.7e-03	-1.2e-02	7.1e-03	5.9e-01	6.1e-02
Cholesterol esters (mmol/l)	Buffer delay	50	25	1.9e-02	8.3e-03	3.0e-02	5.5e-04	6.1e-02
Cholesterol esters (mmol/l)	NMR delay	50	25	-2.3e-03	-1.2e-02	7.1e-03	6.3e-01	6.1e-02
Free cholesterol (mmol/l)	Buffer delay	50	25	-1.6e-03	-3.1e-03	-1.3e-04	3.3e-02	1.2e-02
Free cholesterol (mmol/l)	NMR delay	50	25	-4.0e-04	-1.1e-03	3.0e-04	2.6e-01	1.2e-02
Triglycerides (mmol/l)	Buffer delay	50	25	4.9e-05	-5.3e-04	6.3e-04	8.7e-01	2.2e-02

Metabolic traits	delay	N.obs	N.indiv	Beta	LCI	UCI	Pvalue	SD
Triglycerides (mmol/l)	NMR delay	50	25	4.4e-04	-1.9e-04	1.1e-03	1.7e-01	2.2e-02
**Lipoprotein particle size**								
VLDL particle size (nm)	Buffer delay	50	25	1.0e-01	4.9e-02	1.6e-01	2.0e-04	1.6e+00
VLDL particle size (nm)	NMR delay	50	25	-3.8e-02	-8.2e-02	5.4e-03	8.6e-02	1.6e+00
LDL particle size (nm)	Buffer delay	50	25	-5.2e-02	-7.0e-02	-3.4e-02	9.2e-09	7.7e-02
LDL particle size (nm)	NMR delay	50	25	6.0e-03	-5.7e-03	1.8e-02	3.1e-01	7.7e-02
HDL particle size (nm)	Buffer delay	50	25	-3.6e-02	-5.2e-02	-2.0e-02	6.8e-06	2.8e-01
HDL particle size (nm)	NMR delay	50	25	-2.2e-02	-3.4e-02	-9.2e-03	6.6e-04	2.8e-01
**Cholesterol**								
Total cholesterol (mmol/l)	Buffer delay	50	25	-5.9e-02	-1.2e-01	1.6e-03	5.6e-02	7.3e-01
Total cholesterol (mmol/l)	NMR delay	50	25	-6.1e-02	-1.0e-01	-1.8e-02	5.9e-03	7.3e-01
VLDL cholesterol (mmol/l)	Buffer delay	50	25	-4.7e-02	-6.3e-02	-3.2e-02	2.4e-09	3.8e-01
VLDL cholesterol (mmol/l)	NMR delay	50	25	-4.8e-03	-1.5e-02	5.1e-03	3.4e-01	3.8e-01
Remnant cholesterol (mmol/l)	Buffer delay	50	25	-5.9e-02	-9.0e-02	-2.8e-02	1.9e-04	4.4e-01
Remnant cholesterol (mmol/l)	NMR delay	50	25	-8.3e-03	-2.8e-02	1.1e-02	4.1e-01	4.4e-01
LDL cholesterol (mmol/l)	Buffer delay	50	25	3.2e-02	-2.4e-03	6.7e-02	6.8e-02	3.6e-01
LDL cholesterol (mmol/l)	NMR delay	50	25	-1.3e-02	-3.5e-02	8.5e-03	2.3e-01	3.6e-01
HDL cholesterol (mmol/l)	Buffer delay	50	25	-3.2e-02	-5.7e-02	-7.2e-03	1.1e-02	4.0e-01
HDL cholesterol (mmol/l)	NMR delay	50	25	-4.0e-02	-5.7e-02	-2.3e-02	5.9e-06	4.0e-01
HDL2 cholesterol (mmol/l)	Buffer delay	50	25	-2.4e-02	-4.6e-02	-8.7e-04	4.2e-02	3.6e-01
HDL2 cholesterol (mmol/l)	NMR delay	50	25	-3.6e-02	-5.2e-02	-2.0e-02	6.2e-06	3.6e-01
HDL3 cholesterol (mmol/l)	Buffer delay	50	25	-8.4e-03	-1.1e-02	-5.5e-03	7.3e-09	3.5e-02
HDL3 cholesterol (mmol/l)	NMR delay	50	25	-3.9e-03	-6.1e-03	-1.7e-03	4.6e-04	3.5e-02
Esterified cholesterol (mmol/l)	Buffer delay	42	21	4.0e-02	-1.1e-02	9.2e-02	1.2e-01	5.1e-01
Esterified cholesterol (mmol/l)	NMR delay	42	21	-1.1e-03	-4.2e-02	4.0e-02	9.6e-01	5.1e-01
Free cholesterol (mmol/l)	Buffer delay	42	21	-6.6e-02	-9.2e-02	-4.0e-02	9.1e-07	2.2e-01
Free cholesterol (mmol/l)	NMR delay	42	21	-6.3e-02	-8.4e-02	-4.2e-02	4.8e-09	2.2e-01

Metabolic traits	delay	N.obs	N.indiv	Beta	LCI	UCI	Pvalue	SD
**Glycerides and phospholipids**								
Triglycerides (mmol/l)	Buffer delay	50	25	-6.7e-04	-2.1e-02	2.0e-02	9.5e-01	1.0e+00
Triglycerides (mmol/l)	NMR delay	50	25	-1.0e-02	-2.8e-02	7.3e-03	2.5e-01	1.0e+00
VLDL triglycerides (mmol/l)	Buffer delay	50	25	-7.3e-03	-2.7e-02	1.3e-02	4.7e-01	9.0e-01
VLDL triglycerides (mmol/l)	NMR delay	50	25	-8.2e-03	-2.5e-02	8.6e-03	3.4e-01	9.0e-01
LDL triglycerides (mmol/l)	Buffer delay	50	25	3.5e-03	1.4e-03	5.7e-03	1.1e-03	5.2e-02
LDL triglycerides (mmol/l)	NMR delay	50	25	-1.3e-03	-3.6e-03	9.1e-04	2.5e-01	5.2e-02
HDL triglycerides (mmol/l)	Buffer delay	50	25	3.6e-04	-1.2e-03	1.9e-03	6.4e-01	5.0e-02
HDL triglycerides (mmol/l)	NMR delay	50	25	-1.3e-03	-2.3e-03	-2.1e-04	1.9e-02	5.0e-02
Diacylglycerol (mmol/l)	Buffer delay	40	20	-2.4e-03	-8.1e-03	3.2e-03	4.0e-01	2.3e-02
Diacylglycerol (mmol/l)	NMR delay	40	20	-4.7e-04	-7.0e-03	6.1e-03	8.9e-01	2.3e-02
Phosphoglycerides (mmol/l)	Buffer delay	42	21	-9.3e-02	-1.3e-01	-5.1e-02	1.1e-05	3.7e-01
Phosphoglycerides (mmol/l)	NMR delay	42	21	7.3e-03	-3.5e-02	4.9e-02	7.3e-01	3.7e-01
Phosphatidylcholine + other cholines (mmol/l)	Buffer delay	42	21	-1.1e-01	-1.5e-01	-7.0e-02	6.0e-08	3.5e-01
Phosphatidylcholine + other cholines (mmol/l)	NMR delay	42	21	-4.7e-02	-8.2e-02	-1.2e-02	7.9e-03	3.5e-01
Sphingomyelins (mmol/l)	Buffer delay	42	21	-2.1e-02	-4.0e-02	-9.4e-04	4.0e-02	7.3e-02
Sphingomyelins (mmol/l)	NMR delay	42	21	2.2e-02	2.9e-03	4.1e-02	2.4e-02	7.3e-02
Cholines (mmol/l)	Buffer delay	42	21	-8.2e-02	-1.4e-01	-2.2e-02	7.5e-03	3.7e-01
Cholines (mmol/l)	NMR delay	42	21	5.6e-03	-5.8e-02	6.9e-02	8.6e-01	3.7e-01
**Apolipoproteins**								
Apolipoprotein A-I (g/l)	Buffer delay	50	25	-9.6e-03	-2.3e-02	4.2e-03	1.7e-01	1.9e-01
Apolipoprotein A-I (g/l)	NMR delay	50	25	-2.6e-02	-3.7e-02	-1.6e-02	1.0e-06	1.9e-01
Apolipoprotein B (g/l)	Buffer delay	50	25	-9.6e-04	-1.6e-02	1.4e-02	9.0e-01	2.3e-01
Apolipoprotein B (g/l)	NMR delay	50	25	-3.6e-03	-1.3e-02	5.9e-03	4.6e-01	2.3e-01
**Fatty acids**								
Total fatty acids (mmol/l)	Buffer delay	42	21	-4.5e-02	-2.9e-01	2.0e-01	7.2e-01	3.3e+00
Total fatty acids (mmol/l)	NMR delay	42	21	-1.1e-01	-2.9e-01	6.5e-02	2.2e-01	3.3e+00

Metabolic traits	delay	N.obs	N.indiv	Beta	LCI	UCI	Pvalue	SD
Fatty acid chain length	Buffer delay	42	21	-1.0e-02	-5.0e-02	2.9e-02	6.0e-01	2.8e-01
Fatty acid chain length	NMR delay	42	21	1.1e-01	6.8e-02	1.5e-01	7.8e-08	2.8e-01
Degree of unsaturation	Buffer delay	42	21	-1.9e-02	-3.1e-02	-8.4e-03	5.4e-04	8.0e-02
Degree of unsaturation	NMR delay	42	21	-1.0e-02	-1.6e-02	-4.2e-03	6.6e-04	8.0e-02
Docosahexaenoic acid (mmol/l)	Buffer delay	42	21	-3.7e-03	-7.9e-03	5.7e-04	9.0e-02	4.9e-02
Docosahexaenoic acid (mmol/l)	NMR delay	42	21	-1.4e-03	-4.8e-03	2.0e-03	4.1e-01	4.9e-02
Linoleic acid (mmol/l)	Buffer delay	42	21	-9.0e-03	-6.1e-02	4.3e-02	7.3e-01	6.1e-01
Linoleic acid (mmol/l)	NMR delay	42	21	-2.5e-02	-6.4e-02	1.5e-02	2.2e-01	6.1e-01
Conjugated linoleic acid (mmol/l)	Buffer delay	42	21	-2.7e-04	-6.6e-03	6.0e-03	9.3e-01	2.5e-02
Conjugated linoleic acid (mmol/l)	NMR delay	42	21	6.8e-03	2.3e-03	1.1e-02	2.8e-03	2.5e-02
n-3 fatty acids (mmol/l)	Buffer delay	42	21	1.4e-03	-1.2e-02	1.5e-02	8.4e-01	1.4e-01
n-3 fatty acids (mmol/l)	NMR delay	42	21	-7.3e-03	-1.6e-02	1.1e-03	8.8e-02	1.4e-01
n-6 fatty acids (mmol/l)	Buffer delay	42	21	-4.4e-02	-1.1e-01	2.2e-02	1.9e-01	6.8e-01
n-6 fatty acids (mmol/l)	NMR delay	42	21	-5.0e-02	-1.0e-01	-5.5e-04	4.8e-02	6.8e-01
PUFA (mmol/l)	Buffer delay	42	21	-4.3e-02	-1.2e-01	3.3e-02	2.7e-01	8.0e-01
PUFA (mmol/l)	NMR delay	42	21	-5.8e-02	-1.1e-01	-1.5e-03	4.4e-02	8.0e-01
MUFA (mmol/l)	Buffer delay	42	21	1.0e-02	-5.2e-02	7.3e-02	7.5e-01	1.4e+00
MUFA (mmol/l)	NMR delay	42	21	1.5e-02	-4.0e-02	7.0e-02	6.0e-01	1.4e+00
Saturated fatty acids (mmol/l)	Buffer delay	42	21	-1.2e-02	-1.5e-01	1.3e-01	8.7e-01	1.3e+00
Saturated fatty acids (mmol/l)	NMR delay	42	21	-6.8e-02	-1.5e-01	1.2e-02	9.4e-02	1.3e+00
**Glycolysis related metabolites**								
Glucose (mmol/l)	Buffer delay	50	25	-7.4e-03	-3.7e-02	2.3e-02	6.3e-01	4.9e-01
Glucose (mmol/l)	NMR delay	50	25	-2.5e-02	-5.0e-02	5.5e-04	5.5e-02	4.9e-01
Lactate (mmol/l)	Buffer delay	50	25	2.0e-02	7.6e-04	3.9e-02	4.2e-02	3.9e-01
Lactate (mmol/l)	NMR delay	50	25	9.5e-03	-1.3e-02	3.2e-02	4.0e-01	3.9e-01
Pyruvate (mmol/l)	Buffer delay	74	37	-1.6e-03	-3.2e-03	-1.7e-05	4.8e-02	3.2e-02

Metabolic traits	delay	N.obs	N.indiv	Beta	LCI	UCI	Pvalue	SD
Pyruvate (mmol/l)	NMR delay	74	37	5.7e-04	-8.6e-04	2.0e-03	4.3e-01	3.2e-02
Citrate (mmol/l)	Buffer delay	50	25	-1.3e-03	-1.2e-02	9.3e-03	8.1e-01	2.2e-02
Citrate (mmol/l)	NMR delay	50	25	2.3e-04	-8.2e-03	8.7e-03	9.6e-01	2.2e-02
Glycerol (mmol/l)	Buffer delay	74	37	-1.4e-03	-5.0e-03	2.3e-03	4.6e-01	2.1e-02
Glycerol (mmol/l)	NMR delay	74	37	3.6e-03	1.6e-03	5.5e-03	4.3e-04	2.1e-02
**Amino acids**								
Alanine (mmol/l)	Buffer delay	50	25	-3.0e-03	-6.1e-03	-1.7e-05	4.9e-02	5.5e-02
Alanine (mmol/l)	NMR delay	50	25	5.6e-03	2.9e-03	8.4e-03	6.4e-05	5.5e-02
Glutamine (mmol/l)	Buffer delay	50	25	1.1e-02	4.0e-03	1.8e-02	2.0e-03	5.6e-02
Glutamine (mmol/l)	NMR delay	50	25	-9.4e-03	-1.5e-02	-4.0e-03	6.4e-04	5.6e-02
Histidine (mmol/l)	Buffer delay	50	25	-5.2e-03	-7.9e-03	-2.4e-03	2.2e-04	7.0e-03
Histidine (mmol/l)	NMR delay	50	25	-9.4e-04	-2.7e-03	7.7e-04	2.8e-01	7.0e-03
Glycine (mmol/l)	Buffer delay	74	37	2.3e-02	1.8e-02	2.7e-02	< 0.1e-26	7.6e-02
Glycine (mmol/l)	NMR delay	74	37	2.5e-02	2.1e-02	2.8e-02	< 0.1e-26	7.6e-02
Branched-chain amino acids								
Isoleucine (mmol/l)	Buffer delay	50	25	-1.6e-03	-2.8e-03	-3.4e-04	1.2e-02	2.5e-02
Isoleucine (mmol/l)	NMR delay	50	25	-8.3e-04	-2.4e-03	6.9e-04	2.9e-01	2.5e-02
Leucine (mmol/l)	Buffer delay	50	25	1.5e-04	-7.6e-04	1.1e-03	7.5e-01	2.1e-02
Leucine (mmol/l)	NMR delay	50	25	3.4e-03	2.5e-03	4.4e-03	1.4e-12	2.1e-02
Valine (mmol/l)	Buffer delay	50	25	1.0e-03	-2.0e-04	2.2e-03	1.0e-01	3.5e-02
Valine (mmol/l)	NMR delay	50	25	4.8e-03	3.3e-03	6.2e-03	2.1e-10	3.5e-02
Aromatic amino acids								
Phenylalanine (mmol/l)	Buffer delay	50	25	4.3e-03	2.3e-03	6.3e-03	2.1e-05	8.4e-03
Phenylalanine (mmol/l)	NMR delay	50	25	9.1e-03	7.9e-03	1.0e-02	< 0.1e-26	8.4e-03
Tyrosine (mmol/l)	Buffer delay	50	25	1.4e-03	2.7e-04	2.5e-03	1.4e-02	1.2e-02
Tyrosine (mmol/l)	NMR delay	50	25	4.3e-04	-6.7e-04	1.5e-03	4.4e-01	1.2e-02
**Ketone bodies**								
Acetate (mmol/l)	Buffer delay	50	25	4.9e-03	4.0e-03	5.7e-03	< 0.1e-26	1.1e-02

Metabolic traits	delay	N.obs	N.indiv	Beta	LCI	UCI	Pvalue	SD
Acetate (mmol/l)	NMR delay	50	25	2.4e-03	1.4e-03	3.3e-03	1.1e-06	1.1e-02
Beta-hydroxybutyrate (mmol/l)	Buffer delay	50	25	3.1e-03	1.8e-03	4.5e-03	5.7e-06	1.6e-02
Beta-hydroxybutyrate (mmol/l)	NMR delay	50	25	1.5e-03	1.9e-04	2.8e-03	2.4e-02	1.6e-02
**Fluid balance**								
Creatinine (mmol/l)	Buffer delay	50	25	3.1e-03	1.8e-03	4.3e-03	1.3e-06	9.4e-03
Creatinine (mmol/l)	NMR delay	50	25	1.0e-03	-3.5e-04	2.4e-03	1.4e-01	9.4e-03
Albumin (signal area)	Buffer delay	50	25	-1.7e-05	-1.3e-03	1.3e-03	9.8e-01	4.6e-03
Albumin (signal area)	NMR delay	50	25	-9.5e-04	-1.5e-03	-4.4e-04	2.9e-04	4.6e-03
**Inflammation**								
Glycoprotein acetyls (mmol/l)	Buffer delay	50	25	-9.2e-03	-1.8e-02	-1.7e-04	4.6e-02	3.3e-01
Glycoprotein acetyls (mmol/l)	NMR delay	50	25	-1.2e-02	-2.3e-02	-1.4e-03	2.8e-02	3.3e-01

metabolites MDPI

Table S9. EDTA-plasma, post-storage handling effects (differences in mean levels): mean differences in metabolite concentrations (or trait value) comparing 24 h delays in buffer addition (i.e. Buffer delay) or NMR profiling (i.e. NMR delay) to the reference (no delays), for EDTA-plasma samples. Pyruvate, glycerol and glycine are not quantified in EDTA -plasma samples due to the interfering resonances of EDTA on their signals.

# Associations in Figure S6 are presented in SD-units. These SD point estimate can be obtained by dividing the point estimate (beta) in absolute (clinically meaningful) concentration by the metabolic trait standard deviation (SD), both provided in the below table.

***Abbreviations**: **C**=cholesterol; **IDL**=intermediate-density lipoprotein; **LCI**=lower confidence interval; **LDL**=low-density lipoprotein; **HDL**=high-density lipoprotein; **MUFA**=monounsaturated fatty acids; **N.obs**= number of observations (samples); **N.indiv**=number of individuals; **PUFA**=polyunsaturated fatty acids; **SD**=standard deviation; **UCI**= upper confidence interval; **VLDL**=very-low-density lipoprotein.*

Metabolic traits	delay	N.obs	N.indiv	Beta	LCI	UCI	Pvalue	SD
**Lipoprotein subclasses**								
Extremely large VLDL								
Particle concentration (mol/l)	Buffer delay	50	25	1.0e-11	-1.6e-12	2.2e-11	9.2e-02	2.1e-10
Particle concentration (mol/l)	NMR delay	50	25	9.2e-12	-1.5e-12	2.0e-11	9.1e-02	2.1e-10
Total lipids (mmol/l)	Buffer delay	50	25	2.1e-03	-3.6e-04	4.6e-03	9.4e-02	4.6e-02
Total lipids (mmol/l)	NMR delay	50	25	1.9e-03	-3.5e-04	4.2e-03	9.7e-02	4.6e-02
Phospholipids (mmol/l)	Buffer delay	50	25	2.6e-04	-3.9e-05	5.6e-04	8.9e-02	5.7e-03
Phospholipids (mmol/l)	NMR delay	50	25	2.0e-04	-6.9e-05	4.8e-04	1.4e-01	5.7e-03
Total cholesterol (mmol/l)	Buffer delay	50	25	3.6e-04	-7.1e-05	7.8e-04	1.0e-01	8.5e-03
Total cholesterol (mmol/l)	NMR delay	50	25	2.4e-04	-1.3e-04	6.1e-04	2.0e-01	8.5e-03
Cholesterol esters (mmol/l)	Buffer delay	50	25	2.6e-04	-8.8e-06	5.2e-04	5.8e-02	4.7e-03
Cholesterol esters (mmol/l)	NMR delay	50	25	1.4e-04	-7.8e-05	3.6e-04	2.1e-01	4.7e-03
Free cholesterol (mmol/l)	Buffer delay	50	25	1.0e-04	-6.4e-05	2.7e-04	2.3e-01	3.7e-03
Free cholesterol (mmol/l)	NMR delay	50	25	9.9e-05	-5.9e-05	2.6e-04	2.2e-01	3.7e-03
Triglycerides (mmol/l)	Buffer delay	50	25	1.5e-03	-2.6e-04	3.3e-03	9.4e-02	3.2e-02
Triglycerides (mmol/l)	NMR delay	50	25	1.5e-03	-1.6e-04	3.1e-03	7.7e-02	3.2e-02
Very large VLDL								
Particle concentration (mol/l)	Buffer delay	50	25	1.1e-11	-2.2e-11	4.4e-11	5.2e-01	1.3e-09
Particle concentration (mol/l)	NMR delay	50	25	2.9e-11	-1.4e-11	7.2e-11	1.8e-01	1.3e-09
Total lipids (mmol/l)	Buffer delay	50	25	1.1e-03	-2.2e-03	4.5e-03	5.1e-01	1.2e-01

Metabolic traits	delay	N.obs	N.indiv	Beta	LCI	UCI	Pvalue	SD
Total lipids (mmol/l)	NMR delay	50	25	2.8e-03	-1.3e-03	6.9e-03	1.8e-01	1.2e-01
Phospholipids (mmol/l)	Buffer delay	50	25	1.1e-04	-4.9e-04	7.0e-04	7.3e-01	2.0e-02
Phospholipids (mmol/l)	NMR delay	50	25	4.2e-04	-2.0e-04	1.0e-03	1.9e-01	2.0e-02
Total cholesterol (mmol/l)	Buffer delay	50	25	5.2e-04	-5.0e-04	1.5e-03	3.2e-01	2.5e-02
Total cholesterol (mmol/l)	NMR delay	50	25	5.7e-04	-3.6e-04	1.5e-03	2.3e-01	2.5e-02
Cholesterol esters (mmol/l)	Buffer delay	50	25	4.2e-04	-1.5e-04	9.9e-04	1.5e-01	1.4e-02
Cholesterol esters (mmol/l)	NMR delay	50	25	3.2e-04	-1.9e-04	8.3e-04	2.2e-01	1.4e-02
Free cholesterol (mmol/l)	Buffer delay	50	25	9.6e-05	-3.5e-04	5.5e-04	6.7e-01	1.1e-02
Free cholesterol (mmol/l)	NMR delay	50	25	2.6e-04	-1.7e-04	6.8e-04	2.4e-01	1.1e-02
Triglycerides (mmol/l)	Buffer delay	50	25	5.0e-04	-1.3e-03	2.3e-03	5.8e-01	7.8e-02
Triglycerides (mmol/l)	NMR delay	50	25	1.8e-03	-9.2e-04	4.6e-03	1.9e-01	7.8e-02
Large VLDL								
Particle concentration (mol/l)	Buffer delay	50	25	2.3e-10	6.4e-11	3.9e-10	6.6e-03	7.1e-09
Particle concentration (mol/l)	NMR delay	50	25	1.2e-10	-5.5e-11	3.0e-10	1.8e-01	7.1e-09
Total lipids (mmol/l)	Buffer delay	50	25	1.3e-02	3.3e-03	2.2e-02	8.3e-03	4.1e-01
Total lipids (mmol/l)	NMR delay	50	25	7.2e-03	-3.0e-03	1.7e-02	1.7e-01	4.1e-01
Phospholipids (mmol/l)	Buffer delay	50	25	2.3e-03	5.7e-04	4.0e-03	8.9e-03	7.4e-02
Phospholipids (mmol/l)	NMR delay	50	25	1.3e-03	-5.3e-04	3.1e-03	1.7e-01	7.4e-02
Total cholesterol (mmol/l)	Buffer delay	50	25	1.8e-03	-7.3e-04	4.3e-03	1.6e-01	9.6e-02
Total cholesterol (mmol/l)	NMR delay	50	25	2.2e-03	-3.6e-04	4.7e-03	9.2e-02	9.6e-02
Cholesterol esters (mmol/l)	Buffer delay	50	25	7.5e-04	-6.5e-04	2.2e-03	2.9e-01	4.8e-02
Cholesterol esters (mmol/l)	NMR delay	50	25	1.4e-03	1.7e-05	2.7e-03	4.7e-02	4.8e-02
Free cholesterol (mmol/l)	Buffer delay	50	25	1.0e-03	-1.2e-04	2.2e-03	8.0e-02	4.8e-02
Free cholesterol (mmol/l)	NMR delay	50	25	8.2e-04	-4.1e-04	2.1e-03	1.9e-01	4.8e-02
Triglycerides (mmol/l)	Buffer delay	50	25	8.7e-03	2.9e-03	1.4e-02	3.2e-03	2.4e-01
Triglycerides (mmol/l)	NMR delay	50	25	3.7e-03	-2.4e-03	9.7e-03	2.3e-01	2.4e-01
Medium VLDL								

Metabolic traits	delay	N.obs	N.indiv	Beta	LCI	UCI	Pvalue	SD
Particle concentration (mol/l)	Buffer delay	50	25	5.6e-10	1.7e-10	9.5e-10	4.6e-03	1.8e-08
Particle concentration (mol/l)	NMR delay	50	25	3.0e-10	-8.4e-11	6.9e-10	1.3e-01	1.8e-08
Total lipids (mmol/l)	Buffer delay	50	25	1.7e-02	4.4e-03	3.1e-02	8.6e-03	5.8e-01
Total lipids (mmol/l)	NMR delay	50	25	1.1e-02	-2.5e-03	2.4e-02	1.1e-01	5.8e-01
Phospholipids (mmol/l)	Buffer delay	50	25	3.4e-03	8.7e-04	5.9e-03	8.2e-03	1.1e-01
Phospholipids (mmol/l)	NMR delay	50	25	2.1e-03	-3.6e-04	4.6e-03	9.4e-02	1.1e-01
Total cholesterol (mmol/l)	Buffer delay	50	25	2.6e-04	-3.6e-03	4.1e-03	8.9e-01	1.5e-01
Total cholesterol (mmol/l)	NMR delay	50	25	3.9e-03	3.3e-05	7.7e-03	4.8e-02	1.5e-01
Cholesterol esters (mmol/l)	Buffer delay	50	25	-2.2e-03	-4.6e-03	1.3e-04	6.4e-02	7.5e-02
Cholesterol esters (mmol/l)	NMR delay	50	25	2.4e-03	1.7e-04	4.5e-03	3.4e-02	7.5e-02
Free cholesterol (mmol/l)	Buffer delay	50	25	2.5e-03	8.5e-04	4.2e-03	3.0e-03	7.2e-02
Free cholesterol (mmol/l)	NMR delay	50	25	1.5e-03	-2.7e-04	3.3e-03	9.6e-02	7.2e-02
Triglycerides (mmol/l)	Buffer delay	50	25	1.4e-02	6.9e-03	2.1e-02	1.0e-04	3.2e-01
Triglycerides (mmol/l)	NMR delay	50	25	4.6e-03	-2.4e-03	1.2e-02	2.0e-01	3.2e-01
Small VLDL								
Particle concentration (mol/l)	Buffer delay	50	25	-1.1e-09	-1.6e-09	-6.4e-10	3.2e-06	1.7e-08
Particle concentration (mol/l)	NMR delay	50	25	-1.5e-10	-5.0e-10	2.1e-10	4.2e-01	1.7e-08
Total lipids (mmol/l)	Buffer delay	50	25	-2.5e-02	-3.4e-02	-1.6e-02	9.0e-08	3.1e-01
Total lipids (mmol/l)	NMR delay	50	25	-3.2e-03	-1.0e-02	4.0e-03	3.8e-01	3.1e-01
Phospholipids (mmol/l)	Buffer delay	50	25	-8.3e-03	-1.1e-02	-5.9e-03	1.3e-11	6.2e-02
Phospholipids (mmol/l)	NMR delay	50	25	-2.3e-03	-4.5e-03	-1.7e-04	3.4e-02	6.2e-02
Total cholesterol (mmol/l)	Buffer delay	50	25	-1.7e-02	-2.2e-02	-1.3e-02	< 0.1e-26	8.7e-02
Total cholesterol (mmol/l)	NMR delay	50	25	-7.8e-04	-4.6e-03	3.1e-03	6.9e-01	8.7e-02
Cholesterol esters (mmol/l)	Buffer delay	50	25	-1.3e-02	-1.6e-02	-1.1e-02	< 0.1e-26	4.9e-02
Cholesterol esters (mmol/l)	NMR delay	50	25	-1.8e-04	-3.2e-03	2.8e-03	9.1e-01	4.9e-02
Free cholesterol (mmol/l)	Buffer delay	50	25	-3.9e-03	-5.3e-03	-2.5e-03	3.8e-08	4.0e-02
Free cholesterol (mmol/l)	NMR delay	50	25	-5.9e-04	-1.6e-03	4.3e-04	2.6e-01	4.0e-02

Metabolic traits	delay	N.obs	N.indiv	Beta	LCI	UCI	Pvalue	SD
Triglycerides (mmol/l)	Buffer delay	50	25	6.3e-04	-3.3e-03	4.5e-03	7.5e-01	1.7e-01
Triglycerides (mmol/l)	NMR delay	50	25	-7.8e-05	-3.0e-03	2.9e-03	9.6e-01	1.7e-01
Very Small VLDL								
Particle concentration (mol/l)	Buffer delay	50	25	-2.0e-09	-2.6e-09	-1.5e-09	1.2e-13	9.2e-09
Particle concentration (mol/l)	NMR delay	50	25	3.1e-10	-2.1e-10	8.3e-10	2.4e-01	9.2e-09
Total lipids (mmol/l)	Buffer delay	50	25	-2.7e-02	-3.4e-02	-2.0e-02	5.8e-14	1.1e-01
Total lipids (mmol/l)	NMR delay	50	25	5.1e-03	-1.8e-03	1.2e-02	1.5e-01	1.1e-01
Phospholipids (mmol/l)	Buffer delay	50	25	-1.6e-03	-3.9e-03	6.9e-04	1.7e-01	3.0e-02
Phospholipids (mmol/l)	NMR delay	50	25	-2.7e-04	-2.3e-03	1.8e-03	7.9e-01	3.0e-02
Total cholesterol (mmol/l)	Buffer delay	50	25	-2.3e-02	-2.7e-02	-1.9e-02	< 0.1e-26	4.6e-02
Total cholesterol (mmol/l)	NMR delay	50	25	6.6e-03	1.6e-03	1.2e-02	9.2e-03	4.6e-02
Cholesterol esters (mmol/l)	Buffer delay	50	25	-2.1e-02	-2.5e-02	-1.8e-02	< 0.1e-26	3.3e-02
Cholesterol esters (mmol/l)	NMR delay	50	25	5.7e-03	1.7e-03	9.6e-03	4.9e-03	3.3e-02
Free cholesterol (mmol/l)	Buffer delay	50	25	-1.7e-03	-3.0e-03	-4.8e-04	6.5e-03	1.4e-02
Free cholesterol (mmol/l)	NMR delay	50	25	9.4e-04	-1.6e-04	2.0e-03	9.5e-02	1.4e-02
Triglycerides (mmol/l)	Buffer delay	50	25	-2.4e-03	-4.2e-03	-5.1e-04	1.2e-02	4.6e-02
Triglycerides (mmol/l)	NMR delay	50	25	-1.3e-03	-2.6e-03	1.3e-04	7.6e-02	4.6e-02
IDL								
Particle concentration (mol/l)	Buffer delay	50	25	-4.5e-10	-1.9e-09	1.0e-09	5.6e-01	2.0e-08
Particle concentration (mol/l)	NMR delay	50	25	2.0e-09	3.0e-10	3.7e-09	2.1e-02	2.0e-08
Total lipids (mmol/l)	Buffer delay	50	25	-3.2e-03	-1.9e-02	1.3e-02	7.0e-01	2.0e-01
Total lipids (mmol/l)	NMR delay	50	25	2.2e-02	4.0e-03	4.1e-02	1.7e-02	2.0e-01
Phospholipids (mmol/l)	Buffer delay	50	25	-3.7e-04	-3.8e-03	3.1e-03	8.3e-01	4.9e-02
Phospholipids (mmol/l)	NMR delay	50	25	3.2e-03	-4.8e-04	6.9e-03	8.8e-02	4.9e-02
Total cholesterol (mmol/l)	Buffer delay	50	25	-1.7e-03	-1.5e-02	1.1e-02	7.9e-01	1.3e-01
Total cholesterol (mmol/l)	NMR delay	50	25	2.1e-02	5.6e-03	3.6e-02	7.4e-03	1.3e-01
Cholesterol esters (mmol/l)	Buffer delay	50	25	-3.6e-03	-1.4e-02	6.4e-03	4.8e-01	1.0e-01

Metabolic traits	delay	N.obs	N.indiv	Beta	LCI	UCI	Pvalue	SD
Cholesterol esters (mmol/l)	NMR delay	50	25	1.7e-02	4.9e-03	2.9e-02	5.6e-03	1.0e-01
Free cholesterol (mmol/l)	Buffer delay	50	25	1.9e-03	-1.3e-03	5.0e-03	2.4e-01	3.9e-02
Free cholesterol (mmol/l)	NMR delay	50	25	4.2e-03	6.3e-04	7.7e-03	2.1e-02	3.9e-02
Triglycerides (mmol/l)	Buffer delay	50	25	-1.1e-03	-3.0e-03	8.1e-04	2.6e-01	3.1e-02
Triglycerides (mmol/l)	NMR delay	50	25	-1.8e-03	-3.3e-03	-2.7e-04	2.1e-02	3.1e-02
Large LDL								
Particle concentration (mol/l)	Buffer delay	50	25	1.8e-09	-5.5e-10	4.2e-09	1.3e-01	3.4e-08
Particle concentration (mol/l)	NMR delay	50	25	1.9e-09	-5.4e-10	4.3e-09	1.3e-01	3.4e-08
Total lipids (mmol/l)	Buffer delay	50	25	1.4e-02	-2.9e-03	3.2e-02	1.0e-01	2.4e-01
Total lipids (mmol/l)	NMR delay	50	25	1.5e-02	-2.6e-03	3.3e-02	9.5e-02	2.4e-01
Phospholipids (mmol/l)	Buffer delay	50	25	9.2e-04	-2.5e-03	4.4e-03	6.0e-01	5.1e-02
Phospholipids (mmol/l)	NMR delay	50	25	2.7e-03	-9.4e-04	6.4e-03	1.5e-01	5.1e-02
Total cholesterol (mmol/l)	Buffer delay	50	25	1.6e-02	2.2e-03	3.0e-02	2.3e-02	1.8e-01
Total cholesterol (mmol/l)	NMR delay	50	25	1.5e-02	2.0e-04	2.9e-02	4.7e-02	1.8e-01
Cholesterol esters (mmol/l)	Buffer delay	50	25	1.4e-02	3.5e-03	2.4e-02	8.5e-03	1.4e-01
Cholesterol esters (mmol/l)	NMR delay	50	25	1.1e-02	4.0e-05	2.2e-02	4.9e-02	1.4e-01
Free cholesterol (mmol/l)	Buffer delay	50	25	2.1e-03	-1.3e-03	5.5e-03	2.2e-01	4.5e-02
Free cholesterol (mmol/l)	NMR delay	50	25	3.8e-03	2.2e-04	7.3e-03	3.7e-02	4.5e-02
Triglycerides (mmol/l)	Buffer delay	50	25	-2.5e-03	-4.5e-03	-5.3e-04	1.3e-02	2.5e-02
Triglycerides (mmol/l)	NMR delay	50	25	-2.4e-03	-4.1e-03	-7.6e-04	4.2e-03	2.5e-02
Medium LDL								
Particle concentration (mol/l)	Buffer delay	50	25	4.9e-09	2.9e-09	6.9e-09	1.4e-06	2.9e-08
Particle concentration (mol/l)	NMR delay	50	25	6.7e-10	-1.3e-09	2.6e-09	5.0e-01	2.9e-08
Total lipids (mmol/l)	Buffer delay	50	25	2.5e-02	1.5e-02	3.6e-02	1.1e-06	1.5e-01
Total lipids (mmol/l)	NMR delay	50	25	4.6e-03	-5.6e-03	1.5e-02	3.7e-01	1.5e-01
Phospholipids (mmol/l)	Buffer delay	50	25	2.2e-03	2.7e-04	4.1e-03	2.6e-02	3.5e-02
Phospholipids (mmol/l)	NMR delay	50	25	1.2e-03	-8.8e-04	3.3e-03	2.5e-01	3.5e-02

metabolites MDPI

Metabolic traits	delay	N.obs	N.indiv	Beta	LCI	UCI	Pvalue	SD
Total cholesterol (mmol/l)	Buffer delay	50	25	2.4e-02	1.6e-02	3.2e-02	6.3e-09	1.1e-01
Total cholesterol (mmol/l)	NMR delay	50	25	5.7e-03	-2.5e-03	1.4e-02	1.7e-01	1.1e-01
Cholesterol esters (mmol/l)	Buffer delay	50	25	2.2e-02	1.5e-02	2.9e-02	9.7e-11	9.0e-02
Cholesterol esters (mmol/l)	NMR delay	50	25	4.5e-03	-2.1e-03	1.1e-02	1.8e-01	9.0e-02
Free cholesterol (mmol/l)	Buffer delay	50	25	2.3e-03	7.3e-04	3.9e-03	4.0e-03	2.1e-02
Free cholesterol (mmol/l)	NMR delay	50	25	1.2e-03	-4.6e-04	2.9e-03	1.5e-01	2.1e-02
Triglycerides (mmol/l)	Buffer delay	50	25	-9.7e-04	-2.4e-03	4.5e-04	1.8e-01	1.3e-02
Triglycerides (mmol/l)	NMR delay	50	25	-2.3e-03	-3.7e-03	-9.9e-04	6.7e-04	1.3e-02
Small LDL								
Particle concentration (mol/l)	Buffer delay	50	25	6.3e-09	4.1e-09	8.5e-09	1.7e-08	3.4e-08
Particle concentration (mol/l)	NMR delay	50	25	2.7e-10	-2.0e-09	2.6e-09	8.2e-01	3.4e-08
Total lipids (mmol/l)	Buffer delay	50	25	1.8e-02	1.2e-02	2.4e-02	1.7e-08	9.5e-02
Total lipids (mmol/l)	NMR delay	50	25	1.4e-03	-5.1e-03	8.0e-03	6.7e-01	9.5e-02
Phospholipids (mmol/l)	Buffer delay	50	25	2.6e-03	1.3e-03	3.9e-03	9.3e-05	2.4e-02
Phospholipids (mmol/l)	NMR delay	50	25	2.3e-05	-1.6e-03	1.6e-03	9.8e-01	2.4e-02
Total cholesterol (mmol/l)	Buffer delay	50	25	1.5e-02	1.1e-02	2.0e-02	3.1e-10	6.9e-02
Total cholesterol (mmol/l)	NMR delay	50	25	2.5e-03	-2.5e-03	7.5e-03	3.2e-01	6.9e-02
Cholesterol esters (mmol/l)	Buffer delay	50	25	1.4e-02	9.8e-03	1.8e-02	4.3e-12	5.6e-02
Cholesterol esters (mmol/l)	NMR delay	50	25	1.9e-03	-2.0e-03	5.8e-03	3.3e-01	5.6e-02
Free cholesterol (mmol/l)	Buffer delay	50	25	1.7e-03	7.8e-04	2.7e-03	4.1e-04	1.4e-02
Free cholesterol (mmol/l)	NMR delay	50	25	6.1e-04	-5.1e-04	1.7e-03	2.9e-01	1.4e-02
Triglycerides (mmol/l)	Buffer delay	50	25	-1.0e-04	-7.3e-04	5.3e-04	7.5e-01	1.2e-02
Triglycerides (mmol/l)	NMR delay	50	25	-1.1e-03	-1.7e-03	-5.4e-04	1.5e-04	1.2e-02
Very large HDL								
Particle concentration (mol/l)	Buffer delay	50	25	-4.7e-08	-5.6e-08	-3.7e-08	< 0.1e-26	2.4e-07
Particle concentration (mol/l)	NMR delay	50	25	-4.3e-09	-1.6e-08	7.3e-09	4.7e-01	2.4e-07
Total lipids (mmol/l)	Buffer delay	50	25	-5.1e-02	-6.0e-02	-4.1e-02	< 0.1e-26	2.4e-01

Metabolic traits	delay	N.obs	N.indiv	Beta	LCI	UCI	Pvalue	SD
Total lipids (mmol/l)	NMR delay	50	25	-3.7e-03	-1.6e-02	8.2e-03	5.4e-01	2.4e-01
Phospholipids (mmol/l)	Buffer delay	50	25	-2.1e-02	-2.6e-02	-1.6e-02	6.7e-16	1.4e-01
Phospholipids (mmol/l)	NMR delay	50	25	-8.1e-03	-1.3e-02	-3.1e-03	1.5e-03	1.4e-01
Total cholesterol (mmol/l)	Buffer delay	50	25	-3.2e-02	-3.7e-02	-2.6e-02	< 0.1e-26	1.1e-01
Total cholesterol (mmol/l)	NMR delay	50	25	3.2e-03	-4.3e-03	1.1e-02	4.0e-01	1.1e-01
Cholesterol esters (mmol/l)	Buffer delay	50	25	-2.2e-02	-2.6e-02	-1.8e-02	< 0.1e-26	7.4e-02
Cholesterol esters (mmol/l)	NMR delay	50	25	2.8e-03	-2.8e-03	8.4e-03	3.3e-01	7.4e-02
Free cholesterol (mmol/l)	Buffer delay	50	25	-9.5e-03	-1.1e-02	-8.0e-03	< 0.1e-26	3.2e-02
Free cholesterol (mmol/l)	NMR delay	50	25	4.0e-04	-1.6e-03	2.4e-03	6.9e-01	3.2e-02
Triglycerides (mmol/l)	Buffer delay	50	25	1.6e-03	5.0e-04	2.7e-03	4.2e-03	1.0e-02
Triglycerides (mmol/l)	NMR delay	50	25	1.2e-03	-6.4e-05	2.5e-03	6.2e-02	1.0e-02
Large HDL								
Particle concentration (mol/l)	Buffer delay	50	25	3.1e-08	-6.4e-09	6.8e-08	1.1e-01	6.5e-07
Particle concentration (mol/l)	NMR delay	50	25	-3.6e-08	-7.3e-08	1.6e-09	6.1e-02	6.5e-07
Total lipids (mmol/l)	Buffer delay	50	25	1.9e-02	-3.6e-03	4.1e-02	1.0e-01	4.2e-01
Total lipids (mmol/l)	NMR delay	50	25	-2.3e-02	-4.6e-02	-1.0e-03	4.0e-02	4.2e-01
Phospholipids (mmol/l)	Buffer delay	50	25	5.4e-04	-1.1e-02	1.3e-02	9.3e-01	1.9e-01
Phospholipids (mmol/l)	NMR delay	50	25	-1.7e-02	-2.9e-02	-4.8e-03	6.1e-03	1.9e-01
Total cholesterol (mmol/l)	Buffer delay	50	25	1.3e-02	3.2e-03	2.2e-02	8.4e-03	2.2e-01
Total cholesterol (mmol/l)	NMR delay	50	25	-8.7e-03	-1.9e-02	1.1e-03	8.1e-02	2.2e-01
Cholesterol esters (mmol/l)	Buffer delay	50	25	1.1e-02	2.8e-03	1.9e-02	8.3e-03	1.7e-01
Cholesterol esters (mmol/l)	NMR delay	50	25	-5.2e-03	-1.4e-02	3.0e-03	2.1e-01	1.7e-01
Free cholesterol (mmol/l)	Buffer delay	50	25	1.9e-03	1.3e-04	3.6e-03	3.6e-02	5.5e-02
Free cholesterol (mmol/l)	NMR delay	50	25	-3.5e-03	-5.2e-03	-1.8e-03	8.2e-05	5.5e-02
Triglycerides (mmol/l)	Buffer delay	50	25	5.3e-03	2.1e-03	8.6e-03	1.3e-03	1.4e-02
Triglycerides (mmol/l)	NMR delay	50	25	2.2e-03	-1.1e-03	5.4e-03	1.9e-01	1.4e-02
Medium HDL								

Metabolic traits	delay	N.obs	N.indiv	Beta	LCI	UCI	Pvalue	SD
Particle concentration (mol/l)	Buffer delay	50	25	-6.0e-08	-1.1e-07	-7.2e-09	2.6e-02	3.7e-07
Particle concentration (mol/l)	NMR delay	50	25	-8.8e-08	-1.5e-07	-3.1e-08	2.4e-03	3.7e-07
Total lipids (mmol/l)	Buffer delay	50	25	-2.4e-02	-4.7e-02	-1.6e-03	3.6e-02	1.6e-01
Total lipids (mmol/l)	NMR delay	50	25	-3.8e-02	-6.3e-02	-1.3e-02	2.5e-03	1.6e-01
Phospholipids (mmol/l)	Buffer delay	50	25	-1.7e-02	-2.8e-02	-7.0e-03	9.9e-04	7.4e-02
Phospholipids (mmol/l)	NMR delay	50	25	-1.8e-02	-2.9e-02	-7.0e-03	1.4e-03	7.4e-02
Total cholesterol (mmol/l)	Buffer delay	50	25	-4.3e-03	-1.7e-02	8.0e-03	4.9e-01	8.9e-02
Total cholesterol (mmol/l)	NMR delay	50	25	-1.9e-02	-3.2e-02	-5.6e-03	5.3e-03	8.9e-02
Cholesterol esters (mmol/l)	Buffer delay	50	25	-1.3e-03	-1.1e-02	8.4e-03	7.9e-01	6.9e-02
Cholesterol esters (mmol/l)	NMR delay	50	25	-1.4e-02	-2.5e-02	-4.0e-03	6.6e-03	6.9e-02
Free cholesterol (mmol/l)	Buffer delay	50	25	-3.0e-03	-5.7e-03	-3.0e-04	3.0e-02	2.0e-02
Free cholesterol (mmol/l)	NMR delay	50	25	-4.5e-03	-7.4e-03	-1.6e-03	2.5e-03	2.0e-02
Triglycerides (mmol/l)	Buffer delay	50	25	-2.6e-03	-3.3e-03	-2.0e-03	4.2e-14	1.7e-02
Triglycerides (mmol/l)	NMR delay	50	25	-1.1e-03	-1.8e-03	-4.4e-04	1.2e-03	1.7e-02
Small HDL								
Particle concentration (mol/l)	Buffer delay	50	25	-6.8e-08	-1.6e-07	2.8e-08	1.6e-01	5.0e-07
Particle concentration (mol/l)	NMR delay	50	25	-1.4e-07	-2.5e-07	-3.8e-08	7.7e-03	5.0e-07
Total lipids (mmol/l)	Buffer delay	50	25	-1.5e-02	-3.7e-02	6.9e-03	1.8e-01	1.1e-01
Total lipids (mmol/l)	NMR delay	50	25	-3.3e-02	-5.7e-02	-8.5e-03	8.1e-03	1.1e-01
Phospholipids (mmol/l)	Buffer delay	50	25	-6.1e-03	-1.2e-02	7.7e-05	5.3e-02	7.2e-02
Phospholipids (mmol/l)	NMR delay	50	25	-7.7e-03	-1.4e-02	-1.7e-03	1.1e-02	7.2e-02
Total cholesterol (mmol/l)	Buffer delay	50	25	-8.1e-03	-2.5e-02	8.4e-03	3.4e-01	6.7e-02
Total cholesterol (mmol/l)	NMR delay	50	25	-2.4e-02	-4.3e-02	-5.9e-03	9.8e-03	6.7e-02
Cholesterol esters (mmol/l)	Buffer delay	50	25	-6.8e-03	-2.2e-02	8.6e-03	3.9e-01	6.5e-02
Cholesterol esters (mmol/l)	NMR delay	50	25	-2.3e-02	-4.0e-02	-5.2e-03	1.1e-02	6.5e-02
Free cholesterol (mmol/l)	Buffer delay	50	25	-1.3e-03	-2.6e-03	9.8e-05	6.9e-02	1.2e-02
Free cholesterol (mmol/l)	NMR delay	50	25	-1.8e-03	-3.0e-03	-5.6e-04	4.5e-03	1.2e-02

Metabolic traits	delay	N.obs	N.indiv	Beta	LCI	UCI	Pvalue	SD
Triglycerides (mmol/l)	Buffer delay	50	25	-1.0e-03	-1.8e-03	-2.1e-04	1.4e-02	2.0e-02
Triglycerides (mmol/l)	NMR delay	50	25	-6.0e-04	-1.2e-03	5.2e-05	7.1e-02	2.0e-02
**Lipoprotein particle size**								
VLDL particle size (nm)	Buffer delay	50	25	3.0e-01	2.1e-01	3.9e-01	2.0e-10	1.6e+00
VLDL particle size (nm)	NMR delay	50	25	9.0e-02	-1.2e-02	1.9e-01	8.3e-02	1.6e+00
LDL particle size (nm)	Buffer delay	50	25	-6.7e-02	-8.7e-02	-4.6e-02	2.0e-10	8.8e-02
LDL particle size (nm)	NMR delay	50	25	2.4e-02	5.2e-03	4.4e-02	1.3e-02	8.8e-02
HDL particle size (nm)	Buffer delay	50	25	-1.1e-02	-2.4e-02	1.5e-03	8.3e-02	2.9e-01
HDL particle size (nm)	NMR delay	50	25	2.1e-03	-1.4e-02	1.8e-02	8.0e-01	2.9e-01
**Cholesterol**								
Total cholesterol (mmol/l)	Buffer delay	50	25	-1.6e-02	-5.8e-02	2.5e-02	4.4e-01	7.3e-01
Total cholesterol (mmol/l)	NMR delay	50	25	6.4e-03	-4.0e-02	5.3e-02	7.9e-01	7.3e-01
VLDL cholesterol (mmol/l)	Buffer delay	50	25	-3.7e-02	-4.9e-02	-2.6e-02	1.6e-10	3.9e-01
VLDL cholesterol (mmol/l)	NMR delay	50	25	1.3e-02	1.5e-03	2.4e-02	2.7e-02	3.9e-01
Remnant cholesterol (mmol/l)	Buffer delay	50	25	-3.9e-02	-6.2e-02	-1.6e-02	7.8e-04	4.6e-01
Remnant cholesterol (mmol/l)	NMR delay	50	25	3.4e-02	8.1e-03	5.9e-02	9.8e-03	4.6e-01
LDL cholesterol (mmol/l)	Buffer delay	50	25	5.6e-02	3.0e-02	8.2e-02	3.1e-05	3.6e-01
LDL cholesterol (mmol/l)	NMR delay	50	25	2.3e-02	-4.2e-03	5.1e-02	9.6e-02	3.6e-01
HDL cholesterol (mmol/l)	Buffer delay	50	25	-3.3e-02	-5.8e-02	-8.0e-03	9.7e-03	3.9e-01
HDL cholesterol (mmol/l)	NMR delay	50	25	-5.0e-02	-7.5e-02	-2.6e-02	4.5e-05	3.9e-01
HDL2 cholesterol (mmol/l)	Buffer delay	50	25	-2.3e-02	-4.5e-02	-1.5e-03	3.6e-02	3.6e-01
HDL2 cholesterol (mmol/l)	NMR delay	50	25	-4.4e-02	-6.5e-02	-2.3e-02	5.0e-05	3.6e-01
HDL3 cholesterol (mmol/l)	Buffer delay	50	25	-9.5e-03	-1.4e-02	-5.1e-03	2.1e-05	3.6e-02
HDL3 cholesterol (mmol/l)	NMR delay	50	25	-6.6e-03	-1.0e-02	-2.9e-03	4.6e-04	3.6e-02
Esterified cholesterol (mmol/l)	Buffer delay	42	21	-2.4e-02	-6.7e-02	1.9e-02	2.7e-01	5.2e-01
Esterified cholesterol (mmol/l)	NMR delay	42	21	1.3e-02	-2.9e-02	5.5e-02	5.4e-01	5.2e-01
Free cholesterol (mmol/l)	Buffer delay	42	21	-7.5e-03	-2.5e-02	1.0e-02	4.1e-01	2.2e-01

Metabolic traits	delay	N.obs	N.indiv	Beta	LCI	UCI	Pvalue	SD
Free cholesterol (mmol/l)	NMR delay	42	21	-7.1e-03	-2.8e-02	1.4e-02	5.1e-01	2.2e-01
**Glycerides and phospholipids**								
Triglycerides (mmol/l)	Buffer delay	50	25	1.9e-02	1.3e-03	3.6e-02	3.6e-02	9.8e-01
Triglycerides (mmol/l)	NMR delay	50	25	1.3e-03	-1.5e-02	1.8e-02	8.8e-01	9.8e-01
VLDL triglycerides (mmol/l)	Buffer delay	50	25	2.2e-02	4.6e-03	3.9e-02	1.3e-02	8.8e-01
VLDL triglycerides (mmol/l)	NMR delay	50	25	8.8e-03	-8.3e-03	2.6e-02	3.1e-01	8.8e-01
LDL triglycerides (mmol/l)	Buffer delay	50	25	-3.6e-03	-7.6e-03	4.0e-04	7.8e-02	4.9e-02
LDL triglycerides (mmol/l)	NMR delay	50	25	-5.9e-03	-9.4e-03	-2.3e-03	1.2e-03	4.9e-02
HDL triglycerides (mmol/l)	Buffer delay	50	25	1.7e-03	-1.2e-05	3.4e-03	5.2e-02	4.8e-02
HDL triglycerides (mmol/l)	NMR delay	50	25	7.1e-05	-1.8e-03	1.9e-03	9.4e-01	4.8e-02
Diacylglycerol (mmol/l)	Buffer delay	40	20	-9.9e-04	-7.4e-03	5.4e-03	7.6e-01	2.6e-02
Diacylglycerol (mmol/l)	NMR delay	40	20	1.8e-03	-2.5e-03	6.2e-03	4.1e-01	2.6e-02
Phosphoglycerides (mmol/l)	Buffer delay	42	21	-5.7e-02	-1.0e-01	-1.2e-02	1.3e-02	3.8e-01
Phosphoglycerides (mmol/l)	NMR delay	42	21	-5.6e-02	-1.2e-01	3.6e-03	6.5e-02	3.8e-01
Phosphatidylcholine + other cholines (mmol/l)	Buffer delay	42	21	-1.8e-02	-6.1e-02	2.6e-02	4.3e-01	3.6e-01
Phosphatidylcholine + other cholines (mmol/l)	NMR delay	42	21	-4.7e-02	-1.0e-01	9.2e-03	1.0e-01	3.6e-01
Sphingomyelins (mmol/l)	Buffer delay	42	21	-3.2e-02	-5.4e-02	-1.0e-02	4.0e-03	8.1e-02
Sphingomyelins (mmol/l)	NMR delay	42	21	-1.4e-02	-3.8e-02	9.8e-03	2.5e-01	8.1e-02
Cholines (mmol/l)	Buffer delay	42	21	-1.4e-02	-6.5e-02	3.6e-02	5.7e-01	3.8e-01
Cholines (mmol/l)	NMR delay	42	21	-6.1e-02	-1.2e-01	2.4e-03	5.9e-02	3.8e-01
**Apolipoproteins**								
Apolipoprotein A-I (g/l)	Buffer delay	50	25	2.8e-03	-9.9e-03	1.6e-02	6.7e-01	1.9e-01
Apolipoprotein A-I (g/l)	NMR delay	50	25	-2.3e-02	-3.5e-02	-1.1e-02	1.5e-04	1.9e-01
Apolipoprotein B (g/l)	Buffer delay	50	25	1.3e-02	-2.7e-04	2.7e-02	5.5e-02	2.4e-01
Apolipoprotein B (g/l)	NMR delay	50	25	1.9e-02	4.6e-03	3.3e-02	9.2e-03	2.4e-01
**Fatty acids**								
Total fatty acids (mmol/l)	Buffer delay	42	21	-1.0e-01	-3.4e-01	1.4e-01	4.0e-01	3.5e+00

Metabolic traits	delay	N.obs	N.indiv	Beta	LCI	UCI	Pvalue	SD
Total fatty acids (mmol/l)	NMR delay	42	21	-2.4e-02	-2.0e-01	1.5e-01	7.9e-01	3.5e+00
Fatty acid chain length	Buffer delay	42	21	5.2e-02	-2.0e-03	1.1e-01	5.9e-02	2.8e-01
Fatty acid chain length	NMR delay	42	21	1.1e-01	5.3e-03	2.1e-01	3.9e-02	2.8e-01
Degree of unsaturation	Buffer delay	42	21	5.2e-04	-7.9e-03	8.9e-03	9.0e-01	8.6e-02
Degree of unsaturation	NMR delay	42	21	4.3e-04	-2.1e-02	2.2e-02	9.7e-01	8.6e-02
Docosahexaenoic acid (mmol/l)	Buffer delay	42	21	-3.1e-03	-6.9e-03	7.8e-04	1.2e-01	4.9e-02
Docosahexaenoic acid (mmol/l)	NMR delay	42	21	-5.7e-03	-9.1e-03	-2.2e-03	1.3e-03	4.9e-02
Linoleic acid (mmol/l)	Buffer delay	42	21	-2.6e-02	-7.0e-02	1.7e-02	2.4e-01	6.4e-01
Linoleic acid (mmol/l)	NMR delay	42	21	2.7e-03	-4.3e-02	4.8e-02	9.1e-01	6.4e-01
Conjugated linoleic acid (mmol/l)	Buffer delay	42	21	-4.2e-03	-8.2e-03	-2.3e-04	3.8e-02	2.4e-02
Conjugated linoleic acid (mmol/l)	NMR delay	42	21	-1.8e-03	-6.6e-03	3.1e-03	4.7e-01	2.4e-02
n-3 fatty acids (mmol/l)	Buffer delay	42	21	-1.5e-02	-2.9e-02	-1.5e-03	3.0e-02	1.5e-01
n-3 fatty acids (mmol/l)	NMR delay	42	21	-4.1e-03	-1.6e-02	7.5e-03	4.9e-01	1.5e-01
n-6 fatty acids (mmol/l)	Buffer delay	42	21	-6.9e-03	-6.2e-02	4.8e-02	8.1e-01	7.2e-01
n-6 fatty acids (mmol/l)	NMR delay	42	21	-2.0e-02	-8.1e-02	4.1e-02	5.3e-01	7.2e-01
PUFA (mmol/l)	Buffer delay	42	21	-2.2e-02	-8.2e-02	3.8e-02	4.7e-01	8.5e-01
PUFA (mmol/l)	NMR delay	42	21	-2.4e-02	-8.8e-02	4.0e-02	4.7e-01	8.5e-01
MUFA (mmol/l)	Buffer delay	42	21	4.2e-02	-2.7e-02	1.1e-01	2.3e-01	1.4e+00
MUFA (mmol/l)	NMR delay	42	21	9.5e-02	-2.8e-02	2.2e-01	1.3e-01	1.4e+00
Saturated fatty acids (mmol/l)	Buffer delay	42	21	-1.2e-01	-2.7e-01	2.1e-02	9.3e-02	1.3e+00
Saturated fatty acids (mmol/l)	NMR delay	42	21	-9.5e-02	-1.9e-01	2.9e-03	5.7e-02	1.3e+00
**Glycolysis related metabolites**								
Glucose (mmol/l)	Buffer delay	50	25	1.1e-02	-9.7e-03	3.2e-02	2.9e-01	5.2e-01
Glucose (mmol/l)	NMR delay	50	25	-1.0e-02	-3.5e-02	1.5e-02	4.3e-01	5.2e-01
Lactate (mmol/l)	Buffer delay	50	25	-6.4e-03	-2.1e-02	8.4e-03	4.0e-01	3.2e-01
Lactate (mmol/l)	NMR delay	50	25	-1.2e-02	-2.7e-02	4.2e-03	1.5e-01	3.2e-01

Metabolic traits	delay	N.obs	N.indiv	Beta	LCI	UCI	Pvalue	SD
Pyruvate (mmol/l)	Buffer delay			NA	NA	NA	NA	NA
Pyruvate (mmol/l)	NMR delay			NA	NA	NA	NA	NA
Citrate (mmol/l)	Buffer delay	50	25	8.8e-03	1.7e-03	1.6e-02	1.5e-02	3.4e-02
Citrate (mmol/l)	NMR delay	50	25	9.4e-03	1.6e-03	1.7e-02	1.9e-02	3.4e-02
Glycerol (mmol/l)	Buffer delay			NA	NA	NA	NA	NA
Glycerol (mmol/l)	NMR delay			NA	NA	NA	NA	NA
**Amino acids**								
Alanine (mmol/l)	Buffer delay	50	25	-5.5e- 03	-9.5e- 03	-1.5e- 03	7.7e-03	5.2e-02
Alanine (mmol/l)	NMR delay	50	25	-8.0e- 04	-4.7e- 03	3.1e-03	6.9e-01	5.2e-02
Glutamine (mmol/l)	Buffer delay	50	25	-1.4e- 02	-2.6e- 02	-2.7e- 03	1.5e-02	6.0e-02
Glutamine (mmol/l)	NMR delay	50	25	-7.0e- 03	-1.4e- 02	-1.6e- 04	4.5e-02	6.0e-02
Histidine (mmol/l)	Buffer delay	50	25	-1.3e- 02	-1.6e- 02	-8.9e- 03	4.8e-11	9.3e-03
Histidine (mmol/l)	NMR delay	50	25	-4.4e- 03	-7.2e- 03	-1.7e- 03	1.4e-03	9.3e-03
Glycine (mmol/l)	Buffer delay			NA	NA	NA	NA	NA
Glycine (mmol/l)	NMR delay			NA	NA	NA	NA	NA
Branched-chain amino acids								
Isoleucine (mmol/l)	Buffer delay	50	25	-4.2e- 03	-6.1e- 03	-2.3e- 03	1.3e-05	2.5e-02
Isoleucine (mmol/l)	NMR delay	50	25	-3.5e- 03	-5.0e- 03	-1.9e- 03	1.0e-05	2.5e-02
Leucine (mmol/l)	Buffer delay	50	25	-2.8e- 03	-3.6e- 03	-2.0e- 03	1.9e-11	2.2e-02
Leucine (mmol/l)	NMR delay	50	25	-4.6e- 04	-1.1e- 03	2.2e-04	1.8e-01	2.2e-02
Valine (mmol/l)	Buffer delay	50	25	-2.3e- 03	-4.0e- 03	-5.2e- 04	1.1e-02	3.6e-02
Valine (mmol/l)	NMR delay	50	25	4.7e-04	-7.9e- 04	1.7e-03	4.6e-01	3.6e-02
Aromatic amino acids								
Phenylalanine (mmol/l)	Buffer delay	50	25	-2.9e- 03	-4.3e- 03	-1.4e- 03	1.5e-04	6.7e-03
Phenylalanine (mmol/l)	NMR delay	50	25	-1.1e- 03	-2.7e- 03	5.5e-04	2.0e-01	6.7e-03
Tyrosine (mmol/l)	Buffer delay	50	25	2.9e-04	-1.0e- 03	1.6e-03	6.7e-01	1.2e-02
Tyrosine (mmol/l)	NMR delay	50	25	3.4e-04	-1.1e- 03	1.8e-03	6.6e-01	1.2e-02
**Ketone bodies**								

Metabolic traits	delay	N.obs	N.indiv	Beta	LCI	UCI	Pvalue	SD
Acetate (mmol/l)	Buffer delay	50	25	2.7e-03	1.5e-03	3.9e-03	8.5e-06	9.7e-03
Acetate (mmol/l)	NMR delay	50	25	-1.3e-03	-2.4e-03	-2.7e-04	1.3e-02	9.7e-03
Beta-hydroxybutyrate (mmol/l)	Buffer delay	50	25	5.4e-03	3.7e-03	7.1e-03	2.3e-10	1.5e-02
Beta-hydroxybutyrate (mmol/l)	NMR delay	50	25	2.9e-03	1.0e-03	4.9e-03	3.0e-03	1.5e-02
**Fluid balance**								
Creatinine (mmol/l)	Buffer delay	50	25	2.8e-03	2.0e-03	3.6e-03	2.3e-12	8.9e-03
Creatinine (mmol/l)	NMR delay	50	25	-9.2e-04	-2.2e-03	3.5e-04	1.6e-01	8.9e-03
Albumin (signal area)	Buffer delay	50	25	8.9e-04	2.6e-04	1.5e-03	5.5e-03	3.5e-03
Albumin (signal area)	NMR delay	50	25	-4.6e-04	-1.1e-03	2.2e-04	1.9e-01	3.5e-03
**Inflammation**								
Glycoprotein acetyls (mmol/l)	Buffer delay	50	25	6.6e-03	-2.4e-03	1.6e-02	1.5e-01	3.2e-01
Glycoprotein acetyls (mmol/l)	NMR delay	50	25	-2.8e-03	-9.1e-03	3.6e-03	4.0e-01	3.2e-01

Table S10. Literature table: summary of previous studies assessing the effects of pre and post-storage handling conditions on serum or plasma metabolic traits also measured by Nightingale Health® NMR platform.

***Note 1*: *Untargeted* metabolomics (U), aims to measure as many metabolites as possible in a biological sample, without any requirement that the metabolites' identification be known beforehand. Quantification is given as relative concentrations. *Targeted* metabolomics (T), provides absolute concentration of a selected panel of metabolites. *Semi-targeted* metabolomics (S) provides absolute concentration using one calibration model to quantify multiple metabolites, instead of one quantification model per metabolite. Quantification is therefore less accurate than in targeted metabolomics.**

Note 2: Studies using NMR have very similar metabolite coverage as the NMR platform used here. Untargeted studies due to its nature, i.e. the type of data (e.g. full spectra) and the statistical methods used (e.g. partial least squares- discriminate analysis), are more biased towards reporting non-robust metabolites. Therefore, for all untargeted NMR studies cited in Table S10, it is assumed that non-reported metabolites are stable. For example, Bernini et al. 2011, uses an untargeted NMR platform, that like the one used in this paper will detect isoleucine, since the authors did not report a change in this amino-acid, it is assumed that it is stable under the conditions they tested. However, in the table below we did not list all metabolic traits detected by the platform used in our study alongside references, that use NMR untargeted platform, which report stability of a metabolite by omission.

*Abbreviations: **CPMG**=Carr-Purcell-Meiboom-Gill; **^{13}C**= Carbon-13; **EDTA**=Ethylenediaminetetraacetic acid; **ESI**= Electrospray ionization; **FIA**= Flow injection analysis; **GC**= Gas Chromatography; **(^{1}H) NMR**= Proton nuclear magnetic resonance; **HDL**= High density lipoprotein; **HSQC**=Heteronuclear Single Quantum Coherence; **J-res**=J-resolved; **LC**=Liquid Chromatography; **LDL**= Low density lipoprotein; **MS**= Mass Spectrometry; **N**= number of individuals; **NMR**= Nuclear Magnetic Resonance; **NOESY**=Nuclear Overhauser Effect Spectroscopy; **RT**=Room Temperature; **STOCSY**= Statistical Total Correlation Spectroscopy; **SST**= Serum-Separating Tube; **1D**=one dimension.*

Details of the metabolomics platforms: **Biocrates®= Biocrates® Life Sciences AG (Innsbruck, Austria), commercial suppliers of the AbsoluteIQD™ commercial kits; **ICL NPC**= Imperial College London National Phenome Centre, laboratory of Jeremy Nicolson, John Lindon and colleagues; **Nightingale Health®**=commercial laboratory formerly known as Brainshake Inc. The same platform is also used by the Mika Ala-Korpela lab at the Biocentre Oulu platform (Finland).*

‡ studies testing the effects of pre-analytical variation on a different pre-analytical *phase* e.g. studies testing *post*-centrifugation conditions during pre-storage (i.e. long-term freezing) sample handling, whilst we tested *pre*-centrifugation conditions during pre-storage handling.

Metabolic trait	Reference	Sample handling phase: Pre/post-storage (i.e. long-term freezing); Pre/post-centrifugation;	Sample type	Incubation temperature	Incubation duration	Analytical Platform* [Targeted (T), Untargeted (U), Semi-targeted (S)]	*N*	Message
**Cholesterol**								
**Total cholesterol**	Key et al. 1996 [3]	Pre-storage: pre-centrifugation;	Serum, sodium citrate-plasma; Fasting status not specified; Serum reference: 20°C, 2h Plasma reference: 20°C (20 min) and after 4°C, 2h	4°C	2, 6, 24h	Clinical Chemistry / (T)	28	**Stable** (decrease in mean percentage change up to 3%)

	Clark et al. 2003[4]	Pre-storage: pre-centrifugation;	Potassium EDTA-plasma; Non-fasting; Reference: centrifuged immediately	4°C, 21°C	0, 1-4, 7 days	Clinical Chemistry / (T)	12	**Stable** (mean percentage change less than 0.5% per day)
	Boyanton et al. 2002[5]	Pre-storage: pre and ǂpost-centrifugation;	Serum, lithium heparin-plasma; Non-fasting; Reference: 0.5h, 25°C	25°C	0.5, 4, 8, 16, 24, 32, 40, 48, 56h	Clinical Chemistry / (T)	10	Pre-storage/pre-centrifugation: **increase**, more pronounced in plasma than serum. Pre-storage/ǂpost-centrifugation: **stable**.
	Oddoze et al. 2012[6]	Pre-storage: pre-centrifugation;	Serum, lithium heparin and fluoride plasma; Fasting status not specified; Serum reference: 0.5h; Plasma reference: centrifuged immediately	4°C, 25°C	0, 2, 4, 6, 24h	Clinical Chemistry / (T)	10	**Stable**.
	Current report	*Pre-storage: pre-centrifugation; Post-storage: post-centrifugation;*	*Serum, potassium EDTA-plasma; Non-fasting; Reference, pre-storage: 1.5h, 4°C; Reference, post-storage: no sample or NMR analysis delay*	*Pre-storage/ pre-centrifugation: 4°C, 21°C Post-storage/ post-centrifugation: 4°C*	*Pre-storage/ pre-centrifugation: 1.5, 24, 48h Post-storage/ post-centrifugation: 0, 24h*	*(^{1}H) NMR (1D, NOESY, CPMG), Nightingale Health* / (T)*	*Pre: 23 Post:25*	*Pre-storage/pre-centrifugation: **stable** at 4°C; mean increase up to 0.2SD at 21°C Post-storage/post-centrifugation: **stable**.*
**LDL cholesterol**	Key et al. 1996[3]	Pre-storage: pre-centrifugation;	Serum, sodium citrate-plasma; Fasting status not specified; Serum reference: 20°C, 2h Plasma reference: 20°C (20 min) and after 4°C, 2h	4°C	2, 6, 24h	Clinical Chemistry / (T)	28	**Stable** (mean percentage change up to 3.4%)
	Clark et al. 2003 [4]	Pre-storage: pre-centrifugation;	Potassium EDTA-plasma; Non-fasting; Reference: centrifuged immediately	4°C, 21°C	0, 1-4, 7 days	Clinical Chemistry / (T)	12	**Stable** (mean percentage change less than 1% per day)
	Oddoze et al. 2012[6]	Pre-storage: pre-centrifugation;	Serum, lithium heparin and fluoride plasma; Fasting status not specified; Serum reference: 0.5h; Plasma reference: centrifuged immediately	4°C, 25°C	0, 2, 4, 6, 24h	Clinical Chemistry / (T)	10	**Stable**.
	Current report	*Pre-storage: pre-centrifugation; Post-storage: post-centrifugation;*	*Serum, potassium EDTA-plasma; Non-fasting; Reference, pre-storage: 1.5h, 4°C;*	*Pre-storage/ pre-centrifugation: 4°C, 21°C*	*Pre-storage/ pre-centrifugation: 1.5, 24, 48h Post-storage/ post-*	*(^{1}H) NMR (1D, NOESY, CPMG), Nightingale Health* / (T)*	*Pre: 23 Post:25*	*Pre-storage/pre-centrifugation: **stable** at 4°C; mean increase up to 0.3SD at 21°C*

			Reference, post-storage: no sample or NMR analysis delay	*Post-storage/ post-centrifugation: 4ºC*	*centrifugation: 0, 24h*			*Post-storage/post-centrifugation:* ***stable****.*
**HDL cholesterol**	Key et al. 1996[3]	Pre-storage: pre-centrifugation;	Serum, sodium citrate-plasma; Fasting status not specified; Serum reference: 20ºC, 2h Plasma reference: 20ºC (20 min) and after 4ºC, 2h	4ºC	2, 6, 24h	Clinical Chemistry / (T)	28	**Stable** (decrease in mean percentage change up to 4.4%)
	Clark et al. 2003[4]	Pre-storage: pre-centrifugation;	Potassium EDTA-plasma; Non-fasting; Reference: centrifuged immediately	4ºC, 21ºC	0, 1-4, 7 days	Clinical Chemistry / (T)	12	**Stable** (mean percentage change less than 1% per day)
	Oddoze et al. 2012[6]	Pre-storage: pre-centrifugation;	Serum, lithium heparin and fluoride plasma; Fasting status not specified; Serum reference: 0.5h; Plasma reference: centrifuged immediately	4ºC, 25ºC	0, 2, 4, 6, 24h	Clinical Chemistry / (T)	10	**Stable**.
	Current report	*Pre-storage: pre-centrifugation; Post-storage: post-centrifugation;*	*Serum, potassium EDTA-plasma; Non-fasting; Reference, pre-storage: 1.5h, 4ºC; Reference, post-storage: no sample or NMR analysis delay*	*Pre-storage/ pre-centrifugation: 4ºC, 21ºC Post-storage/ post-centrifugation: 4ºC*	*Pre-storage/ pre-centrifugation: 1.5, 24, 48h Post-storage/ post-centrifugation: 0, 24h*	*(^{1}H) NMR (1D, NOESY, CPMG), Nightingale Health* / (T)*	*Pre: 23 Post:25*	*Pre-storage/pre-centrifugation:* ***stable****; Post-storage/post-centrifugation:* ***stable****.*
**Glycerides and phospholipids**								
**Triglycerides**	Key et al. 1996[3]	Pre-storage: pre-centrifugation;	Serum, sodium citrate-plasma; Fasting status not specified; Serum reference: 20ºC, 2h Plasma reference: 20ºC (20 min) and after 4ºC, 2h	4ºC	2, 6, 24h	Clinical Chemistry / (T)	28	**Stable** (increase in mean percentage change up to 5.9% but spearman rank correlation between reference and variant conditions is equal or above 0.95)
	Boyanton et al. 2002[5]	Pre-storage: pre and ǂpost-centrifugation;	Serum, lithium heparin-plasma; Non-fasting; Reference: 0.5, 25ºC	25ºC	0.5, 4, 8, 16, 24, 32, 40, 48, 56h	Clinical Chemistry / (T)	10	Pre-storage: pre and ǂpost-centrifugation: **stable**.
	Clark et al. 2003[4]	Pre-storage: pre-centrifugation;	Potassium EDTA-plasma; Non-fasting;	4ºC, 21ºC	0, 1-4, 7 days	Clinical Chemistry / (T)	12	**Stable** (mean percentage change

			Reference: centrifuged immediately					less than 0.5% per day)
	Oddoze et al. 2012[6]	Pre-storage: pre-centrifugation;	Serum, lithium heparin and fluoride plasma; Fasting status not specified; Serum reference: 0.5h; Plasma reference: centrifuged immediately	4°C, 25°C	0, 2, 4, 6, 24h	Clinical Chemistry / (T)	10	**Stable**.
	Bernini et al. 2011[7]	Pre-storage: pre and ǂpost-centrifugation;	Serum (SST), potassium EDTA-plasma; Fasting status not specified; Reference: centrifuged immediately (for serum after 30 min) and frozen immediately	Pre-centrifugation: 4°C, 25°C Post-centrifugation: 25°C	Pre-centrifugation: 0-4h Post-centrifugation: 0,6,12, 24h	(^{1}H) NMR (1D, NOESY CPMG) / (U)	Pre:6 Post:5	Pre-storage/pre-centrifugation: **stable;** Pre-storage/ǂpost-centrifugation: **decrease**.
	Current report	*Pre-storage: pre-centrifugation; Post-storage: post-centrifugation;*	*Serum, potassium EDTA-plasma; Non-fasting; Reference, pre-storage: 1.5h, 4°C; Reference, post-storage: no sample or NMR analysis delay*	*Pre-storage/ pre-centrifugation: 4°C, 21°C Post-storage/ post-centrifugation: 4°C*	*Pre-storage/ pre-centrifugation: 1.5, 24, 48h Post-storage/ post-centrifugation: 0, 24h*	*(^{1}H) NMR (1D, NOESY, CPMG), Nightingale Health* / (T)*	*Pre: 23 Post:25*	*Pre-storage/pre-centrifugation: **stable**; Post-storage/post-centrifugation: **stable**.*
**Phosphatidylcholine**	ǂAnton et al. 2015[8]	Pre-storage: ǂpost-centrifugation;	Serum; Fasting; Reference:max 5h, on ice	Dry ice, wet ice, RT (22-24°C)	0,12, 24, 36h	FIA-ESI-MS/MS, Biocrates*/ (S)	19 (males)	**Decrease** (at RT i.e. 22-24°C)
	ǂPinto et al 2014[9]	Pre-storage: ǂpost-centrifugation;	Heparin or EDTA-plasma (not clear); Fasting status not specified;	RT	1-21h	(^{1}H) NMR (1D, NOESY, CPMG, STOCSY) / (U)	3 (pregnant)	**Increase**
	Current report	*Pre-storage: pre-centrifugation; Post-storage: post-centrifugation;*	*Serum, potassium EDTA-plasma; Non-fasting; Reference, pre-storage: 1.5h, 4°C; Reference, post-storage: no sample or NMR analysis delay*	*Pre-storage/ pre-centrifugation: 4°C, 21°C Post-storage/ post-centrifugation: 4°C*	*Pre-storage/ pre-centrifugation: 1.5, 24, 48h Post-storage/ post-centrifugation: 0, 24h*	*(^{1}H) NMR (1D, NOESY, CPMG), Nightingale Health* / (T)*	*Pre: 20 Post:21*	*Pre-storage/pre-centrifugation: **stable** at 4°C; mean decrease up to 0.2SD at 21°C. Post-storage/post-centrifugation: mean decrease up to 0.3SD.*
**Sphingomyelins**	ǂAnton et al. 2015[8]	Pre-storage: ǂpost-centrifugation;	Serum; Fasting; Reference: max 5h, on ice	Dry ice, wet ice, RT (22-24°C)	0,12, 24, 36h	FIA-ESI-MS/MS, Biocrates* / (S)	19 (males)	**Stable**
	ǂPinto et al 2014[9]	Pre-storage: ǂpost-centrifugation;	Heparin or EDTA-plasma (not clear); Fasting status not specified;	RT	1-21h	(^{1}H) NMR (1D, NOESY, CPMG, STOCSY) / (U)	3 (pregnant)	**Increase**

	Breier et al. 2014[10]	Pre-storage: pre-centrifugation;	Serum, potassium EDTA-plasma; Fasting; Reference, EDTA-plasma: centrifuged immediately; Reference, serum: 0.5h, 21°C.	Serum and EDTA-plasma: 4°C; EDTA-plasma: 21°C	Serum and EDTA-plasma: 0, 3, 6, 24h EDTA-plasma: 24h	ESI-LC-MS/MS, MS/MS, Biocrates*/ (S)	22	**Stable**
	Current report	*Pre-storage: pre-centrifugation; Post-storage: post-centrifugation;*	*Serum, potassium EDTA-plasma; Non-fasting; Reference, pre-storage: 1.5h, 4°C; Reference, post-storage: no sample or NMR analysis delay*	*Pre-storage/ pre-centrifugation: 4°C, 21°C Post-storage/ post-centrifugation: 4°C*	*Pre-storage/ pre-centrifugation: 1.5, 24, 48h Post-storage/ post-centrifugation: 0, 24h*	*(^{1}H) NMR (1D, NOESY, CPMG), Nightingale Health* / (T)*	*Pre: 20 Post:21*	*Pre-storage/pre-centrifugation: **stable**. Post-storage/post-centrifugation: mean changes up to 0.3SD.*
**Cholines**	Jobard et al. 2016[11]	Pre-storage: pre and ǂpost-centrifugation;	Serum, heparin-plasma; Fasting; Reference (pre-centrifugation): 1h,22°C; Reference (post-centrifugation): 15min	Pre-centrifugation: 4°C, 22°C Post-centrifugation: 22°C	Pre-centrifugation: 1h (4°C), 6h (4°C, 22°C); Post-centrifugation: 15min, 1h	(^{1}H) NMR (1D, CPMG, NOESY, (^{1}H-^{13}C) HSQC, STOCSY, J-res) / (U)	96	Pre-storage/pre-centrifugation: **Increase** at 22°C, 6h; **stable** at 4°C. Pre-storage/ǂpost-centrifugation: **stable**
	ǂPinto et al 2014[9]	Pre-storage: ǂpost-centrifugation;	Heparin or EDTA-plasma (not clear); Fasting status not specified;	RT	1-21h	(^{1}H) NMR (1D, NOESY, CPMG, STOCSY) / (U)	3 (pregnant)	**Increase**
	Bernini et al. 2011[7]	Pre-storage: pre and ǂpost-centrifugation;	Serum (SST), potassium EDTA-plasma; Fasting status not specified; Reference: centrifuged immediately (for serum after 30 min) and frozen immediately	Pre-centrifugation: 4°C, 25°C Post-centrifugation: 25°C	Pre-centrifugation: 0-4h Post-centrifugation: 0,6,12, 24h	(^{1}H) NMR (1D, NOESY CPMG) / (U)	Pre: 6 Post: 5	Pre-storage/pre-centrifugation: **stable;** Pre-storage/ǂpost-centrifugation: **decrease**.
	Current report	*Pre-storage: pre-centrifugation; Post-storage: post-centrifugation;*	*Serum, potassium EDTA-plasma; Non-fasting; Reference, pre-storage: 1.5h, 4°C; Reference, post-storage: no sample or NMR analysis delay*	*Pre-storage/ pre-centrifugation: 4°C, 21°C Post-storage/ post-centrifugation: 4°C*	*Pre-storage/ pre-centrifugation: 1.5, 24, 48h Post-storage/ post-centrifugation: 0, 24h*	*(^{1}H) NMR (1D, NOESY, CPMG), Nightingale Health* / (T)*	*Pre: 20 Post:21*	*Pre-storage/pre-centrifugation: **stable**. Post-storage/post-centrifugation: **stable**.*

Apolipoproteins

**Apolipoprotein A-I**	Clark et al. 2003[4]	Pre-storage: pre-centrifugation;	Potassium EDTA-plasma; Non-fasting; Reference: centrifuged immediately	4°C, 21°C	0,1-4, 7 days	Clinical Chemistry / (T)	12	**Stable** (mean percentage change less than 0.5% per day)
	Oddoze et al. 2012[6]	Pre-storage: pre-centrifugation;	Serum (SST), lithium heparin and fluoride plasma; Fasting status not specified; Serum reference: 0.5h; Plasma reference: centrifuged immediately	4°C, 25°C	0, 2, 4, 6, 24h	Clinical Chemistry / (T)	10	**Stable**.
	Current report	*Pre-storage: pre-centrifugation; Post-storage: post-centrifugation;*	*Serum, potassium EDTA-plasma; Non-fasting; Reference, pre-storage: 1.5h, 4°C; Reference, post-storage: no sample or NMR analysis delay*	*Pre-storage/ pre-centrifugation: 4°C, 21°C Post-storage/ post-centrifugation: 4°C*	*Pre-storage/ pre-centrifugation: 1.5, 24, 48h Post-storage/ post-centrifugation: 0, 24h*	*(^{1}H) NMR (1D, NOESY, CPMG), Nightingale Health* / (T)*	*Pre: 23 Post:25*	*Pre-storage/pre-centrifugation: **stable**. Post-storage/post-centrifugation: mean **decrease** up to 0.1SD.*
**Apolipoprotein B**	Clark et al. 2003[4]	Pre-storage: pre-centrifugation;	Potassium EDTA-plasma; Non-fasting; Reference: centrifuged immediately	4°C, 21°C	0,1-4, 7 days	Clinical Chemistry	12	**Stable** (mean percentage change less than 0.5% per day)
	Oddoze et al. 2012[6]	Pre-storage: pre-centrifugation;	Serum, lithium heparin and fluoride plasma; Fasting status not specified; Serum reference: 0.5h; Plasma reference: centrifuged immediately	4°C, 25°C	0, 2, 4, 6, 24h	Clinical Chemistry / (T)	10	**Stable**.
	Current report	*Pre-storage: pre-centrifugation; Post-storage: post-centrifugation;*	*Serum, potassium EDTA-plasma; Non-fasting; Reference, pre-storage: 1.5h, 4°C; Reference, post-storage: no sample or NMR analysis delay*	*Pre-storage/ pre-centrifugation: 4°C, 21°C Post-storage/ post-centrifugation: 4°C*	*Pre-storage/ pre-centrifugation: 1.5, 24, 48h Post-storage/ post-centrifugation: 0, 24h*	*(^{1}H) NMR (1D, NOESY, CPMG), Nightingale Health* / (T)*	*Pre: 23 Post:25*	*Pre-storage/pre-centrifugation: **stable** at 4°C; mean increase up to 0.1SD at 21°C. Post-storage/post-centrifugation: **stable**.*

**Fatty Acids**								
**Total fatty acids**	Jobard et al. 2016[11]	Pre-storage: pre and ǂpost-centrifugation;	Serum, heparin-plasma; Fasting; Reference (pre-centrifugation): 1h,22°C; Reference (post-centrifugation): 15min	Pre-centrifugation: 4°C, 22°C Post-centrifugation: 22°C	Pre-centrifugation: 1h (4°C), 6h (4°C, 22°C); Post-centrifugation: 15min, 1h	(^{1}H) NMR (1D, CPMG, NOESY, (^{1}H-^{13}C) HSQC, STOCSY, J-res) / (U)	96	Pre-storage/pre-centrifugation: **Increase** at 22°C, 6h; **stable** at 4°C, 1 and 6h. Pre-storage/ǂpost-centrifugation: **stable**
	Bernini et al. 2011[7]	Pre-storage: pre and ǂpost-centrifugation;	Serum (SST), potassium EDTA-plasma; Fasting status not specified; Reference: centrifuged immediately (for serum after 30 min) and frozen immediately	Pre-centrifugation: 4°C, 25°C Post-centrifugation: 25°C	Pre-centrifugation: 0-4h Post-centrifugation: 0,6,12, 24h	(^{1}H) NMR (1D, NOESY CPMG) / (U)	Pre:6 Post:5	Pre-storage/pre-centrifugation: **stable;** Pre-storage/ǂpost-centrifugation: **decrease**.
	Current report	*Pre-storage: pre-centrifugation; Post-storage: post-centrifugation;*	*Serum, potassium EDTA-plasma; Non-fasting; Reference, pre-storage: 1.5h, 4°C; Reference, post-storage: no sample or NMR analysis delay*	*Pre-storage/ pre-centrifugation: 4°C, 21°C Post-storage/ post-centrifugation: 4°C*	*Pre-storage/ pre-centrifugation: 1.5, 24, 48h Post-storage/ post-centrifugation: 0, 24h*	*(^{1}H) NMR (1D, NOESY, CPMG), Nightingale Health* / (T)*	*Pre: 20 Post:21*	*Pre-storage/pre-centrifugation: **stable**. Post-storage/post-centrifugation: **stable**.*
**Glycolysis related metabolites**								
**Glucose**	Boyanton et al. 2002[5]	Pre-storage: pre and ǂpost-centrifugation;	Serum, lithium heparin-plasma; Non-fasting; Reference: 0.5, 25°C	25°C	0.5, 4, 8, 16, 24, 32, 40, 48, 56h	Clinical Chemistry / (T)	10	Pre-storage/pre-centrifugation: **decrease** rapidly in 24h, afterwards more slowly. More pronounced in plasma than serum. Pre-storage/ǂpost centrifugation: **stable**.
	Oddoze et al. 2012[6]	Pre-storage: pre-centrifugation;	Serum, lithium heparin and fluoride plasma; Fasting status not specified; Serum reference: 0.5h; Plasma reference: centrifuged immediately	4°C, 25°C	0, 2, 4, 6, 24h	Clinical Chemistry / (T)	10	**Decrease**, greater at 25°C (-10%) than 4°C (-4%). Stable, at 4°C and 25°C, if in sodium fluorite tubes.
	Bernini et al. 2011[7]	Pre-storage: pre and ǂpost-centrifugation;	Serum (SST), potassium EDTA-plasma; Fasting status not specified; Reference: centrifuged immediately (for serum	Pre-centrifugation: 4°C, 25°C Post-centrifugation: 25°C	Pre-centrifugation: 0-4h Post-centrifugation: 0,6,12, 24h	(^{1}H) NMR (1D, NOESY CPMG) / (U)	Pre:6 Post:5	Pre-storage/pre-centrifugation: **decrease** (greater in serum); Pre-storage/ǂpost-centrifugation: **stable**.

			after 30 min) and frozen immediately					
	Jobard et al. 2016[11]	Pre-storage: pre and ǂpost-centrifugation;	Serum, heparin-plasma; Fasting; Reference (pre-centrifugation): 1h,22°C; Reference (post-centrifugation): 15min	Pre-centrifugation: 4°C, 22°C Post-centrifugation: 22°C	Pre-centrifugation: 1h (4°C), 6h (4°C, 22°C); Post-centrifugation: 15min, 1h	(^1H) NMR (1D, CPMG, NOESY, (^1H-^{13}C) HSQC, STOCSY, J-res) / (U)	96	Pre-storage/pre-centrifugation: **decrease** at 22°C, 6h; **stable** at 4°C, 1 and 6h. Pre-storage/ǂpost-centrifugation: **stable.**
	Kamlage et al. 2014 [12]	Pre-storage: pre and ǂpost-centrifugation;	Potassium EDTA -plasma; Fasting status not specified; Pooled plasma	Pre-centrifugation: wet ice, RT (19-22°C) Post-centrifugation: 4°C, 12°C, RT (19-22°C)	Pre-centrifugation: 2, 6h Post-centrifugation: 0, 0.5, 2, 5, 16h	GC-MS, LC-MS/MS, MxP® Broad profiling, MxP®Lipids, Catecholamines, Eicosanoids / (U and T)	20	Pre-storage/pre-centrifugation: **decrease** Pre-storage/ǂpost-centrifugation: **stable.**
	Fliniaux et al. 2011[13]	Pre-storage: pre-centrifugation;	Serum (SST); Fasting status not specified; Reference: 4h, RT	4°C, RT	4, 24h	(^1H) NMR (1D and CPMG, (^1H-^{13}C) HSQC, J-res) / (U)	7	**Decrease** at RT. **Stable** at 4°C.
	Bervoets et al 2015[14]	Pre-storage: pre-centrifugation;	LiHe plasma; Fasting; Reference: 0.5, ice	4°C	0.5, 3, 8h	(^1H) NMR (1D, CPMG) / (U)	20	**Decrease**
	Current report	*Pre-storage: pre-centrifugation; Post-storage: post-centrifugation;*	*Serum, potassium EDTA-plasma; Non-fasting; Reference, pre-storage: 1.5h, 4°C; Reference, post-storage: no sample or NMR analysis delay*	*Pre-storage/ pre-centrifugation: 4°C, 21°C Post-storage/ post-centrifugation: 4°C*	*Pre-storage/ pre-centrifugation: 1.5, 24, 48h Post-storage/ post-centrifugation: 0, 24h*	*(^1H) NMR (1D, NOESY, CPMG), Nightingale Health* / (T)*	*Pre: 23 Post:25*	*Pre-storage/pre-centrifugation: mean **decrease** up to 1.4SD (more pronounced at 21°C). Post-storage/post-centrifugation: **stable**.*
**Lactate**	Boyanton et al. 2002 [5]	Pre-storage: pre and ǂpost-centrifugation;	Serum, lithium heparin-plasma; Non-fasting; Reference: 0.5, 25°C	25°C	0.5, 4, 8, 16, 24, 32, 40, 48, 56h	Clinical Chemistry / (T)	10	Pre-storage/pre-centrifugation: **increase**. Pre-storage/ǂpost centrifugation: **stable**.
	Oddoze et al. 2012 [6]	Pre-storage: pre-centrifugation;	Serum, lithium heparin and fluoride plasma; Fasting status not specified; Serum reference: 0.5h; Plasma reference: centrifuged immediately	4°C, 25°C	0, 2, 4, 6, 24h	Clinical Chemistry / (T)	10	**Increase.** Stable, at 4°C and 25°C, if in sodium fluorite tubes.
	Bernini et al. 2011 [7]	Pre-storage: pre and ǂpost centrifugation;	Serum (SST), potassium EDTA-plasma; Fasting status not specified; Reference: centrifuged	Pre-centrifugation: 4°C, 25°C	Pre-centrifugation: 0-4h Post-centrifugation: 0,6,12, 24h	(^1H) NMR (1D, NOESY CPMG) / (U)	Pre:6 Post:5	Pre-storage/pre-centrifugation: **increase**;

			immediately (for serum after 30 min) and frozen immediately	Post-centrifugation: 25ºC				Pre-storage/ǂpost-centrifugation: **stable**.
	Jobard et al. 2016 [11]	Pre-storage: pre and ǂpost-centrifugation;	Serum, heparin-plasma; Fasting; Reference (pre-centrifugation): 1h,22ºC; Reference (post-centrifugation): 15min	Pre-centrifugation: 4ºC, 22ºC Post-centrifugation: 22ºC	Pre-centrifugation: 1h (4ºC), 6h (4ºC, 22ºC); Post-centrifugation: 15min, 1h	(^{1}H) NMR (1D, CPMG, NOESY, (^{1}H-^{13}C) HSQC, STOCSY, J-res) / (U)	96	Pre-storage/pre-centrifugation: **decrease** at 22ºC, 6h; **stable** at 4ºC, 1 and 6h. Pre-storage/ǂpost-centrifugation: **stable**
	Kamlage et al. 2014 [12]	Pre-storage: pre and ǂpost-centrifugation;	Potassium EDTA -plasma; Fasting status not specified; Pooled plasma	Pre-centrifugation: wet ice, RT (19-22ºC) Post-centrifugation: 4ºC, 12ºC, RT (19-22ºC)	Pre-centrifugation: 2, 6h Post-centrifugation: 0, 0.5, 2, 5, 16h	GC-MS, LC-MS/MS, MxP® Broad profiling, MxP®Lipids, Catecholamines, Eicosanoids / (U and T)	20	Pre-storage/pre-centrifugation: **increase** Pre-storage/ǂpost-centrifugation: **stable.**
	Fliniaux et al. 2011 [13]	Pre-storage: pre-centrifugation;	Serum (SST); Fasting status not specified; Reference: 4h, RT	4ºC, RT	4, 24h	(^{1}H) NMR (1D and CPMG, (^{1}H-^{13}C) HSQC, J-res) / (U)	7	**Increase** at RT. **Stable** at 4ºC.
	Trezzi et al 2016[15]	Pre-storage: pre-centrifugation;	Potassium EDTA-plasma; Fasting status not specified;	4ºC, RT (18-23ºC)	U: 10, 30 and 60 min T: 0.5, 3, 23h	GC-MS, clinical chemistry/ (U and T)	U: 3 T: 10	**Increase**
	Bervoets et al 2015[14]	Pre-storage: pre-centrifugation;	LiHe plasma; Fasting; Reference: 0.5, ice	4ºC	0.5, 3, 8h	(^{1}H) NMR (1D, CPMG) / (U)	20	**Increase**
	Current report	*Pre-storage: pre-centrifugation; Post-storage: post-centrifugation;*	*Serum, potassium EDTA-plasma; Non-fasting; Reference, pre-storage: 1.5h, 4ºC; Reference, post-storage: no sample or NMR analysis delay*	*Pre-storage/ pre-centrifugation: 4ºC, 21ºC Post-storage/ post-centrifugation: 4ºC*	*Pre-storage/ pre-centrifugation: 1.5, 24, 48h Post-storage/ post-centrifugation: 0, 24h*	*(^{1}H) NMR (1D, NOESY, CPMG), Nightingale Health* / (T)*	*Pre: 23 Post:25*	*Pre-storage/pre-centrifugation: mean **increase** up to 1.4SD (more pronounced at 21ºC). Post-storage/post-centrifugation: **stable**.*
**Pyruvate**	Bernini et al. 2011 [7]	Pre-storage: pre and ǂpost-centrifugation;	Serum (SST), potassium EDTA-plasma; Fasting status not specified; Reference: centrifuged immediately (for serum after 30 min) and frozen immediately	Pre-centrifugation: 4ºC, 25ºC Post-centrifugation: 25ºC	Pre-centrifugation: 0-4h Post-centrifugation: 0,6,12, 24h	(^{1}H) NMR (1D, NOESY CPMG) / (U)	Pre:6 Post:5	Pre-storage/pre-centrifugation: **plasma**: slightly **decrease/stable** at 4ºC; **increase** at 25ºC; **serum**: **decreases** at 4ºC, **stable** at 25ºC Pre-storage/ǂpost-centrifugation: **stable**.
	Nishiumi et al 2017[16]	Pre-storage: pre-centrifugation;	Sodium EDTA-plasma; Fasting status not specified;	Cold temperature, room temperature	Cold temperature: 1, 4, 8h Room temperature: 0, 15, 30 min	GC-MS, LC-MS/MS/(T)	1	Room temperature: **increase**; Cold temperature: **decrease**.

	Kamlage et al. 2014 [12]	Pre-storage: pre and ǂpost-centrifugation;	Potassium EDTA -plasma; Fasting status not specified; Pooled plasma	Pre-centrifugation: wet ice, RT (19-22°C) Post-centrifugation: 4°C, 12°C, RT (19-22°C)	Pre-centrifugation: 2, 6h Post-centrifugation: 0, 0.5, 2, 5, 16h	GC-MS, LC-MS/MS, MxP® Broad profiling, MxP®Lipids, Catecholamines, Eicosanoids / (U and T)	20	Pre-storage/pre-centrifugation: **decrease.** Pre-storage/ǂpost-centrifugation: **decrease.**
	Bervoets et al 2015[14]	Pre-storage: pre-centrifugation;	LiHe plasma; Fasting; Reference: 0.5, ice	4°C	0.5, 3, 8h	(^{1}H) NMR (1D, CPMG) / (U)	20	**Decrease**
	Current report	*Pre-storage: pre-centrifugation; Post-storage: post-centrifugation;*	*Serum; Non-fasting; Reference, pre-storage: 1.5h, 4°C; Reference, post-storage: no sample or NMR analysis delay*	*Pre-storage/ pre-centrifugation: 4°C, 21°C Post-storage/ post-centrifugation: 4°C*	*Pre-storage/ pre-centrifugation: 1.5, 24, 48h Post-storage/ post-centrifugation: 0, 24h*	*(^{1}H) NMR (1D, NOESY, CPMG), Nightingale Health* / (T)*	*Pre:20 Post:37*	*Pre-storage/pre-centrifugation: slight **decrease/stable** at 4°C; mean increase of 1.2SD at 21°C. Post-storage/post-centrifugation: **stable**.*
**Citrate**	Jobard et al. 2016 [11]	Pre-storage: pre and ǂpost-centrifugation;	Serum, heparin-plasma; Fasting; Reference (pre-centrifugation): 1h,22°C; Reference (post-centrifugation): 15min	Pre-centrifugation: 4°C, 22°C Post-centrifugation: 22°C	Pre-centrifugation: 1h (4°C), 6h (4°C, 22°C); Post-centrifugation: 15min, 1h	(^{1}H) NMR (1D, CPMG, NOESY, (^{1}H-^{13}C) HSQC, STOCSY, J-res) / (U)	96	Pre-storage/pre-centrifugation: **Increase** at 22°C, 6h (but Variable Importance in Projection <1); **stable** at 4°C, 1 and 6h. Pre-storage/ǂpost-centrifugation: **stable**
	Bernini et al. 2011[7]	Pre-storage: pre and ǂpost-centrifugation;	Serum (SST), potassium EDTA-plasma; Fasting status not specified; Reference: centrifuged immediately (for serum after 30 min) and frozen immediately	Pre-centrifugation: 4°C, 25°C Post-centrifugation: 25°C	Pre-centrifugation: 0-4h Post-centrifugation: 0,6,12, 24h	(^{1}H) NMR (1D, NOESY CPMG) / (U)	Pre:6 Post:5	Pre-storage/pre-centrifugation: **stable;** Pre-storage/ǂpost-centrifugation: **increase**.
	Current report	*Pre-storage: pre-centrifugation; Post-storage: post-centrifugation;*	*Serum, potassium EDTA-plasma; Non-fasting; Reference, pre-storage: 1.5h, 4°C; Reference, post-storage: no sample or NMR analysis delay*	*Pre-storage/ pre-centrifugation: 4°C, 21°C Post-storage/ post-centrifugation: 4°C*	*Pre-storage/ pre-centrifugation: 1.5, 24, 48h Post-storage/ post-centrifugation: 0, 24h*	*(^{1}H) NMR (1D, NOESY, CPMG), Nightingale Health* / (T)*	*Pre:23 Post:25*	*Pre-storage/pre-centrifugation: changes up to 0.2SD. Post-storage/post-centrifugation: serum **stable**; EDTA-plasma, **increase** up to 0.3SD.*

**Glycerol**	Jobard et al. 2016 [11]	Pre-storage: pre and ǂpost-centrifugation;	Serum, heparin-plasma; Fasting; Reference (pre-centrifugation): 1h,22°C; Reference (post-centrifugation): 15min	Pre-centrifugation: 4°C, 22°C Post-centrifugation: 22°C	Pre-centrifugation: 1h (4°C), 6h (4°C, 22°C); Post-centrifugation: 15min, 1h	(^{1}H) NMR (1D, CPMG, NOESY, (^{1}H-^{13}C) HSQC, STOCSY, J-res) / (U)	96	Pre-storage/pre-centrifugation: **Increase** at 22°C, 6h (but Variable Importance in Projection <1); **stable** at 4°C, 1 and 6h. Pre-storage/ǂpost-centrifugation: **stable**
	Kamlage et al. 2014 [12]	Pre-storage: pre and ǂpost-centrifugation;	Potassium EDTA -plasma; Fasting status not specified; Pooled plasma	Pre-centrifugation: wet ice, RT (19-22°C) Post-centrifugation: 4°C, 12°C, RT (19-22°C)	Pre-centrifugation: 2, 6h Post-centrifugation: 0, 0.5, 2, 5, 16h	GC-MS, LC-MS/MS, MxP® Broad profiling, MxP®Lipids, Catecholamines, Eicosanoids / (U and T)	20	Pre-storage/pre-centrifugation: **decrease.** Pre-storage/ǂpost-centrifugation: **increase.**
	Current report	*Pre-storage: pre-centrifugation; Post-storage: post-centrifugation;*	*Serum, potassium EDTA-plasma; Non-fasting; Reference, pre-storage: 1.5h, 4°C; Reference, post-storage: no sample or NMR analysis delay*	*Pre-storage/ pre-centrifugation: 4°C, 21°C Post-storage/ post-centrifugation: 4°C*	*Pre-storage/ pre-centrifugation: 1.5, 24, 48h Post-storage/ post-centrifugation: 0, 24h*	*(^{1}H) NMR (1D, NOESY, CPMG), Nightingale Health* / (T)*	*Pre:23 Post:37*	*Pre-storage/pre-centrifugation: **stable** at 21°C; mean increase of 0.5SD at 4°C. Post-storage/post-centrifugation: **stable**.*
**Amino Acids**								
**Alanine**	Jobard et al. 2016 [11]	Pre-storage: pre and ǂpost-centrifugation;	Serum, heparin-plasma; Fasting; Reference (pre-centrifugation): 1h,22°C; Reference (post-centrifugation): 15min	Pre-centrifugation: 4°C, 22°C Post-centrifugation: 22°C	Pre-centrifugation: 1h (4°C), 6h (4°C, 22°C); Post-centrifugation: 15min, 1h	(^{1}H) NMR (1D, CPMG, NOESY, (^{1}H-^{13}C) HSQC, STOCSY, J-res) / (U)	96	Pre-storage/pre-centrifugation: **increase** at 22°C, 6h (but for serum Variable Importance in Projection <1); **stable** at 4°C, 1 and 6h. Pre-storage/ǂpost-centrifugation: **stable**
	Breier et al. 2014[10]	Pre-storage: pre-centrifugation;	Serum, potassium EDTA-plasma; Fasting; Reference, EDTA-plasma: centrifuged immediately; Reference, serum: 0.5h, 21°C.	Serum and EDTA-plasma: 4°C; EDTA-plasma: 21°C	Serum and EDTA-plasma: 0, 3, 6, 24h EDTA-plasma: 24h	ESI-LC-MS/MS, MS/MS, Biocrates*/ (S)	22	EDTA-plasma: **increase**;
	Current report	*Pre-storage: pre-centrifugation; Post-storage: post-centrifugation;*	*Serum, potassium EDTA-plasma; Non-fasting; Reference, pre-storage: 1.5h, 4°C; Reference, post-storage: no sample or NMR analysis delay*	*Pre-storage/ pre-centrifugation: 4°C, 21°C Post-storage/ post-centrifugation: 4°C*	*Pre-storage/ pre-centrifugation: 1.5, 24, 48h Post-storage/ post-centrifugation: 0, 24h*	*(^{1}H) NMR (1D, NOESY, CPMG), Nightingale Health* / (T)*	*Pre:23 Post:25*	*Pre-storage/pre-centrifugation: mean **increase** up to 1.2SD (more pronounced at 21°C). Post-storage/post-centrifugation: **stable**.*

Glutamine	Jobard et al. 2016 [11]	Pre-storage: pre and ‡post-centrifugation;	Serum, heparin-plasma; Fasting; Reference (pre-centrifugation): 1h,22ºC; Reference (post-centrifugation): 15min	Pre-centrifugation: 4ºC, 22ºC Post-centrifugation: 22ºC	Pre-centrifugation: 1h (4ºC), 6h (4ºC, 22ºC); Post-centrifugation: 15min, 1h	(^{1}H) NMR (1D, CPMG, NOESY, (^{1}H-^{13}C) HSQC, STOCSY, J-res) / (U)	96	Pre-storage/pre-centrifugation: **decrease** at 22ºC, 6h (but Variable Importance in Projection <1); **stable** at 4ºC, 1 and 6h. Pre-storage/‡post-centrifugation: **stable**
	‡Anton et al. 2015[8]	Pre-storage: ‡post-centrifugation;	Serum; Fasting; Reference: max 5h, on ice	Dry ice, wet ice, RT (22-24ºC)	0,12, 24, 36h	FIA-ESI-MS/MS, Biocrates*/ (S)	19 (males)	**Decrease** (22-24ºC and wet ice)
	Current report	*Pre-storage: pre-centrifugation; Post-storage: post-centrifugation;*	*Serum, potassium EDTA-plasma; Non-fasting; Reference, pre-storage: 1.5h, 4ºC; Reference, post-storage: no sample or NMR analysis delay*	*Pre-storage/ pre-centrifugation: 4ºC, 21ºC Post-storage/ post-centrifugation: 4ºC*	*Pre-storage/ pre-centrifugation: 1.5, 24, 48h Post-storage/ post-centrifugation: 0, 24h*	*(^{1}H) NMR (1D, NOESY, CPMG), Nightingale Health* / (T)*	*Pre: 23 Post:25*	*Pre-storage/pre-centrifugation: mean de**crease** up to 0.9SD (more pronounced at 21ºC). Post-storage/post-centrifugation: changes up to 0.2SD.*
Histidine	Jobard et al. 2016 [11]	Pre-storage: pre and ‡post-centrifugation;	Serum, heparin-plasma; Fasting; Reference (pre-centrifugation): 1h,22ºC; Reference (post-centrifugation): 15min	Pre-centrifugation: 4ºC, 22ºC Post-centrifugation: 22ºC	Pre-centrifugation: 1h (4ºC), 6h (4ºC, 22ºC); Post-centrifugation: 15min, 1h	(^{1}H) NMR (1D, CPMG, NOESY, (^{1}H-^{13}C) HSQC, STOCSY, J-res) / (U)	96	Pre-storage/pre-centrifugation: **Increase** at 22ºC, 6h (but Variable Importance in Projection <1); **stable** at 4ºC, 1 and 6h. Pre-storage/‡post-centrifugation: **stable**
	Bernini et al. 2011[7]	Pre-storage: pre and ‡post-centrifugation;	Serum (SST), potassium EDTA-plasma; Fasting status not specified; Reference: centrifuged immediately (for serum after 30 min) and frozen immediately	Pre-centrifugation: 4ºC, 25ºC Post-centrifugation: 25ºC	Pre-centrifugation: 0-4h Post-centrifugation: 0,6,12, 24h	(^{1}H) NMR (1D, NOESY CPMG) / (U)	Pre:6 Post:5	Pre-storage/pre-centrifugation: **stable;** Pre-storage/‡post-centrifugation: **decrease**.
	Breier et al. 2014[10]	Pre-storage: pre-centrifugation;	Serum, potassium EDTA-plasma; Fasting; Reference, EDTA-plasma: centrifuged immediately; Reference, serum: 0.5h, 21ºC.	Serum and EDTA-plasma: 4ºC; EDTA-plasma: 21ºC	Serum and EDTA-plasma: 0, 3, 6, 24h EDTA-plasma: 24h	ESI-LC-MS/MS, MS/MS, Biocrates*/ (S)	22	EDTA-plasma: **increase** only at 21ºC.
	Current report	*Pre-storage: pre-centrifugation; Post-storage: post-centrifugation;*	*Serum, potassium EDTA-plasma; Non-fasting; Reference, pre-storage: 1.5h, 4ºC; Reference, post-storage: no sample or*	*Pre-storage/ pre-centrifugation: 4ºC, 21ºC Post-storage/ post-centrifugation: 4ºC*	*Pre-storage/ pre-centrifugation: 1.5, 24, 48h Post-storage/ post-centrifugation: 0, 24h*	*(^{1}H) NMR (1D, NOESY, CPMG), Nightingale Health* / (T)*	*Pre:23 Post:25*	*Pre-storage/pre-centrifugation: serum, mean **increase** up to 1.2SD; EDTA-plasma, mean **decrease** up to 0.8SD. Post-storage/post-centrifugation:*

			NMR analysis delay					***decrease** up to 1.4SD.*
**Glycine**	Jobard et al. 2016 [11]	Pre-storage: pre and ‡post-centrifugation;	Serum, heparin-plasma; Fasting; Reference (pre-centrifugation): 1h,22°C; Reference (post-centrifugation): 15min	Pre-centrifugation: 4°C, 22°C Post-centrifugation: 22°C	Pre-centrifugation: 1h (4°C), 6h (4°C, 22°C); Post-centrifugation: 15min, 1h	(^{1}H) NMR (1D, CPMG, NOESY, (^{1}H-^{13}C) HSQC, STOCSY, J-res) / (U)	96	Pre-storage/pre-centrifugation: **Increase** at 22°C, 6h (but Variable Importance in Projection <1); **stable** at 4°C, 1 and 6h. Pre-storage/‡post-centrifugation: **stable**
	‡Anton et al. 2015[8]	Pre-storage: ‡post-centrifugation;	Serum; Fasting; Reference:max 5h, on ice	Dry ice, wet ice, RT (22-24°C)	0,12, 24, 36h	FIA-ESI-MS/MS, Biocrates*/ (S)	19 (males)	**Increase** (at RT i.e. 22-24°C)
	Breier et al. 2014[10]	Pre-storage: pre-centrifugation;	Serum, potassium EDTA-plasma; Fasting; Reference, EDTA-plasma: centrifuged immediately; Reference, serum: 0.5h, 21°C.	Serum and EDTA-plasma: 4°C; EDTA-plasma: 21°C	Serum and EDTA-plasma: 0, 3, 6, 24h EDTA-plasma: 24h	ESI-LC-MS/MS, MS/MS, Biocrates*/ (S)	22	Serum: **increase.**
	Current report	*Pre-storage: pre-centrifugation; Post-storage: post-centrifugation;*	*Serum; Non-fasting; Reference, pre-storage: 1.5h, 4°C; Reference, post-storage: no sample or NMR analysis delay*	*Pre-storage/ pre-centrifugation: 4°C, 21°C Post-storage/ post-centrifugation: 4°C*	*Pre-storage/ pre-centrifugation: 1.5, 24, 48h Post-storage/ post-centrifugation: 0, 24h*	*(^{1}H) NMR (1D, NOESY, CPMG), Nightingale Health* / (T)*	*Pre:23 Post:37*	*Pre-storage/pre-centrifugation: mean **increase** up to 1SD. Post-storage/post-centrifugation: mean **increase** up to 0.3SD.*
Branched-chain Amino Acids								
**Isoleucine**	Jobard et al. 2016 [11]	Pre-storage: pre and ‡post-centrifugation;	Serum, heparin-plasma; Fasting; Reference (pre-centrifugation): 1h,22°C; Reference (post-centrifugation): 15min	Pre-centrifugation: 4°C, 22°C Post-centrifugation: 22°C	Pre-centrifugation: 1h (4°C), 6h (4°C, 22°C); Post-centrifugation: 15min, 1h	(^{1}H) NMR (1D, CPMG, NOESY, (^{1}H-^{13}C) HSQC, STOCSY, J-res) / (U)	96	Pre-storage/pre-centrifugation: **Increase** at 22°C, 6h (but Variable Importance in Projection <1); **stable** at 4°C, 1 and 6h. Pre-storage/‡post-centrifugation: **stable**
	‡Anton et al. 2015[8]	Pre-storage: ‡post-centrifugation;	Serum; Fasting; Reference:max 5h, on ice	Dry ice, wet ice, RT (22-24°C)	0,12, 24, 36h	FIA-ESI-MS/MS, Biocrates*/ (S)	19 (males)	**Increase** (at RT i.e. 22-24°C)
	Breier et al. 2014[10]	Pre-storage: pre-centrifugation;	Serum, potassium EDTA-plasma; Fasting; Reference, EDTA-plasma: centrifuged immediately; Reference, serum: 0.5h, 21°C.	Serum and EDTA-plasma: 4°C; EDTA-plasma: 21°C	Serum and EDTA-plasma: 0, 3, 6, 24h EDTA-plasma: 24h	ESI-LC-MS/MS, MS/MS, Biocrates*/ (S)	22	EDTA-plasma: **increase** only at 21°C.
	Current report	*Pre-storage: pre-centrifugation; Post-storage: post-centrifugation;*	*Serum, potassium EDTA-plasma; Non-fasting;*	*Pre-storage/ pre-centrifugation: 4°C, 21°C*	*Pre-storage/ pre-centrifugation: 1.5, 24, 48h*	*(^{1}H) NMR (1D, NOESY, CPMG), Nightingale Health* / (T)*	*Pre:23 Post:25*	*Pre-storage/pre-centrifugation: EDTA-plasma, stable; serum, mean*

			Reference, pre-storage: 1.5h, 4°C; Reference, post-storage: no sample or NMR analysis delay	*Post-storage/ post-centrifugation: 4°C*	*Post-storage/ post-centrifugation: 0, 24h*			***increase** up to 0.4SD. Post-storage/post-centrifugation: stable.*
**Leucine**	Jobard et al. 2016 [11]	Pre-storage: pre and ‡post-centrifugation;	Serum, heparin-plasma; Fasting; Reference (pre-centrifugation): 1h,22°C; Reference (post-centrifugation): 15min	Pre-centrifugation: 4°C, 22°C Post-centrifugation: 22°C	Pre-centrifugation: 1h (4°C), 6h (4°C, 22°C); Post-centrifugation: 15min, 1h	(^{1}H) NMR (1D, CPMG, NOESY, (^{1}H-^{13}C) HSQC, STOCSY, J-res) / (U)	96	Pre-storage/pre-centrifugation: **Increase** at 22°C, 6h (but Variable Importance in Projection <1); **stable** at 4°C, 1 and 6h. Pre-storage/‡post-centrifugation: **stable.**
	Breier et al. 2014[10]	Pre-storage: pre-centrifugation;	Serum, potassium EDTA-plasma; Fasting; Reference, EDTA-plasma: centrifuged immediately; Reference, serum: 0.5h, 21°C.	Serum and EDTA-plasma: 4°C; EDTA-plasma: 21°C	Serum and EDTA-plasma: 0, 3, 6, 24h EDTA-plasma: 24h	ESI-LC-MS/MS, MS/MS, Biocrates*/ (S)	22	**Increase**.
	Current report	*Pre-storage: pre-centrifugation; Post-storage: post-centrifugation;*	*Serum, potassium EDTA-plasma; Non-fasting; Reference, pre-storage: 1.5h, 4°C; Reference, post-storage: no sample or NMR analysis delay*	*Pre-storage/ pre-centrifugation: 4°C, 21°C Post-storage/ post-centrifugation: 4°C*	*Pre-storage/ pre-centrifugation: 1.5, 24, 48h Post-storage/ post-centrifugation: 0, 24h*	*(^{1}H) NMR (1D, NOESY, CPMG), Nightingale Health* / (T)*	*Pre:23 Post:25*	*Pre-storage/pre-centrifugation: mean **increase** up to 0.8SD. Post-storage/post-centrifugation: **stable**.*
**Valine**	Jobard et al. 2016 [11]	Pre-storage: pre and ‡post-centrifugation;	Serum, heparin-plasma; Fasting; Reference (pre-centrifugation): 1h,22°C; Reference (post-centrifugation): 15min	Pre-centrifugation: 4°C, 22°C Post-centrifugation: 22°C	Pre-centrifugation: 1h (4°C), 6h (4°C, 22°C); Post-centrifugation: 15min, 1h	(^{1}H) NMR (1D, CPMG, NOESY, (^{1}H-^{13}C) HSQC, STOCSY, J-res) / (U)	96	Pre-storage/pre-centrifugation: **Increase** at 22°C, 6h (but Variable Importance in Projection <1); **stable** at 4°C, 1 and 6h. Pre-storage/‡post-centrifugation: **stable.**
	Current report	*Pre-storage: pre-centrifugation; Post-storage: post-centrifugation;*	*Serum, potassium EDTA-plasma; Non-fasting; Reference, pre-storage: 1.5h, 4°C; Reference, post-storage: no sample or NMR analysis delay*	*Pre-storage/ pre-centrifugation: 4°C, 21°C Post-storage/ post-centrifugation: 4°C*	*Pre-storage/ pre-centrifugation: 1.5, 24, 48h Post-storage/ post-centrifugation: 0, 24h*	*(^{1}H) NMR (1D, NOESY, CPMG), Nightingale Health* / (T)*	*Pre:23 Post:25*	*Pre-storage/pre-centrifugation: mean **increase** up to 0.8SD. Post-storage/post-centrifugation: **stable**.*
Aromatic Amino Acids								
**Phenylalanine**	Jobard et al. 2016 [11]	Pre-storage: pre and ‡post-centrifugation;	Serum, heparin-plasma; Fasting; Reference (pre-centrifugation): 1h,22°C; Reference	Pre-centrifugation: 4°C, 22°C Post-centrifugation: 22°C	Pre-centrifugation: 1h (4°C), 6h (4°C, 22°C); Post-centrifugation: 15min, 1h	(^{1}H) NMR (1D, CPMG, NOESY, (^{1}H-^{13}C) HSQC, STOCSY, J-res) / (U)	96	Pre-storage/pre-centrifugation: **Increase** at 22°C, 6h (but Variable Importance in Projection <1); **stable** at 4°C, 1 and 6h.

			(post-centrifugation): 15min					Pre-storage/‡post-centrifugation: **stable.**
	‡Anton et al. 2015[8]	Pre-storage: ‡post-centrifugation;	Serum; Fasting; Reference:max 5h, on ice	Dry ice, wet ice, RT (22-24°C)	0,12, 24, 36h	FIA-ESI-MS/MS, Biocrates*/ (S)	19 (males)	**Increase** (at RT i.e. 22-24°C)
	Breier et al. 2014[10]	Pre-storage: pre-centrifugation;	Serum, potassium EDTA-plasma; Fasting; Reference, EDTA-plasma: centrifuged immediately; Reference, serum: 0.5h, 21°C.	Serum and EDTA-plasma: 4°C; EDTA-plasma: 21°C	Serum and EDTA-plasma: 0, 3, 6, 24h EDTA-plasma: 24h	ESI-LC-MS/MS, MS/MS, Biocrates*/ (S)	22	EDTA-plasma: **increase** only at 21°C. Serum: **increase.**
	Current report	*Pre-storage: pre-centrifugation; Post-storage: post-centrifugation;*	*Serum, potassium EDTA-plasma; Non-fasting; Reference, pre-storage: 1.5h, 4°C; Reference, post-storage: no sample or NMR analysis delay*	*Pre-storage/ pre-centrifugation: 4°C, 21°C Post-storage/ post-centrifugation: 4°C*	*Pre-storage/ pre-centrifugation: 1.5, 24, 48h Post-storage/ post-centrifugation: 0, 24h*	*(^{1}H) NMR (1D, NOESY, CPMG), Nightingale Health* / (T)*	*Pre:23 Post:25*	*Pre-storage/pre-centrifugation: mean **increase** up to 1.2SD; Post-storage/post-centrifugation: serum, mean **increase** up to 1.1SD EDTA-plasma, **decrease** up to 0.4SD.*
**Tyrosine**	Jobard et al. 2016 [11]	Pre-storage: pre and ‡post-centrifugation;	Serum, heparin-plasma; Fasting; Reference (pre-centrifugation): 1h,22°C; Reference (post-centrifugation): 15min	Pre-centrifugation: 4°C, 22°C Post-centrifugation: 22°C	Pre-centrifugation: 1h (4°C), 6h (4°C, 22°C); Post-centrifugation: 15min, 1h	(^{1}H) NMR (1D, CPMG, NOESY, (^{1}H-^{13}C) HSQC, STOCSY, J-res) / (U)	96	Pre-storage/pre-centrifugation: **Increase** at 22°C, 6h (but Variable Importance in Projection <1); **stable** at 4°C, 1 and 6h. Pre-storage/‡post-centrifugation: **stable.**
	Breier et al. 2014[10]	Pre-storage: pre-centrifugation;	Serum, potassium EDTA-plasma; Fasting; Reference, EDTA-plasma: centrifuged immediately; Reference, serum: 0.5h, 21°C.	Serum and EDTA-plasma: 4°C; EDTA-plasma: 21°C	Serum and EDTA-plasma: 0, 3, 6, 24h EDTA-plasma: 24h	ESI-LC-MS/MS, MS/MS, Biocrates*/ (S)	22	EDTA-plasma: **increase** only at 21°C. Serum: **increase.**
	Current report	*Pre-storage: pre-centrifugation; Post-storage: post-centrifugation;*	*Serum, potassium EDTA-plasma; Non-fasting; Reference, pre-storage: 1.5h, 4°C; Reference, post-storage: no sample or NMR analysis delay*	*Pre-storage/ pre-centrifugation: 4°C, 21°C Post-storage/ post-centrifugation: 4°C*	*Pre-storage/ pre-centrifugation: 1.5, 24, 48h Post-storage/ post-centrifugation: 0, 24h*	*(^{1}H) NMR (1D, NOESY, CPMG), Nightingale Health* / (T)*	*Pre:23 Post:25*	*Pre-storage/pre-centrifugation: mean **increase** up to 0.5SD; Post-storage/post-centrifugation: **stable**.*
**Ketone bodies**								
**Acetat e**	Jobard et al. 2016 [11]	Pre-storage: pre and ‡post-centrifugation;	Serum, heparin-plasma; Fasting; Reference (pre-centrifugation):	Pre-centrifugation: 4°C, 22°C Post-centrifugation: 22°C	Pre-centrifugation: 1h (4°C), 6h (4°C, 22°C);	(^{1}H) NMR (1D, CPMG, NOESY, (^{1}H-^{13}C) HSQC, STOCSY, J-res) / (U)	96	Pre-storage/pre-centrifugation: **decrease** at 22°C, 6h (but Variable Importance in Projection <1);

			1h,22ºC; Reference (post-centrifugation): 15min		Post-centrifugation: 15min, 1h			**stable** at 4ºC, 1 and 6h. Pre-storage/‡post-centrifugation: **stable.**
	Current report	*Pre-storage: pre-centrifugation; Post-storage: post-centrifugation;*	*Serum, potassium EDTA-plasma; Non-fasting; Reference, pre-storage: 1.5h, 4ºC; Reference, post-storage: no sample or NMR analysis delay*	*Pre-storage/ pre-centrifugation: 4ºC, 21ºC Post-storage/ post-centrifugation: 4ºC*	*Pre-storage/ pre-centrifugation: 1.5, 24, 48h Post-storage/ post-centrifugation: 0, 24h*	*(¹H) NMR (1D, NOESY, CPMG), Nightingale Health* / (T)*	*Pre:23 Post:25*	*Pre-storage/pre-centrifugation: serum, mean **increase** up to 0.9SD; EDTA-plasma, mean **decrease** up to 0.9SD. Post-storage/post-centrifugation: mean **increase** up to 0.5SD.*
**Beta-hydroxybutyrate**	Jobard et al. 2016 [11]	Pre-storage: pre and ‡post-centrifugation;	Serum, heparin-plasma; Fasting; Reference (pre-centrifugation): 1h,22ºC; Reference (post-centrifugation): 15min	Pre-centrifugation: 4ºC, 22ºC Post-centrifugation: 22ºC	Pre-centrifugation: 1h (4ºC), 6h (4ºC, 22ºC); Post-centrifugation: 15min, 1h	(¹H) NMR (1D, CPMG, NOESY, (¹H-¹³C) HSQC, STOCSY, J-res) / (U)	96	Pre-storage/pre-centrifugation: **decrease** at 22ºC, 6h (but Variable Importance in Projection <1); **stable** at 4ºC, 1 and 6h. Pre-storage/‡post-centrifugation: **stable.**
	Current report	*Pre-storage: pre-centrifugation; Post-storage: post-centrifugation;*	*Serum, potassium EDTA-plasma; Non-fasting; Reference, pre-storage: 1.5h, 4ºC; Reference, post-storage: no sample or NMR analysis delay*	*Pre-storage/ pre-centrifugation: 4ºC, 21ºC Post-storage/ post-centrifugation: 4ºC*	*Pre-storage/ pre-centrifugation: 1.5, 24, 48h Post-storage/ post-centrifugation: 0, 24h*	*(¹H) NMR (1D, NOESY, CPMG), Nightingale Health* / (T)*	*Pre:23 Post:25*	*Pre-storage/pre-centrifugation: **stable**. Post-storage/post-centrifugation: mean **increase** up to 0.4SD.*
**Fluid Balance**								
**Creatinine**	Clark et al. 2003 [4]	Pre-storage: pre-centrifugation;	Potassium EDTA-plasma; Non-fasting; Reference: centrifuged immediately	4ºC, 21ºC	0, 1-4, 7 days	Clinical Chemistry / (T)	12	**Stable at 4ºC** (mean percentage change less than 0.5% per day); **Increase** more than 5% per day at **21ºC**.
	Boyanton et al. 2002 [5]	Pre-storage: pre and ‡post-centrifugation;	Serum, lithium heparin-plasma; Non-fasting; Reference: 0.5, 25ºC	25ºC	0.5, 4, 8, 16, 24, 32, 40, 48, 56h	Clinical Chemistry / (T)	10	Pre-storage/pre-centrifugation: **Increase** by 110% in plasma and 60% in serum after 24h (possibly due to interference of pseudo-creatinines in the assay). Pre-storage/‡post-centrifugation: **stable**.
	Oddoze et al. 2012 [6]	Pre-storage: pre-centrifugation;	Serum, lithium heparin and fluoride plasma; Fasting status not specified;	4ºC, 25ºC	0, 2, 4, 6, 24h	Clinical Chemistry / (T)	10	**Stable**.

			Serum reference: 0.5h; Plasma reference: centrifuged immediately					
	Jobard et al. 2016 [11]	Pre-storage: pre and ‡post-centrifugation;	Serum, heparin-plasma; Fasting; Reference (pre-centrifugation): 1h,22°C; Reference (post-centrifugation): 15min	Pre-centrifugation: 4°C, 22°C Post-centrifugation: 22°C	Pre-centrifugation: 1h (4°C), 6h (4°C, 22°C); Post-centrifugation: 15min, 1h	(^{1}H) NMR (1D, CPMG, NOESY, (^{1}H-^{13}C) HSQC, STOCSY, J-res) / (U)	96	Pre-storage/pre-centrifugation: **decrease** at 22°C, 6h (but Variable Importance in Projection <1); **stable** at 4°C, 1 and 6h. Pre-storage/‡post-centrifugation: **stable.**
	Current report	*Pre-storage: pre-centrifugation; Post-storage: post-centrifugation;*	*Serum, potassium EDTA-plasma; Non-fasting; Reference, pre-storage: 1.5h, 4°C; Reference, post-storage: no sample or NMR analysis delay*	*Pre-storage/ pre-centrifugation: 4°C, 21°C Post-storage/ post-centrifugation: 4°C*	*Pre-storage/ pre-centrifugation: 1.5, 24, 48h Post-storage/ post-centrifugation: 0, 24h*	*(^{1}H) NMR (1D, NOESY, CPMG), Nightingale Health* / (T)*	*Pre:23 Post:25*	*Pre-storage/pre-centrifugation: **stable**. Post-storage/post-centrifugation: mean **increase** up to 0.4SD.*
**Albumin**	Clark et al. 2003 [4]	Pre-storage: pre-centrifugation;	Potassium EDTA-plasma; Non-fasting; Reference: centrifuged immediately	4°C, 21°C	0, 1-4, 7 days	Clinical Chemistry / (T)	12	**Stable** (mean percentage change less than 0.5% per day)
	Boyanton et al. 2002 [5]	Pre-storage: pre and ‡post centrifugation;	Serum, lithium heparin-plasma; Non-fasting; Reference: 0.5, 25°C	25°C	0.5, 4, 8, 16, 24, 32, 40, 48, 56h	Clinical Chemistry / (T)	10	Pre-storage/pre-centrifugation: **Increase** after 24h of 7%. Pre-storage/‡post-centrifugation: **stable**.
	Oddoze et al. 2012 [6]	Pre-storage: pre-centrifugation;	Serum, lithium heparin and fluoride plasma; Fasting status not specified; Serum reference: 0.5h; Plasma reference: centrifuged immediately	4°C, 25°C	0, 2, 4, 6, 24h	Clinical Chemistry / (T)	10	**Stable**.
	Bernini et al. 2011[7]	Pre-storage: pre and ‡post-centrifugation;	Serum (SST), potassium EDTA-plasma; Fasting status not specified; Reference: centrifuged immediately (for serum after 30 min) and frozen immediately	Pre-centrifugation: 4°C, 25°C Post-centrifugation: 25°C	Pre-centrifugation: 0-4h Post-centrifugation: 0,6,12, 24h	(^{1}H) NMR (1D, NOESY CPMG) / (U)	Pre:6 Post:5	Pre-storage/pre-centrifugation: **stable;** Pre-storage/‡post-centrifugation: **decrease**.
	Current report	*Pre-storage: pre-centrifugation; Post-storage: post-centrifugation;*	*Serum, potassium EDTA-plasma; Non-fasting;*	*Pre-storage/ pre-centrifugation: 4°C, 21°C*	*Pre-storage/ pre-centrifugation: 1.5, 24, 48h*	*(^{1}H) NMR (1D, NOESY, CPMG), Nightingale Health* / (T)*	*Pre:23 Post:25*	*Pre-storage/pre-centrifugation: **stable** at 4°C; mean increase up to 0.6SD at 21°C;*

			Reference, pre-storage: 1.5h, 4ºC; Reference, post-storage: no sample or NMR analysis delay	*Post-storage/ post-centrifugation: 4ºC*	*Post-storage/ post-centrifugation: 0, 24h*			*Post-storage/post-centrifugation: **stable**.*
**Other untargeted NMR studies**								
Not applicable	ǂBarton et al. 2008[17]	Post-storage: ǂpost-centrifugation;	Serum; Fasting status not specified;	4ºC	0, 24, 36h	(^{1}H) NMR (1D, NOESY), ICL NPC* / (U)	40	Alterations in proteins and protein fragments.
Not applicable	Teahan et al. 2006[18]	Pre-storage: pre and ǂpost-centrifugation;	Serum (pre-centrifugation only), heparin-plasma; Non-fasting; Reference: 0.5h	pre-centrifugation: Ice, RT post-centrifugation: RT	0.5,1,2,3h	(^{1}H) NMR (1D, NOESY, CPMG), ICL NPC* / (U)	4	Pre-storage/pre-centrifugation: **stable** overall on ice. Pre-storage/ǂpost-centrifugation: **stable** overall. Changes in some lipids and possibly some low-molecular-weight metabolites.

References

1. Gao, X.; Starmer, J.; Martin, E.R. A multiple testing correction method for genetic association studies using correlated single nucleotide polymorphisms. *Genet Epidemiol* **2008**, *32*, 361-369, doi:10.1002/gepi.20310.
2. Bro, R.; Smilde, A.K. Principal component analysis. *Anal Methods-Uk* **2014**, *6*, 2812-2831, doi:10.1039/c3ay41907j.
3. Key, T.; Oakes, S.; Davey, G.; Moore, J.; Edmond, L.M.; McLoone, U.J.; Thurnham, D.I. Stability of vitamins A, C, and E, carotenoids, lipids, and testosterone in whole blood stored at 4 degrees C for 6 and 24 hours before separation of serum and plasma. *Cancer Epidem Biomar* **1996**, *5*, 811-814.
4. Clark, S.; Youngman, L.D.; Palmer, A.; Parish, S.; Peto, R.; Collins, R. Stability of plasma analytes after delayed separation of whole blood: implications for epidemiological studies. *Int J Epidemiol* **2003**, *32*, 125-130.
5. Boyanton, B.L., Jr.; Blick, K.E. Stability studies of twenty-four analytes in human plasma and serum. *Clin Chem* **2002**, *48*, 2242-2247.
6. Oddoze, C.; Lombard, E.; Portugal, H. Stability study of 81 analytes in human whole blood, in serum and in plasma. *Clin Biochem* **2012**, *45*, 464-469, doi:10.1016/j.clinbiochem.2012.01.012.
7. Bernini, P.; Bertini, I.; Luchinat, C.; Nincheri, P.; Staderini, S.; Turano, P. Standard operating procedures for pre-analytical handling of blood and urine for metabolomic studies and biobanks. *J Biomol NMR* **2011**, *49*, 231-243, doi:10.1007/s10858-011-9489-1.
8. Anton, G.; Wilson, R.; Yu, Z.H.; Prehn, C.; Zukunft, S.; Adamski, J.; Heier, M.; Meisinger, C.; Romisch-Margl, W.; Wang-Sattler, R., et al. Pre-analytical sample quality: metabolite ratios as an intrinsic marker for prolonged room temperature exposure of serum samples. *PloS one* **2015**, *10*, e0121495, doi:10.1371/journal.pone.0121495.

9. Pinto, J.; Domingues, M.R.; Galhano, E.; Pita, C.; Almeida Mdo, C.; Carreira, I.M.; Gil, A.M. Human plasma stability during handling and storage: impact on NMR metabolomics. *Analyst* **2014**, *139*, 1168-1177, doi:10.1039/c3an02188b.
10. Breier, M.; Wahl, S.; Prehn, C.; Fugmann, M.; Ferrari, U.; Weise, M.; Banning, F.; Seissler, J.; Grallert, H.; Adamski, J., et al. Targeted Metabolomics Identifies Reliable and Stable Metabolites in Human Serum and Plasma Samples. *PloS one* **2014**, *9*, doi:ARTN e89728

10.1371/journal.pone.0089728.
11. Jobard, E.; Tredan, O.; Postoly, D.; Andre, F.; Martin, A.L.; Elena-Herrmann, B.; Boyault, S. A Systematic Evaluation of Blood Serum and Plasma Pre-Analytics for Metabolomics Cohort Studies. *Int J Mol Sci* **2016**, *17*, doi:10.3390/ijms17122035.
12. Kamlage, B.; Maldonado, S.G.; Bethan, B.; Peter, E.; Schmitz, O.; Liebenberg, V.; Schatz, P. Quality markers addressing preanalytical variations of blood and plasma processing identified by broad and targeted metabolite profiling. *Clin Chem* **2014**, *60*, 399-412, doi:10.1373/clinchem.2013.211979.
13. Fliniaux, O.; Gaillard, G.; Lion, A.; Cailleu, D.; Mesnard, F.; Betsou, F. Influence of common preanalytical variations on the metabolic profile of serum samples in biobanks. *Journal of Biomolecular Nmr* **2011**, *51*, 457-465, doi:10.1007/s10858-011-9574-5.
14. Bervoets, L.; Louis, E.; Reekmans, G.; Mesotten, L.; Thomeer, M.; Adriaensens, P.; Linsen, L. Influence of preanalytical sampling conditions on the H-1 NMR metabolic profile of human blood plasma and introduction of the Standard PREanalytical Code used in biobanking. *Metabolomics* **2015**, *11*, 1197-1207, doi:10.1007/s11306-015-0774-y.
15. Trezzi, J.P.; Bulla, A.; Bellora, C.; Rose, M.; Lescuyer, P.; Kiehntopf, M.; Hiller, K.; Betsou, F. LacaScore: a novel plasma sample quality control tool based on ascorbic acid and lactic acid levels. *Metabolomics* **2016**, *12*, 96, doi:10.1007/s11306-016-1038-1.
16. Nishiumi, S.; Suzuki, M.; Kobayashi, T.; Yoshida, M. Differences in metabolite profiles caused by pre-analytical blood processing procedures. *J Biosci Bioeng* **2018**, *125*, 613-618, doi:10.1016/j.jbiosc.2017.11.011.
17. Barton, R.H.; Nicholson, J.K.; Elliott, P.; Holmes, E. High-throughput 1H NMR-based metabolic analysis of human serum and urine for large-scale epidemiological studies: validation study. *Int J Epidemiol* **2008**, *37 Suppl 1*, i31-40, doi:10.1093/ije/dym284.
18. Teahan, O.; Gamble, S.; Holmes, E.; Waxman, J.; Nicholson, J.K.; Bevan, C.; Keun, H.C. Impact of analytical bias in metabonomic studies of human blood serum and plasma. *Anal Chem* **2006**, *78*, 4307-4318, doi:10.1021/ac051972y.
